# CIA‐II is associated with lower‐grade glioma survival and cell proliferation

**DOI:** 10.1111/cns.14340

**Published:** 2023-07-14

**Authors:** Feng Xiao, Hong Zhu, Yun Guo, Zhe Zhang, Gufeng Sun, Yao Xiao, Guowen Hu, Kai Huang, Hua Guo

**Affiliations:** ^1^ Department of Neurosurgery The Second Affiliated Hospital of Nanchang University Nanchang China; ^2^ Jiangxi Key Laboratory of Neurological Tumors and Cerebrovascular Diseases Nanchang China; ^3^ Jiangxi Health Commission Key Laboratory of Neurological Medicine Nanchang China; ^4^ Institute of Neuroscience Nanchang University Nanchang China

**Keywords:** cell proliferation, chemotherapeutics, CIA‐II, immune cell infiltration, lower‐grade glioma, prognosis

## Abstract

**Background:**

The role of CIA‐II has been clarified in several types of tumors; however, whether dysregulated CIA‐II expression is also involved in the pathophysiology of lower‐grade glioma (LGG) remains undisclosed.

**Methods:**

A comprehensive pan‐cancer analysis of the expression patterns and prognostic significance of CIA‐II in miscellaneous tumors was undertaken. Subsequently, a detailed bioinformatics analysis was executed to identify putative correlations between CIA‐II expression and clinical features, prognosis, biological functions, immunological characteristics, genomic alterations, and chemotherapeutics in LGG. In vitro studies were implemented to examine the potential roles of CIA‐II in LGG.

**Results:**

CIA‐II expression was found to be abnormally elevated in a variety of tumors, including LGG. Additionally, patients with LGG with higher CIA‐II expression owned worse prognosis. Importantly, the results declared that CIA‐II expression was an independent prognostic indicator for LGG. Moreover, the expression of CIA‐II was tightly interrelated with immune cell infiltration, gene mutations, and chemotherapeutics in LGG. In vitro studies revealed that CIA‐II was increased and strongly related to the cell proliferation in LGG.

**Conclusion:**

CIA‐II may be an independent prognostic factor and a serviceable therapeutic target in LGG.

## Graphical abstract

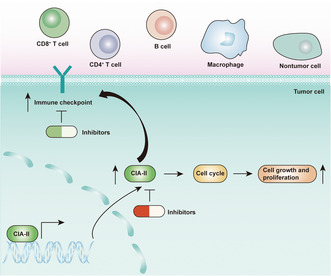

## 1 INTRODUCTION

Gliomas are the most commonly diagnosed intracranial neoplasms.^1^ They are classified from grade I to IV according to World Health Organization (WHO)‐defined standard.^2^ Grade II and III gliomas are considered as LGGs by The Cancer Genome Atlas (TCGA).^3^ Despite the substantial progress in the development of new anticancer therapeutics, drugs for the therapy of LGG remain scarce, and the prognosis of LGG patients continues to be unfavorable. In consequence, it is imperative to discover novel therapeutic targets for LGG patients.

CIA‐II, also known as antisilencing function 1 B, is one of paralogous forms of antisilencing function 1. CIA‐II is a histone chaperone with a critical part in cell proliferation and cell death.^4^, ^5^ Numerous studies have implicated dysregulated CIA‐II expression in the malignant progression of multiple cancers, including cervical, lung, liver, and pancreatic cancers.^6^, ^7^, ^8^, ^9^ For example, one particular study has demonstrated that CIA‐II may promote the malignant development of cervical cancer by stabilizing the expression of CDK9.^6^ Additionally, CIA‐II was tightly interrelated with the cell cycle and proliferation in hepatocellular carcinoma.^8^ In pancreatic cancer, the capacity of cell proliferation and the cell cycle distribution was affected after knocking down the expression of CIA‐II.^10^ Interestingly, it has been reported that CIA‐II played a crucial part in the tumor microenvironment (TME) by regulating the infiltration of immune cells.^11^ However, the underlying roles of CIA‐II in LGG remain unknown. Thus, we implemented this research to inspect the potential functions of CIA‐II in LGG patients.

To check the prognostic ability of CIA‐II in LGG, we undertook a bioinformatic analysis of CIA‐II in cohorts from TCGA and the Chinese Glioma Genome Atlas (CGGA). LGG samples were separated into low‐CIA‐II and high‐CIA‐II expression subtypes in line with the median value of CIA‐II expression. The high‐CIA‐II subtype was found to possess worse survival than the low‐CIA‐II subtype in both datasets. We also inspected the independent prognostic value of CIA‐II and its correlation with clinical characteristics of LGG patients. Functional enrichment analysis was exploited to appraise the underlying molecular mechanisms of CIA‐II in LGG. We further identified the relationship between 13 immune‐related gene signatures and CIA‐II expression and detected the connection between CIA‐II expression and immunological features (including immune checkpoint genes [ICPGs] expression, stromal and immune scores, and tumor‐infiltrating immune cells [TIICs]), gene variations, and chemotherapeutics. In vitro studies were also performed to confirm that the CIA‐II was increased and strictly interrelated with the cell proliferation in LGG. Our findings suggested that CIA‐II may become a prognostic factor and underlying therapeutic target for LGG patients.

## 2 MATERIALS AND METHODS

### 2.1 Data sources and management

**FIGURE 1:**
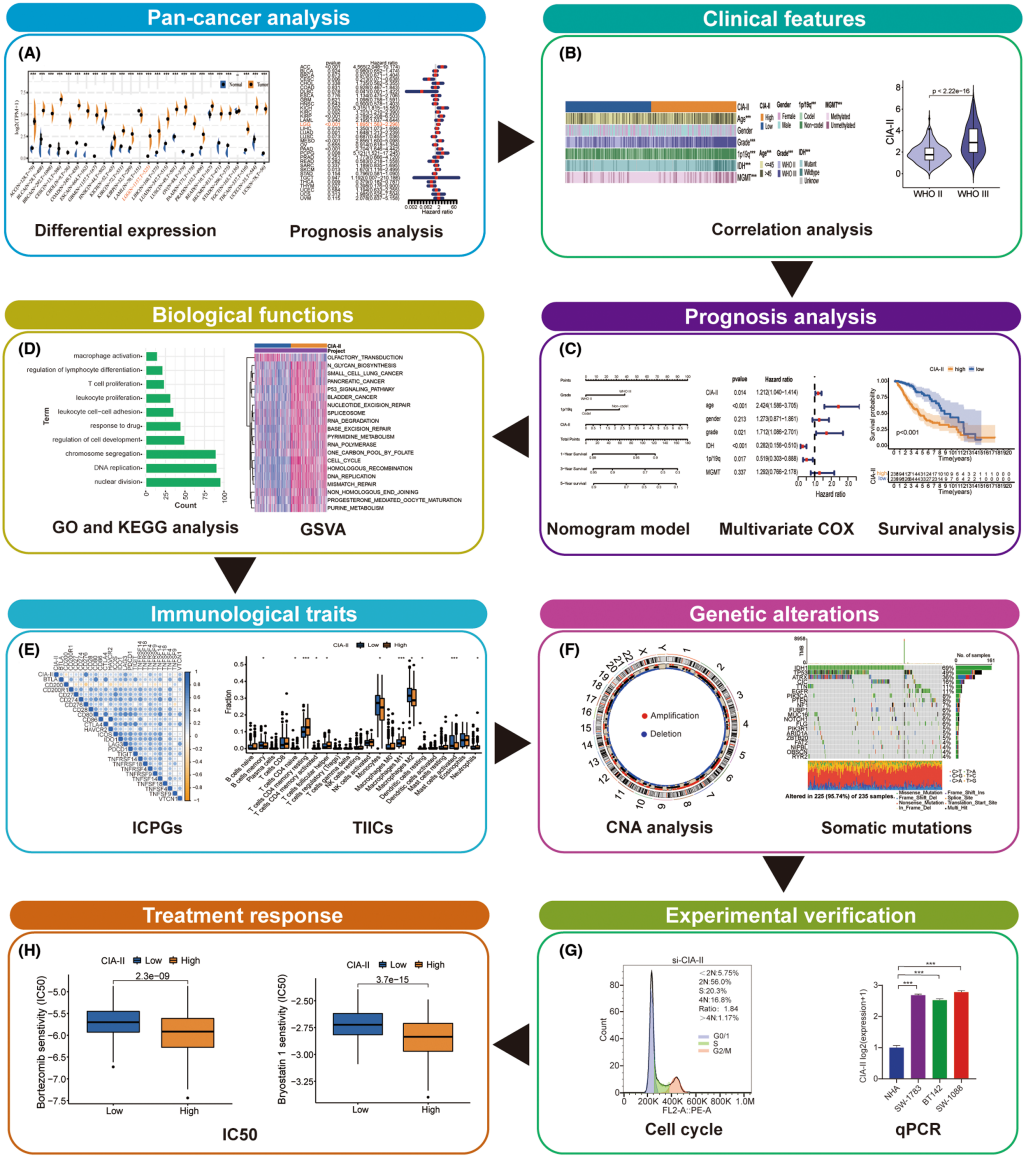
Flow diagram of the study. (A) Pan‐cancer analysis. (B) Clinical features. (C) Prognosis analysis. (D) Biological functions. (E) Immunological traits. (F) Genetic alterations. (G) Experimental verification. (H) Treatment response of CIA‐II in LGG.

The flow chart of the entire study is displayed in Figure 1. The clinical, survival, mRNA expression, and tumor mutation burden (TMB) data for 33 cancer types were obtained from TCGA database. CIA‐II expression data in normal tissue were acquired from the Genotype‐Tissue Expression (GTEx) database.

Two independent LGG cohorts—TCGA and CGGA (CGGA_325)—were used in this research. The clinical, survival, and mRNA expression data for LGG patients were gathered from TCGA and CGGA databases. The mRNA expression data were obtained in Fragments per Kilobase Million format and then transformed into Transcripts per Kilobase Million values by implementing a previously reported algorithm.^12^, ^13^ Afterward, these values were transformed by log2 for further analysis. Gene alteration information of LGG were gathered from TCGA database.

### 2.2 Inclusion criteria for patients

We employed the following inclusion criteria: LGG patients with mRNA sequencing data, WHO grade II and III information, and OS longer than 30 days. In total, 477 LGG patients were attained from TCGA database (Table S1) and 170 from the CGGA database (Table S2). To guarantee consistency among the 33 tumor types, LGG patients with OS shorter than 30 days were included in the pan‐cancer analysis of CIA‐II.

### 2.3 Prognostic value of CIA‐II and validation

LGG patients in TCGA and CGGA cohorts were categorized into low‐CIA‐II expression and high‐CIA‐II expression subtypes in line with the median value of CIA‐II expression. We implemented Kaplan–Meier curve, receiver operating characteristic (ROC) curve, and area under the curve (AUC) analysis to check the correctness of CIA‐II expression in predicting LGG prognosis among the samples in the two datasets. Cox regression analysis was executed to evaluate the independent prognostic value of CIA‐II expression in LGG.

### 2.4 Establishment and confirmation of the clinical nomogram model

The clinical nomogram model was built according to the results of the Cox regression analysis in the TCGA and CGGA datasets using the R package “rms”. Clinical features such as CIA‐II expression, 1p/19q status, and WHO grade were included in the nomogram model. Additionally, calibration curves were generated in the “rms” package for TCGA dataset and confirmed in CGGA cohort.

### 2.5 Functional annotations

A gene set variation analysis (GSVA) was undertaken to determine the majorly enriched molecular pathways between the low‐CIA‐II and high‐CIA‐II subsets using the R package “GSVA”.^14^ Enriched pathways were obtained in the light of the KEGG (c2.cp.kegg.v7.2.symbols) gene set (|log2 [fold change] > 0.5 and the false discovery rate (FDR) < 0.05).

We ascertained differentially expressed genes (DEGs) between the low‐CIA‐II and high‐CIA‐II subsets by conducting the R package “limma” (|log2 [fold change] > 0.5 and FDR < 0.05).^15^ In TCGA and CGGA datasets, 1430 (Table S3) and 3127 (Table S4) DEGs were filtered out, respectively, and Gene Ontology biological process (GO‐BP) and Kyoto Encyclopedia of Genes and Genomes (KEGG) enrichment analyses of the obtained DEGs was conducted in R package “clusterProfiler”.^16^

### 2.6 Co‐expression analysis of CIA‐II

A correlation analysis was undertaken in TCGA cohort to screen out the genes most linked to CIA‐II expression using the R package “circlize” (cor > 0.6, *p* < 0.001).^17^ Afterward, we further verified the results in CGGA dataset. Co‐expression analysis was employed to examine the connection between CIA‐II and 25 ICPGs selected from previous studies^18^ in 33 cancer types using the R package “reshape2”. In both cohorts, we also implemented co‐expression analysis to further detect the connection between CIA‐II and the 25 selected ICPGs in LGG.

### 2.7 Immunological features of LGG

LGG patients were separated into high‐CIA‐II and low‐CIA‐II subsets according to the median expression of CIA‐II. The ssGSEA algorithm was implemented to examine the differential abundance of 13 previously identified immune‐connected indicators^19^ between the two subsets. To inspect the composition of the TME and forecast tumor purity, the enrichment of stromal and immune cells was determined employing the ESTIMATE algorithm.^20^ The stromal score (representing the abundance of stromal cells), the immune score (representing the abundance of immune cells), the ESTIMATE score (representing nontumor composites), and tumor purity were generated by conducting the ESTIMATE algorithm. Additionally, the CIBERSORT algorithm was implemented to compare the infiltration level of 22 types of TIICs between the two subsets.

### 2.8 Genomic mutation analysis

Circos plots were generated to visualize chromosome copy number variation in the low‐CIA‐II and high‐CIA‐II subtypes by implementing the R package “RCircos”.^21^ A correlation analysis was excavated to identify the interrelation between the TMB and CIA‐II expression in 33 types of cancers by exploiting the R package “fmsb”. Meanwhile, the R package ggplot2 was executed to conduct correlation analysis between the TMB and the CIA‐II expression level in TCGA dataset. Waterfall plots were generated to display the frequencies of genes mutations and mutation types in the two subtypes using R package “maftools”.^22^

### 2.9 Ethical approval

This protocol was approved by the medical ethics committee of the Second Affiliated Hospital of Nanchang University (NO. Review [2021] NO. [033]). Informed consent to participate in this research were acquired from LGG patients.

### 2.10 Cell culture and transfection

We purchased three LGG lines, including SW1088, SW1783, and BT142, from the American Type Culture Collection and normal human astrocyte (NHA) cell line from Culture Collection of the Chinese Academy of Sciences (Shanghai, China). SW‐1783 and SW‐1088 cell lines were cultured with Leibovitz's L‐15 medium with 10% fetal bovine serum (Gibco). Dulbecco's modified Eagle's medium/F12 medium was utilized to incubate BT142 and NHA cell lines. These cell lines incubation conditions are as follows: 5% CO_2_ and 37°C. SW1088 cells were transfected with lentiviral vector containing CIA‐II shRNA (5′‐CCCACTCAACTGCACTCCTAT‐3′) and negative control (NC) vector at a multiplicity of infection of 10. The puromycin was utilized to select out positive cells.

### 2.11 Western blot analysis

Brain tissue samples were achieved from the Second Affiliated Hospital of Nanchang University. We extracted total protein by exploiting radioimmunoprecipitation assay buffer (Solarbio) with protease inhibitors. Afterward, we separated protein by utilizing 10% SDS–PAGE and transferred onto PVDF membranes, incubated with primary antibodies targeting CIA‐II (1:1000; AF9022, Proteintech) and β‐actin (1:10,000; 66009‐1‐lg, Proteintech), and then with the corresponding secondary antibodies. Ultimately, the membranes were displayed by operating the GV6000M imaging system (GelView 6000pro). Protein band intensity was quantified with ImageJ.

### 2.12 Quantitative real‐time PCR (qRT‐PCR)

Total RNA was extracted from cells using the Simply P Total RNA Extraction Kit (Bioflux) and then reverse‐transcribed into cDNA using HiScript III‐RT SuperMix (Vazyme). The primer sequences were as follows: CIA‐II forward 5′‐TCATCACCTGCACCTACCAT‐3′ and reverse 5′‐ AGCCTGTCCATGTTGTC‐3′; β‐actin forward 5′‐TGACGTGGACATCCGCAAAG‐3′ and reverse 5′‐CTGGAAGGTGGACAGCGAGG‐3′.

### 2.13 CCK‐8 assay

We seeded the transfected cells in 96‐well plates at 2 × 10^3^/well and incubated for 0, 24, 48, 72, 96 h. Next, we evaluated the cell proliferation by Cell Counting Kit 8 assay (Glpbio) according to the protocol.

### 2.14 Colony formation assay

We plated 2 × 10^3^ cells per well in 6‐well plates and incubated for 2 weeks. Afterward, we stained the cells with 0.1% crystal violet stain solution and quantified the number of colonies using ImageJ.

### 2.15 EdU assay

We plated transfected cells (2 × 10^4^) in 24‐well plates. After incubating for 3 days, cells were treated with EdU reagent for 2 h. The 4% paraformaldehyde and 0.5% Triton X‐100 were used to fix the cells. The Hoechst staining was employed to stain the cells. We counted the EdU incorporation rate by ImageJ.

### 2.16 Cell cycle analysis

The transfected cells were fixed with 70% ethanol and stored at 4°C overnight. Next, the fixed SW1088 cells were resuspended in 500 μL of PI/RNase Staining Buffer, and then examined by flow cytometry.

### 2.17 Evaluation of CIA‐II expression and chemotherapeutics

The R package “pRRophetic” was employed to inspect the differences in sensitivity to several chemotherapeutic drugs, such as the PI3K/AKT inhibitors A‐443654, AKT inhibitor VIII, AS605240, ZSTK474, and CAL‐101; the MAPK inhibitor AP‐24534; the proteasome inhibitors bryostain 1 and bortezomib, between the low‐CIA‐II and high‐CIA‐II subtypes.^23^

### 2.18 Statistics

Kaplan–Meier analysis was executed to contrast the prognosis between the low‐CIA‐II and high‐CIA‐II subtypes of LGG. The accuracy of CIA‐II expression in predicting prognosis was examined by exploiting ROC curves and AUC values. Additionally, cox regression analyses were performed to estimate the independent prognostic value of CIA‐II. The Student's *t*‐test was used to explore the distinct levels of the immune‐associated biomarkers (including 25 ICPGs, 13 immune‐related signatures, TIICs, TMB, and CNA burden) between the two subtypes. Correlations between distributed variables were ascertained by exploiting Pearson's or Spearman's correlation tests. In addition, the Wilcoxon signed‐rank test was exploited to detect the difference in sensitivity to anticancer drugs between the low‐CIA‐II and high‐CIA‐II subtypes. In vitro experiments, two‐tailed Student's *t*‐tests were applied for comparisons between two groups. Two‐way ANOVA followed by Tukey's tests was applied for multiple comparisons. Statistical analysis was implemented in R version 4.1.0, GraphPad Prism 8, and ImageJ. *p*‐values < 0.05 were considered as significant.

## 3 RESULTS

### 3.1 Pan‐cancer analysis of CIA‐II expression

**FIGURE 2:**
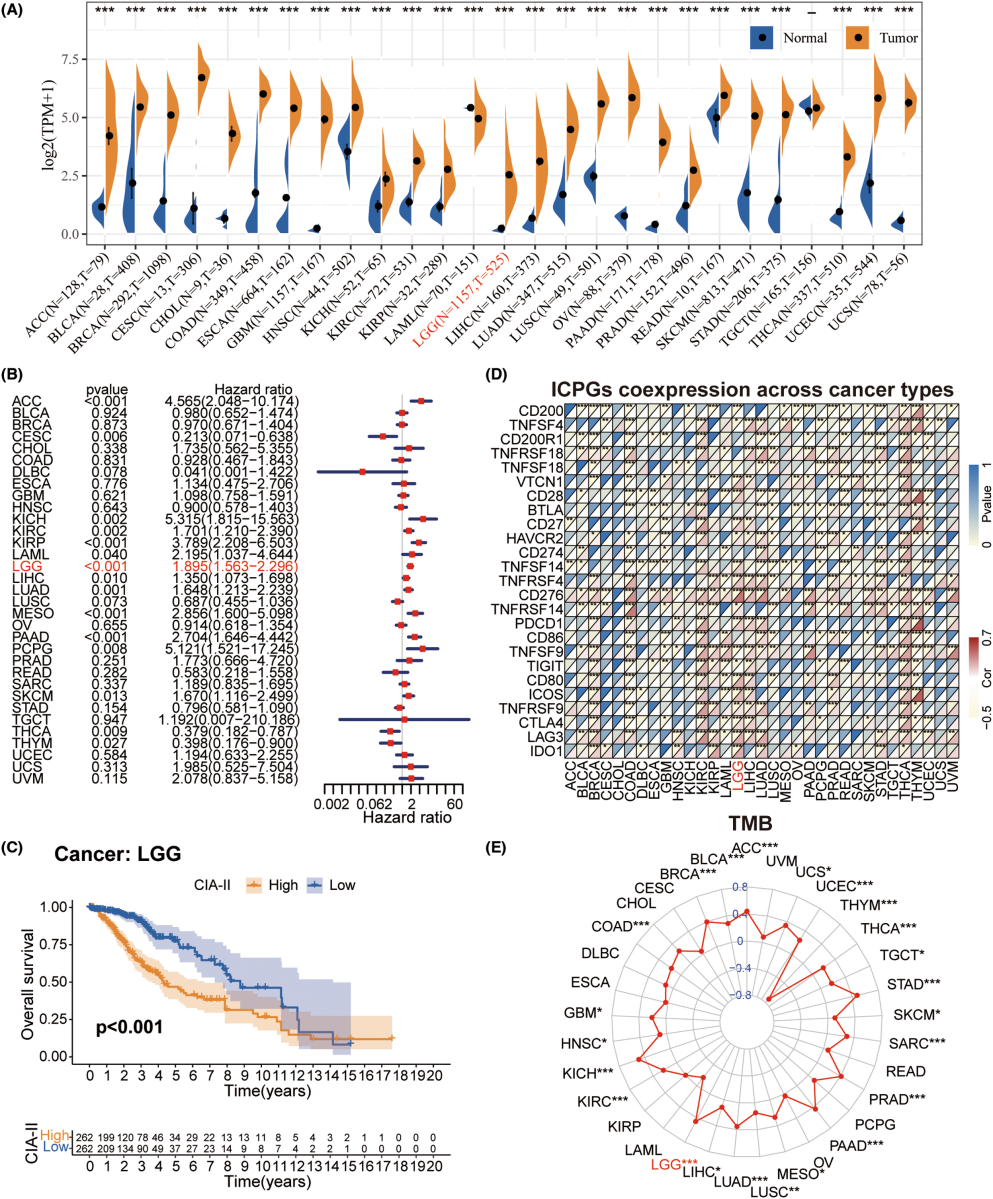
Pan‐cancer analysis of CIA‐II expression in 33 types of tumors. (A) The differential expression of CIA‐II in multiple tumor tissues and corresponding normal tissues. (B) Univariate Cox regression analysis of CIA‐II expression in various tumors. (C) Kaplan–Meier analysis of CIA‐II in pan‐lower‐grade glioma (LGG). (D) Co‐expression of CIA‐II and immune checkpoint genes (ICPGs) across diverse tumors. (E) Differences in the TMB in different tumors (*p < 0.05, **p < 0.01, ***p < 0.001).

Obvious differences in CIA‐II expression were found between numerous types of cancer (TCGA database) and normal tissue (GTEx database). The CIA‐II expression level was distinctly higher in 25 of 27 cancers assessed than in normal tissues; however, the opposite was seen in LAML (Figure 2A).

To check the prognostic significance of CIA‐II expression in 33 tumors, we inspected the interrelation between CIA‐II expression and OS by conducting univariate Cox regression analysis. CIA‐II was found to be elevated in ACC, CESC, KICH, KIRC, KIRP, LAML, LGG, LIHC, LUAD, MESO, PAAD, PCPG, SKCM, THCA, and THYM (Figure 2B). Interestingly, subsequent survival analyses also imported that LGG patients with higher CIA‐II expression owned worse prognosis (Figure 2C).

Afterward, we detected the conjunction between CIA‐II expression and the expression of ICPGs in 33 tumors. The greatest level of CIA‐II/ICPGs co‐expression was found in BRCA, COAD, GBM, KIRC, KIRP, LAML, LGG, LIHC, LUAD, LUSC, PAAD, PRAD, READ, SKCM, STAD, THCA, THYM, and UCEC (Figure 2D). We also detected the conjunction between CIA‐II expression and the TMB. In ACC, BLCA, BRCA, COAD, GBM, HNSC, KICH, KIRC, LGG, LIHC, LUAD, LUSC, MESO, PAAD, PRAD, SARC, SKCM, STAD, TGCT, THCA, THYM, UCEC, and UCS, CIA‐II expression was positively linked to the TMB level, whereas the opposite was true in THYM (Figure 2E).

### 3.2 The conjunction between CIA‐II and clinical traits in LGG

**FIGURE 3:**
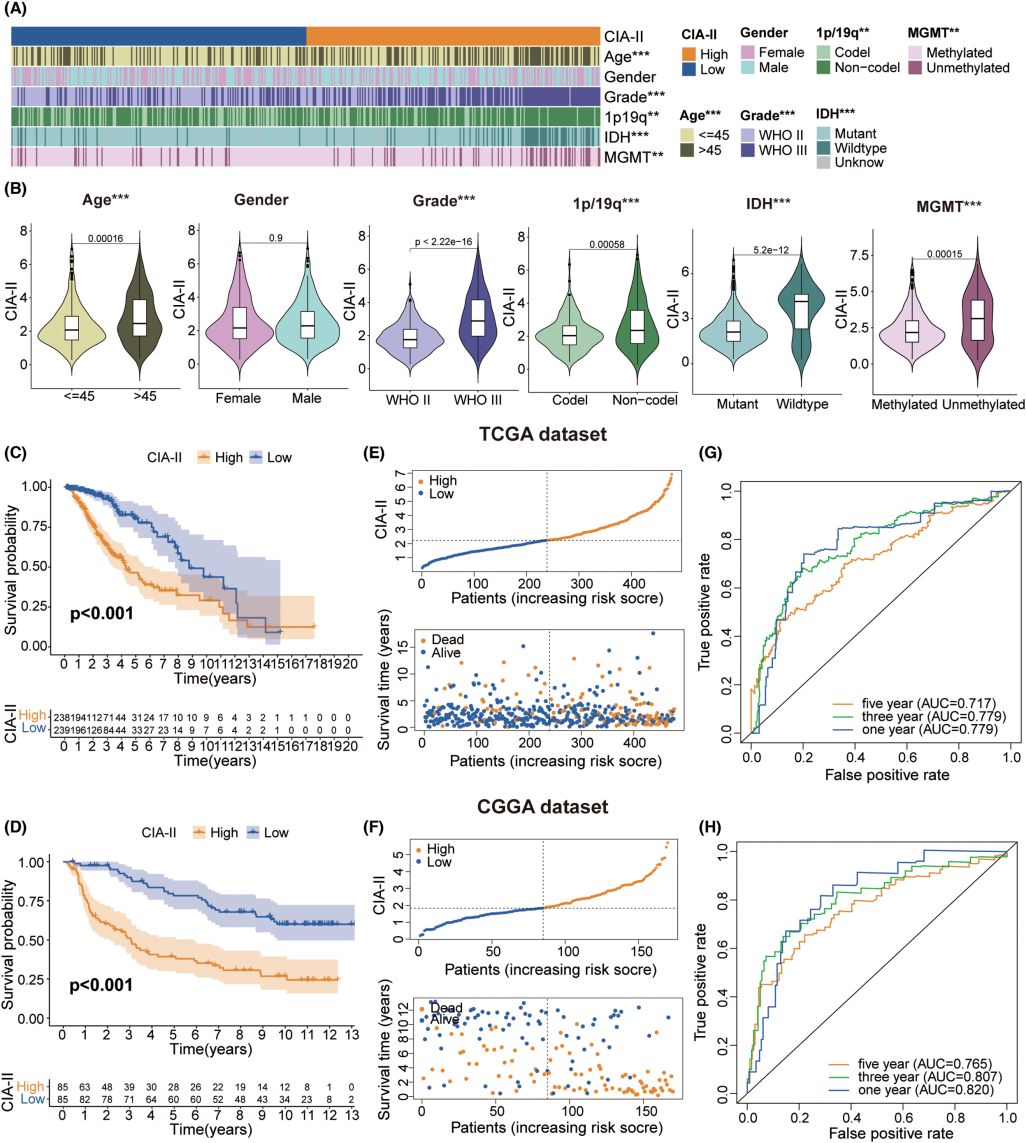
Correlation analysis between CIA‐II and the clinical characteristics of patients with LGG. (A) Association between CIA‐II expression and the clinical characteristics of LGG in TCGA dataset. (B) Analysis of the correlation between CIA‐II expression and clinical characteristics in TCGA dataset. (C, D) Analysis of the prognostic value of the low‐CIA‐II and high‐CIA‐II expression subtypes in TCGA (C) and CGGA (D) datasets. (E, F) Distribution of the risk score, OS, and OS status of the low‐CIA‐II and high‐CIA‐II subtypes in TCGA (E) and CGGA (F) cohorts. (G, H) ROC curves depicting the predictive value of the risk score in TCGA (G) and CGGA (H) datasets (*p < 0.05, **p < 0.01, ***p < 0.001).

We detected the connection between the CIA‐II expression level and clinical traits (age, gender, WHO grade, isocitrate dehydrogenase [IDH] mutation status, 1p/19q codeletion status, and 6‐*O*‐methylguanine‐DNA methyltransferase [MGMT] promoter methylation status) in LGG. We found that higher CIA‐II expression was tightly interrelated with higher WHO grade, IDH wildtype status, unmethylated MGMT promoter status, older age, and 1p/19q noncodeletion status in TCGA cohort (Figure 3A,B). Elevated CIA‐II expression was also found to be correlated with higher WHO grade, 1p/19q noncodeletion status, and IDH wildtype status in CGGA cohort (Figure S1A,B). Moreover, we further evaluated the clinical characteristics of LGG patients between the two subsets in both TCGA (Figure S2A) and CGGA (Figure S2B) datasets. These results confirmed that CIA‐II had considerable correlations with the clinical traits of LGG patients.

### 3.3 High‐CIA‐II mRNA expression correlates with bad prognosis

Survival analysis affirmed that the prognosis of patients in low‐CIA‐II subset was markedly better than in high‐CIA‐II subset in both TCGA (Figure 3C) and CGGA (Figure 3D) cohorts. Afterward, we examined the differences in OS between the two subsets stratified by crucial clinical traits (age, WHO grade, and IDH status) in both cohorts. The results demonstrated that, except for the WHO grade II cohort in TCGA dataset, OS was greater in low‐CIA‐II subset than in high‐CIA‐II subset (Figure S1 and Figure 2C). Additionally, we inspected the interrelation between CIA‐II expression, OS, and risk score in LGG patients, and found that higher CIA‐II expression was correlated with reduced OS and higher risk score in both TCGA (Figure 3E) and CGGA (Figure 3F) cohorts. To verify the accuracy of CIA‐II expression in estimating the OS of LGG patients in the TCGA and CGGA datasets, we performed ROC curve analysis. The AUC values for 1/3/5‐year OS were 0.779, 0.779, and 0.717, respectively, in TCGA cohort (Figure 3G) and 0.820, 0.807, and 0.765, respectively, in CGGA cohort (Figure 3H). Thus, CIA‐II may be a strong prognostic factor for LGG patients.

### 3.4 Independent prognostic value of CIA‐II

To assess whether CIA‐II was an independent prognostic biomarker in LGG patients, Cox regression analyses were employed. In TCGA dataset, we detected that CIA‐II expression, age, WHO grade, IDH status, and 1p/19q status were independent prognostic indicators in LGG patients (Figure S3A). In CGGA dataset, meanwhile, CIA‐II expression, WHO grade, and 1p/19q status were the independent prognostic indicators (Figure S3B). These differences may be related to incomplete information relating to IDH, 1p/19q, and MGMT status in the clinical samples. These results imply that CIA‐II has potential as an independent prognostic biomarker in LGG patients.

### 3.5 Creation and confirmation of the nomogram model

To identify the potential clinical prognostic ability of CIA‐II in LGG, we established a nomogram model using CIA‐II expression, WHO grade, and 1p/19q status data obtained with the common results of the multivariate Cox regression analyses in both TCGA and CGGA datasets (Figure S3C). The calculated scores were used for forecasting 1/3/5‐year OS among LGG patients and a calibration plot was employed to examine the exactness of the nomogram model in estimating the prognosis. The results demonstrated that the nomogram model possessed a high precision in forecasting 1/3/5‐year OS in LGG patients (Figure S3D,E). These findings demonstrated that the established nomogram model may be implemented to accurately estimate the prognosis of LGG patients.

### 3.6 Functional annotations of CIA‐II

**FIGURE 4:**
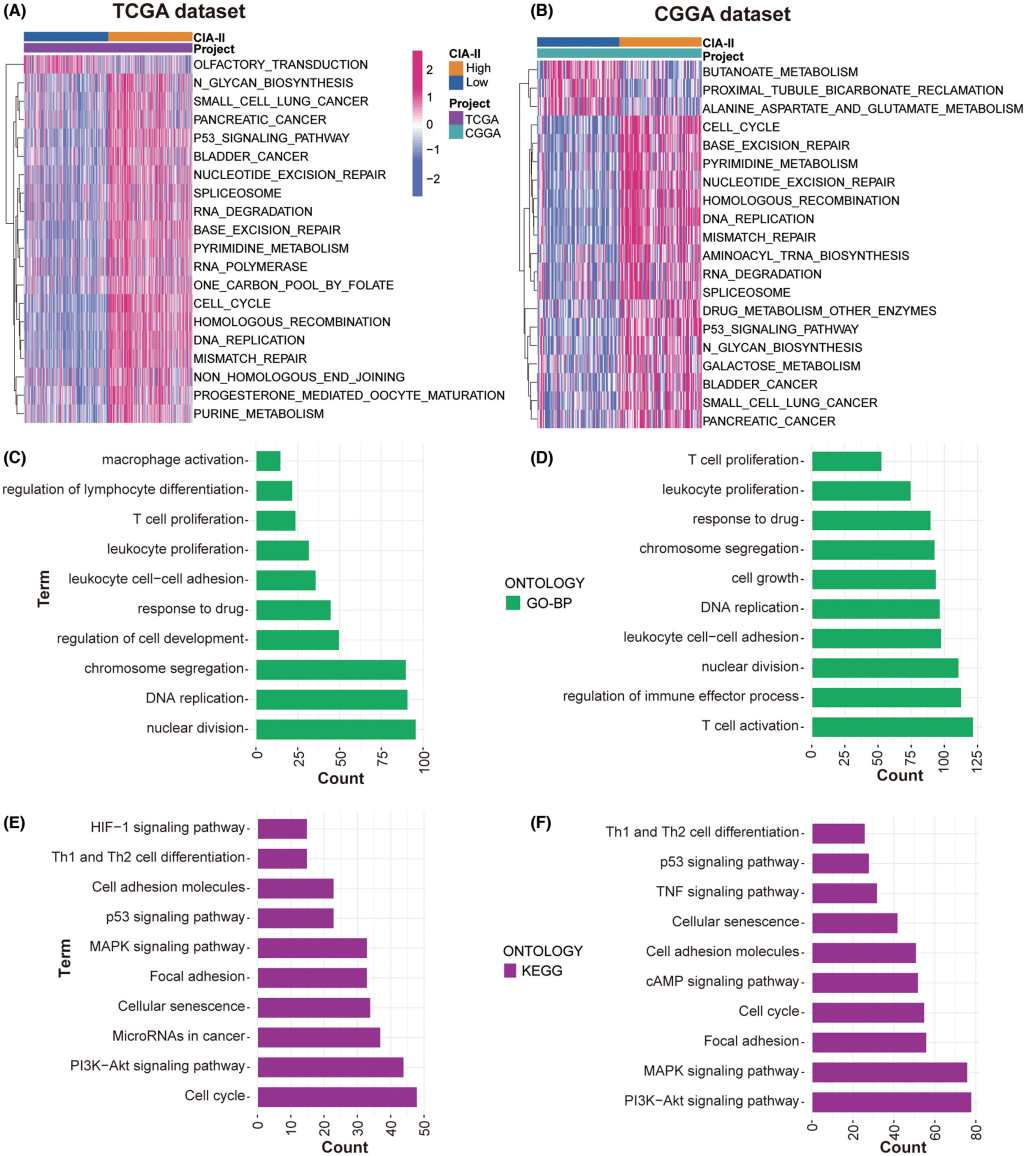
Biological functions of CIA‐II in low‐grade glioma (LGG) in The Cancer Genome Atlas (TCGA) and Chinese Glioma Genome Atlas (CGGA) datasets. (A, B) CIA‐II‐related GSVA in patients with LGG in TCGA (A) and CGGA (B) datasets. (C, D) GO‐BP analysis of DEGs according to CIA‐II expression levels in patients with LGG in TCGA (C) and CGGA (D) datasets. (E, F) KEGG analysis of DEGs according to CIA‐II expression levels in patients with LGG in TCGA (E) and CGGA (F) datasets.

To detect the molecular mechanisms interrelated with the differential expression of CIA‐II, we conducted a GSVA analysis using the TCGA (Figure 4A) and CGGA (Figure 4B) datasets. The high‐CIA‐II subtype was found to be mainly associated with mitogenic pathways such as the p53 signaling pathway, RNA degradation, DNA replication, and cell cycle in both datasets.

To ascertain the effect of differential CIA‐II expression on the OS of LGG patients, a differential expression analysis was implemented. A total of 1430 and 3127 DEGs were screened out in TCGA and CGGA datasets, respectively. Subsequently, these DEGs were applied to GO‐BP and KEGG enrichment analyses. The results of GO‐BP analysis declared that these DEGs were closely interrelated with nuclear division, DNA replication, chromosome segregation, T‐cell proliferation, leukocyte proliferation, and leukocyte cell–cell adhesion in the TCGA (Figure 4C) and CGGA (Figure 4D) datasets. The results of the KEGG analysis illustrated that these DEGs were tightly linked to cell cycle, the PI3K‐AKT, MAPK, and p53 signaling pathways, and cell adhesion molecules in the TCGA and CGGA datasets (Figure 4E,F, respectively). These results provide some clues regarding the molecular mechanisms underlying the value of CIA‐II in LGG.

### 3.7 Analysis of the connection between CIA‐II and related genes

**FIGURE 5:**
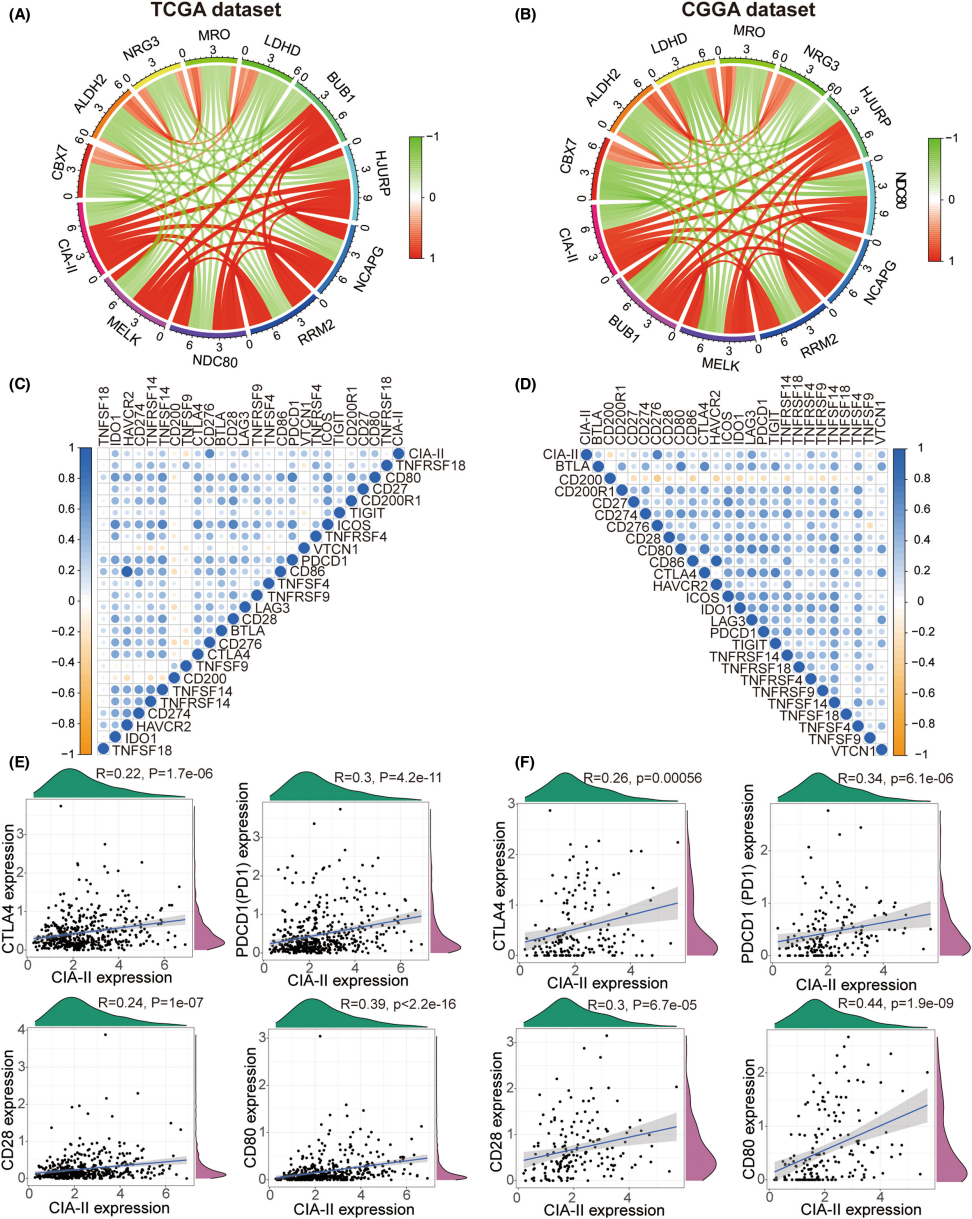
Co‐expression of CIA‐II in LGG in TCGA and CGGA cohorts. (A, B) Circos plots showing the genes most positively or negatively linked to CIA‐II expression in TCGA (A) and CGGA (B) cohorts. (C, D) Co‐expression analysis of CIA‐II and 25 immune checkpoint genes (ICPGs) in TCGA (C) and CGGA (D) datasets. (E, F) Correlation analysis between the expression levels of CIA‐II and those of four common ICPGs in TCGA (E) and CGGA (F) datasets (*p < 0.05, **p < 0.01, ***p < 0.001).

To identify genes that are tightly linked to CIA‐II expression, we carried out a correlation analysis using TCGA cohort (cor > 0.6, *p* < 0.001). Circos plots showed that CIA‐II was most positively associated with *MELK*, *NDC80*, *RRM2*, *NCAPG*, *HJURP*, and *BUB1* and negatively associated with *CBX7*, *ALDH2*, *NRG3*, *MRO*, and *LDHD* (Figure 5A). Analogical outcomes were acquired for the CGGA dataset (Figure 5B).

Next, we undertook a correlation analysis to examine the differences in ICPGs expression between the two subsets in the TCGA and CGGA datasets. The results disclosed that CIA‐II expression was positively linked to most ICPGs in the TCGA (Figure 5C) and CGGA (Figure 5D) datasets. In addition, a detailed analysis of the correlation between CIA‐II and several well‐known ICPGs (including *CTLA4*, *PD1*, *CD28*, and *CD80*) was executed in the TCGA (Figure 5E) and CGGA (Figure 5F) cohorts. Nevertheless, the relationship between CIA‐II and ICPGs was inadequate and the potential role of CIA‐II in immunosuppression requires further investigation.

### 3.8 The conjunction between CIA‐II and immune traits

**FIGURE 6:**
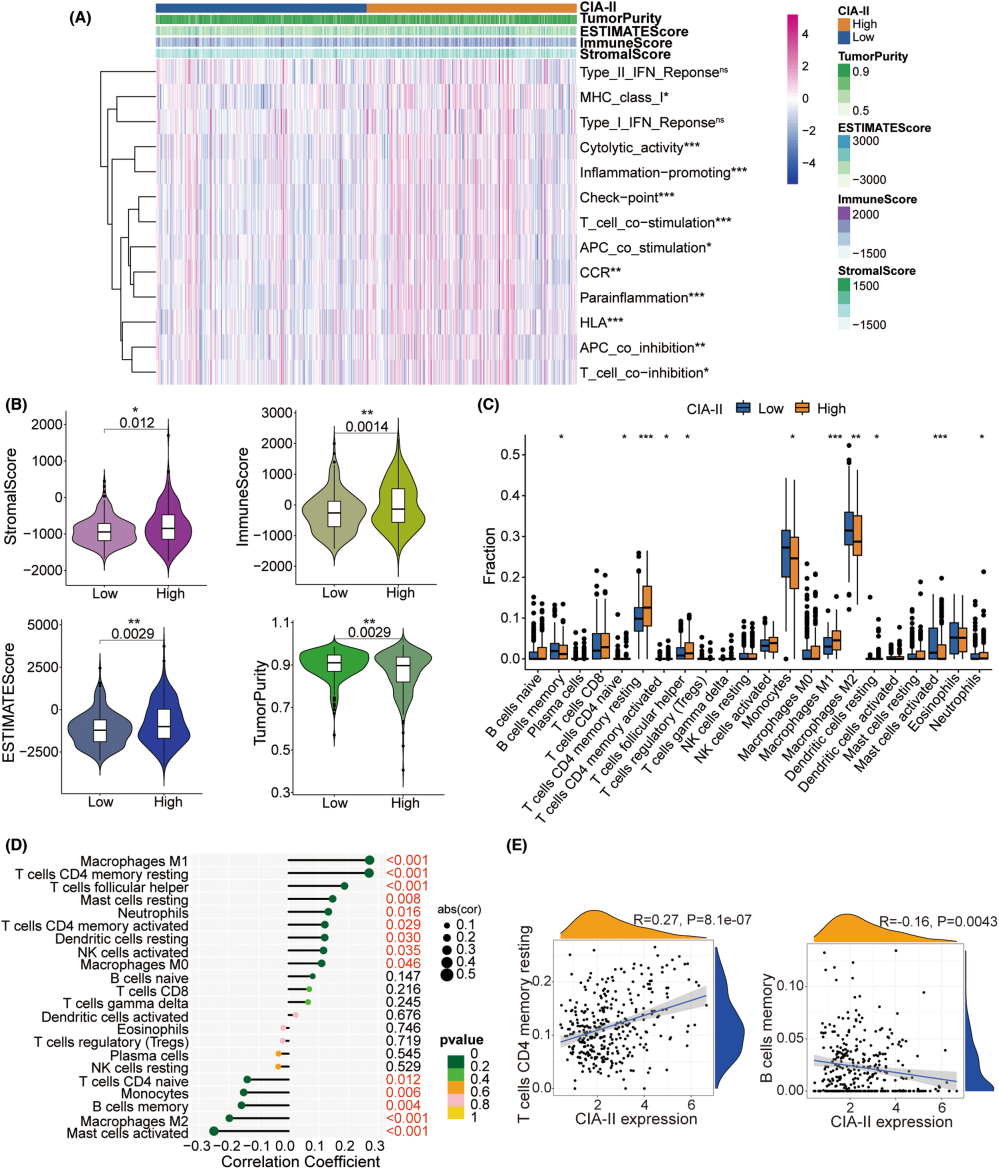
Distinct TIME and immunological features of the low‐CIA‐II and high‐CIA‐II subtypes in TCGA. (A) Differences in immune‐associated functions between the low‐CIA‐II and high‐CIA‐II subtypes. (B) Comparisons of the ESTIMATE, stromal, immune scores, and tumor purity between the low‐CIA‐II and high‐CIA‐II subgroups. (C) Comparisons of the abundances of 22 types of immune cells in the low‐CIA‐II and high‐CIA‐II subtypes. (D) Lollipop plots displaying the association between CIA‐II expression and TIICs. (E) Detailed analysis of the connection between CIA‐II expression and TIICs (*p < 0.05, **p < 0.01, ***p < 0.001).

Given that the results of the GO‐BP enrichment analysis were linked to T‐cell proliferation, leukocyte proliferation, and leukocyte cell–cell adhesion, we further detected the interrelation between CIA‐II expression and immune features. The ssGSEA algorithm was employed to inspect the enrichment of 13 immune‐connected indicators. As shown in the heatmap in Figure 6, most immune‐related features, including checkpoint, T‐cell costimulation, and inflammation‐promoting, were positively associated with CIA‐II expression in the TCGA (Figure 6A) and CGGA datasets (Figure S4A). We executed the ESTIMATE algorithm to measure the stromal, immune scores, tumor purity, and ESTIMATE in the two subgroups. The results revealed that, in TCGA (Figure 6B) and CGGA (Figure S4B) cohorts, the ESTIMATE, stromal, and immune scores were higher in high‐CIA‐II subtype than in low‐CIA‐II subtype, whereas the opposite was observed for the tumor purity score. Moreover, we employed the CIBERSORT algorithm to investigate the differences in TIIC abundance between the two subtypes in patients with LGG. As shown in the box plots in Figure 6C and the lollipop plots in Figure 6D, in TCGA dataset, the levels of infiltration of M1 macrophages, resting memory CD4^+^ T cells, and activated memory CD4^+^ T cells were positively related to CIA‐II expression whereas memory B cell, M2 macrophage, and activated mast cell infiltration levels were inversely correlated with CIA‐II expression. Analogical results were acquired in the CGGA dataset (Figure S4C,D). To better understand immune cell infiltration, we implemented a correlation analysis between CIA‐II expression and infiltration of TIICs in TCGA (Figure 6E) and CGGA (Figure S4E) datasets. These results clearly ascertained that CIA‐II expression was tightly linked to immune cell infiltration in patients with LGG.

### 3.9 Association between CIA‐II expression and genomic variations in LGG

**FIGURE 7:**
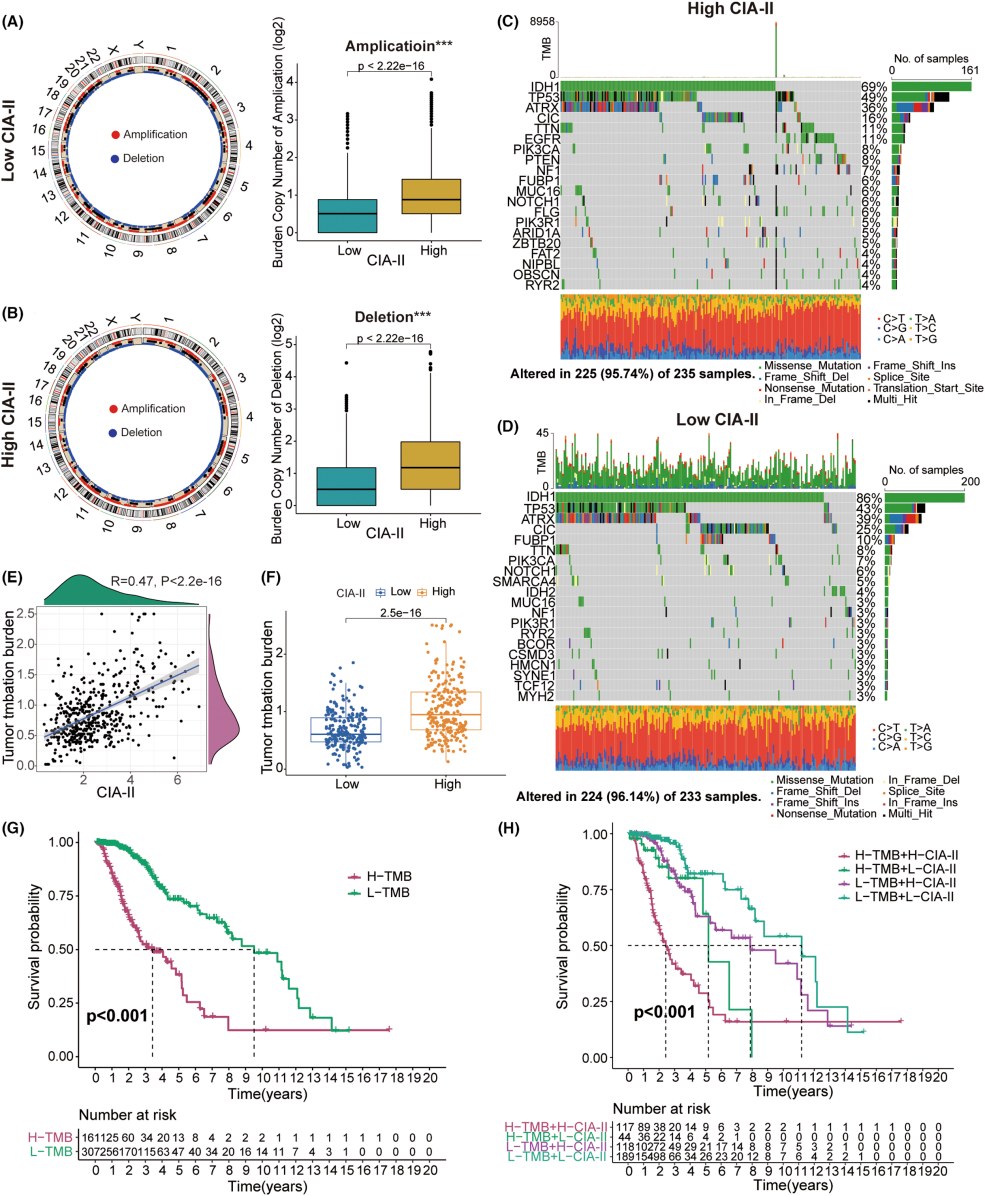
Comparison of the genomic variation between the two subtypes in TCGA dataset. (A, B) Circos plots showing the chromosomal amplifications and deletions in low‐CIA‐II and high‐CIA‐II subtypes. The boxplots show that the copy number amplification and deletion burden was lower in the low‐CIA‐II expression subtype. (C, D) Waterfall plots showing the top 20 mutated genes in the high‐CIA‐II (C) and low‐CIA‐II (D) subtypes. (E, F) TMB levels were higher in high‐CIA‐II expression subtype. (G, H) Correlation between TMB level and the prognosis of patients with LGG (G) and the differential prognostic value in the two subgroups with distinct TMB levels (H) (*p < 0.05, **p < 0.01, ***p < 0.001).

Numerous studies have illustrated that genomic alterations might play a critical part in the modulation of tumor immune infiltration and the prediction of tumor prognosis.^24^, ^25^, ^26^, ^27^ Accordingly, we conducted a CNA and somatic mutation analysis to examine the gene variation between the two subgroups. CNA analysis demonstrated that the burden of copy number amplification and deletion in high‐CIA‐II subset was higher than that in low‐CIA‐II subset (Figure 7A,B). Somatic mutation analysis further indicated that *IDH1* was the most frequently mutated gene in both CIA‐II expression subtypes and that the *IDH1* mutation frequency was higher in low‐CIA‐II subgroup than in high‐CIA‐II subgroup (Figure 7C,D). Additionally, we found that the mutation frequency of the *ATRX* and *CIC* genes was higher in low‐CIA‐II subgroup than in high‐CIA‐II subgroup. However, the variation frequencies of *TP53* and *TTN* in high‐CIA‐II subset were higher than those in low‐CIA‐II subset (Figure 7C,D). Next, we discovered that TMB levels were positively connected with the CIA‐II expression (Figure 7E,F). Moreover, we further inspected the differential OS of distinct CIA‐II expression in the low‐ and high‐TMB subgroups and detected that higher CIA‐II expression and TMB level owned to worser OS in patients with LGG (Figure 7G,H).

### 3.10 In vitro experiments of CIA‐II in LGG

**FIGURE 8:**
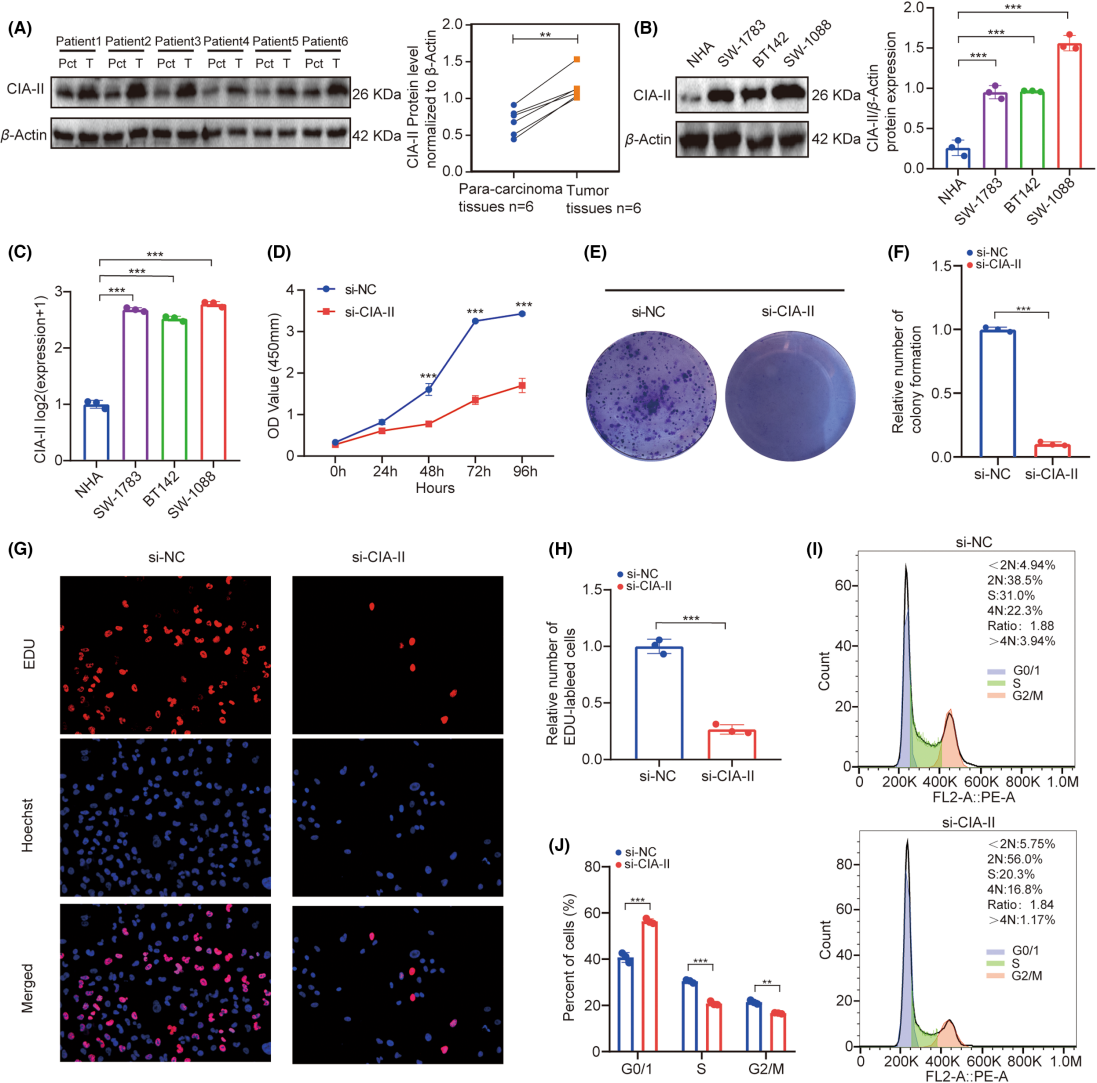
In vitro experiment confirmation of CIA‐II expression in LGG. (A) Western blot analysis of CIA‐II expression in LGG tissues and relevant para‐carcinoma tissues. (B) Western blot and (C) qRT‐PCR analysis of CIA‐II expression in LGG and NHA cell lines. (D) The cell viability of si‐CIA‐II‐transfected and si‐NC‐transfected SW1088 cells by CCK‐8 assays. (E, F) Effect of CIA‐II knockdown on colony formation in SW1088 cells was evaluated. (G, H) Representative images (G) and statistical analysis (H) of EdU assays after CIA‐II knockdown in SW1088 cells. (I, J) Cell cycle assays were employed to inspect the cell distribution of the SW1088 cell lines transfected with si‐CIA‐II or si‐NC lentiviruses. Significance level was evaluated using two‐tailed *t*‐tests and two‐way analysis of variance (ANOVA) followed by Tukey's tests for multiple comparison. Figures with exact data points displayed standard deviations of three independent experiments (*p < 0.05, **p < 0.01, ***p < 0.001).

We next checked the protein expression levels of CIA‐II in six postoperative LGG and paired para‐cancerous samples. We detected that the protein expression of CIA‐II in LGG tissues was higher than that in para‐cancerous tissue (Figure 8A). Additionally, we checked the protein and mRNA expression of CIA‐II in a NHA cell line and three LGG cell lines (SW1088, SW1783, and BT142). We observed that CIA‐II expression was higher in the LGG cell lines than in the NHA cell line (Figure 8B,C).

CCK‐8 assays suggested that the viability of SW1088 decreased significantly after silencing CIA‐II (Figure 8D). Next, colony formation assays displayed that CIA‐II knockdown significantly reduced cell colonies when compared to NC (Figure 8E,F). Furthermore, EdU assays revealed that CIA‐II knockdown obviously inhibited cells proliferation (Figure 8G,H). The cell cycle analysis illustrated that CIA‐II downregulation induced cell cycle arrest (Figure 8I,J). These results suggest that CIA‐II is essential for cell proliferation in LGG.

### 3.11 The association between CIA‐II expression and chemotherapeutics

To estimate the ability of different levels of CIA‐II expression to guide clinical decision making in LGG, we explored the correlations between the low‐CIA‐II and high‐CIA‐II subtypes of LGG and some common anticancer drugs, such as bryostain 1, bortezomib, AP‐24534, AS605240, A‐443654, AKT inhibitor VIII, CAL‐101, and ZSTK474. The results demonstrated that the high‐CIA‐II subtype was linked to a lower inhibitory concentration (IC50) of these anticancer drugs, indicating that tumors with elevated CIA‐II expression were more sensitive to these chemotherapeutics (Figure S5).

## 4 DISCUSSION

The effects of conventional treatments for gliomas, including surgery, radiation therapy, and chemotherapy, remain limited.^28^, ^29^ Hence, novel biomarkers are vitally important to improve the therapeutic effects for patients with glioma. CIA‐II has been widely reported to play a pivotal part in numerous cancers; however, whether it is also involved in the pathophysiology of LGG is undiscovered. Herein, we comprehensively inspect the interrelation between CIA‐II expression and prognosis, clinical traits, specific functions, tumor immunity, gene mutations, and sensitivity to chemotherapeutics in LGG patients from TCGA and CGGA cohorts.

We undertook a pan‐cancer analysis of CIA‐II in 33 cancer types and detected that higher CIA‐II expression was tightly linked to poorer prognosis, higher ICPGs expression, and higher TMB in pan‐LGG. To detect the prognostic value of CIA‐II in LGG, we implemented a survival analysis in TCGA and CGGA cohorts and observed that higher CIA‐II expression was linked to poorer survival. The results of Cox regression analyses suggested that CIA‐II was a robust independent prognostic indicator of LGG patients. Moreover, we generated a clinical nomogram model to forecast 1/3/5‐year OS in LGG patients in line with the results of the Cox regression analyses and verified the accurateness of this model through calibration curves.

Subsequently, we performed a GSVA to identify the molecular pathways underlying the effects of CIA‐II in LGG. Meanwhile, DEGs between the two CIA‐II expression subtypes were filtered out and applied for GO‐BP and KEGG analyses to investigate the underlying biological functions. The results disclosed that the DEGs identified in the high‐CIA‐II LGG subtype were strongly linked to processes such as T‐cell proliferation and leukocyte cell–cell adhesion and pathways such as the PI3K‐AKT and p53 signaling pathways, and the cell cycle. To examine the conjunction between CIA‐II and related genes, we performed a correlation analysis and found that CIA‐II was most negatively correlated with *CBX7*, *ALDH2*, *NRG3*, *MRO*, and *LDHD* and positively correlated with *MELK*, *NDC80*, *RRM2*, *NCAPG*, *HJURP*, and *BUB1*. Over recent years, checkpoint inhibitors, such as those targeting CTLA‐4 and PD1 (PDCD1), have been employed as immunotherapy in several cancers.^30^, ^31^, ^32^ Accordingly, we ascertained the conjunction between CIA‐II expression and the expression of ICPGs in LGG patients. The results verified that CIA‐II expression was positively interrelated with most of the ICPGs evaluated (such as *CTLA4*, *PD1*, *CD28*, and *CD80*), suggesting that checkpoint inhibitors other than CTLA‐4 and PD1 may represent novel immunotherapeutic targets for LGG patients.

Increased research has elaborated that LGG immune microenvironment may be closely linked to the survival of LGG patients.^33^, ^34^ Thus, we examined the interrelation between the CIA‐II expression and immune signatures in LGG. The ssGSEA algorithm was exploited to detect the difference in immune‐connected features between the two subtypes. Additionally, the ESTIMATE and CIBERSORT algorithms were exploited to check the composition of the TME and TIIC infiltration levels between the two CIA‐II expression subsets. The results demonstrated that CIA‐II expression was prominently related to immune infiltration. Considering the potential influence of genomic variation in tumor immunity, we further undertook genomic mutation analyses and found that the TMB and the CNA burden were lower in low‐CIA‐II subgroup than in high‐CIA‐II subgroup.

Although TMZ is the most commonly used chemotherapeutic drug for the therapy of patients with glioma, its efficacy is limited.^35^ Here, we compared the efficacy of the PI3K/AKT inhibitors A‐443654, AKT inhibitor VIII, AS605240, ZSTK474, and CAL‐101; the MAPK inhibitor AP‐24534; the proteasome inhibitors bryostain 1 and bortezomib between the low‐CIA‐II and high‐CIA‐II LGG subtypes and observed that the high‐CIA‐II subgroup exhibited greater sensitivity to these chemotherapeutic drugs. These findings imply that CIA‐II may be an underlying predictor for the chemosensitivity of LGG patients.

Finally, we confirmed that CIA‐II was increased and crucial for the cell proliferation in LGG through in vitro studies. We recognized that the proliferation capacity of LGG cells was distinctly decreased after silencing the expression of CIA‐II. However, there are still some limitations in this work. This is a preliminary study, and we will continue to detect the special roles of CIA‐II high‐grade glioma in the next article. Further study should be considered to inspect whether CIA‐II is a reliable therapeutic target for LGG patients. Additionally, the underlying functions of CIA‐II in LGG should be excavated by implementing the in vitro and in vivo studies in the near future.

## 5 CONCLUSION

In conclusion, our data indicated that CIA‐II has potential as a prognostic biomarker for LGG and is tightly correlated with the immunological characteristics of this malignancy. Accordingly, CIA‐II may be an underlying therapeutic target for LGG patients.

## AUTHOR CONTRIBUTIONS

FX designed and drafted the manuscript; FX, HZ, YG, ZZ, GS, YX, and GH wrote figure legends and revised the article; FX, HZ, and YG, conducted the data analysis. All authors read and approved the final manuscript.

## FUNDING INFORMATION

This study was supported by The National Natural Science Foundation of China (No. 81960457), Graduate Innovative Special Fund Projects of Jiangxi Province (No. YC2022—B073), Health Commission of Jiangxi Province (No. 202130356), and Education Department of Jiangxi Province (No. GJJ200178).

## CONFLICT OF INTEREST STATEMENT

All authors declare that they have no competing interests.

## Supporting information


Figure S1.

Figure S2.

Figure S3.

Figure S4.

Figure S5.

Table S1.

Table S2.

Table S3.

Table S4.

Data S1.

Data S2.

## Data Availability

The data analyzed in this research can be found in the TCGA (https://portal.gdc.cancer.gov/) and CGGA (http://www.cgga.org.cn/) websites.
