# Supplementary figures and images for: CIA‐II is associated with lower‐grade glioma survival and cell proliferation
Source: CNS Neurosci Ther. 2023 Jul 14;30(2):e14340. doi: 10.1111/cns.14340 (PMC10848044; doi:10.1111/cns.14340)

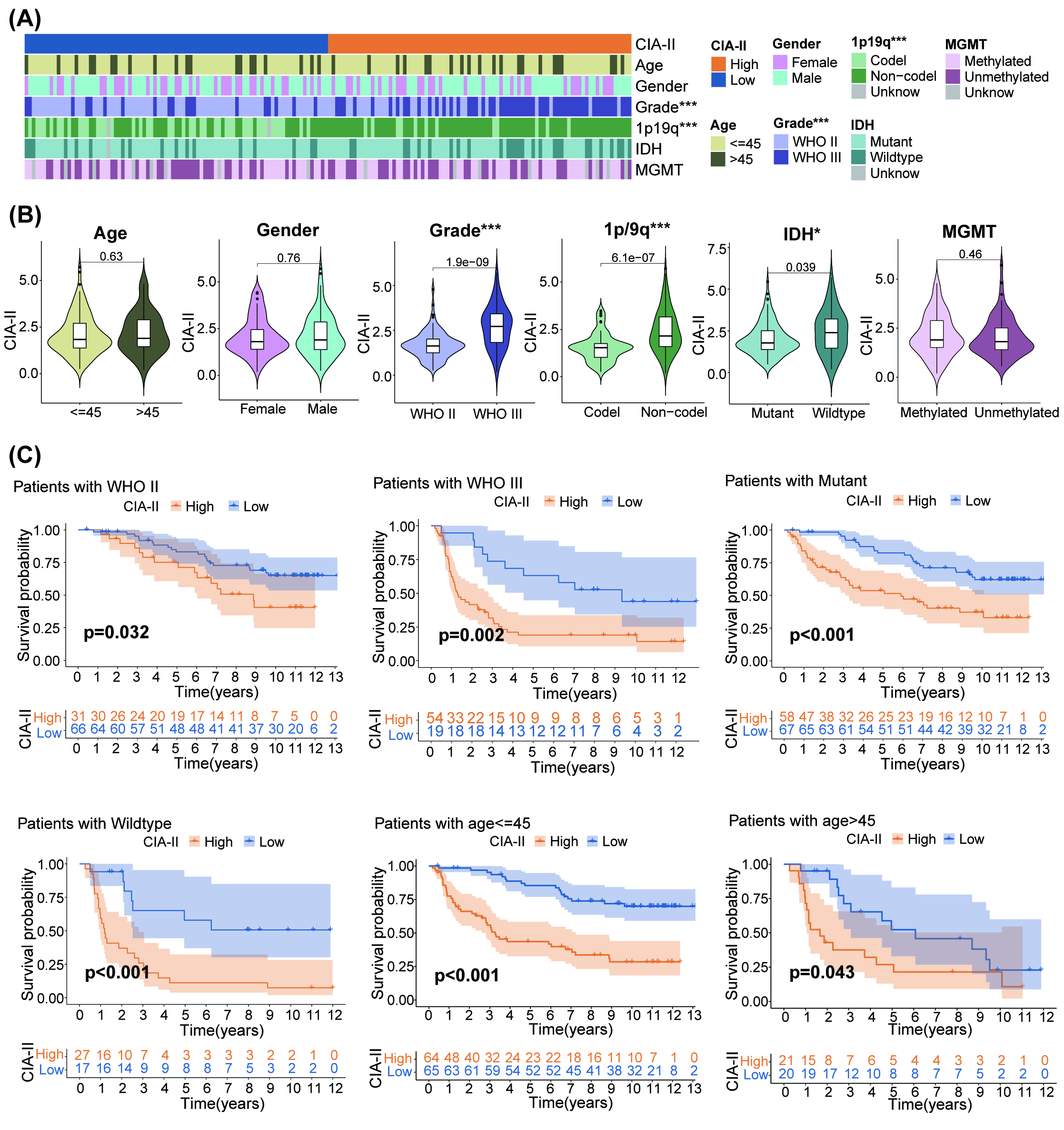

Supplement: Supplementary file 1 — Figure S1. [file CNS-30-e14340-s002.tif]

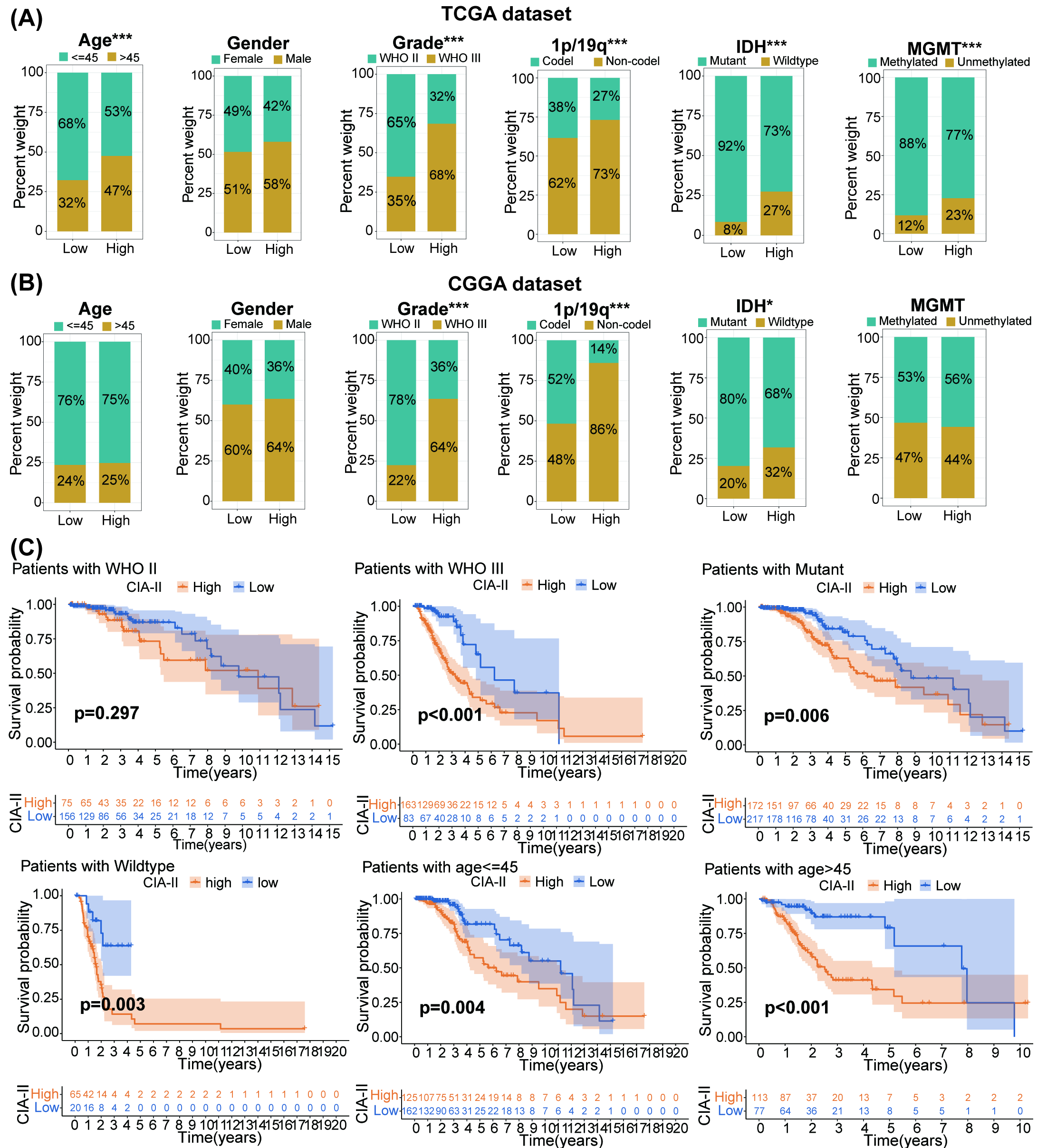

Supplement: Supplementary file 2 — Figure S2. [file CNS-30-e14340-s004.tif]

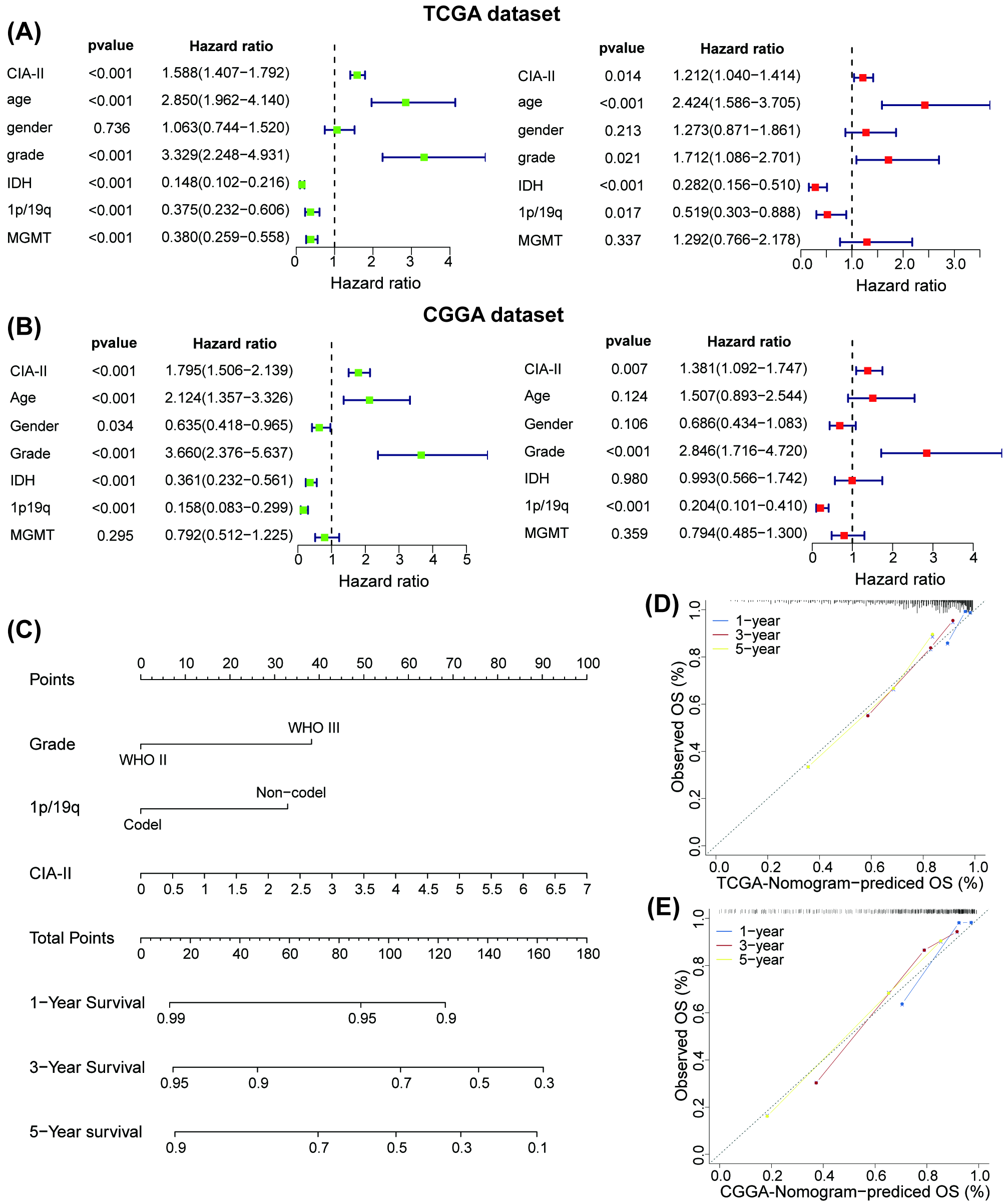

Supplement: Supplementary file 3 — Figure S3. [file CNS-30-e14340-s011.tif]

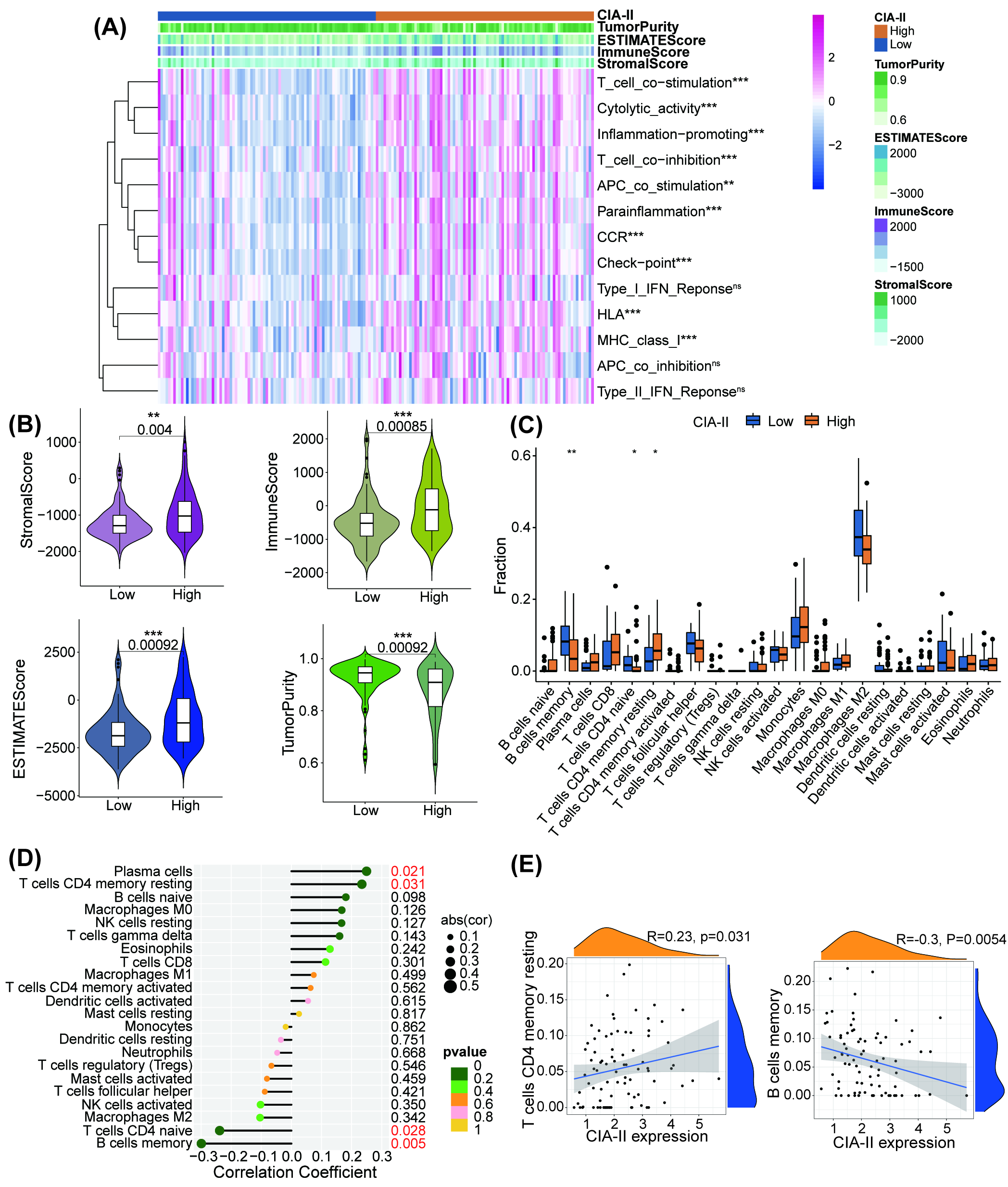

Supplement: Supplementary file 4 — Figure S4. [file CNS-30-e14340-s001.tif]

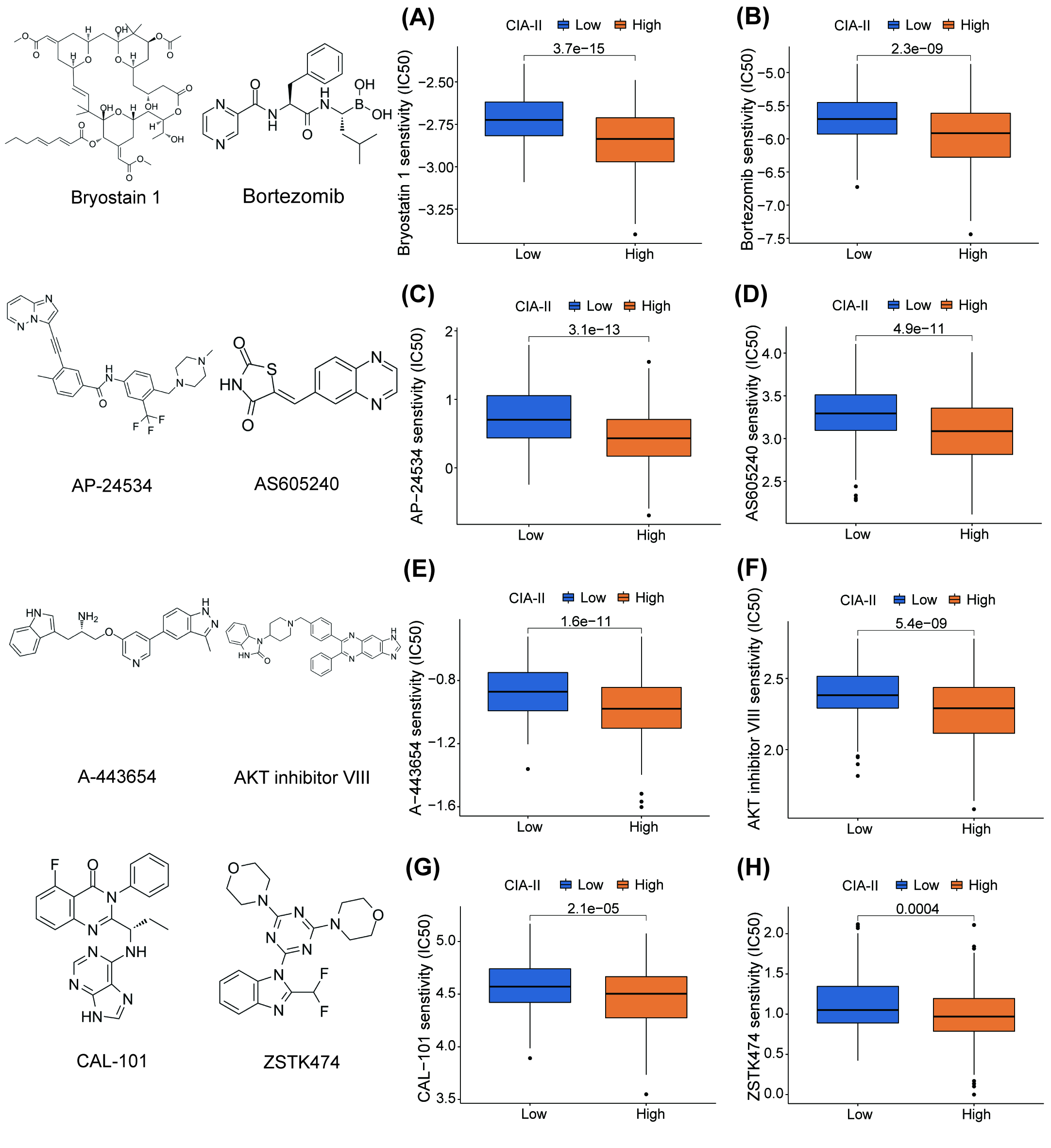

Supplement: Supplementary file 5 — Figure S5. [file CNS-30-e14340-s009.tif]

Full unedited blot for Figure 8A

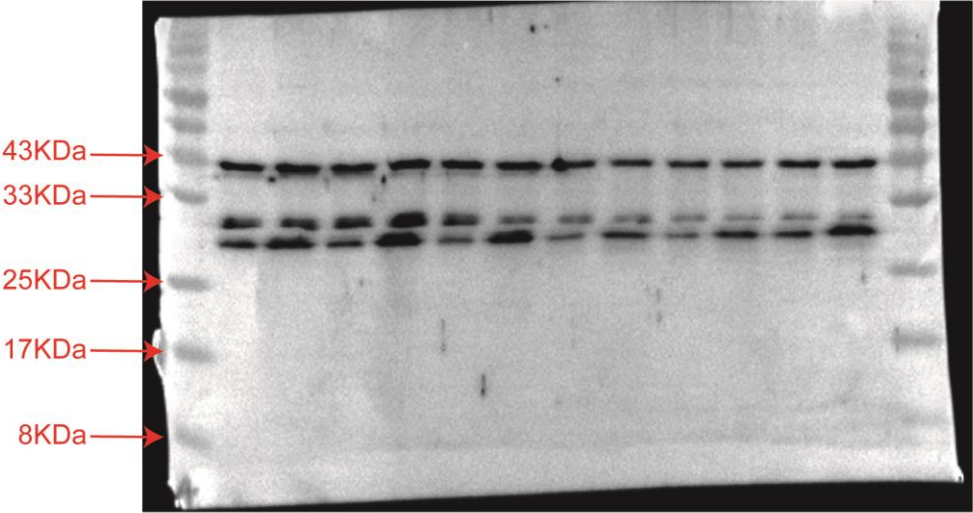

Full unedited blot for Figure 8B

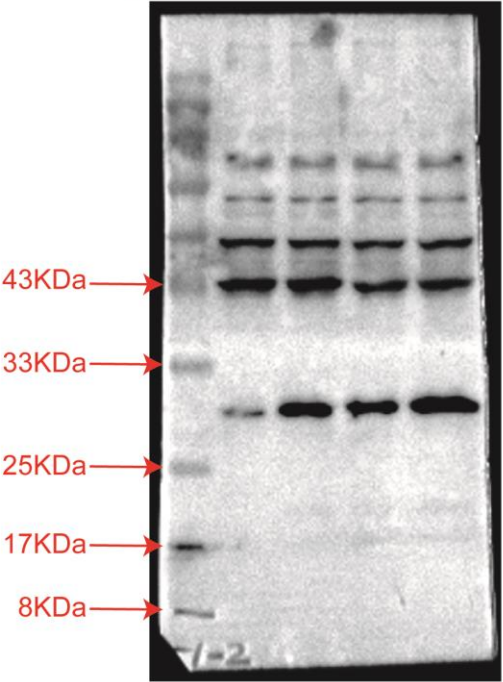

Supplement: Supplementary file 10 — Data S1. [file CNS-30-e14340-s008.pdf]
