# Supplementary material for: CIA‐II is associated with lower‐grade glioma survival and cell proliferation
Source: CNS Neurosci Ther. 2023 Jul 14;30(2):e14340. doi: 10.1111/cns.14340 (PMC10848044; doi:10.1111/cns.14340)
Supplement: Supplementary file 6 — Table S1. [file CNS-30-e14340-s005.docx]

**Table S1.** Clinical features of LGG patients from TCGA

| Clinical features |  | Total (477) | % |
| --- | --- | --- | --- |
| Age | Age <=45 | 287 | 60.17% |
|  | Age >45 | 190 | 39.83 % |
| Gender | Female | 216 | 45.28% |
|  | Male | 261 | 54.72% |
| Grade | WHO II | 231 | 48.43% |
|  | WHO III | 246 | 51.57% |
| 1p/19q | Non-codel | 321 | 67.30% |
|  | Codel | 156 | 32.70% |
| IDH | Mutant | 389 | 81.55% |
|  | Wildtype | 85 | 17.82% |
|  | Unknow | 3 | 0.63% |
| MGMT | Unmethylated | 82 | 17.19% |
|  | Methylated | 395 | 82.81% |
