# Supplementary material for: CIA‐II is associated with lower‐grade glioma survival and cell proliferation
Source: CNS Neurosci Ther. 2023 Jul 14;30(2):e14340. doi: 10.1111/cns.14340 (PMC10848044; doi:10.1111/cns.14340)
Supplement: Supplementary file 7 — Table S2. [file CNS-30-e14340-s003.docx]

**Table S2.** Clinical features of LGG patients from CGGA.

| Clinical features |  | Total (170) | % |
| --- | --- | --- | --- |
| Age | Age <=45 | 129 | 75.88% |
|  | Age >45 | 41 | 24.12% |
| Gender | Female | 65 | 38.24% |
|  | Male | 105 | 61.76% |
| Grade | WHO II | 97 | 57.06% |
|  | WHO III | 73 | 42.94% |
| 1p/19q | Non-codel | 113 | 66.47% |
|  | Codel | 55 | 32.35% |
|  | Unknow | 2 | 1.18% |
| IDH | Mutant | 125 | 73.53% |
|  | Wildtype | 44 | 25.88% |
|  | Unknow | 1 | 0.59% |
| MGMT | Unmethylated | 70 | 41.18% |
|  | Methylated | 84 | 49.41% |
|  | Unknow | 16 | 9.41% |
