# Supplementary material for: CIA‐II is associated with lower‐grade glioma survival and cell proliferation
Source: CNS Neurosci Ther. 2023 Jul 14;30(2):e14340. doi: 10.1111/cns.14340 (PMC10848044; doi:10.1111/cns.14340)
Supplement: Supplementary file 8 — Table S3. [file CNS-30-e14340-s010.docx]

**Table S3.** DEGs in TCGA LGG cohort.

| **id** | **logFC** | **AveExpr** | **t** | **P.Value** | **adj.P.Val** | **B** |
| --- | --- | --- | --- | --- | --- | --- |
| ASF1B | 2.0524 | 2.498543 | 26.5508 | 5.82E-96 | 3.19E-91 | 207.738 |
| RRM2 | 2.32649 | 2.367335 | 25.7902 | 2.06E-92 | 5.65E-88 | 199.655 |
| TPX2 | 2.21723 | 3.632731 | 25.4668 | 6.74E-91 | 1.23E-86 | 196.205 |
| BIRC5 | 2.40342 | 2.716624 | 25.1766 | 1.55E-89 | 2.12E-85 | 193.104 |
| PBK | 2.40722 | 2.85034 | 24.664 | 3.99E-87 | 4.37E-83 | 187.611 |
| NUSAP1 | 2.31412 | 3.640789 | 24.5471 | 1.42E-86 | 1.29E-82 | 186.357 |
| KIFC1 | 2.14694 | 2.842828 | 24.4349 | 4.79E-86 | 3.75E-82 | 185.151 |
| UBE2C | 2.61754 | 3.433359 | 24.3938 | 7.49E-86 | 4.30E-82 | 184.709 |
| CDK1 | 1.98671 | 2.588331 | 24.3927 | 7.57E-86 | 4.30E-82 | 184.698 |
| PIMREG | 2.29805 | 2.583662 | 24.3894 | 7.85E-86 | 4.30E-82 | 184.663 |
| TOP2A | 2.71833 | 3.433001 | 24.2147 | 5.23E-85 | 2.61E-81 | 182.785 |
| FOXM1 | 2.19386 | 3.068525 | 24.0246 | 4.13E-84 | 1.89E-80 | 180.739 |
| AURKB | 2.10565 | 2.134077 | 23.7623 | 7.17E-83 | 3.02E-79 | 177.914 |
| MKI67 | 2.09787 | 2.284385 | 23.6854 | 1.66E-82 | 6.49E-79 | 177.085 |
| MYBL2 | 2.47426 | 2.573085 | 23.6539 | 2.33E-82 | 8.53E-79 | 176.746 |
| MELK | 1.90438 | 1.839841 | 23.5513 | 7.14E-82 | 2.45E-78 | 175.639 |
| DTL | 1.76742 | 1.994989 | 23.5154 | 1.06E-81 | 3.41E-78 | 175.252 |
| NCAPH | 1.72464 | 2.207485 | 23.5011 | 1.23E-81 | 3.76E-78 | 175.097 |
| TK1 | 1.83292 | 2.949029 | 23.0014 | 2.87E-79 | 8.27E-76 | 169.703 |
| PCLAF | 1.67478 | 1.758283 | 22.7768 | 3.33E-78 | 9.12E-75 | 167.275 |
| HJURP | 1.80774 | 1.719816 | 22.762 | 3.91E-78 | 1.02E-74 | 167.115 |
| SPC24 | 1.83711 | 2.235054 | 22.6963 | 8.02E-78 | 2.00E-74 | 166.404 |
| CDCA5 | 1.68107 | 3.003299 | 22.6587 | 1.21E-77 | 2.88E-74 | 165.998 |
| CDC45 | 1.71199 | 1.87498 | 22.4936 | 7.34E-77 | 1.68E-73 | 164.212 |
| FAM111B | 1.58967 | 1.510245 | 22.4476 | 1.21E-76 | 2.66E-73 | 163.715 |
| NCAPG | 1.75381 | 1.662871 | 22.4083 | 1.86E-76 | 3.93E-73 | 163.29 |
| KIF20A | 1.81686 | 1.676325 | 22.3598 | 3.17E-76 | 6.43E-73 | 162.764 |
| BUB1 | 1.63886 | 1.783074 | 22.3528 | 3.42E-76 | 6.69E-73 | 162.689 |
| SKA3 | 1.58741 | 1.790446 | 22.2965 | 6.32E-76 | 1.19E-72 | 162.08 |
| CEP55 | 1.53524 | 1.43643 | 22.0831 | 6.51E-75 | 1.19E-71 | 159.77 |
| CENPF | 2.00778 | 2.407997 | 22.0561 | 8.74E-75 | 1.54E-71 | 159.479 |
| CKAP2L | 1.61694 | 1.463633 | 21.98 | 2.01E-74 | 3.44E-71 | 158.654 |
| GTSE1 | 1.70446 | 1.879185 | 21.9765 | 2.09E-74 | 3.46E-71 | 158.617 |
| CCNB2 | 1.92563 | 2.277399 | 21.9551 | 2.64E-74 | 4.25E-71 | 158.385 |
| CENPU | 1.81002 | 2.408627 | 21.8307 | 1.03E-73 | 1.61E-70 | 157.039 |
| NDC80 | 1.75897 | 1.848322 | 21.7904 | 1.59E-73 | 2.43E-70 | 156.602 |
| ZWINT | 1.52332 | 3.591578 | 21.6338 | 8.83E-73 | 1.31E-69 | 154.907 |
| KIF11 | 1.48659 | 2.799759 | 21.5696 | 1.78E-72 | 2.57E-69 | 154.212 |
| TROAP | 1.72074 | 1.622025 | 21.5332 | 2.65E-72 | 3.73E-69 | 153.818 |
| PTTG1 | 1.80369 | 3.2565 | 21.5286 | 2.79E-72 | 3.82E-69 | 153.768 |
| IQGAP3 | 1.58061 | 1.61023 | 21.5145 | 3.25E-72 | 4.35E-69 | 153.615 |
| ESPL1 | 1.38003 | 1.199167 | 21.4696 | 5.31E-72 | 6.93E-69 | 153.13 |
| RAD51 | 1.27 | 1.586038 | 21.2405 | 6.50E-71 | 8.28E-68 | 150.649 |
| BUB1B | 1.78444 | 1.884441 | 21.0947 | 3.20E-70 | 3.98E-67 | 149.071 |
| ZNF367 | 1.26853 | 2.660244 | 21.0142 | 7.70E-70 | 9.38E-67 | 148.2 |
| RAD51AP1 | 1.43357 | 2.390513 | 20.8815 | 3.28E-69 | 3.91E-66 | 146.764 |
| CCNA2 | 1.60545 | 2.538669 | 20.8761 | 3.48E-69 | 4.06E-66 | 146.705 |
| NUF2 | 1.53635 | 2.072836 | 20.8686 | 3.78E-69 | 4.31E-66 | 146.624 |
| GINS1 | 1.25618 | 2.847124 | 20.7905 | 8.86E-69 | 9.91E-66 | 145.78 |
| SKA1 | 1.37748 | 1.273558 | 20.6995 | 2.39E-68 | 2.62E-65 | 144.795 |
| TTK | 1.48267 | 1.383982 | 20.6736 | 3.18E-68 | 3.41E-65 | 144.515 |
| POC1A | 1.22319 | 2.674527 | 20.659 | 3.72E-68 | 3.92E-65 | 144.357 |
| DLGAP5 | 1.65059 | 1.366562 | 20.6096 | 6.38E-68 | 6.60E-65 | 143.823 |
| MCM10 | 1.43179 | 1.323006 | 20.5585 | 1.12E-67 | 1.13E-64 | 143.27 |
| PKMYT1 | 1.40217 | 2.24747 | 20.4663 | 3.05E-67 | 3.04E-64 | 142.273 |
| TIMELESS | 1.26532 | 3.590061 | 20.4315 | 4.46E-67 | 4.37E-64 | 141.897 |
| KIF4A | 1.70873 | 2.167521 | 20.4241 | 4.84E-67 | 4.65E-64 | 141.817 |
| SHCBP1 | 1.31355 | 1.78694 | 20.3966 | 6.52E-67 | 6.16E-64 | 141.521 |
| EXO1 | 1.39554 | 1.301788 | 20.348 | 1.11E-66 | 1.03E-63 | 140.995 |
| SGO1 | 1.21781 | 1.061803 | 20.3222 | 1.47E-66 | 1.34E-63 | 140.716 |
| FANCD2 | 1.113 | 1.863117 | 20.3085 | 1.71E-66 | 1.53E-63 | 140.568 |
| CENPM | 1.38369 | 2.51935 | 20.2309 | 3.98E-66 | 3.52E-63 | 139.729 |
| CDCA8 | 1.69501 | 2.287186 | 20.2027 | 5.40E-66 | 4.70E-63 | 139.426 |
| ARHGAP11A | 1.34875 | 2.04483 | 20.1238 | 1.28E-65 | 1.09E-62 | 138.573 |
| KNL1 | 1.29758 | 1.189545 | 20.0763 | 2.14E-65 | 1.81E-62 | 138.06 |
| TCF19 | 1.22285 | 3.274626 | 20.0541 | 2.73E-65 | 2.27E-62 | 137.821 |
| TACC3 | 1.51876 | 2.96001 | 20.0205 | 3.94E-65 | 3.22E-62 | 137.457 |
| KIF2C | 1.76399 | 2.335134 | 19.971 | 6.75E-65 | 5.44E-62 | 136.923 |
| CDC25C | 1.22324 | 1.135558 | 19.9544 | 8.09E-65 | 6.42E-62 | 136.745 |
| CENPK | 1.26893 | 1.314827 | 19.7986 | 4.41E-64 | 3.45E-61 | 135.064 |
| CENPA | 1.48473 | 1.378783 | 19.7705 | 5.99E-64 | 4.62E-61 | 134.76 |
| CDT1 | 1.39637 | 2.906918 | 19.5774 | 4.89E-63 | 3.72E-60 | 132.68 |
| FANCI | 1.36792 | 2.663886 | 19.5611 | 5.84E-63 | 4.38E-60 | 132.504 |
| HMMR | 1.3155 | 1.567149 | 19.5281 | 8.36E-63 | 6.19E-60 | 132.148 |
| KIF23 | 1.40125 | 1.357645 | 19.4239 | 2.59E-62 | 1.90E-59 | 131.026 |
| NEK2 | 1.52522 | 1.569797 | 19.4149 | 2.86E-62 | 2.06E-59 | 130.93 |
| PRC1 | 1.41688 | 3.356271 | 19.4112 | 2.98E-62 | 2.12E-59 | 130.89 |
| CDC20 | 1.95625 | 2.845754 | 19.3459 | 6.05E-62 | 4.25E-59 | 130.187 |
| KIF15 | 1.30909 | 2.34256 | 19.2432 | 1.84E-61 | 1.28E-58 | 129.082 |
| GINS2 | 1.26001 | 2.548089 | 19.086 | 1.01E-60 | 6.95E-58 | 127.394 |
| CENPH | 1.08967 | 3.175401 | 19.0703 | 1.20E-60 | 8.13E-58 | 127.225 |
| ESCO2 | 1.07099 | 0.886959 | 19.0203 | 2.07E-60 | 1.38E-57 | 126.688 |
| ORC1 | 1.21613 | 1.391068 | 19 | 2.58E-60 | 1.70E-57 | 126.47 |
| AURKA | 1.23406 | 2.340212 | 18.9192 | 6.18E-60 | 4.03E-57 | 125.602 |
| EME1 | 1.10532 | 1.870804 | 18.9006 | 7.56E-60 | 4.87E-57 | 125.403 |
| E2F2 | 1.39244 | 1.372179 | 18.8298 | 1.63E-59 | 1.04E-56 | 124.644 |
| CDCA2 | 1.21129 | 1.069682 | 18.8005 | 2.23E-59 | 1.41E-56 | 124.33 |
| KIF14 | 1.21872 | 1.092046 | 18.7902 | 2.50E-59 | 1.55E-56 | 124.219 |
| CDC6 | 1.20104 | 1.655195 | 18.778 | 2.85E-59 | 1.75E-56 | 124.088 |
| TRIP13 | 1.12793 | 2.075088 | 18.7167 | 5.53E-59 | 3.37E-56 | 123.431 |
| DDIAS | 1.0252 | 1.295355 | 18.7097 | 5.96E-59 | 3.59E-56 | 123.356 |
| E2F1 | 1.3751 | 3.850835 | 18.7018 | 6.50E-59 | 3.87E-56 | 123.271 |
| NEIL3 | 1.13078 | 0.917318 | 18.6529 | 1.10E-58 | 6.49E-56 | 122.748 |
| HASPIN | 0.97265 | 0.862939 | 18.639 | 1.28E-58 | 7.46E-56 | 122.599 |
| CCNB1 | 1.38608 | 3.591426 | 18.5931 | 2.10E-58 | 1.21E-55 | 122.108 |
| RAD54L | 1.24013 | 1.606793 | 18.59 | 2.17E-58 | 1.24E-55 | 122.075 |
| OIP5 | 1.18445 | 1.851748 | 18.4224 | 1.33E-57 | 7.49E-55 | 120.282 |
| LMNB1 | 1.46024 | 4.247986 | 18.2218 | 1.15E-56 | 6.44E-54 | 118.14 |
| BRIP1 | 1.0303 | 0.913412 | 18.1869 | 1.68E-56 | 9.28E-54 | 117.768 |
| MCM2 | 1.23246 | 4.057274 | 18.0253 | 9.53E-56 | 5.22E-53 | 116.046 |
| CHAF1A | 1.01773 | 3.59669 | 17.9261 | 2.76E-55 | 1.50E-52 | 114.991 |
| ASPM | 1.39088 | 1.135268 | 17.924 | 2.83E-55 | 1.52E-52 | 114.968 |
| WDR62 | 1.15361 | 1.346531 | 17.8605 | 5.59E-55 | 2.97E-52 | 114.293 |
| CDKN3 | 1.25568 | 2.218512 | 17.8573 | 5.79E-55 | 3.05E-52 | 114.258 |
| CHEK1 | 0.96876 | 2.364265 | 17.8497 | 6.27E-55 | 3.27E-52 | 114.178 |
| TYMS | 1.63757 | 3.872871 | 17.7454 | 1.92E-54 | 9.93E-52 | 113.069 |
| FANCA | 0.89487 | 1.487022 | 17.5651 | 1.32E-53 | 6.77E-51 | 111.157 |
| CKS2 | 1.32246 | 5.186447 | 17.4173 | 6.41E-53 | 3.25E-50 | 109.593 |
| BRCA2 | 0.75797 | 0.820383 | 17.328 | 1.66E-52 | 8.35E-50 | 108.65 |
| RNASEH2A | 0.92069 | 4.832033 | 17.1764 | 8.34E-52 | 4.16E-49 | 107.05 |
| HROB | 0.79903 | 1.726168 | 17.1652 | 9.39E-52 | 4.64E-49 | 106.932 |
| PLK4 | 1.02447 | 1.830197 | 17.1409 | 1.22E-51 | 5.96E-49 | 106.675 |
| CLSPN | 1.14164 | 1.15752 | 17.1108 | 1.68E-51 | 8.13E-49 | 106.358 |
| CHEK2 | 0.85457 | 1.830043 | 17.0583 | 2.93E-51 | 1.41E-48 | 105.805 |
| DEPDC1B | 1.12157 | 1.314785 | 17.0342 | 3.78E-51 | 1.80E-48 | 105.552 |
| TRAIP | 0.84205 | 2.248239 | 17.0269 | 4.09E-51 | 1.93E-48 | 105.475 |
| CENPW | 1.1064 | 2.848567 | 16.9295 | 1.15E-50 | 5.38E-48 | 104.451 |
| CENPE | 1.11166 | 1.090901 | 16.9239 | 1.22E-50 | 5.66E-48 | 104.392 |
| MND1 | 1.08783 | 1.590292 | 16.9018 | 1.54E-50 | 7.09E-48 | 104.16 |
| PLK1 | 1.1831 | 2.637768 | 16.8937 | 1.68E-50 | 7.66E-48 | 104.075 |
| RMI2 | 1.17638 | 2.05318 | 16.8594 | 2.42E-50 | 1.09E-47 | 103.714 |
| ERCC6L | 0.74214 | 0.681118 | 16.8033 | 4.37E-50 | 1.96E-47 | 103.126 |
| EZH2 | 1.31239 | 3.368986 | 16.7809 | 5.54E-50 | 2.47E-47 | 102.892 |
| POLQ | 0.74229 | 0.648036 | 16.7382 | 8.71E-50 | 3.85E-47 | 102.444 |
| PCNA | 0.878 | 6.781405 | 16.7337 | 9.12E-50 | 4.00E-47 | 102.397 |
| NRM | 1.03025 | 4.086687 | 16.7309 | 9.40E-50 | 4.09E-47 | 102.368 |
| ECT2 | 1.12833 | 2.808463 | 16.5498 | 6.36E-49 | 2.74E-46 | 100.473 |
| PARPBP | 0.72042 | 1.123983 | 16.5291 | 7.91E-49 | 3.39E-46 | 100.257 |
| CDK2 | 1.15022 | 3.447407 | 16.5198 | 8.72E-49 | 3.71E-46 | 100.16 |
| ORC6 | 0.99738 | 2.041639 | 16.506 | 1.01E-48 | 4.26E-46 | 100.015 |
| HELLS | 0.83416 | 1.360265 | 16.4909 | 1.18E-48 | 4.95E-46 | 99.8578 |
| CDC25A | 1.01122 | 2.178473 | 16.4549 | 1.73E-48 | 7.17E-46 | 99.4824 |
| CDCA3 | 0.99162 | 2.127872 | 16.4322 | 2.19E-48 | 9.04E-46 | 99.2455 |
| MAD2L1 | 0.9715 | 2.724292 | 16.2963 | 9.15E-48 | 3.74E-45 | 97.8303 |
| UBE2T | 1.28856 | 3.948774 | 16.2884 | 9.95E-48 | 4.04E-45 | 97.748 |
| POLA2 | 0.65185 | 3.155265 | 16.1243 | 5.56E-47 | 2.24E-44 | 96.0432 |
| HAUS8 | 0.70347 | 1.996368 | 16.1149 | 6.13E-47 | 2.45E-44 | 95.9453 |
| CENPI | 0.83433 | 1.241948 | 16.0482 | 1.23E-46 | 4.89E-44 | 95.2542 |
| KNTC1 | 0.87812 | 2.097282 | 16.0332 | 1.44E-46 | 5.68E-44 | 95.0991 |
| SPAG5 | 1.11951 | 2.899135 | 16.0324 | 1.45E-46 | 5.69E-44 | 95.0907 |
| DSN1 | 0.84289 | 3.631657 | 15.9743 | 2.67E-46 | 1.04E-43 | 94.4896 |
| RACGAP1 | 1.0058 | 4.050205 | 15.9195 | 4.72E-46 | 1.82E-43 | 93.9237 |
| FANCC | 0.79135 | 2.205319 | 15.8841 | 6.83E-46 | 2.62E-43 | 93.5572 |
| KIF18A | 0.94987 | 0.976464 | 15.8692 | 7.98E-46 | 3.04E-43 | 93.4033 |
| CDCA4 | 0.86399 | 2.764281 | 15.8529 | 9.45E-46 | 3.57E-43 | 93.2349 |
| SGO2 | 0.91172 | 1.897193 | 15.8465 | 1.01E-45 | 3.79E-43 | 93.1689 |
| FEN1 | 0.76693 | 5.133955 | 15.8358 | 1.13E-45 | 4.21E-43 | 93.0594 |
| E2F7 | 1.06696 | 0.877468 | 15.808 | 1.51E-45 | 5.58E-43 | 92.7721 |
| KPNA2 | 0.9353 | 5.592686 | 15.7908 | 1.80E-45 | 6.64E-43 | 92.5947 |
| TUBB | 0.74964 | 8.781661 | 15.7864 | 1.89E-45 | 6.90E-43 | 92.5495 |
| RTKN2 | 0.87459 | 1.201509 | 15.7175 | 3.87E-45 | 1.40E-42 | 91.8395 |
| RFC2 | 0.7118 | 5.197461 | 15.6509 | 7.71E-45 | 2.78E-42 | 91.155 |
| MCM5 | 0.81749 | 4.03472 | 15.6251 | 1.01E-44 | 3.61E-42 | 90.8895 |
| FAM83D | 1.26315 | 1.78354 | 15.5554 | 2.08E-44 | 7.39E-42 | 90.1741 |
| GAS2L3 | 1.0871 | 1.151583 | 15.5406 | 2.42E-44 | 8.55E-42 | 90.0228 |
| C5orf34 | 0.73542 | 1.629714 | 15.5373 | 2.50E-44 | 8.80E-42 | 89.9882 |
| DEPDC1 | 0.99823 | 0.770522 | 15.533 | 2.62E-44 | 9.14E-42 | 89.9442 |
| E2F8 | 0.8243 | 0.758065 | 15.3735 | 1.36E-43 | 4.72E-41 | 88.3119 |
| KIF18B | 1.41126 | 2.641614 | 15.2607 | 4.35E-43 | 1.50E-40 | 87.1606 |
| DBF4 | 0.80582 | 2.72411 | 15.2476 | 4.97E-43 | 1.70E-40 | 87.0271 |
| MCM6 | 0.81871 | 4.629359 | 15.166 | 1.15E-42 | 3.91E-40 | 86.1964 |
| XRCC2 | 0.91489 | 1.464416 | 15.1247 | 1.76E-42 | 5.94E-40 | 85.7764 |
| CENPL | 0.67834 | 2.03032 | 15.0621 | 3.34E-42 | 1.12E-39 | 85.1404 |
| LRR1 | 0.72335 | 2.641016 | 15.0329 | 4.51E-42 | 1.51E-39 | 84.8435 |
| RFWD3 | 0.73889 | 3.465605 | 15.0248 | 4.89E-42 | 1.62E-39 | 84.762 |
| ARHGEF39 | 0.58088 | 0.933505 | 15.018 | 5.25E-42 | 1.73E-39 | 84.6924 |
| MCM4 | 0.89947 | 4.814891 | 15.0057 | 5.95E-42 | 1.95E-39 | 84.5677 |
| NCAPG2 | 0.85026 | 3.301794 | 15.0035 | 6.08E-42 | 1.98E-39 | 84.5459 |
| IKBIP | 0.84756 | 2.676219 | 14.9962 | 6.55E-42 | 2.13E-39 | 84.4721 |
| TONSL | 0.84098 | 2.378196 | 14.9833 | 7.48E-42 | 2.41E-39 | 84.3415 |
| FBXO5 | 0.93125 | 2.900005 | 14.982 | 7.58E-42 | 2.43E-39 | 84.3284 |
| WEE1 | 1.20222 | 2.484842 | 14.979 | 7.82E-42 | 2.49E-39 | 84.2979 |
| LMNB2 | 0.79955 | 5.082778 | 14.9201 | 1.43E-41 | 4.52E-39 | 83.7011 |
| MCM3 | 0.70015 | 5.674403 | 14.7866 | 5.56E-41 | 1.75E-38 | 82.3537 |
| ZWILCH | 0.81048 | 2.541989 | 14.7775 | 6.10E-41 | 1.91E-38 | 82.2614 |
| POLE2 | 0.73507 | 1.508822 | 14.7563 | 7.58E-41 | 2.36E-38 | 82.0476 |
| CENPO | 0.68491 | 3.332151 | 14.7458 | 8.43E-41 | 2.61E-38 | 81.9423 |
| ATAD2 | 0.87682 | 3.56733 | 14.7433 | 8.64E-41 | 2.66E-38 | 81.9168 |
| RECQL4 | 0.95338 | 3.387928 | 14.6929 | 1.44E-40 | 4.42E-38 | 81.4095 |
| WDHD1 | 0.74569 | 1.956704 | 14.6766 | 1.70E-40 | 5.18E-38 | 81.2455 |
| CCDC15 | 0.64983 | 1.570535 | 14.6452 | 2.34E-40 | 7.07E-38 | 80.9305 |
| GINS4 | 0.68907 | 1.352755 | 14.6449 | 2.35E-40 | 7.07E-38 | 80.9277 |
| MXD3 | 1.05791 | 2.218622 | 14.6235 | 2.92E-40 | 8.74E-38 | 80.7119 |
| SNRPB | 0.63546 | 7.415729 | 14.5984 | 3.76E-40 | 1.12E-37 | 80.4602 |
| MCM8 | 0.78623 | 2.131316 | 14.5372 | 6.98E-40 | 2.07E-37 | 79.8472 |
| ODF2 | 0.62804 | 4.298456 | 14.4991 | 1.03E-39 | 3.03E-37 | 79.4653 |
| SMC4 | 1.12478 | 2.469226 | 14.4742 | 1.32E-39 | 3.87E-37 | 79.2164 |
| CKS1B | 0.70003 | 3.25976 | 14.456 | 1.59E-39 | 4.63E-37 | 79.0339 |
| STIL | 0.81404 | 1.407518 | 14.4052 | 2.65E-39 | 7.68E-37 | 78.5268 |
| FAAP24 | 0.64892 | 1.698821 | 14.3518 | 4.54E-39 | 1.31E-36 | 77.994 |
| RFC4 | 0.68505 | 3.905579 | 14.3165 | 6.47E-39 | 1.86E-36 | 77.642 |
| MTFR2 | 0.67424 | 1.028936 | 14.2879 | 8.63E-39 | 2.46E-36 | 77.3571 |
| PRIM2 | 0.57709 | 2.516918 | 14.2391 | 1.41E-38 | 4.00E-36 | 76.8727 |
| AC092718.4 | 0.91964 | 3.185686 | 14.173 | 2.73E-38 | 7.72E-36 | 76.2156 |
| ZNF90 | 0.59283 | 1.203203 | 14.1703 | 2.81E-38 | 7.88E-36 | 76.1892 |
| DNMT1 | 0.72898 | 4.79685 | 14.1649 | 2.96E-38 | 8.28E-36 | 76.1359 |
| CENPN | 0.711 | 2.553642 | 14.138 | 3.87E-38 | 1.08E-35 | 75.8695 |
| TEDC2 | 0.8293 | 2.292758 | 13.9779 | 1.92E-37 | 5.30E-35 | 74.2862 |
| RCC1 | 0.94546 | 3.96526 | 13.9047 | 3.97E-37 | 1.09E-34 | 73.5654 |
| MGME1 | 0.63461 | 4.327572 | 13.8339 | 8.02E-37 | 2.19E-34 | 72.8686 |
| SDC1 | 1.16376 | 2.163034 | 13.8098 | 1.02E-36 | 2.76E-34 | 72.6326 |
| CASP2 | 0.69247 | 3.590564 | 13.7739 | 1.45E-36 | 3.92E-34 | 72.28 |
| CDC7 | 0.87322 | 3.490338 | 13.7729 | 1.47E-36 | 3.94E-34 | 72.2704 |
| WDR76 | 0.89393 | 2.811579 | 13.7498 | 1.84E-36 | 4.93E-34 | 72.0442 |
| POLE | 0.72608 | 2.445523 | 13.7374 | 2.08E-36 | 5.55E-34 | 71.9224 |
| CKAP2 | 0.91721 | 4.267233 | 13.7097 | 2.74E-36 | 7.26E-34 | 71.6514 |
| CDKN2C | 1.37368 | 4.363295 | 13.6979 | 3.08E-36 | 8.11E-34 | 71.5363 |
| POLD1 | 0.77245 | 3.427404 | 13.6672 | 4.17E-36 | 1.09E-33 | 71.2361 |
| NKIRAS2 | 0.63346 | 4.671914 | 13.6503 | 4.92E-36 | 1.28E-33 | 71.0716 |
| MIS18A | 0.58103 | 4.029717 | 13.5197 | 1.78E-35 | 4.62E-33 | 69.7989 |
| TMPO-AS1 | 0.60665 | 1.542235 | 13.5109 | 1.94E-35 | 5.02E-33 | 69.7131 |
| RBL1 | 0.70224 | 2.776908 | 13.5095 | 1.97E-35 | 5.06E-33 | 69.6995 |
| NEMP1 | 0.76325 | 3.665335 | 13.5071 | 2.01E-35 | 5.16E-33 | 69.6764 |
| COQ2 | 0.54543 | 3.218008 | 13.4897 | 2.39E-35 | 6.09E-33 | 69.5075 |
| MMP2 | 1.16004 | 4.856895 | 13.3834 | 6.77E-35 | 1.71E-32 | 68.4758 |
| DDX39A | 0.66486 | 5.494411 | 13.3079 | 1.42E-34 | 3.56E-32 | 67.7463 |
| KDELR2 | 0.62919 | 6.45647 | 13.2869 | 1.74E-34 | 4.35E-32 | 67.5439 |
| HNRNPAB | 0.5646 | 7.056557 | 13.2705 | 2.04E-34 | 5.07E-32 | 67.386 |
| BRCA1 | 0.72169 | 2.310533 | 13.2671 | 2.11E-34 | 5.22E-32 | 67.3524 |
| TMPO | 0.69348 | 4.503526 | 13.2519 | 2.44E-34 | 6.03E-32 | 67.2058 |
| KIF20B | 0.63027 | 1.848175 | 13.2351 | 2.87E-34 | 7.06E-32 | 67.0448 |
| KNSTRN | 0.67483 | 3.436277 | 13.2321 | 2.96E-34 | 7.25E-32 | 67.0153 |
| PTBP1 | 0.67511 | 5.928092 | 13.209 | 3.71E-34 | 9.03E-32 | 66.7932 |
| NCAPD2 | 0.77665 | 4.845041 | 13.1763 | 5.09E-34 | 1.23E-31 | 66.4787 |
| SPC25 | 1.28084 | 2.757687 | 13.1631 | 5.79E-34 | 1.40E-31 | 66.3518 |
| PRR11 | 0.92708 | 2.816904 | 13.1582 | 6.07E-34 | 1.46E-31 | 66.3045 |
| RFC3 | 0.70616 | 3.483987 | 13.1124 | 9.46E-34 | 2.25E-31 | 65.8655 |
| PKN3 | 0.67091 | 2.680462 | 13.1116 | 9.54E-34 | 2.26E-31 | 65.8574 |
| MCM7 | 0.80788 | 6.427643 | 13.0909 | 1.17E-33 | 2.75E-31 | 65.6587 |
| CIP2A | 0.71709 | 2.020937 | 13.0697 | 1.43E-33 | 3.35E-31 | 65.456 |
| TOP3A | 0.50395 | 3.934503 | 13.0536 | 1.67E-33 | 3.90E-31 | 65.3019 |
| MEST | 1.13216 | 5.083993 | 13.0469 | 1.78E-33 | 4.14E-31 | 65.2382 |
| HAUS1 | 0.60473 | 4.178325 | 13.012 | 2.50E-33 | 5.78E-31 | 64.904 |
| GEN1 | 0.62474 | 1.590542 | 12.9738 | 3.61E-33 | 8.32E-31 | 64.5394 |
| H2AZ1 | 0.53498 | 7.129538 | 12.9549 | 4.33E-33 | 9.90E-31 | 64.3588 |
| CTPS1 | 0.6939 | 3.763824 | 12.9218 | 5.96E-33 | 1.35E-30 | 64.0437 |
| RAD18 | 0.60703 | 2.585541 | 12.8374 | 1.34E-32 | 3.01E-30 | 63.2414 |
| POLD3 | 0.51492 | 3.323983 | 12.8091 | 1.76E-32 | 3.93E-30 | 62.9728 |
| CDCA7 | 1.10247 | 2.943438 | 12.7952 | 2.01E-32 | 4.46E-30 | 62.8408 |
| GGH | 0.78204 | 3.813639 | 12.7642 | 2.70E-32 | 5.98E-30 | 62.547 |
| LIN9 | 0.58349 | 2.276969 | 12.7446 | 3.26E-32 | 7.18E-30 | 62.3615 |
| APOBEC3B | 0.78783 | 1.292577 | 12.7165 | 4.26E-32 | 9.35E-30 | 62.0962 |
| CCDC77 | 0.60324 | 3.129268 | 12.6917 | 5.40E-32 | 1.18E-29 | 61.8616 |
| BORA | 0.54611 | 1.65134 | 12.6489 | 8.13E-32 | 1.77E-29 | 61.4577 |
| CHST14 | 0.6445 | 4.3409 | 12.6368 | 9.12E-32 | 1.97E-29 | 61.3443 |
| AC025176.1 | 0.74838 | 0.61378 | 12.6354 | 9.24E-32 | 1.99E-29 | 61.3304 |
| ATAD5 | 0.59939 | 1.671542 | 12.5694 | 1.73E-31 | 3.72E-29 | 60.7097 |
| AC091057.1 | 0.64639 | 0.840037 | 12.5685 | 1.75E-31 | 3.74E-29 | 60.7014 |
| MASTL | 0.6208 | 2.897278 | 12.5256 | 2.62E-31 | 5.57E-29 | 60.2986 |
| DTYMK | 0.64332 | 4.903739 | 12.5229 | 2.69E-31 | 5.69E-29 | 60.273 |
| CBX3 | 0.5084 | 6.764164 | 12.4784 | 4.10E-31 | 8.64E-29 | 59.8562 |
| GAS1 | 1.12373 | 4.279858 | 12.4095 | 7.86E-31 | 1.64E-28 | 59.2123 |
| FANCG | 0.60156 | 4.011992 | 12.3947 | 9.04E-31 | 1.88E-28 | 59.0742 |
| SMC2 | 0.72719 | 4.028361 | 12.3827 | 1.01E-30 | 2.09E-28 | 58.9624 |
| HMGB2 | 0.80299 | 5.978076 | 12.3718 | 1.12E-30 | 2.31E-28 | 58.8602 |
| LIG1 | 0.68081 | 3.648605 | 12.3561 | 1.30E-30 | 2.67E-28 | 58.714 |
| ALYREF | 0.57716 | 6.646291 | 12.3475 | 1.41E-30 | 2.88E-28 | 58.6343 |
| NCAPD3 | 0.62671 | 3.562738 | 12.3269 | 1.71E-30 | 3.49E-28 | 58.4422 |
| FOXN4 | 0.64247 | 0.760684 | 12.3204 | 1.82E-30 | 3.69E-28 | 58.3818 |
| C18orf54 | 0.65167 | 2.227421 | 12.2764 | 2.75E-30 | 5.56E-28 | 57.9734 |
| DSCC1 | 0.61981 | 2.930166 | 12.276 | 2.76E-30 | 5.56E-28 | 57.9695 |
| XRN2 | 0.57283 | 5.944902 | 12.2519 | 3.46E-30 | 6.92E-28 | 57.7457 |
| EML4 | 0.57841 | 3.651178 | 12.2149 | 4.90E-30 | 9.72E-28 | 57.4028 |
| RHNO1 | 0.54704 | 5.098933 | 12.1963 | 5.83E-30 | 1.15E-27 | 57.2305 |
| PDIA4 | 0.78411 | 6.465939 | 12.174 | 7.18E-30 | 1.41E-27 | 57.0245 |
| CEP135 | 0.63574 | 1.726993 | 12.1655 | 7.77E-30 | 1.52E-27 | 56.9456 |
| NXT1 | 0.56719 | 4.984184 | 12.1377 | 1.01E-29 | 1.96E-27 | 56.6888 |
| RBBP8 | 0.75056 | 3.636841 | 12.134 | 1.04E-29 | 2.03E-27 | 56.6554 |
| AC012073.1 | 0.63413 | 1.717554 | 12.1031 | 1.39E-29 | 2.67E-27 | 56.3697 |
| C21orf58 | 0.68398 | 2.110729 | 12.1013 | 1.41E-29 | 2.70E-27 | 56.3536 |
| GLA | 0.55639 | 4.207822 | 12.0531 | 2.21E-29 | 4.20E-27 | 55.9097 |
| ALDH2 | -0.6571 | 6.598337 | -12.04 | 2.51E-29 | 4.74E-27 | 55.7869 |
| EMC3-AS1 | 0.52657 | 1.488041 | 12.0085 | 3.35E-29 | 6.27E-27 | 55.5003 |
| VRK1 | 0.51561 | 3.317154 | 11.9847 | 4.18E-29 | 7.76E-27 | 55.2818 |
| TP53I3 | 0.88119 | 4.097424 | 11.9572 | 5.39E-29 | 9.98E-27 | 55.03 |
| REXO5 | 0.62319 | 2.604042 | 11.9077 | 8.52E-29 | 1.56E-26 | 54.5766 |
| CCDC18 | 0.62069 | 1.288294 | 11.9068 | 8.59E-29 | 1.57E-26 | 54.5684 |
| CBX7 | -0.6951 | 4.777827 | -11.899 | 9.22E-29 | 1.67E-26 | 54.4989 |
| C19orf48 | 0.66609 | 4.537642 | 11.869 | 1.22E-28 | 2.19E-26 | 54.2241 |
| TRIM24 | 0.65691 | 4.651188 | 11.8688 | 1.22E-28 | 2.19E-26 | 54.2224 |
| CARHSP1 | 0.66657 | 4.766089 | 11.8682 | 1.23E-28 | 2.20E-26 | 54.2167 |
| KIF22 | 0.59384 | 4.935128 | 11.8517 | 1.43E-28 | 2.55E-26 | 54.0659 |
| IDH1 | 0.66992 | 5.712688 | 11.821 | 1.89E-28 | 3.37E-26 | 53.7866 |
| ZNF486 | 0.56444 | 2.588048 | 11.751 | 3.60E-28 | 6.39E-26 | 53.1509 |
| DCLRE1B | 0.62019 | 3.479883 | 11.7369 | 4.10E-28 | 7.23E-26 | 53.0231 |
| CEP152 | 0.55001 | 1.252594 | 11.6243 | 1.15E-27 | 1.99E-25 | 52.0048 |
| RRM1 | 0.57031 | 5.330784 | 11.6093 | 1.32E-27 | 2.27E-25 | 51.8695 |
| C1orf112 | 0.51301 | 1.856325 | 11.6052 | 1.37E-27 | 2.35E-25 | 51.8326 |
| NDC1 | 0.58625 | 3.672418 | 11.5826 | 1.68E-27 | 2.85E-25 | 51.6293 |
| NUP205 | 0.61361 | 4.598062 | 11.5513 | 2.23E-27 | 3.72E-25 | 51.3479 |
| C4orf46 | 0.50929 | 2.968622 | 11.5411 | 2.45E-27 | 4.05E-25 | 51.2563 |
| PSMC3IP | 0.52619 | 3.041823 | 11.5189 | 3.00E-27 | 4.93E-25 | 51.0565 |
| FAAP100 | 0.56863 | 4.879146 | 11.5148 | 3.11E-27 | 5.10E-25 | 51.0195 |
| CD276 | 0.74241 | 4.812796 | 11.5088 | 3.28E-27 | 5.37E-25 | 50.9662 |
| RCC2 | 0.66021 | 6.260328 | 11.5038 | 3.44E-27 | 5.60E-25 | 50.9212 |
| NUP107 | 0.54217 | 3.494366 | 11.4802 | 4.25E-27 | 6.90E-25 | 50.7099 |
| PFN1 | 0.53266 | 8.559887 | 11.4577 | 5.22E-27 | 8.41E-25 | 50.5083 |
| BARD1 | 0.73188 | 2.806078 | 11.4414 | 6.05E-27 | 9.69E-25 | 50.3626 |
| DHFR | 0.73592 | 3.846521 | 11.4197 | 7.35E-27 | 1.16E-24 | 50.1691 |
| DESI2 | 0.57236 | 4.270905 | 11.3998 | 8.80E-27 | 1.39E-24 | 49.9913 |
| ZNF43 | 0.57295 | 3.390362 | 11.3739 | 1.11E-26 | 1.74E-24 | 49.7601 |
| ZNF724 | 0.59761 | 1.186245 | 11.3733 | 1.12E-26 | 1.75E-24 | 49.7552 |
| CCNF | 0.56544 | 2.450973 | 11.3308 | 1.64E-26 | 2.54E-24 | 49.3773 |
| INTS7 | 0.5927 | 3.869493 | 11.3118 | 1.94E-26 | 2.98E-24 | 49.2086 |
| AC011447.7 | 0.67923 | 1.973902 | 11.3082 | 2.01E-26 | 3.07E-24 | 49.1765 |
| XPO5 | 0.50541 | 4.542173 | 11.2711 | 2.80E-26 | 4.28E-24 | 48.847 |
| FIGNL1 | 0.5479 | 3.188704 | 11.2452 | 3.53E-26 | 5.36E-24 | 48.6178 |
| TMEM45A | 0.7434 | 2.319668 | 11.2289 | 4.08E-26 | 6.15E-24 | 48.4741 |
| DDX12P | 0.77233 | 1.484568 | 11.198 | 5.39E-26 | 8.07E-24 | 48.2007 |
| CKLF | 0.51667 | 3.912982 | 11.186 | 6.00E-26 | 8.95E-24 | 48.0948 |
| H2AX | 0.67275 | 6.661801 | 11.1829 | 6.17E-26 | 9.18E-24 | 48.0672 |
| ZIM2-AS1 | 0.62894 | 1.926232 | 11.1763 | 6.54E-26 | 9.71E-24 | 48.0096 |
| C19orf57 | 0.60222 | 3.134104 | 11.1554 | 7.88E-26 | 1.16E-23 | 47.8251 |
| ZGRF1 | 0.52794 | 1.638516 | 11.1492 | 8.32E-26 | 1.23E-23 | 47.7705 |
| SEC61A1 | 0.50496 | 7.54515 | 11.1129 | 1.15E-25 | 1.66E-23 | 47.4509 |
| COL4A1 | 1.55529 | 4.092979 | 11.1036 | 1.25E-25 | 1.80E-23 | 47.3698 |
| GJC1 | 0.82741 | 2.055578 | 11.0935 | 1.37E-25 | 1.95E-23 | 47.2812 |
| GUSB | 0.57444 | 4.783109 | 11.0881 | 1.43E-25 | 2.04E-23 | 47.233 |
| NUDT1 | 0.54807 | 4.818688 | 11.0855 | 1.47E-25 | 2.08E-23 | 47.2107 |
| PUS7 | 0.56006 | 3.169531 | 11.0722 | 1.65E-25 | 2.34E-23 | 47.0942 |
| CDK4 | 0.88543 | 6.24616 | 11.0645 | 1.77E-25 | 2.50E-23 | 47.0262 |
| BMP1 | 0.59622 | 3.803299 | 11.0594 | 1.85E-25 | 2.61E-23 | 46.9814 |
| EIF4EBP1 | 0.79265 | 5.754211 | 11.056 | 1.91E-25 | 2.68E-23 | 46.9522 |
| CALU | 0.7246 | 6.04556 | 11.0553 | 1.92E-25 | 2.69E-23 | 46.9461 |
| SMARCD1 | 0.51034 | 5.751049 | 11.0386 | 2.22E-25 | 3.10E-23 | 46.7997 |
| AC026401.3 | 0.92716 | 3.224583 | 11.0204 | 2.61E-25 | 3.63E-23 | 46.6401 |
| CCT6A | 0.53866 | 7.085704 | 11.0057 | 2.98E-25 | 4.12E-23 | 46.5118 |
| KIF24 | 0.58222 | 1.584216 | 10.9936 | 3.31E-25 | 4.55E-23 | 46.4064 |
| PRIM1 | 0.56883 | 3.281811 | 10.9886 | 3.46E-25 | 4.74E-23 | 46.3628 |
| TICRR | 0.62322 | 1.488986 | 10.9723 | 4.00E-25 | 5.42E-23 | 46.2201 |
| THOC6 | 0.53785 | 4.68439 | 10.9581 | 4.53E-25 | 6.12E-23 | 46.0964 |
| COL3A1 | 1.59068 | 2.895843 | 10.9372 | 5.45E-25 | 7.28E-23 | 45.9146 |
| NOP2 | 0.51458 | 4.568889 | 10.928 | 5.91E-25 | 7.82E-23 | 45.8339 |
| NR2C2AP | 0.50722 | 4.747991 | 10.9162 | 6.55E-25 | 8.57E-23 | 45.7315 |
| MYO9B | 0.59695 | 5.342057 | 10.9128 | 6.75E-25 | 8.81E-23 | 45.7019 |
| BAX | 0.55619 | 5.555291 | 10.9123 | 6.78E-25 | 8.83E-23 | 45.698 |
| E2F3 | 0.63216 | 4.012424 | 10.895 | 7.90E-25 | 1.02E-22 | 45.547 |
| DEK | 0.51696 | 5.961526 | 10.8947 | 7.92E-25 | 1.02E-22 | 45.5448 |
| COLGALT1 | 0.58616 | 5.090985 | 10.892 | 8.11E-25 | 1.04E-22 | 45.5209 |
| TP53 | 0.78582 | 5.448271 | 10.8906 | 8.21E-25 | 1.05E-22 | 45.5087 |
| WDR34 | 0.6028 | 5.590765 | 10.8515 | 1.16E-24 | 1.46E-22 | 45.1697 |
| MRO | -0.9672 | 5.440223 | -10.821 | 1.51E-24 | 1.87E-22 | 44.9052 |
| AL035461.3 | 0.68131 | 2.655876 | 10.807 | 1.71E-24 | 2.10E-22 | 44.7847 |
| COL4A2 | 1.37026 | 4.475923 | 10.7611 | 2.55E-24 | 3.09E-22 | 44.389 |
| XXYLT1 | 0.51104 | 3.622094 | 10.7565 | 2.66E-24 | 3.19E-22 | 44.349 |
| ORAI1 | 0.56072 | 4.03921 | 10.7519 | 2.77E-24 | 3.32E-22 | 44.3092 |
| TPM4 | 0.60297 | 5.830605 | 10.732 | 3.29E-24 | 3.92E-22 | 44.138 |
| CDC25B | 0.54739 | 5.834465 | 10.7217 | 3.60E-24 | 4.28E-22 | 44.0496 |
| HAUS5 | 0.58922 | 3.584366 | 10.6908 | 4.71E-24 | 5.55E-22 | 43.784 |
| JPT1 | 0.62281 | 6.311491 | 10.6791 | 5.21E-24 | 6.12E-22 | 43.6834 |
| ALG6 | 0.52356 | 2.872595 | 10.6653 | 5.88E-24 | 6.85E-22 | 43.5648 |
| MAN2B1 | 0.63564 | 4.778668 | 10.649 | 6.77E-24 | 7.86E-22 | 43.4251 |
| FAM126A | 0.68714 | 2.571375 | 10.6177 | 8.88E-24 | 1.02E-21 | 43.1574 |
| STK38 | 0.53209 | 4.644124 | 10.6054 | 9.87E-24 | 1.13E-21 | 43.0524 |
| KIRREL1 | 0.72381 | 3.193358 | 10.5989 | 1.04E-23 | 1.19E-21 | 42.9966 |
| PTMS | 0.61339 | 9.483033 | 10.5975 | 1.06E-23 | 1.20E-21 | 42.9851 |
| NASP | 0.57966 | 5.475785 | 10.5884 | 1.14E-23 | 1.29E-21 | 42.9067 |
| ZYX | 0.67831 | 6.832735 | 10.5683 | 1.36E-23 | 1.52E-21 | 42.7351 |
| LINC01224 | 0.60899 | 0.564211 | 10.5516 | 1.57E-23 | 1.75E-21 | 42.5927 |
| CMTM6 | 0.58799 | 4.906582 | 10.5513 | 1.58E-23 | 1.75E-21 | 42.591 |
| ZNF736 | 0.53628 | 2.862204 | 10.5503 | 1.59E-23 | 1.76E-21 | 42.5824 |
| HMGN2 | 0.5762 | 7.279494 | 10.5183 | 2.09E-23 | 2.29E-21 | 42.3098 |
| DHRSX | 0.5242 | 4.137737 | 10.4917 | 2.63E-23 | 2.87E-21 | 42.0835 |
| AC099850.4 | 0.90928 | 3.04298 | 10.4752 | 3.03E-23 | 3.30E-21 | 41.9435 |
| PLEKHG2 | 0.72048 | 3.523084 | 10.4656 | 3.29E-23 | 3.57E-21 | 41.8626 |
| BNIP3P17 | 0.55117 | 1.296857 | 10.4534 | 3.66E-23 | 3.95E-21 | 41.7589 |
| FAM111A | 0.71845 | 3.287099 | 10.4511 | 3.73E-23 | 4.02E-21 | 41.74 |
| DONSON | 0.52227 | 3.621304 | 10.4228 | 4.75E-23 | 5.03E-21 | 41.5002 |
| GPX7 | 0.71789 | 3.866759 | 10.3833 | 6.67E-23 | 6.92E-21 | 41.1665 |
| TMEM106C | 0.62517 | 4.850956 | 10.3743 | 7.20E-23 | 7.44E-21 | 41.0909 |
| ZNF85 | 0.52349 | 2.48598 | 10.3728 | 7.29E-23 | 7.53E-21 | 41.078 |
| H3C10 | 0.66043 | 1.04166 | 10.3627 | 7.95E-23 | 8.17E-21 | 40.993 |
| BAZ1A | 0.61799 | 3.44833 | 10.349 | 8.93E-23 | 9.10E-21 | 40.8778 |
| IFNGR2 | 0.53026 | 5.586358 | 10.3484 | 8.98E-23 | 9.13E-21 | 40.8725 |
| SPIN4 | 0.53705 | 2.309268 | 10.3381 | 9.80E-23 | 9.91E-21 | 40.7856 |
| MAD2L2 | 0.61284 | 5.287246 | 10.3283 | 1.07E-22 | 1.08E-20 | 40.7036 |
| AL391069.2 | 0.62614 | 3.973528 | 10.3213 | 1.13E-22 | 1.14E-20 | 40.6447 |
| ZNF107 | 0.59636 | 3.082654 | 10.2978 | 1.38E-22 | 1.38E-20 | 40.4471 |
| JAG1 | 0.8341 | 4.225007 | 10.2961 | 1.40E-22 | 1.39E-20 | 40.433 |
| UBE2S | 0.6659 | 4.034526 | 10.2956 | 1.41E-22 | 1.40E-20 | 40.4286 |
| PTK7 | 0.62676 | 3.791052 | 10.2871 | 1.51E-22 | 1.49E-20 | 40.358 |
| ODC1 | 0.53361 | 6.969857 | 10.2712 | 1.73E-22 | 1.69E-20 | 40.2248 |
| PFAS | 0.50873 | 4.330016 | 10.2694 | 1.76E-22 | 1.71E-20 | 40.2091 |
| NUP188 | 0.51065 | 4.913892 | 10.2496 | 2.08E-22 | 2.01E-20 | 40.0434 |
| ADHFE1 | -0.6562 | 4.501171 | -10.225 | 2.57E-22 | 2.45E-20 | 39.8361 |
| TMEM131L | 0.58345 | 2.731958 | 10.2196 | 2.68E-22 | 2.55E-20 | 39.7931 |
| TGFB1I1 | 0.70628 | 3.110305 | 10.1812 | 3.71E-22 | 3.50E-20 | 39.4729 |
| BZW2 | 0.58566 | 5.013351 | 10.1698 | 4.08E-22 | 3.83E-20 | 39.3777 |
| YBX1 | 0.62051 | 8.970929 | 10.1514 | 4.77E-22 | 4.46E-20 | 39.225 |
| CTNNAL1 | 0.53857 | 4.292895 | 10.1383 | 5.32E-22 | 4.95E-20 | 39.1156 |
| PGM2 | 0.57624 | 4.052959 | 10.1265 | 5.88E-22 | 5.40E-20 | 39.0181 |
| EFNB1 | 0.52503 | 4.327619 | 10.1181 | 6.31E-22 | 5.78E-20 | 38.948 |
| HYAL2 | 0.52391 | 4.772188 | 10.1056 | 7.01E-22 | 6.40E-20 | 38.8447 |
| CMTM3 | 0.67025 | 5.542587 | 10.1034 | 7.13E-22 | 6.51E-20 | 38.8268 |
| SKP2 | 0.52627 | 3.717075 | 10.088 | 8.12E-22 | 7.34E-20 | 38.6985 |
| ENPEP | 0.72407 | 1.328918 | 10.0817 | 8.56E-22 | 7.70E-20 | 38.647 |
| CHAF1B | 0.77104 | 1.800795 | 10.0637 | 9.96E-22 | 8.90E-20 | 38.4977 |
| COL1A2 | 1.13232 | 3.450604 | 10.0625 | 1.01E-21 | 8.98E-20 | 38.4875 |
| LDHD | -0.623 | 4.612273 | -10.025 | 1.38E-21 | 1.21E-19 | 38.1772 |
| CDCA7L | 0.96538 | 3.3403 | 10.0077 | 1.59E-21 | 1.39E-19 | 38.0359 |
| ZNF93 | 0.55258 | 2.243119 | 9.99667 | 1.74E-21 | 1.51E-19 | 37.9449 |
| NT5DC2 | 0.60528 | 5.204717 | 9.99412 | 1.78E-21 | 1.54E-19 | 37.9238 |
| SLC2A4 | -0.7763 | 1.952185 | -9.9758 | 2.07E-21 | 1.79E-19 | 37.7734 |
| HTRA3 | 0.81139 | 1.179284 | 9.97579 | 2.08E-21 | 1.79E-19 | 37.7731 |
| PRDX4 | 0.50139 | 6.078823 | 9.96556 | 2.26E-21 | 1.94E-19 | 37.689 |
| PIF1 | 0.71942 | 1.463128 | 9.96118 | 2.34E-21 | 2.01E-19 | 37.6531 |
| TGFBR1 | 0.63813 | 4.704927 | 9.95842 | 2.40E-21 | 2.05E-19 | 37.6304 |
| P3H1 | 0.51784 | 3.008683 | 9.95733 | 2.42E-21 | 2.07E-19 | 37.6214 |
| CNTRL | 0.53844 | 2.870196 | 9.95694 | 2.43E-21 | 2.07E-19 | 37.6183 |
| MNS1 | 0.63182 | 3.089478 | 9.92728 | 3.11E-21 | 2.62E-19 | 37.375 |
| RAB42 | 0.75071 | 1.54573 | 9.91323 | 3.49E-21 | 2.93E-19 | 37.2599 |
| SLC30A7 | 0.52181 | 2.756453 | 9.90883 | 3.62E-21 | 3.03E-19 | 37.2239 |
| ETV6 | 0.51339 | 3.548154 | 9.90251 | 3.82E-21 | 3.17E-19 | 37.1722 |
| CCDC167 | 0.52374 | 6.767583 | 9.90211 | 3.83E-21 | 3.18E-19 | 37.169 |
| HSPG2 | 0.96364 | 2.370424 | 9.89987 | 3.90E-21 | 3.23E-19 | 37.1506 |
| TCF3 | 0.55813 | 5.671663 | 9.89242 | 4.15E-21 | 3.43E-19 | 37.0897 |
| IQGAP2 | 1.0691 | 2.226323 | 9.8693 | 5.02E-21 | 4.13E-19 | 36.9008 |
| LSM7 | 0.53252 | 6.196242 | 9.85804 | 5.52E-21 | 4.49E-19 | 36.8089 |
| AKNAD1 | 0.51654 | 0.995481 | 9.84234 | 6.28E-21 | 5.08E-19 | 36.681 |
| AC010729.2 | 0.55639 | 1.224008 | 9.83217 | 6.83E-21 | 5.49E-19 | 36.5981 |
| SBNO2 | 0.56386 | 3.755338 | 9.82762 | 7.09E-21 | 5.68E-19 | 36.5611 |
| KDM4A | 0.50639 | 4.989873 | 9.82674 | 7.14E-21 | 5.71E-19 | 36.554 |
| AC007240.1 | 0.55742 | 0.402735 | 9.81825 | 7.66E-21 | 6.10E-19 | 36.4849 |
| NRAS | 0.5006 | 5.327374 | 9.81468 | 7.89E-21 | 6.27E-19 | 36.4558 |
| TTF2 | 0.50477 | 1.910391 | 9.8076 | 8.36E-21 | 6.60E-19 | 36.3982 |
| DRAXIN | 0.94378 | 2.467875 | 9.77874 | 1.06E-20 | 8.23E-19 | 36.1639 |
| CD101 | 0.70239 | 1.317013 | 9.76385 | 1.20E-20 | 9.26E-19 | 36.0432 |
| RPS2 | 0.56939 | 9.127467 | 9.75002 | 1.34E-20 | 1.03E-18 | 35.9312 |
| MAP3K1 | 0.67112 | 3.338883 | 9.74472 | 1.40E-20 | 1.07E-18 | 35.8882 |
| ETNPPL | -1.5684 | 6.574444 | -9.732 | 1.56E-20 | 1.18E-18 | 35.7857 |
| CCND2 | 0.82238 | 6.677452 | 9.73084 | 1.57E-20 | 1.19E-18 | 35.7759 |
| AL137025.1 | -0.6133 | 1.641776 | -9.7265 | 1.63E-20 | 1.22E-18 | 35.741 |
| DNMBP-AS1 | -0.8208 | 1.45502 | -9.7102 | 1.86E-20 | 1.39E-18 | 35.6088 |
| TMSB15A | 1.30289 | 3.682333 | 9.70212 | 1.99E-20 | 1.47E-18 | 35.5439 |
| SPINK8 | 0.89147 | 2.335905 | 9.65602 | 2.90E-20 | 2.11E-18 | 35.1725 |
| ACTL6A | 0.50031 | 4.604242 | 9.64428 | 3.19E-20 | 2.30E-18 | 35.0781 |
| CCDC34 | 0.50955 | 3.185602 | 9.64234 | 3.24E-20 | 2.34E-18 | 35.0625 |
| LAPTM4B | 0.50777 | 7.197817 | 9.63941 | 3.32E-20 | 2.39E-18 | 35.039 |
| CD93 | 1.04046 | 3.190174 | 9.62225 | 3.82E-20 | 2.72E-18 | 34.9011 |
| NDRG2 | -0.7439 | 9.358185 | -9.6217 | 3.83E-20 | 2.73E-18 | 34.897 |
| YBX1P10 | 0.60126 | 3.072662 | 9.62089 | 3.86E-20 | 2.74E-18 | 34.8902 |
| RNF122 | 0.75532 | 3.934552 | 9.61426 | 4.07E-20 | 2.88E-18 | 34.837 |
| PTN | 0.80612 | 9.333912 | 9.60995 | 4.22E-20 | 2.98E-18 | 34.8024 |
| DPEP1 | 1.01286 | 1.047969 | 9.59924 | 4.60E-20 | 3.23E-18 | 34.7166 |
| PTGFRN | 0.87054 | 4.429092 | 9.54922 | 6.91E-20 | 4.73E-18 | 34.3165 |
| CRISPLD1 | 0.88768 | 5.013715 | 9.51949 | 8.78E-20 | 5.94E-18 | 34.0794 |
| COL1A1 | 1.3734 | 2.61381 | 9.51454 | 9.14E-20 | 6.18E-18 | 34.04 |
| CPE | -0.7462 | 10.42988 | -9.4909 | 1.11E-19 | 7.38E-18 | 33.8519 |
| PPP1R18 | 0.55928 | 5.543133 | 9.48842 | 1.13E-19 | 7.50E-18 | 33.8321 |
| CD248 | 0.98939 | 2.705331 | 9.48264 | 1.18E-19 | 7.83E-18 | 33.7861 |
| FNDC3B | 0.56792 | 2.506928 | 9.47603 | 1.25E-19 | 8.22E-18 | 33.7337 |
| ALDH6A1 | -0.5475 | 6.203405 | -9.4749 | 1.26E-19 | 8.28E-18 | 33.7247 |
| LINC02321 | 0.51244 | 0.980251 | 9.41361 | 2.06E-19 | 1.32E-17 | 33.239 |
| CHTF18 | 0.68307 | 3.194022 | 9.40739 | 2.17E-19 | 1.38E-17 | 33.1897 |
| IL1RAP | 0.90431 | 2.425706 | 9.38692 | 2.55E-19 | 1.61E-17 | 33.0281 |
| ZNF300 | 0.72291 | 3.758576 | 9.35965 | 3.17E-19 | 1.98E-17 | 32.8131 |
| AC069499.2 | 0.54857 | 1.224626 | 9.35683 | 3.25E-19 | 2.02E-17 | 32.7909 |
| BGN | 0.98096 | 6.503714 | 9.34206 | 3.65E-19 | 2.26E-17 | 32.6747 |
| PDIA5 | 0.6382 | 2.148924 | 9.32933 | 4.04E-19 | 2.49E-17 | 32.5746 |
| SELENON | 0.55727 | 7.069672 | 9.31982 | 4.36E-19 | 2.68E-17 | 32.4998 |
| TP73 | 0.69114 | 1.052887 | 9.30346 | 4.97E-19 | 3.04E-17 | 32.3714 |
| ITGB3BP | 0.51011 | 2.357006 | 9.30184 | 5.03E-19 | 3.07E-17 | 32.3588 |
| C1QTNF6 | 0.50661 | 2.315082 | 9.3018 | 5.04E-19 | 3.07E-17 | 32.3584 |
| RECQL | 0.64776 | 3.863868 | 9.28319 | 5.84E-19 | 3.52E-17 | 32.2126 |
| EPHB2 | 0.69814 | 2.766203 | 9.2772 | 6.12E-19 | 3.68E-17 | 32.1657 |
| TUBA1C | 0.79298 | 3.187109 | 9.24208 | 8.09E-19 | 4.77E-17 | 31.8911 |
| DOT1L | 0.6371 | 3.906889 | 9.22588 | 9.20E-19 | 5.35E-17 | 31.7647 |
| TSPAN12 | 0.83329 | 4.794125 | 9.22351 | 9.37E-19 | 5.45E-17 | 31.7462 |
| AC093673.1 | 0.65724 | 4.837239 | 9.21275 | 1.02E-18 | 5.91E-17 | 31.6624 |
| ALDH16A1 | 0.50248 | 3.647529 | 9.20486 | 1.09E-18 | 6.24E-17 | 31.6009 |
| REEP4 | 0.58143 | 3.975426 | 9.19565 | 1.17E-18 | 6.69E-17 | 31.5293 |
| HMGB3 | 0.53077 | 5.137356 | 9.15521 | 1.61E-18 | 9.08E-17 | 31.2151 |
| PCOLCE | 0.81295 | 2.937605 | 9.15327 | 1.63E-18 | 9.20E-17 | 31.2 |
| SMC5 | 0.51134 | 4.018117 | 9.15322 | 1.63E-18 | 9.20E-17 | 31.1997 |
| UBALD2 | 0.52129 | 6.278616 | 9.14984 | 1.68E-18 | 9.44E-17 | 31.1734 |
| HPSE2 | -1.394 | 3.5224 | -9.1461 | 1.73E-18 | 9.70E-17 | 31.1446 |
| CASP3 | 0.52144 | 4.967352 | 9.13703 | 1.85E-18 | 1.04E-16 | 31.0742 |
| TMEM51 | 0.59712 | 3.96951 | 9.13478 | 1.89E-18 | 1.05E-16 | 31.0567 |
| HNF4G | 0.51009 | 1.425357 | 9.1307 | 1.95E-18 | 1.09E-16 | 31.0251 |
| PIK3R3 | 0.52842 | 3.999373 | 9.11659 | 2.18E-18 | 1.20E-16 | 30.916 |
| SLC4A7 | 0.55266 | 2.751705 | 9.11417 | 2.22E-18 | 1.22E-16 | 30.8972 |
| KDM1A | 0.51216 | 5.816963 | 9.07803 | 2.95E-18 | 1.60E-16 | 30.6182 |
| METTL1 | 0.69222 | 3.787699 | 9.06476 | 3.27E-18 | 1.76E-16 | 30.5159 |
| NEDD1 | 0.54055 | 3.599146 | 9.05231 | 3.60E-18 | 1.92E-16 | 30.4201 |
| KLHDC8A | 1.03579 | 4.764474 | 9.04368 | 3.85E-18 | 2.04E-16 | 30.3537 |
| SERPINB8 | 0.57065 | 2.526972 | 9.01163 | 4.95E-18 | 2.58E-16 | 30.1076 |
| TTYH3 | 0.63551 | 6.328224 | 9.01042 | 4.99E-18 | 2.60E-16 | 30.0983 |
| CHIC2 | 0.54709 | 4.523164 | 8.98093 | 6.28E-18 | 3.22E-16 | 29.8723 |
| AC015540.1 | -0.861 | 3.137744 | -8.9793 | 6.36E-18 | 3.25E-16 | 29.8597 |
| PPP1R14B | 0.56627 | 6.522739 | 8.97299 | 6.68E-18 | 3.41E-16 | 29.8116 |
| IL18BP | 0.55674 | 3.636484 | 8.96973 | 6.85E-18 | 3.49E-16 | 29.7867 |
| UHRF1 | 0.81406 | 4.097236 | 8.87954 | 1.38E-17 | 6.70E-16 | 29.0995 |
| NEURL1B | 0.65404 | 4.027802 | 8.87396 | 1.44E-17 | 6.97E-16 | 29.0572 |
| ATP2A1-AS1 | 0.53029 | 2.501593 | 8.86695 | 1.52E-17 | 7.33E-16 | 29.004 |
| JPT2 | 0.5588 | 4.360031 | 8.84677 | 1.77E-17 | 8.52E-16 | 28.8511 |
| CAPN5 | 0.72908 | 4.835605 | 8.8263 | 2.07E-17 | 9.85E-16 | 28.6963 |
| SERPINH1 | 0.84284 | 4.26308 | 8.81114 | 2.33E-17 | 1.09E-15 | 28.5818 |
| GMIP | 0.51431 | 3.989402 | 8.79999 | 2.54E-17 | 1.19E-15 | 28.4976 |
| SLC24A4 | -0.9651 | 2.562702 | -8.794 | 2.66E-17 | 1.24E-15 | 28.4528 |
| TPD52L1 | -0.821 | 3.220105 | -8.7714 | 3.16E-17 | 1.46E-15 | 28.2826 |
| PTX3 | 0.94328 | 1.996733 | 8.76147 | 3.41E-17 | 1.56E-15 | 28.2076 |
| DGCR6 | -0.8351 | 3.606755 | -8.7476 | 3.79E-17 | 1.73E-15 | 28.1035 |
| S1PR2 | 0.56872 | 3.355496 | 8.73838 | 4.07E-17 | 1.85E-15 | 28.0342 |
| LAMC1 | 0.73836 | 4.215442 | 8.73073 | 4.31E-17 | 1.94E-15 | 27.9768 |
| CCNE2 | 0.5551 | 2.177713 | 8.72772 | 4.41E-17 | 1.99E-15 | 27.9542 |
| AGRN | 0.54773 | 5.940864 | 8.71209 | 4.97E-17 | 2.22E-15 | 27.8371 |
| SOX11 | 0.92696 | 4.173683 | 8.7035 | 5.30E-17 | 2.36E-15 | 27.7728 |
| BTG3 | 0.51701 | 5.084021 | 8.69588 | 5.62E-17 | 2.49E-15 | 27.7158 |
| NID1 | 0.82406 | 4.729207 | 8.69092 | 5.84E-17 | 2.57E-15 | 27.6787 |
| CDK6 | 0.88396 | 3.493505 | 8.6712 | 6.78E-17 | 2.95E-15 | 27.5314 |
| GRN | 0.5005 | 7.011392 | 8.66845 | 6.92E-17 | 3.00E-15 | 27.511 |
| DTX3L | 0.65041 | 4.112368 | 8.66786 | 6.95E-17 | 3.01E-15 | 27.5065 |
| ELN | 0.80771 | 5.094504 | 8.66 | 7.38E-17 | 3.19E-15 | 27.4479 |
| FSTL1 | 0.82178 | 4.585006 | 8.64961 | 7.98E-17 | 3.43E-15 | 27.3705 |
| H2BC9 | 0.529 | 0.428025 | 8.62234 | 9.81E-17 | 4.16E-15 | 27.1676 |
| ST14 | 0.86956 | 2.179745 | 8.61373 | 1.05E-16 | 4.43E-15 | 27.1036 |
| ACE | 0.68449 | 1.706208 | 8.58685 | 1.28E-16 | 5.36E-15 | 26.9043 |
| IGFBP2 | 1.35789 | 3.750105 | 8.58657 | 1.28E-16 | 5.37E-15 | 26.9022 |
| MIS18BP1 | 0.57553 | 2.437928 | 8.58237 | 1.33E-16 | 5.52E-15 | 26.871 |
| MT-RNR1 | -0.5896 | 12.94464 | -8.5809 | 1.34E-16 | 5.58E-15 | 26.8599 |
| NFIL3 | 0.556 | 4.959314 | 8.57506 | 1.40E-16 | 5.82E-15 | 26.8169 |
| WNT5A-AS1 | 0.64936 | 1.638604 | 8.57444 | 1.41E-16 | 5.84E-15 | 26.8123 |
| WASF3 | -0.5617 | 6.671669 | -8.5719 | 1.43E-16 | 5.94E-15 | 26.7936 |
| FBLIM1 | 0.70327 | 2.228987 | 8.5645 | 1.52E-16 | 6.26E-15 | 26.7388 |
| SHOX2 | 0.92488 | 0.678925 | 8.56301 | 1.53E-16 | 6.33E-15 | 26.7278 |
| AC027130.1 | -0.7571 | 2.39269 | -8.5437 | 1.77E-16 | 7.27E-15 | 26.5849 |
| GABRG1 | -1.1425 | 3.624659 | -8.5343 | 1.90E-16 | 7.78E-15 | 26.5159 |
| RAVER1 | 0.53784 | 4.41308 | 8.53322 | 1.92E-16 | 7.83E-15 | 26.5078 |
| SNRPGP10 | 0.74575 | 2.268941 | 8.52684 | 2.01E-16 | 8.20E-15 | 26.4607 |
| DSEL | 0.62174 | 3.978402 | 8.50645 | 2.34E-16 | 9.49E-15 | 26.3105 |
| PPP4R4 | -0.9225 | 2.464906 | -8.4965 | 2.53E-16 | 1.02E-14 | 26.2377 |
| AC108463.1 | 0.63334 | 0.908222 | 8.48979 | 2.66E-16 | 1.07E-14 | 26.1881 |
| LIMA1 | 0.69005 | 5.786333 | 8.47682 | 2.93E-16 | 1.17E-14 | 26.0928 |
| CBX2 | 0.67897 | 2.441722 | 8.46627 | 3.17E-16 | 1.26E-14 | 26.0154 |
| MXRA5 | 0.83886 | 1.475305 | 8.46271 | 3.25E-16 | 1.29E-14 | 25.9893 |
| ITPRIPL1 | 0.70452 | 1.718131 | 8.43451 | 4.01E-16 | 1.58E-14 | 25.7829 |
| MICB | 0.56874 | 1.593246 | 8.42689 | 4.24E-16 | 1.66E-14 | 25.7272 |
| PPP1R14BP3 | 0.54982 | 5.753174 | 8.4099 | 4.82E-16 | 1.87E-14 | 25.6031 |
| MMP25 | 0.57432 | 1.637032 | 8.40979 | 4.82E-16 | 1.87E-14 | 25.6023 |
| NRG3 | -0.7108 | 3.805285 | -8.4034 | 5.05E-16 | 1.95E-14 | 25.5555 |
| PTGDS | -0.9887 | 9.226872 | -8.3945 | 5.40E-16 | 2.08E-14 | 25.4906 |
| DDX11 | 0.62764 | 2.797071 | 8.39134 | 5.53E-16 | 2.12E-14 | 25.4678 |
| CYS1 | -0.6519 | 2.886283 | -8.3908 | 5.55E-16 | 2.13E-14 | 25.4641 |
| GRIN2C | -0.7342 | 3.088455 | -8.3899 | 5.59E-16 | 2.14E-14 | 25.457 |
| PLAU | 1.00882 | 2.708551 | 8.38591 | 5.75E-16 | 2.20E-14 | 25.4282 |
| IGF2BP3 | 0.641 | 0.474906 | 8.38472 | 5.80E-16 | 2.22E-14 | 25.4196 |
| PTP4A3 | 0.56838 | 4.964352 | 8.37291 | 6.33E-16 | 2.41E-14 | 25.3336 |
| MT-ND6 | -0.5736 | 13.43817 | -8.3577 | 7.09E-16 | 2.67E-14 | 25.2232 |
| CETP | 0.63363 | 1.893449 | 8.34992 | 7.51E-16 | 2.82E-14 | 25.1666 |
| MFAP2 | 0.74749 | 1.034503 | 8.34307 | 7.90E-16 | 2.96E-14 | 25.1169 |
| H2BC11 | 0.55037 | 1.262854 | 8.33256 | 8.53E-16 | 3.17E-14 | 25.0407 |
| GAL3ST4 | 0.61099 | 4.816872 | 8.32608 | 8.95E-16 | 3.32E-14 | 24.9937 |
| MIR4435-2HG | 0.62939 | 1.195232 | 8.32065 | 9.32E-16 | 3.44E-14 | 24.9545 |
| ADAM12 | 0.73086 | 1.057144 | 8.31836 | 9.47E-16 | 3.49E-14 | 24.9379 |
| FAM20C | 0.6137 | 5.258398 | 8.30928 | 1.01E-15 | 3.71E-14 | 24.8722 |
| AC015967.1 | -0.5128 | 1.773471 | -8.3029 | 1.06E-15 | 3.88E-14 | 24.8257 |
| HLF | -0.6287 | 4.473885 | -8.2906 | 1.16E-15 | 4.23E-14 | 24.7374 |
| EN1 | 0.90636 | 0.787804 | 8.27819 | 1.27E-15 | 4.61E-14 | 24.6476 |
| AL109918.1 | 0.55588 | 3.472043 | 8.27048 | 1.35E-15 | 4.86E-14 | 24.592 |
| SPARCL1 | -0.6594 | 11.42158 | -8.2575 | 1.48E-15 | 5.29E-14 | 24.4982 |
| SLC26A2 | 0.5434 | 2.616757 | 8.25306 | 1.53E-15 | 5.45E-14 | 24.4666 |
| TMEM255A | 0.77305 | 4.90809 | 8.24543 | 1.62E-15 | 5.76E-14 | 24.4118 |
| HOXD9 | 1.0321 | 0.98933 | 8.24323 | 1.64E-15 | 5.84E-14 | 24.3959 |
| TTC26 | 0.53434 | 2.291466 | 8.2361 | 1.73E-15 | 6.14E-14 | 24.3447 |
| FN1 | 0.82348 | 5.719356 | 8.22988 | 1.81E-15 | 6.41E-14 | 24.3 |
| ALDOC | -0.8139 | 9.933016 | -8.2241 | 1.89E-15 | 6.67E-14 | 24.2582 |
| STK17A | 0.5038 | 4.574098 | 8.20432 | 2.18E-15 | 7.65E-14 | 24.1167 |
| AC090692.1 | 0.85971 | 2.427308 | 8.19876 | 2.28E-15 | 7.95E-14 | 24.0769 |
| LINC00836 | -1.0978 | 3.282366 | -8.1988 | 2.28E-15 | 7.95E-14 | 24.0768 |
| ANXA2R | 0.54887 | 2.070714 | 8.19713 | 2.30E-15 | 8.04E-14 | 24.0652 |
| COL6A1 | 0.60325 | 6.556066 | 8.1682 | 2.84E-15 | 9.77E-14 | 23.8584 |
| ZFP36L2 | 0.57859 | 6.850456 | 8.1675 | 2.86E-15 | 9.82E-14 | 23.8534 |
| AL021807.1 | 0.50668 | 1.048309 | 8.16233 | 2.97E-15 | 1.02E-13 | 23.8165 |
| ECSCR | 0.64467 | 2.274969 | 8.15739 | 3.07E-15 | 1.05E-13 | 23.7813 |
| FREM2 | 0.82737 | 1.510933 | 8.14362 | 3.40E-15 | 1.15E-13 | 23.6831 |
| IGFBP4 | 0.75697 | 5.684422 | 8.1292 | 3.77E-15 | 1.27E-13 | 23.5804 |
| CPXM1 | 1.04201 | 5.807333 | 8.11959 | 4.04E-15 | 1.36E-13 | 23.5121 |
| CD151 | 0.54682 | 6.000616 | 8.11815 | 4.09E-15 | 1.37E-13 | 23.5019 |
| ADAM19 | 0.64051 | 2.262554 | 8.11512 | 4.18E-15 | 1.40E-13 | 23.4803 |
| SLC14A1 | -1.4749 | 4.140513 | -8.1131 | 4.24E-15 | 1.42E-13 | 23.466 |
| KLF10 | 0.67294 | 4.277097 | 8.10147 | 4.61E-15 | 1.54E-13 | 23.3835 |
| H2AC8 | 0.6724 | 2.303618 | 8.09827 | 4.72E-15 | 1.57E-13 | 23.3607 |
| STAC3 | 0.50446 | 2.769661 | 8.09313 | 4.90E-15 | 1.62E-13 | 23.3243 |
| PYCR1 | 0.51078 | 5.449813 | 8.09229 | 4.93E-15 | 1.63E-13 | 23.3183 |
| PIM1 | 0.5839 | 4.360932 | 8.09188 | 4.94E-15 | 1.63E-13 | 23.3154 |
| ZNF217 | 0.57251 | 2.865004 | 8.08065 | 5.36E-15 | 1.76E-13 | 23.2359 |
| CALD1 | 0.55657 | 5.136988 | 8.05415 | 6.48E-15 | 2.10E-13 | 23.0484 |
| AL355916.2 | -1.266 | 3.802927 | -8.0426 | 7.04E-15 | 2.27E-13 | 22.967 |
| AC079946.1 | -0.6537 | 1.462182 | -8.0358 | 7.40E-15 | 2.38E-13 | 22.919 |
| HOGA1 | -0.6543 | 2.725472 | -8.0338 | 7.50E-15 | 2.41E-13 | 22.905 |
| LCN12 | -0.5815 | 1.833173 | -8.0242 | 8.04E-15 | 2.57E-13 | 22.837 |
| MRVI1 | -0.6993 | 5.082622 | -8.0024 | 9.40E-15 | 2.98E-13 | 22.6836 |
| FOXD3-AS1 | 1.00623 | 1.730808 | 7.99662 | 9.80E-15 | 3.09E-13 | 22.6431 |
| TRIM14 | 0.65324 | 4.039255 | 7.99529 | 9.89E-15 | 3.12E-13 | 22.6338 |
| H2AC11 | 0.51742 | 0.974113 | 7.98544 | 1.06E-14 | 3.33E-13 | 22.5646 |
| WSCD1 | 0.73718 | 5.606061 | 7.97797 | 1.12E-14 | 3.50E-13 | 22.5122 |
| POU3F2 | 0.53025 | 5.369563 | 7.96905 | 1.19E-14 | 3.71E-13 | 22.4497 |
| COL5A2 | 0.89007 | 3.028377 | 7.96426 | 1.23E-14 | 3.83E-13 | 22.4161 |
| AC010615.1 | 0.52295 | 2.211315 | 7.95761 | 1.29E-14 | 4.00E-13 | 22.3695 |
| MKX | -0.8737 | 2.584784 | -7.957 | 1.30E-14 | 4.02E-13 | 22.3651 |
| PLAT | 0.93055 | 3.124572 | 7.94546 | 1.41E-14 | 4.34E-13 | 22.2846 |
| AL033519.1 | -0.9884 | 2.027954 | -7.944 | 1.43E-14 | 4.38E-13 | 22.274 |
| CYP27B1 | 0.61939 | 0.842117 | 7.94393 | 1.43E-14 | 4.38E-13 | 22.2739 |
| ENG | 0.52443 | 5.457214 | 7.94265 | 1.44E-14 | 4.41E-13 | 22.2649 |
| LINC01831 | 0.55533 | 0.761077 | 7.93826 | 1.49E-14 | 4.54E-13 | 22.2342 |
| MYL3 | -0.6387 | 2.710543 | -7.9363 | 1.51E-14 | 4.60E-13 | 22.2205 |
| HELZ2 | 0.61321 | 2.831392 | 7.93013 | 1.57E-14 | 4.79E-13 | 22.1775 |
| CASP4 | 0.55128 | 1.777904 | 7.91997 | 1.69E-14 | 5.13E-13 | 22.1066 |
| CXCL9 | 0.75966 | 0.962416 | 7.91888 | 1.71E-14 | 5.16E-13 | 22.0989 |
| CLIC1 | 0.77603 | 5.867621 | 7.91822 | 1.71E-14 | 5.18E-13 | 22.0944 |
| ZNF528-AS1 | 0.52399 | 3.881494 | 7.91032 | 1.81E-14 | 5.45E-13 | 22.0393 |
| HPCAL4 | -1.1335 | 5.135069 | -7.9053 | 1.88E-14 | 5.63E-13 | 22.0046 |
| EEF1AKMT3 | 0.62657 | 3.064262 | 7.90247 | 1.92E-14 | 5.74E-13 | 21.9846 |
| F2R | 0.74617 | 5.422002 | 7.90114 | 1.93E-14 | 5.79E-13 | 21.9754 |
| MIR3153 | 0.54422 | 0.658353 | 7.8897 | 2.10E-14 | 6.24E-13 | 21.8957 |
| TGIF1 | 0.66517 | 2.741515 | 7.88135 | 2.23E-14 | 6.60E-13 | 21.8377 |
| AC080112.2 | 0.51842 | 1.638047 | 7.87733 | 2.29E-14 | 6.77E-13 | 21.8098 |
| CRLF1 | -1.3203 | 4.353198 | -7.8767 | 2.30E-14 | 6.80E-13 | 21.8056 |
| SEC61G | 0.80056 | 5.845392 | 7.87158 | 2.38E-14 | 7.03E-13 | 21.7699 |
| TNFRSF11B | 0.79554 | 1.25776 | 7.86897 | 2.43E-14 | 7.15E-13 | 21.7517 |
| RNU6-529P | -0.7539 | 5.861358 | -7.8586 | 2.61E-14 | 7.65E-13 | 21.6796 |
| HOXC4 | 0.78587 | 1.381108 | 7.85452 | 2.69E-14 | 7.86E-13 | 21.6515 |
| KIAA1671 | -0.516 | 3.477643 | -7.8535 | 2.71E-14 | 7.91E-13 | 21.6442 |
| CYP46A1 | -0.5887 | 3.410572 | -7.8534 | 2.71E-14 | 7.91E-13 | 21.6438 |
| MSR1 | 0.8518 | 2.524604 | 7.84794 | 2.82E-14 | 8.19E-13 | 21.606 |
| SYNE2 | 0.53534 | 3.331708 | 7.83808 | 3.02E-14 | 8.74E-13 | 21.5377 |
| MEX3A | 0.74487 | 4.558144 | 7.83743 | 3.03E-14 | 8.76E-13 | 21.5332 |
| SOGA1 | 0.50332 | 5.376322 | 7.83561 | 3.07E-14 | 8.86E-13 | 21.5206 |
| AC068535.1 | 0.66165 | 3.740302 | 7.82501 | 3.31E-14 | 9.51E-13 | 21.4473 |
| LCNL1 | -1.0098 | 3.173715 | -7.8236 | 3.35E-14 | 9.60E-13 | 21.4376 |
| FBXO2 | -0.9233 | 5.965213 | -7.823 | 3.36E-14 | 9.63E-13 | 21.4337 |
| HSD3B7 | 0.51304 | 2.615525 | 7.82185 | 3.39E-14 | 9.70E-13 | 21.4255 |
| HAPLN3 | 0.57461 | 2.211201 | 7.81513 | 3.55E-14 | 1.01E-12 | 21.3791 |
| COL5A1 | 0.80215 | 1.499718 | 7.81497 | 3.55E-14 | 1.01E-12 | 21.378 |
| FLNA | 0.69482 | 6.569307 | 7.80988 | 3.68E-14 | 1.05E-12 | 21.3428 |
| SCD | -0.677 | 9.499706 | -7.8069 | 3.76E-14 | 1.06E-12 | 21.3221 |
| GABRD | -1.1339 | 4.798308 | -7.7783 | 4.60E-14 | 1.29E-12 | 21.1255 |
| LAMC3 | 0.58374 | 2.35467 | 7.75273 | 5.50E-14 | 1.52E-12 | 20.9497 |
| KSR2 | -0.7019 | 1.811504 | -7.7509 | 5.57E-14 | 1.54E-12 | 20.937 |
| COL6A2 | 0.96614 | 3.579202 | 7.7476 | 5.70E-14 | 1.58E-12 | 20.9145 |
| LINC01778 | 0.51338 | 1.999462 | 7.74225 | 5.92E-14 | 1.64E-12 | 20.8778 |
| PLSCR1 | 0.71107 | 3.731421 | 7.73481 | 6.23E-14 | 1.72E-12 | 20.8268 |
| EHD4 | 0.54666 | 3.238804 | 7.72531 | 6.66E-14 | 1.83E-12 | 20.7618 |
| APOBEC3C | 0.71261 | 3.632842 | 7.72148 | 6.84E-14 | 1.88E-12 | 20.7356 |
| CCND1 | 0.78268 | 5.947241 | 7.72095 | 6.87E-14 | 1.88E-12 | 20.732 |
| ILDR2 | 0.59122 | 3.802053 | 7.71117 | 7.35E-14 | 2.01E-12 | 20.6651 |
| SAMD9 | 0.67889 | 2.562879 | 7.7003 | 7.93E-14 | 2.16E-12 | 20.5909 |
| NAP1L2 | -0.7749 | 5.125218 | -7.6996 | 7.97E-14 | 2.17E-12 | 20.5861 |
| IFI44 | 0.70639 | 4.648992 | 7.69363 | 8.31E-14 | 2.25E-12 | 20.5454 |
| ARAP3 | 0.62463 | 2.911708 | 7.68459 | 8.85E-14 | 2.39E-12 | 20.4838 |
| CRNDE | 0.94326 | 1.865574 | 7.68458 | 8.85E-14 | 2.39E-12 | 20.4837 |
| TIMP1 | 1.26578 | 5.13141 | 7.68251 | 8.97E-14 | 2.42E-12 | 20.4696 |
| ADAMTS7 | 0.54939 | 1.157024 | 7.67923 | 9.18E-14 | 2.47E-12 | 20.4472 |
| LIMD1 | 0.60438 | 2.840524 | 7.67894 | 9.20E-14 | 2.47E-12 | 20.4453 |
| AC073046.1 | 0.50744 | 1.352107 | 7.67578 | 9.40E-14 | 2.51E-12 | 20.4238 |
| HMGCLL1 | -0.7097 | 2.056915 | -7.6688 | 9.87E-14 | 2.63E-12 | 20.3764 |
| GPR82 | 0.58693 | 0.811445 | 7.65826 | 1.06E-13 | 2.82E-12 | 20.3046 |
| PPP1R1A | -0.8491 | 3.88481 | -7.6493 | 1.13E-13 | 3.00E-12 | 20.2434 |
| MTURN | -0.6032 | 8.136882 | -7.6366 | 1.23E-13 | 3.25E-12 | 20.1578 |
| NEBL | -0.6158 | 4.949971 | -7.6345 | 1.25E-13 | 3.30E-12 | 20.1432 |
| PLEKHA4 | 0.96479 | 4.240709 | 7.63068 | 1.29E-13 | 3.37E-12 | 20.1175 |
| TEF | -0.5105 | 5.697619 | -7.6167 | 1.42E-13 | 3.70E-12 | 20.0225 |
| ST8SIA4 | 0.52964 | 2.297743 | 7.61557 | 1.43E-13 | 3.72E-12 | 20.0151 |
| LAMB1 | 0.79754 | 3.246525 | 7.60523 | 1.53E-13 | 3.98E-12 | 19.9452 |
| SNAI2 | 0.65454 | 2.020504 | 7.58707 | 1.74E-13 | 4.47E-12 | 19.8226 |
| CYTOR | 0.61446 | 1.235258 | 7.58615 | 1.75E-13 | 4.50E-12 | 19.8164 |
| CACNA2D3 | -0.7896 | 2.592313 | -7.5852 | 1.76E-13 | 4.52E-12 | 19.81 |
| LUM | 0.78921 | 1.896734 | 7.58065 | 1.81E-13 | 4.66E-12 | 19.7793 |
| CDH24 | 0.50621 | 3.980309 | 7.58054 | 1.82E-13 | 4.66E-12 | 19.7786 |
| ADARB2 | -0.8326 | 3.123375 | -7.5782 | 1.84E-13 | 4.72E-12 | 19.763 |
| CLIC4 | 0.54748 | 7.524552 | 7.57652 | 1.87E-13 | 4.77E-12 | 19.7515 |
| NR3C2 | -0.527 | 3.527102 | -7.5751 | 1.88E-13 | 4.82E-12 | 19.7422 |
| STEAP3 | 0.9407 | 3.119714 | 7.57023 | 1.95E-13 | 4.97E-12 | 19.7091 |
| FAM107A | -0.7289 | 9.1898 | -7.5673 | 1.99E-13 | 5.06E-12 | 19.6895 |
| BCL3 | 0.51141 | 2.768499 | 7.5647 | 2.02E-13 | 5.15E-12 | 19.6718 |
| TRIM47 | 0.6005 | 6.453793 | 7.56332 | 2.04E-13 | 5.19E-12 | 19.6625 |
| TRAF4 | 0.5759 | 5.539872 | 7.55761 | 2.13E-13 | 5.38E-12 | 19.6242 |
| MARCHF9 | 0.56884 | 4.531879 | 7.55419 | 2.18E-13 | 5.50E-12 | 19.6011 |
| GPX8 | 0.71447 | 1.115361 | 7.53815 | 2.43E-13 | 6.08E-12 | 19.4934 |
| MCF2L2 | -0.5117 | 2.042775 | -7.5381 | 2.43E-13 | 6.08E-12 | 19.4929 |
| SLC25A48 | -0.9577 | 3.621057 | -7.5228 | 2.70E-13 | 6.70E-12 | 19.3908 |
| SPRY1 | 0.72618 | 3.086609 | 7.52192 | 2.71E-13 | 6.74E-12 | 19.3846 |
| STAT1 | 0.62501 | 5.881738 | 7.49404 | 3.28E-13 | 8.07E-12 | 19.198 |
| MMP14 | 0.83973 | 5.13582 | 7.49261 | 3.32E-13 | 8.13E-12 | 19.1885 |
| ERFE | 0.55769 | 1.416518 | 7.49196 | 3.33E-13 | 8.17E-12 | 19.1841 |
| ADGRE5 | 0.65016 | 3.356041 | 7.48083 | 3.59E-13 | 8.75E-12 | 19.1098 |
| AC064875.1 | 0.72364 | 0.839569 | 7.47948 | 3.63E-13 | 8.82E-12 | 19.1008 |
| FAIM2 | -0.6167 | 7.615607 | -7.4754 | 3.73E-13 | 9.04E-12 | 19.0733 |
| INAVA | 0.61883 | 2.31993 | 7.47495 | 3.74E-13 | 9.06E-12 | 19.0706 |
| TLCD4 | -0.6293 | 2.665281 | -7.4738 | 3.77E-13 | 9.13E-12 | 19.0628 |
| CALN1 | -1.0674 | 3.665769 | -7.468 | 3.92E-13 | 9.45E-12 | 19.0244 |
| SNCG | -1.0578 | 5.251109 | -7.4675 | 3.93E-13 | 9.48E-12 | 19.0209 |
| EMILIN1 | 0.62753 | 4.410591 | 7.46392 | 4.03E-13 | 9.69E-12 | 18.9971 |
| HOXA7 | 0.69057 | 0.491356 | 7.45906 | 4.17E-13 | 9.99E-12 | 18.9647 |
| BRINP1 | -0.9268 | 5.027764 | -7.4483 | 4.48E-13 | 1.07E-11 | 18.893 |
| PPP1R3B | 0.51597 | 2.617406 | 7.44709 | 4.52E-13 | 1.08E-11 | 18.8851 |
| COL18A1 | 0.56972 | 4.224764 | 7.44525 | 4.58E-13 | 1.09E-11 | 18.8729 |
| ZC3HAV1L | 0.5525 | 1.315215 | 7.44299 | 4.65E-13 | 1.10E-11 | 18.8579 |
| RIDA | -0.5683 | 6.788804 | -7.4429 | 4.65E-13 | 1.10E-11 | 18.8573 |
| NAPSB | 1.14724 | 3.712827 | 7.44094 | 4.71E-13 | 1.12E-11 | 18.8442 |
| TUBB6 | 0.77689 | 3.666378 | 7.43832 | 4.80E-13 | 1.14E-11 | 18.8268 |
| RXRG | -0.6814 | 3.010892 | -7.4381 | 4.80E-13 | 1.14E-11 | 18.8257 |
| POGLUT3 | 0.63057 | 3.42497 | 7.4281 | 5.14E-13 | 1.21E-11 | 18.759 |
| LINC00844 | -0.8047 | 7.730063 | -7.4272 | 5.17E-13 | 1.22E-11 | 18.7531 |
| SUSD1 | 0.50791 | 3.255505 | 7.42645 | 5.20E-13 | 1.22E-11 | 18.7481 |
| ATP6V1G2 | -0.6787 | 7.211446 | -7.4247 | 5.26E-13 | 1.24E-11 | 18.7366 |
| SLC25A18 | -0.6632 | 5.959319 | -7.4246 | 5.26E-13 | 1.24E-11 | 18.7357 |
| TSPYL2 | -0.6026 | 6.234965 | -7.4169 | 5.54E-13 | 1.30E-11 | 18.6851 |
| HOXA5 | 0.93915 | 0.731893 | 7.41509 | 5.61E-13 | 1.31E-11 | 18.6727 |
| AFAP1L1 | 0.58686 | 2.667329 | 7.41135 | 5.76E-13 | 1.34E-11 | 18.648 |
| OAS3 | 0.77896 | 3.531148 | 7.40762 | 5.90E-13 | 1.38E-11 | 18.6232 |
| ITGA5 | 0.64546 | 3.060782 | 7.40353 | 6.07E-13 | 1.41E-11 | 18.5962 |
| MCUB | 0.72165 | 2.484559 | 7.39479 | 6.44E-13 | 1.49E-11 | 18.5384 |
| PARP9 | 0.5871 | 3.725618 | 7.39127 | 6.59E-13 | 1.53E-11 | 18.5151 |
| WNT5A | 0.67739 | 3.260585 | 7.39123 | 6.59E-13 | 1.53E-11 | 18.5149 |
| AC010273.3 | 0.56908 | 1.24784 | 7.38069 | 7.08E-13 | 1.63E-11 | 18.4453 |
| H3P6 | 0.50954 | 3.958916 | 7.38069 | 7.08E-13 | 1.63E-11 | 18.4452 |
| IL17D | -0.5921 | 6.23067 | -7.3794 | 7.14E-13 | 1.64E-11 | 18.4367 |
| TMEM71 | 0.55958 | 1.015045 | 7.37733 | 7.24E-13 | 1.66E-11 | 18.4231 |
| PYGL | 0.68658 | 3.859163 | 7.37056 | 7.58E-13 | 1.73E-11 | 18.3784 |
| GDF10 | -1.0318 | 2.906191 | -7.3679 | 7.72E-13 | 1.76E-11 | 18.3606 |
| SPX | -1.0094 | 3.860313 | -7.3603 | 8.12E-13 | 1.85E-11 | 18.3109 |
| PPP1R1B | -0.9598 | 5.125547 | -7.3508 | 8.66E-13 | 1.96E-11 | 18.2483 |
| AC112777.1 | 0.54388 | 1.774918 | 7.34622 | 8.93E-13 | 2.02E-11 | 18.2181 |
| YBX3 | 0.54839 | 3.900431 | 7.33485 | 9.64E-13 | 2.17E-11 | 18.1434 |
| CHST1 | -0.7769 | 5.465782 | -7.3287 | 1.00E-12 | 2.26E-11 | 18.1031 |
| HOXA6 | 0.58722 | 0.370708 | 7.32036 | 1.06E-12 | 2.37E-11 | 18.0483 |
| TAGLN2 | 0.73802 | 6.341035 | 7.31766 | 1.08E-12 | 2.42E-11 | 18.0306 |
| CREB5 | 0.58492 | 3.569243 | 7.3146 | 1.10E-12 | 2.46E-11 | 18.0106 |
| HTR2A | -0.7743 | 2.301878 | -7.3137 | 1.11E-12 | 2.47E-11 | 18.0045 |
| SLC27A3 | 0.53886 | 2.903903 | 7.31297 | 1.12E-12 | 2.48E-11 | 17.9998 |
| GPC2 | 0.64157 | 3.881886 | 7.30825 | 1.15E-12 | 2.56E-11 | 17.9689 |
| TNFRSF12A | 1.04417 | 3.715604 | 7.30089 | 1.21E-12 | 2.68E-11 | 17.9207 |
| TRAM2 | 0.5137 | 3.112836 | 7.29868 | 1.23E-12 | 2.71E-11 | 17.9063 |
| SFRP2 | -1.6508 | 5.963052 | -7.2964 | 1.25E-12 | 2.75E-11 | 17.8913 |
| HMGN2P5 | 0.51293 | 2.462366 | 7.28802 | 1.32E-12 | 2.90E-11 | 17.8366 |
| CLEC18B | 0.55621 | 1.206936 | 7.28783 | 1.32E-12 | 2.90E-11 | 17.8353 |
| APOL4 | 0.97996 | 2.255877 | 7.28628 | 1.33E-12 | 2.93E-11 | 17.8252 |
| LOXL2 | 0.73332 | 2.852381 | 7.28534 | 1.34E-12 | 2.95E-11 | 17.819 |
| KBTBD11 | -0.611 | 5.563792 | -7.2754 | 1.43E-12 | 3.14E-11 | 17.7544 |
| RND3 | 0.68525 | 4.229833 | 7.2729 | 1.46E-12 | 3.18E-11 | 17.7378 |
| LXN | 0.54332 | 2.397856 | 7.27236 | 1.46E-12 | 3.19E-11 | 17.7343 |
| NRP1 | 0.50246 | 3.221988 | 7.26947 | 1.49E-12 | 3.25E-11 | 17.7154 |
| TYMP | 0.89419 | 3.084205 | 7.26864 | 1.50E-12 | 3.26E-11 | 17.71 |
| HOXD10 | 0.71836 | 0.54187 | 7.25901 | 1.60E-12 | 3.47E-11 | 17.6473 |
| SLC43A3 | 0.71359 | 2.486289 | 7.24973 | 1.70E-12 | 3.67E-11 | 17.5869 |
| MMP15 | 0.52464 | 4.725494 | 7.24627 | 1.74E-12 | 3.75E-11 | 17.5644 |
| RUNX1 | 0.64312 | 1.738724 | 7.24132 | 1.80E-12 | 3.87E-11 | 17.5321 |
| NES | 0.74685 | 8.163706 | 7.23931 | 1.82E-12 | 3.92E-11 | 17.5191 |
| AC141557.1 | 0.93271 | 2.281883 | 7.23873 | 1.83E-12 | 3.94E-11 | 17.5153 |
| SGSM1 | -0.6391 | 3.123457 | -7.2376 | 1.84E-12 | 3.96E-11 | 17.5077 |
| TEAD2 | 0.59403 | 3.015714 | 7.22653 | 1.98E-12 | 4.25E-11 | 17.4361 |
| GNG5 | 0.60992 | 6.620684 | 7.21652 | 2.12E-12 | 4.52E-11 | 17.3712 |
| FOXD3 | 0.65714 | 0.896292 | 7.20895 | 2.23E-12 | 4.74E-11 | 17.3221 |
| MS4A6A | 0.89695 | 3.395942 | 7.20281 | 2.32E-12 | 4.92E-11 | 17.2823 |
| KDELR3 | 0.51794 | 1.531569 | 7.19918 | 2.38E-12 | 5.04E-11 | 17.2588 |
| PTPRZ1 | 0.6801 | 9.027271 | 7.19582 | 2.43E-12 | 5.14E-11 | 17.2371 |
| ANGPT2 | 0.77702 | 2.281965 | 7.19257 | 2.48E-12 | 5.23E-11 | 17.2161 |
| PABPC1L | 0.69755 | 2.85166 | 7.176 | 2.77E-12 | 5.81E-11 | 17.109 |
| NMI | 0.53736 | 2.833636 | 7.17317 | 2.82E-12 | 5.91E-11 | 17.0908 |
| USH1C | -1.1538 | 4.217872 | -7.171 | 2.87E-12 | 6.00E-11 | 17.0765 |
| AL031710.1 | -0.5575 | 1.037351 | -7.1689 | 2.91E-12 | 6.08E-11 | 17.0631 |
| AC025171.5 | 0.50909 | 0.913419 | 7.16218 | 3.04E-12 | 6.33E-11 | 17.0199 |
| AC008760.2 | 0.65659 | 1.174504 | 7.15816 | 3.12E-12 | 6.49E-11 | 16.994 |
| RUNX3 | 0.50377 | 1.505358 | 7.15698 | 3.14E-12 | 6.54E-11 | 16.9864 |
| AEN | 0.57629 | 3.465881 | 7.15558 | 3.17E-12 | 6.59E-11 | 16.9773 |
| AC141557.2 | 0.57203 | 1.044639 | 7.15552 | 3.17E-12 | 6.59E-11 | 16.977 |
| VN1R81P | 0.50572 | 3.022123 | 7.15517 | 3.18E-12 | 6.60E-11 | 16.9747 |
| AL391834.1 | -0.5904 | 3.501262 | -7.1548 | 3.19E-12 | 6.62E-11 | 16.9721 |
| NIBAN1 | 0.70465 | 2.36081 | 7.15353 | 3.21E-12 | 6.67E-11 | 16.9642 |
| NALCN | -0.5029 | 4.288143 | -7.1375 | 3.57E-12 | 7.36E-11 | 16.8613 |
| CXCL10 | 1.08183 | 1.8355 | 7.13195 | 3.70E-12 | 7.62E-11 | 16.8254 |
| ARC | 0.98537 | 5.013251 | 7.12476 | 3.88E-12 | 7.95E-11 | 16.7793 |
| FCGBP | 1.1529 | 3.633422 | 7.11316 | 4.19E-12 | 8.55E-11 | 16.7048 |
| OLFML3 | 0.67819 | 5.125341 | 7.1036 | 4.46E-12 | 9.07E-11 | 16.6436 |
| PHYHD1 | -0.6343 | 4.589803 | -7.0827 | 5.11E-12 | 1.03E-10 | 16.5102 |
| TRIO | 0.50385 | 4.858342 | 7.07284 | 5.45E-12 | 1.09E-10 | 16.4471 |
| FSTL5 | -0.9005 | 2.63452 | -7.0727 | 5.46E-12 | 1.09E-10 | 16.4461 |
| NFAM1 | 0.54702 | 2.416641 | 7.06583 | 5.71E-12 | 1.14E-10 | 16.4024 |
| PLIN1 | -0.6993 | 2.165438 | -7.0613 | 5.88E-12 | 1.17E-10 | 16.3737 |
| GPR65 | 0.63472 | 1.439722 | 7.05877 | 5.98E-12 | 1.19E-10 | 16.3574 |
| ADGRE1 | 0.53447 | 0.71276 | 7.05251 | 6.23E-12 | 1.23E-10 | 16.3175 |
| AP001972.5 | -0.7182 | 5.443593 | -7.0386 | 6.82E-12 | 1.34E-10 | 16.2289 |
| INSM1 | 0.80268 | 3.402476 | 7.03198 | 7.11E-12 | 1.40E-10 | 16.187 |
| LINC02716 | -0.5897 | 2.848597 | -7.0173 | 7.83E-12 | 1.53E-10 | 16.0939 |
| LOX | 0.5758 | 1.522183 | 7.01576 | 7.90E-12 | 1.54E-10 | 16.0841 |
| HOXD11 | 0.52282 | 0.316366 | 7.01377 | 8.01E-12 | 1.56E-10 | 16.0715 |
| NLGN4X | 0.527 | 4.553903 | 7.01031 | 8.19E-12 | 1.59E-10 | 16.0495 |
| ACBD7 | -0.9195 | 4.429142 | -7.0064 | 8.40E-12 | 1.63E-10 | 16.0246 |
| LSP1 | 0.72129 | 2.157995 | 7.00577 | 8.43E-12 | 1.63E-10 | 16.0208 |
| AC010332.1 | 0.52689 | 4.634958 | 7.00047 | 8.73E-12 | 1.68E-10 | 15.9873 |
| AC074135.1 | 0.5779 | 1.542615 | 6.99846 | 8.84E-12 | 1.70E-10 | 15.9745 |
| VAV3 | 0.73075 | 1.197098 | 6.99567 | 9.00E-12 | 1.73E-10 | 15.9569 |
| LINC01088 | -1.1287 | 4.420159 | -6.9932 | 9.15E-12 | 1.76E-10 | 15.9411 |
| HS3ST4 | -0.778 | 2.433887 | -6.9898 | 9.35E-12 | 1.79E-10 | 15.9199 |
| OAS2 | 0.72286 | 2.845675 | 6.97149 | 1.05E-11 | 2.00E-10 | 15.8042 |
| SCIMP | 0.50596 | 1.696929 | 6.96773 | 1.08E-11 | 2.05E-10 | 15.7806 |
| NID2 | 0.57733 | 1.861898 | 6.94592 | 1.24E-11 | 2.32E-10 | 15.6432 |
| CSDC2 | -0.9615 | 5.90945 | -6.9451 | 1.25E-11 | 2.34E-10 | 15.6378 |
| LMO1 | 0.75435 | 2.856148 | 6.92192 | 1.45E-11 | 2.69E-10 | 15.4926 |
| HAS2 | 0.72598 | 2.453512 | 6.91909 | 1.47E-11 | 2.74E-10 | 15.4748 |
| WNK2 | -0.7342 | 3.381495 | -6.9112 | 1.55E-11 | 2.87E-10 | 15.4255 |
| LIF | 0.72115 | 1.160317 | 6.91094 | 1.55E-11 | 2.87E-10 | 15.4238 |
| ADGRL4 | 0.51623 | 3.483648 | 6.90776 | 1.59E-11 | 2.93E-10 | 15.4039 |
| CA3 | 0.87826 | 1.57169 | 6.90599 | 1.60E-11 | 2.96E-10 | 15.3928 |
| NPNT | 0.93643 | 2.616432 | 6.90362 | 1.63E-11 | 3.00E-10 | 15.378 |
| SECTM1 | 0.67269 | 1.840872 | 6.90339 | 1.63E-11 | 3.00E-10 | 15.3766 |
| B2M | 0.51799 | 9.68731 | 6.89973 | 1.67E-11 | 3.07E-10 | 15.3537 |
| ETS1 | 0.58485 | 4.849539 | 6.89374 | 1.73E-11 | 3.18E-10 | 15.3162 |
| HSPB8 | -0.757 | 7.732305 | -6.8931 | 1.74E-11 | 3.19E-10 | 15.3125 |
| TGFBI | 0.89864 | 3.423529 | 6.89216 | 1.75E-11 | 3.20E-10 | 15.3064 |
| MYD88 | 0.58957 | 3.949349 | 6.8896 | 1.78E-11 | 3.25E-10 | 15.2904 |
| PTCRA | 0.51098 | 0.836596 | 6.88215 | 1.87E-11 | 3.40E-10 | 15.2439 |
| KCNB1 | -0.584 | 4.455521 | -6.877 | 1.93E-11 | 3.51E-10 | 15.2116 |
| FUT9 | -0.5653 | 3.941817 | -6.8763 | 1.94E-11 | 3.52E-10 | 15.2072 |
| CTSC | 0.59387 | 3.079417 | 6.85955 | 2.16E-11 | 3.89E-10 | 15.103 |
| CNN3 | 0.52399 | 8.597016 | 6.85709 | 2.19E-11 | 3.94E-10 | 15.0878 |
| MCAM | 0.56876 | 4.555218 | 6.85298 | 2.25E-11 | 4.04E-10 | 15.0622 |
| TAP1 | 0.52989 | 5.311425 | 6.85061 | 2.28E-11 | 4.10E-10 | 15.0474 |
| IRX5 | 0.54299 | 0.731293 | 6.84047 | 2.44E-11 | 4.36E-10 | 14.9844 |
| HOXD-AS2 | 0.62782 | 1.004341 | 6.83212 | 2.57E-11 | 4.59E-10 | 14.9326 |
| HOXA10 | 0.75548 | 0.70459 | 6.82033 | 2.77E-11 | 4.92E-10 | 14.8596 |
| HAND2 | 0.56638 | 0.571387 | 6.81163 | 2.92E-11 | 5.19E-10 | 14.8057 |
| MAP1LC3C | 0.68251 | 0.843333 | 6.81018 | 2.95E-11 | 5.23E-10 | 14.7968 |
| LYNX1 | -0.5584 | 5.589141 | -6.8091 | 2.97E-11 | 5.26E-10 | 14.7903 |
| TUBA4A | -0.8505 | 4.600422 | -6.8083 | 2.99E-11 | 5.29E-10 | 14.785 |
| HOXD13 | 0.64661 | 0.4016 | 6.80596 | 3.03E-11 | 5.36E-10 | 14.7706 |
| AC002456.1 | 0.54944 | 2.512223 | 6.80508 | 3.05E-11 | 5.38E-10 | 14.7652 |
| EGFR | 0.94346 | 6.499868 | 6.80343 | 3.08E-11 | 5.43E-10 | 14.755 |
| GRIK3 | 0.63731 | 5.381561 | 6.7921 | 3.31E-11 | 5.81E-10 | 14.685 |
| EPSTI1 | 0.58575 | 1.991575 | 6.79193 | 3.31E-11 | 5.81E-10 | 14.684 |
| SPSB4 | 0.61983 | 3.324001 | 6.78066 | 3.56E-11 | 6.22E-10 | 14.6144 |
| ANXA1 | 1.12264 | 4.41343 | 6.77707 | 3.64E-11 | 6.36E-10 | 14.5924 |
| RTN1 | -0.6626 | 7.330221 | -6.7632 | 3.97E-11 | 6.88E-10 | 14.5072 |
| SERPINI1 | -0.8766 | 5.383357 | -6.7628 | 3.98E-11 | 6.90E-10 | 14.5045 |
| PLEKHB1 | -0.5212 | 8.52929 | -6.7619 | 4.00E-11 | 6.93E-10 | 14.499 |
| SCN2B | -0.759 | 3.805081 | -6.7605 | 4.04E-11 | 6.99E-10 | 14.4901 |
| PDPN | 1.16543 | 3.571739 | 6.75739 | 4.12E-11 | 7.11E-10 | 14.4712 |
| MGP | 1.07719 | 4.239912 | 6.75608 | 4.15E-11 | 7.17E-10 | 14.4632 |
| PNMA8B | -0.5583 | 4.066198 | -6.7558 | 4.16E-11 | 7.17E-10 | 14.4617 |
| PNCK | -0.6272 | 3.199931 | -6.754 | 4.21E-11 | 7.25E-10 | 14.4506 |
| PLBD1 | 0.55252 | 1.901187 | 6.75383 | 4.21E-11 | 7.26E-10 | 14.4494 |
| CHI3L2 | 1.20425 | 3.834745 | 6.7345 | 4.76E-11 | 8.14E-10 | 14.3308 |
| FPR3 | 0.7749 | 2.163526 | 6.73259 | 4.81E-11 | 8.22E-10 | 14.3191 |
| CELSR1 | 0.54469 | 0.966652 | 6.72682 | 4.99E-11 | 8.51E-10 | 14.2837 |
| RNU6-850P | 0.62016 | 2.538931 | 6.72627 | 5.01E-11 | 8.53E-10 | 14.2803 |
| PDLIM1 | 0.75125 | 2.975107 | 6.72382 | 5.09E-11 | 8.66E-10 | 14.2653 |
| GNLY | 0.5855 | 1.273646 | 6.70273 | 5.80E-11 | 9.79E-10 | 14.1364 |
| IGF2BP2 | 0.70885 | 1.000981 | 6.70036 | 5.89E-11 | 9.92E-10 | 14.122 |
| GBP1P1 | 0.60035 | 1.329102 | 6.69795 | 5.98E-11 | 1.00E-09 | 14.1072 |
| KIAA0040 | 0.72263 | 2.917751 | 6.69773 | 5.99E-11 | 1.01E-09 | 14.1059 |
| HOTAIRM1 | 0.82055 | 1.468628 | 6.69472 | 6.10E-11 | 1.02E-09 | 14.0875 |
| PLAUR | 0.58354 | 2.171583 | 6.69426 | 6.12E-11 | 1.03E-09 | 14.0847 |
| TEAD4 | 0.67516 | 2.203098 | 6.6919 | 6.21E-11 | 1.04E-09 | 14.0704 |
| ADCY2 | -0.5335 | 5.057411 | -6.6835 | 6.54E-11 | 1.09E-09 | 14.0192 |
| LINC01956 | 0.56256 | 0.782824 | 6.68294 | 6.57E-11 | 1.09E-09 | 14.0157 |
| SOCS1 | 0.53587 | 1.659984 | 6.67989 | 6.69E-11 | 1.11E-09 | 13.9972 |
| ANXA2P2 | 0.56577 | 1.262584 | 6.6753 | 6.89E-11 | 1.14E-09 | 13.9692 |
| CARD16 | 0.59844 | 1.865567 | 6.67504 | 6.90E-11 | 1.14E-09 | 13.9676 |
| FST | 0.51573 | 1.506319 | 6.67326 | 6.98E-11 | 1.15E-09 | 13.9568 |
| HLA-A | 0.5271 | 8.894674 | 6.6728 | 7.00E-11 | 1.16E-09 | 13.954 |
| PNMA6F | -0.7454 | 1.243802 | -6.6633 | 7.42E-11 | 1.22E-09 | 13.896 |
| LINC01579 | 0.78666 | 0.97884 | 6.65848 | 7.65E-11 | 1.26E-09 | 13.867 |
| PRLHR | -1.1166 | 2.761247 | -6.6552 | 7.81E-11 | 1.28E-09 | 13.8468 |
| ENHO | -0.6463 | 8.558421 | -6.6435 | 8.39E-11 | 1.37E-09 | 13.7763 |
| SLCO1A2 | -0.672 | 4.11219 | -6.6406 | 8.55E-11 | 1.39E-09 | 13.7582 |
| NKG7 | 0.55633 | 1.769232 | 6.63041 | 9.11E-11 | 1.48E-09 | 13.6968 |
| S100A11 | 0.74535 | 6.872417 | 6.63026 | 9.11E-11 | 1.48E-09 | 13.696 |
| TCIM | 0.83633 | 4.065146 | 6.60622 | 1.06E-10 | 1.70E-09 | 13.5507 |
| SNTA1 | -0.6383 | 6.62741 | -6.5991 | 1.11E-10 | 1.77E-09 | 13.5078 |
| EMX2OS | -0.7109 | 2.934297 | -6.5869 | 1.19E-10 | 1.89E-09 | 13.434 |
| LINC01532 | -0.8548 | 2.896534 | -6.5849 | 1.21E-10 | 1.92E-09 | 13.422 |
| HIF3A | -0.8245 | 3.367652 | -6.5729 | 1.30E-10 | 2.05E-09 | 13.35 |
| IER5L | 0.55132 | 3.107404 | 6.56735 | 1.34E-10 | 2.12E-09 | 13.3168 |
| PAQR8 | -0.568 | 6.390851 | -6.5643 | 1.37E-10 | 2.16E-09 | 13.2987 |
| KCNN1 | -0.57 | 3.713385 | -6.541 | 1.58E-10 | 2.46E-09 | 13.1589 |
| LRRC17 | 0.71373 | 3.546479 | 6.51611 | 1.84E-10 | 2.84E-09 | 13.0102 |
| AC245297.1 | -0.5119 | 5.426607 | -6.5117 | 1.89E-10 | 2.91E-09 | 12.9839 |
| AKR1C3 | -0.6314 | 4.150417 | -6.5035 | 1.99E-10 | 3.05E-09 | 12.9353 |
| MIR3682 | -0.5246 | 7.593545 | -6.4986 | 2.05E-10 | 3.13E-09 | 12.9059 |
| FZD2 | 0.53163 | 2.537244 | 6.49342 | 2.11E-10 | 3.22E-09 | 12.8751 |
| MT-TP | -0.5067 | 10.96847 | -6.49 | 2.16E-10 | 3.28E-09 | 12.8548 |
| MIR25 | 0.56984 | 3.963056 | 6.48243 | 2.26E-10 | 3.43E-09 | 12.8098 |
| AL023284.4 | -0.644 | 3.537336 | -6.471 | 2.42E-10 | 3.66E-09 | 12.7422 |
| AL354863.1 | -0.5692 | 1.208993 | -6.4676 | 2.47E-10 | 3.73E-09 | 12.7219 |
| COL14A1 | 0.66743 | 1.806846 | 6.46157 | 2.57E-10 | 3.86E-09 | 12.6861 |
| SEPTIN4 | -0.6093 | 5.809015 | -6.4614 | 2.57E-10 | 3.86E-09 | 12.6851 |
| C2 | 0.57417 | 2.14639 | 6.45183 | 2.72E-10 | 4.08E-09 | 12.6284 |
| H2BC5 | 0.57251 | 3.770878 | 6.44909 | 2.77E-10 | 4.14E-09 | 12.6123 |
| CPNE6 | -0.9636 | 2.798883 | -6.4465 | 2.81E-10 | 4.20E-09 | 12.5972 |
| TFPI | 0.70362 | 1.817988 | 6.44166 | 2.90E-10 | 4.32E-09 | 12.5684 |
| TNFAIP6 | 0.72872 | 1.729264 | 6.44115 | 2.91E-10 | 4.33E-09 | 12.5653 |
| IRF7 | 0.56704 | 3.731115 | 6.42752 | 3.16E-10 | 4.67E-09 | 12.4849 |
| STK32B | 0.66633 | 2.232163 | 6.42647 | 3.18E-10 | 4.70E-09 | 12.4788 |
| MMP9 | 0.79324 | 1.099773 | 6.42465 | 3.21E-10 | 4.75E-09 | 12.468 |
| CORO6 | -0.7186 | 2.440833 | -6.4245 | 3.21E-10 | 4.75E-09 | 12.467 |
| RASL10A | -0.8127 | 4.974262 | -6.4225 | 3.25E-10 | 4.80E-09 | 12.4553 |
| AC125616.1 | -0.6042 | 1.247207 | -6.42 | 3.30E-10 | 4.87E-09 | 12.4406 |
| PAPLN | -0.6565 | 3.177962 | -6.4189 | 3.33E-10 | 4.90E-09 | 12.4339 |
| FKBP10 | 0.60288 | 5.804477 | 6.41207 | 3.46E-10 | 5.09E-09 | 12.3939 |
| SLAMF8 | 0.61646 | 1.763949 | 6.41127 | 3.48E-10 | 5.11E-09 | 12.3892 |
| ARHGAP44 | -0.5969 | 2.518052 | -6.4101 | 3.51E-10 | 5.14E-09 | 12.3822 |
| SREBF1 | -0.5068 | 5.639314 | -6.4088 | 3.53E-10 | 5.17E-09 | 12.3749 |
| SERPINA1 | 0.77859 | 3.190844 | 6.4073 | 3.57E-10 | 5.22E-09 | 12.3658 |
| KRT222 | -0.6005 | 1.166459 | -6.3997 | 3.73E-10 | 5.45E-09 | 12.3213 |
| RHOJ | 0.51003 | 3.654066 | 6.38949 | 3.97E-10 | 5.78E-09 | 12.2613 |
| KCNJ3 | -0.7463 | 2.588248 | -6.3747 | 4.34E-10 | 6.28E-09 | 12.1745 |
| MYO1B | 0.50525 | 2.707698 | 6.36822 | 4.51E-10 | 6.52E-09 | 12.1366 |
| ARRB1 | -0.5213 | 4.261804 | -6.3643 | 4.62E-10 | 6.66E-09 | 12.1139 |
| SERINC2 | 0.61102 | 1.985175 | 6.36143 | 4.70E-10 | 6.77E-09 | 12.097 |
| KIF6 | -0.56 | 1.846149 | -6.3568 | 4.83E-10 | 6.94E-09 | 12.0702 |
| PLVAP | 0.61115 | 3.975636 | 6.35371 | 4.92E-10 | 7.06E-09 | 12.0518 |
| NTSR2 | -1.0517 | 5.281338 | -6.3378 | 5.41E-10 | 7.72E-09 | 11.9593 |
| IFI6 | 0.75687 | 8.044144 | 6.32184 | 5.96E-10 | 8.42E-09 | 11.8662 |
| HLA-H | 0.5733 | 5.14868 | 6.31426 | 6.23E-10 | 8.79E-09 | 11.8221 |
| SOX4 | 0.71562 | 6.7989 | 6.3092 | 6.42E-10 | 9.05E-09 | 11.7928 |
| HOXC10 | 0.68253 | 0.49417 | 6.30827 | 6.46E-10 | 9.09E-09 | 11.7873 |
| PANX2 | -0.5788 | 3.257257 | -6.3023 | 6.69E-10 | 9.39E-09 | 11.7529 |
| NKX2-5 | 0.52103 | 0.485536 | 6.30171 | 6.72E-10 | 9.42E-09 | 11.7493 |
| AC002428.2 | -0.6701 | 1.518014 | -6.2923 | 7.10E-10 | 9.91E-09 | 11.6948 |
| MRC2 | 0.65906 | 4.655127 | 6.28933 | 7.23E-10 | 1.01E-08 | 11.6776 |
| C6orf118 | 0.53917 | 1.275695 | 6.28318 | 7.50E-10 | 1.04E-08 | 11.642 |
| MSN | 0.66345 | 6.114863 | 6.28275 | 7.52E-10 | 1.05E-08 | 11.6395 |
| EFHD1 | -0.5537 | 6.963398 | -6.2755 | 7.85E-10 | 1.09E-08 | 11.5975 |
| ANXA2 | 0.7233 | 4.052747 | 6.26599 | 8.30E-10 | 1.15E-08 | 11.5427 |
| ARL4C | 0.70581 | 4.902875 | 6.26389 | 8.41E-10 | 1.16E-08 | 11.5306 |
| LAMA2 | 0.55363 | 2.624251 | 6.25397 | 8.92E-10 | 1.22E-08 | 11.4734 |
| TENT5A | 0.5147 | 2.684452 | 6.24621 | 9.34E-10 | 1.28E-08 | 11.4287 |
| KLHL32 | -0.5291 | 4.469286 | -6.2455 | 9.38E-10 | 1.28E-08 | 11.4245 |
| TNC | 0.80993 | 5.808766 | 6.24468 | 9.42E-10 | 1.29E-08 | 11.4199 |
| RAC2 | 0.56066 | 2.768979 | 6.24264 | 9.54E-10 | 1.30E-08 | 11.4082 |
| SH3GL2 | -0.7737 | 6.436623 | -6.2383 | 9.79E-10 | 1.34E-08 | 11.3829 |
| CA11 | -0.6192 | 6.678273 | -6.2283 | 1.04E-09 | 1.41E-08 | 11.3255 |
| SINHCAF | 0.51705 | 3.407478 | 6.22509 | 1.06E-09 | 1.43E-08 | 11.3073 |
| PAIP2B | -0.523 | 4.109159 | -6.2243 | 1.06E-09 | 1.44E-08 | 11.3026 |
| NETO2 | 0.61272 | 3.364656 | 6.22427 | 1.06E-09 | 1.44E-08 | 11.3026 |
| S100A4 | 0.7953 | 3.284167 | 6.22299 | 1.07E-09 | 1.45E-08 | 11.2952 |
| F5 | -1.023 | 2.544825 | -6.2167 | 1.11E-09 | 1.50E-08 | 11.259 |
| PXDN | 0.61658 | 3.809472 | 6.20612 | 1.18E-09 | 1.59E-08 | 11.1986 |
| CD48 | 0.53248 | 1.136844 | 6.20382 | 1.20E-09 | 1.61E-08 | 11.1854 |
| PPIC | 0.53165 | 3.179155 | 6.1945 | 1.27E-09 | 1.69E-08 | 11.1322 |
| SLC7A7 | 0.51309 | 3.046834 | 6.19318 | 1.28E-09 | 1.70E-08 | 11.1246 |
| HLA-DQA1 | 0.91752 | 2.838389 | 6.18516 | 1.34E-09 | 1.78E-08 | 11.0788 |
| AC084880.1 | -0.6593 | 3.919873 | -6.1816 | 1.37E-09 | 1.81E-08 | 11.0585 |
| SMIM10L2B | -0.6489 | 3.365413 | -6.1774 | 1.40E-09 | 1.85E-08 | 11.0347 |
| FMOD | 0.94635 | 2.349088 | 6.17378 | 1.43E-09 | 1.89E-08 | 11.0139 |
| TWIST1 | 0.54751 | 1.597133 | 6.16857 | 1.47E-09 | 1.94E-08 | 10.9842 |
| GSC | 0.57735 | 1.772439 | 6.16554 | 1.50E-09 | 1.97E-08 | 10.967 |
| SAMD9L | 0.62164 | 3.106961 | 6.16367 | 1.52E-09 | 1.99E-08 | 10.9563 |
| LBH | 0.54835 | 4.270448 | 6.16234 | 1.53E-09 | 2.01E-08 | 10.9488 |
| CCR5 | 0.53538 | 1.403822 | 6.15831 | 1.57E-09 | 2.05E-08 | 10.9258 |
| SERPINE1 | 1.0223 | 3.683263 | 6.15759 | 1.57E-09 | 2.06E-08 | 10.9217 |
| IL9 | -0.5315 | 1.246044 | -6.1542 | 1.60E-09 | 2.09E-08 | 10.9025 |
| KLK7 | -0.6433 | 1.000682 | -6.1535 | 1.61E-09 | 2.10E-08 | 10.8988 |
| AKR1C1 | -0.6053 | 2.247102 | -6.1438 | 1.70E-09 | 2.21E-08 | 10.8435 |
| ESM1 | 0.63808 | 0.787114 | 6.13981 | 1.74E-09 | 2.26E-08 | 10.8208 |
| LYZ | 0.89428 | 3.059093 | 6.13555 | 1.79E-09 | 2.31E-08 | 10.7966 |
| HLA-B | 0.57424 | 8.835342 | 6.13505 | 1.79E-09 | 2.32E-08 | 10.7938 |
| ITGAL | 0.55152 | 2.14138 | 6.13323 | 1.81E-09 | 2.34E-08 | 10.7835 |
| TM4SF1 | 0.64752 | 3.691036 | 6.13313 | 1.81E-09 | 2.34E-08 | 10.7829 |
| HGF | 0.50255 | 1.227988 | 6.13184 | 1.83E-09 | 2.36E-08 | 10.7756 |
| LYVE1 | -0.7861 | 2.937928 | -6.1292 | 1.85E-09 | 2.39E-08 | 10.7608 |
| EMILIN2 | 0.60303 | 1.742383 | 6.11727 | 1.99E-09 | 2.55E-08 | 10.6931 |
| LINC01561 | -0.5351 | 3.138455 | -6.117 | 1.99E-09 | 2.55E-08 | 10.6919 |
| KNDC1 | -0.5883 | 4.217204 | -6.1136 | 2.03E-09 | 2.60E-08 | 10.6722 |
| ENPP5 | -0.6563 | 3.545306 | -6.1096 | 2.08E-09 | 2.65E-08 | 10.6499 |
| DPYD | 0.56405 | 2.458752 | 6.10809 | 2.10E-09 | 2.68E-08 | 10.6413 |
| MACORIS | 0.54428 | 3.503467 | 6.10712 | 2.11E-09 | 2.69E-08 | 10.6358 |
| CTHRC1 | 0.60205 | 1.786824 | 6.10301 | 2.16E-09 | 2.75E-08 | 10.6126 |
| HEPH | -0.6013 | 3.616429 | -6.1023 | 2.17E-09 | 2.76E-08 | 10.6087 |
| RGS16 | 0.7432 | 3.364377 | 6.10219 | 2.17E-09 | 2.76E-08 | 10.608 |
| GDF15 | 0.67126 | 1.280611 | 6.09378 | 2.28E-09 | 2.89E-08 | 10.5605 |
| MAP7 | -0.5499 | 4.488254 | -6.0931 | 2.29E-09 | 2.90E-08 | 10.5567 |
| TSPAN13 | 0.55041 | 5.730404 | 6.0908 | 2.32E-09 | 2.94E-08 | 10.5438 |
| ATP1A2 | -0.7672 | 8.784691 | -6.0891 | 2.34E-09 | 2.96E-08 | 10.5343 |
| LINC02607 | -0.65 | 1.080101 | -6.0849 | 2.40E-09 | 3.03E-08 | 10.5106 |
| MEOX2 | 0.92633 | 1.091397 | 6.08403 | 2.41E-09 | 3.04E-08 | 10.5057 |
| DUSP10 | 0.50522 | 3.005613 | 6.08388 | 2.41E-09 | 3.05E-08 | 10.5048 |
| GGTA1P | -0.6108 | 4.896022 | -6.0826 | 2.43E-09 | 3.06E-08 | 10.4978 |
| ISG15 | 0.77624 | 6.22974 | 6.08224 | 2.44E-09 | 3.07E-08 | 10.4956 |
| PDGFD | 0.66871 | 1.652294 | 6.08008 | 2.47E-09 | 3.10E-08 | 10.4834 |
| RDH10 | 0.56855 | 3.280766 | 6.0779 | 2.50E-09 | 3.14E-08 | 10.4712 |
| CFH | 0.51712 | 2.297503 | 6.07165 | 2.59E-09 | 3.25E-08 | 10.436 |
| HLA-C | 0.51448 | 8.689781 | 6.06982 | 2.62E-09 | 3.28E-08 | 10.4257 |
| BCAT1 | 0.67729 | 2.595445 | 6.06622 | 2.67E-09 | 3.34E-08 | 10.4055 |
| ACSL6 | -0.5652 | 4.118572 | -6.0644 | 2.70E-09 | 3.37E-08 | 10.3953 |
| H19 | 0.77666 | 0.744637 | 6.06216 | 2.74E-09 | 3.41E-08 | 10.3828 |
| CPVL | 0.64202 | 4.229588 | 6.06191 | 2.74E-09 | 3.42E-08 | 10.3814 |
| IDH2-DT | -0.5303 | 2.578677 | -6.0553 | 2.85E-09 | 3.53E-08 | 10.3442 |
| DUSP6 | 0.59024 | 4.314707 | 6.04594 | 3.01E-09 | 3.71E-08 | 10.2918 |
| AC136475.2 | -0.5775 | 4.190594 | -6.0303 | 3.29E-09 | 4.04E-08 | 10.2043 |
| VEGFA | 0.57463 | 3.217954 | 6.02241 | 3.44E-09 | 4.21E-08 | 10.1604 |
| MYOM1 | -0.5099 | 2.772312 | -6.0204 | 3.48E-09 | 4.26E-08 | 10.149 |
| METTL7B | 1.0285 | 4.005341 | 6.01584 | 3.57E-09 | 4.37E-08 | 10.1237 |
| AJAP1 | -0.6226 | 2.417193 | -6.0036 | 3.83E-09 | 4.66E-08 | 10.0559 |
| LY96 | 0.57999 | 3.893165 | 5.99959 | 3.92E-09 | 4.76E-08 | 10.0333 |
| FBN3 | 0.5264 | 1.150821 | 5.98869 | 4.17E-09 | 5.05E-08 | 9.97274 |
| AC022075.1 | 0.51526 | 2.003777 | 5.98631 | 4.23E-09 | 5.11E-08 | 9.95948 |
| NAMPT | 0.53051 | 4.456867 | 5.97583 | 4.49E-09 | 5.40E-08 | 9.90139 |
| AF131216.3 | -0.6427 | 3.516004 | -5.9715 | 4.60E-09 | 5.53E-08 | 9.87746 |
| LINC02308 | 0.52429 | 1.322708 | 5.96828 | 4.69E-09 | 5.62E-08 | 9.85957 |
| AC107398.3 | -0.6593 | 3.879386 | -5.9609 | 4.89E-09 | 5.85E-08 | 9.81889 |
| AJ011932.1 | 0.55725 | 0.921569 | 5.96084 | 4.89E-09 | 5.85E-08 | 9.8184 |
| TBX15 | 0.5549 | 2.216734 | 5.95532 | 5.05E-09 | 6.02E-08 | 9.78789 |
| CIITA | 0.56226 | 1.73813 | 5.95362 | 5.10E-09 | 6.07E-08 | 9.77852 |
| TRIB2 | 0.53507 | 6.158377 | 5.94855 | 5.25E-09 | 6.23E-08 | 9.75052 |
| CD58 | 0.5333 | 2.953826 | 5.94671 | 5.30E-09 | 6.29E-08 | 9.74037 |
| SLC22A6 | -0.6561 | 2.509707 | -5.9414 | 5.46E-09 | 6.47E-08 | 9.71112 |
| PDE1A | -0.6287 | 2.760063 | -5.9397 | 5.52E-09 | 6.52E-08 | 9.70182 |
| DLGAP2 | -0.517 | 1.245175 | -5.9335 | 5.71E-09 | 6.73E-08 | 9.66761 |
| KCNQ5 | -0.6046 | 3.122246 | -5.9305 | 5.81E-09 | 6.83E-08 | 9.65109 |
| SLC1A2 | -0.6977 | 7.68188 | -5.9272 | 5.92E-09 | 6.95E-08 | 9.63269 |
| SOCS2 | 0.59217 | 2.488652 | 5.90549 | 6.70E-09 | 7.78E-08 | 9.51356 |
| AC009533.1 | 0.52006 | 2.056333 | 5.90434 | 6.74E-09 | 7.83E-08 | 9.50724 |
| IL13RA2 | 0.94509 | 1.815502 | 5.90424 | 6.74E-09 | 7.83E-08 | 9.50667 |
| SNORC | -0.7891 | 3.79365 | -5.9042 | 6.75E-09 | 7.83E-08 | 9.50641 |
| INSYN2B | -0.5924 | 2.331886 | -5.8994 | 6.93E-09 | 8.02E-08 | 9.4804 |
| NUP210 | 0.56278 | 3.617707 | 5.89601 | 7.07E-09 | 8.17E-08 | 9.46158 |
| SDS | -0.6237 | 3.323076 | -5.8861 | 7.47E-09 | 8.61E-08 | 9.40711 |
| SELL | -1.1135 | 5.527587 | -5.8718 | 8.10E-09 | 9.28E-08 | 9.32908 |
| PNMA3 | -0.6353 | 3.707093 | -5.8676 | 8.29E-09 | 9.48E-08 | 9.30645 |
| AL391845.2 | -0.5511 | 1.454129 | -5.8643 | 8.45E-09 | 9.64E-08 | 9.28821 |
| HLA-DPA1 | 0.83042 | 5.392753 | 5.86322 | 8.50E-09 | 9.69E-08 | 9.28238 |
| C1QB | 0.6862 | 8.076688 | 5.85962 | 8.67E-09 | 9.87E-08 | 9.26278 |
| FEZF2 | -0.5822 | 2.154702 | -5.8523 | 9.04E-09 | 1.03E-07 | 9.22286 |
| LOXL1 | 0.5599 | 1.391924 | 5.85 | 9.15E-09 | 1.04E-07 | 9.21038 |
| POSTN | 0.92796 | 0.977807 | 5.84852 | 9.23E-09 | 1.05E-07 | 9.20233 |
| CASP1 | 0.53446 | 2.838972 | 5.83731 | 9.83E-09 | 1.11E-07 | 9.14141 |
| UCP2 | 0.59222 | 4.93057 | 5.83232 | 1.01E-08 | 1.14E-07 | 9.11433 |
| BEX2 | -0.5596 | 6.802021 | -5.8261 | 1.05E-08 | 1.17E-07 | 9.08075 |
| STAC | 0.59581 | 0.740868 | 5.82401 | 1.06E-08 | 1.19E-07 | 9.06925 |
| NPY | -0.8969 | 4.576563 | -5.82 | 1.08E-08 | 1.21E-07 | 9.04776 |
| EMX2 | -0.6204 | 3.307766 | -5.8194 | 1.09E-08 | 1.21E-07 | 9.04419 |
| LINC01485 | -0.5474 | 1.535602 | -5.815 | 1.11E-08 | 1.24E-07 | 9.02031 |
| OBI1-AS1 | -0.5691 | 2.202376 | -5.812 | 1.13E-08 | 1.26E-07 | 9.00429 |
| RGS20 | -0.5514 | 2.990318 | -5.7993 | 1.21E-08 | 1.35E-07 | 8.93581 |
| CCER2 | 0.60072 | 2.183336 | 5.78634 | 1.31E-08 | 1.44E-07 | 8.86567 |
| HOXA4 | 0.56435 | 0.453057 | 5.76984 | 1.43E-08 | 1.57E-07 | 8.77686 |
| EN2 | 0.50297 | 1.25664 | 5.76722 | 1.45E-08 | 1.59E-07 | 8.76275 |
| CYTL1 | 0.60889 | 3.571733 | 5.76062 | 1.51E-08 | 1.64E-07 | 8.72731 |
| DMRTA2 | 0.71646 | 0.99097 | 5.75432 | 1.56E-08 | 1.70E-07 | 8.69349 |
| FOXD1 | 0.68155 | 2.71331 | 5.75157 | 1.58E-08 | 1.72E-07 | 8.67878 |
| ABI3 | 0.54867 | 4.015679 | 5.75144 | 1.58E-08 | 1.72E-07 | 8.67805 |
| FYB1 | 0.57846 | 3.448408 | 5.75111 | 1.59E-08 | 1.73E-07 | 8.67631 |
| LINC02587 | 0.80871 | 0.882262 | 5.74917 | 1.60E-08 | 1.74E-07 | 8.66591 |
| NTM-AS1 | -0.6317 | 2.363872 | -5.7318 | 1.77E-08 | 1.91E-07 | 8.57278 |
| HLA-DRB1 | 0.83208 | 7.561012 | 5.73132 | 1.77E-08 | 1.91E-07 | 8.57037 |
| AL450311.1 | -0.5526 | 2.484943 | -5.7301 | 1.78E-08 | 1.92E-07 | 8.56408 |
| PHYHIP | -0.9053 | 5.263286 | -5.7295 | 1.79E-08 | 1.93E-07 | 8.56064 |
| SSTR1 | -0.7969 | 3.119609 | -5.7276 | 1.81E-08 | 1.95E-07 | 8.55072 |
| CHI3L1 | 1.49679 | 4.577935 | 5.72618 | 1.82E-08 | 1.96E-07 | 8.54292 |
| ASCL1 | 0.53874 | 6.454046 | 5.72351 | 1.85E-08 | 1.98E-07 | 8.52865 |
| OAS1 | 0.62469 | 3.075679 | 5.72105 | 1.87E-08 | 2.01E-07 | 8.51552 |
| HLA-DMA | 0.62295 | 5.169884 | 5.71937 | 1.89E-08 | 2.03E-07 | 8.50652 |
| ACSBG1 | -0.5384 | 4.062082 | -5.7088 | 2.00E-08 | 2.14E-07 | 8.45032 |
| TPPP | -0.6248 | 6.287595 | -5.7077 | 2.02E-08 | 2.15E-07 | 8.44415 |
| SIGLEC9 | 0.5112 | 2.324193 | 5.70331 | 2.07E-08 | 2.20E-07 | 8.42094 |
| RASGRF1 | -0.6855 | 3.341665 | -5.6989 | 2.12E-08 | 2.25E-07 | 8.39738 |
| KCNIP2 | -0.8022 | 5.166371 | -5.682 | 2.32E-08 | 2.45E-07 | 8.30796 |
| HLA-DRA | 0.85465 | 8.54253 | 5.67991 | 2.35E-08 | 2.47E-07 | 8.29665 |
| AGAP2-AS1 | 0.63342 | 2.083904 | 5.66521 | 2.55E-08 | 2.67E-07 | 8.21876 |
| LRRC25 | 0.5193 | 2.681143 | 5.66058 | 2.61E-08 | 2.73E-07 | 8.19426 |
| LINC01007 | -0.5864 | 0.60364 | -5.6605 | 2.61E-08 | 2.73E-07 | 8.19376 |
| PPP2R2C | -0.7754 | 4.16648 | -5.6585 | 2.64E-08 | 2.76E-07 | 8.18312 |
| SLC25A21-AS1 | -0.5007 | 2.823731 | -5.6583 | 2.64E-08 | 2.76E-07 | 8.18211 |
| NTRK2 | -0.5593 | 8.016876 | -5.6569 | 2.66E-08 | 2.78E-07 | 8.17489 |
| CTSS | 0.57365 | 4.451737 | 5.65292 | 2.72E-08 | 2.84E-07 | 8.15379 |
| RANBP3L | -0.569 | 4.601965 | -5.6494 | 2.78E-08 | 2.89E-07 | 8.13521 |
| HCP5 | 0.51117 | 2.769105 | 5.6456 | 2.83E-08 | 2.95E-07 | 8.11516 |
| ACTN1 | 0.61258 | 4.416941 | 5.64244 | 2.88E-08 | 2.99E-07 | 8.09852 |
| S100A3 | 0.75163 | 2.037544 | 5.63306 | 3.03E-08 | 3.14E-07 | 8.04911 |
| PLCXD3 | -0.5962 | 2.310387 | -5.6322 | 3.05E-08 | 3.15E-07 | 8.04476 |
| ARHGDIG | -0.6947 | 4.508013 | -5.6315 | 3.06E-08 | 3.16E-07 | 8.04108 |
| AC109439.1 | -0.7346 | 2.178321 | -5.6297 | 3.09E-08 | 3.19E-07 | 8.03157 |
| PYGM | -0.6431 | 3.210558 | -5.6222 | 3.22E-08 | 3.31E-07 | 7.99211 |
| GRB14 | 0.50464 | 1.031084 | 5.61954 | 3.26E-08 | 3.36E-07 | 7.97797 |
| HOXB3 | 0.52961 | 0.480432 | 5.61801 | 3.29E-08 | 3.38E-07 | 7.96992 |
| AP000843.1 | -0.5304 | 1.490109 | -5.6142 | 3.36E-08 | 3.44E-07 | 7.95002 |
| SOCS3 | 0.89643 | 3.544597 | 5.61344 | 3.37E-08 | 3.46E-07 | 7.94595 |
| FOXJ1 | 0.7785 | 3.476257 | 5.60737 | 3.49E-08 | 3.56E-07 | 7.91409 |
| CXCL11 | 0.5184 | 0.882529 | 5.60685 | 3.50E-08 | 3.57E-07 | 7.91138 |
| DYNC1I1 | -0.6197 | 4.018909 | -5.606 | 3.51E-08 | 3.59E-07 | 7.90685 |
| FAM131C | -0.57 | 2.839323 | -5.6019 | 3.59E-08 | 3.66E-07 | 7.8853 |
| HSPA12A | -0.5084 | 4.370546 | -5.6007 | 3.62E-08 | 3.68E-07 | 7.87913 |
| DAAM2 | -0.7081 | 7.001274 | -5.5963 | 3.70E-08 | 3.76E-07 | 7.85609 |
| C1QC | 0.62099 | 8.251769 | 5.59626 | 3.70E-08 | 3.76E-07 | 7.8559 |
| GJB6 | -0.9977 | 2.453792 | -5.5943 | 3.74E-08 | 3.80E-07 | 7.84566 |
| SEMA5B | 0.51327 | 4.374555 | 5.59225 | 3.78E-08 | 3.83E-07 | 7.8349 |
| MYOF | 0.50832 | 3.081192 | 5.56846 | 4.30E-08 | 4.31E-07 | 7.71067 |
| SAA1 | 0.65797 | 0.604498 | 5.56483 | 4.39E-08 | 4.39E-07 | 7.69174 |
| TMEM151B | -0.7507 | 4.357 | -5.5616 | 4.46E-08 | 4.46E-07 | 7.675 |
| COL8A1 | 0.60873 | 0.976426 | 5.5519 | 4.70E-08 | 4.69E-07 | 7.62449 |
| CITED1 | 0.61614 | 3.693564 | 5.54959 | 4.76E-08 | 4.74E-07 | 7.61246 |
| EMP3 | 0.89212 | 3.60591 | 5.5464 | 4.84E-08 | 4.81E-07 | 7.59589 |
| FGL2 | 0.57966 | 3.271167 | 5.53981 | 5.02E-08 | 4.97E-07 | 7.56168 |
| NEUROD6 | -0.766 | 1.396378 | -5.533 | 5.20E-08 | 5.13E-07 | 7.52653 |
| SLC8A2 | -0.7254 | 3.668358 | -5.5268 | 5.38E-08 | 5.29E-07 | 7.49419 |
| ADAMTS15 | 0.66617 | 2.438625 | 5.51704 | 5.67E-08 | 5.55E-07 | 7.44377 |
| ADGRL2 | 0.51862 | 2.982752 | 5.51579 | 5.71E-08 | 5.58E-07 | 7.43731 |
| BCYRN1 | -0.6578 | 2.318353 | -5.5146 | 5.74E-08 | 5.61E-07 | 7.43114 |
| BLM | 0.58406 | 2.755648 | 5.50443 | 6.07E-08 | 5.89E-07 | 7.37861 |
| PEBP4 | -0.5519 | 3.15769 | -5.5026 | 6.12E-08 | 5.95E-07 | 7.36915 |
| AL139393.3 | 0.53442 | 4.0552 | 5.50226 | 6.14E-08 | 5.96E-07 | 7.36743 |
| FCGR3A | 0.77565 | 5.167167 | 5.49924 | 6.24E-08 | 6.05E-07 | 7.35186 |
| SPOCD1 | 0.84772 | 2.027913 | 5.48626 | 6.68E-08 | 6.45E-07 | 7.28502 |
| GPIHBP1 | -0.5726 | 4.228247 | -5.474 | 7.13E-08 | 6.85E-07 | 7.22221 |
| CMYA5 | 0.51312 | 1.204779 | 5.47359 | 7.15E-08 | 6.86E-07 | 7.2199 |
| AC104024.2 | -0.5441 | 0.899515 | -5.4641 | 7.52E-08 | 7.19E-07 | 7.17098 |
| CALHM6 | 0.53943 | 2.250708 | 5.45687 | 7.81E-08 | 7.46E-07 | 7.13417 |
| FCGR2A | 0.57338 | 3.419206 | 5.45546 | 7.87E-08 | 7.50E-07 | 7.12696 |
| OLFML2B | 0.62075 | 3.547267 | 5.45192 | 8.02E-08 | 7.63E-07 | 7.10887 |
| MIR3176 | 0.50807 | 2.089692 | 5.44564 | 8.29E-08 | 7.87E-07 | 7.07671 |
| APOL1 | 0.5111 | 3.286158 | 5.43361 | 8.83E-08 | 8.35E-07 | 7.01529 |
| STAB1 | 0.538 | 4.292045 | 5.4252 | 9.23E-08 | 8.71E-07 | 6.97243 |
| FAM133A | -0.5156 | 3.02335 | -5.4217 | 9.41E-08 | 8.86E-07 | 6.95449 |
| HLA-DPB1 | 0.76115 | 6.011524 | 5.42095 | 9.44E-08 | 8.89E-07 | 6.95079 |
| CLEC5A | 0.53813 | 0.9295 | 5.41859 | 9.56E-08 | 8.99E-07 | 6.93877 |
| HLA-DQB1 | 0.80624 | 3.714582 | 5.41723 | 9.63E-08 | 9.05E-07 | 6.93188 |
| MS4A7 | 0.56804 | 4.043645 | 5.41663 | 9.66E-08 | 9.07E-07 | 6.92884 |
| SFRP4 | 0.71604 | 2.894937 | 5.41044 | 9.98E-08 | 9.34E-07 | 6.89734 |
| BCL2A1 | 0.6158 | 2.081397 | 5.40511 | 1.03E-07 | 9.58E-07 | 6.8703 |
| CLCF1 | 0.52977 | 1.56726 | 5.39521 | 1.08E-07 | 1.00E-06 | 6.82003 |
| PSRC1 | 0.53866 | 5.201746 | 5.38987 | 1.11E-07 | 1.03E-06 | 6.79301 |
| S1PR3 | 0.60183 | 3.477774 | 5.38661 | 1.13E-07 | 1.05E-06 | 6.77647 |
| GPC3 | 0.53664 | 1.546523 | 5.38455 | 1.14E-07 | 1.06E-06 | 6.76608 |
| NNMT | 0.72688 | 2.000651 | 5.3841 | 1.15E-07 | 1.06E-06 | 6.76378 |
| IQCA1 | -0.5501 | 2.062921 | -5.3837 | 1.15E-07 | 1.06E-06 | 6.76157 |
| H3C6 | 0.50407 | 1.595 | 5.36937 | 1.24E-07 | 1.14E-06 | 6.68935 |
| HPR | -0.7595 | 3.834047 | -5.3674 | 1.25E-07 | 1.15E-06 | 6.67946 |
| CALY | -0.8677 | 3.121867 | -5.3642 | 1.27E-07 | 1.17E-06 | 6.66317 |
| MDK | 0.62842 | 5.356403 | 5.35677 | 1.32E-07 | 1.21E-06 | 6.62579 |
| AL354919.2 | 0.65529 | 1.21668 | 5.35655 | 1.32E-07 | 1.21E-06 | 6.6247 |
| GADD45A | 0.55614 | 5.414513 | 5.35303 | 1.35E-07 | 1.23E-06 | 6.60697 |
| MIR3125 | 0.51892 | 1.55203 | 5.34672 | 1.39E-07 | 1.27E-06 | 6.57521 |
| AK5 | -0.7982 | 3.925127 | -5.3357 | 1.48E-07 | 1.34E-06 | 6.51969 |
| FAM81A | -0.5388 | 3.09481 | -5.3318 | 1.51E-07 | 1.36E-06 | 6.50039 |
| CCL5 | 0.58382 | 2.173303 | 5.32852 | 1.53E-07 | 1.38E-06 | 6.48387 |
| FRMPD4 | -0.5859 | 1.506096 | -5.3268 | 1.54E-07 | 1.39E-06 | 6.47531 |
| KCTD4 | -0.5129 | 3.164872 | -5.3138 | 1.65E-07 | 1.48E-06 | 6.41003 |
| MAP3K7CL | 0.51329 | 1.580716 | 5.30925 | 1.69E-07 | 1.51E-06 | 6.38741 |
| HLA-DRB5 | 0.83008 | 5.979075 | 5.30699 | 1.71E-07 | 1.53E-06 | 6.37608 |
| PKIB | 0.57015 | 1.923562 | 5.30637 | 1.72E-07 | 1.53E-06 | 6.37298 |
| MYBPC1 | -0.605 | 3.226323 | -5.3044 | 1.74E-07 | 1.55E-06 | 6.36323 |
| CAPG | 0.61637 | 5.322924 | 5.30183 | 1.76E-07 | 1.56E-06 | 6.35034 |
| GBP1 | 0.69013 | 3.628924 | 5.28296 | 1.94E-07 | 1.71E-06 | 6.25628 |
| MATN2 | 0.5343 | 5.374021 | 5.28216 | 1.95E-07 | 1.72E-06 | 6.25232 |
| ANKRD22 | 0.69647 | 2.425002 | 5.27725 | 2.00E-07 | 1.76E-06 | 6.22793 |
| OASL | 0.51433 | 1.405668 | 5.27083 | 2.06E-07 | 1.81E-06 | 6.19603 |
| PLP2 | 0.60102 | 4.643193 | 5.27011 | 2.07E-07 | 1.82E-06 | 6.19245 |
| C1QA | 0.59366 | 7.805767 | 5.26801 | 2.09E-07 | 1.84E-06 | 6.182 |
| LINC02381 | 0.60081 | 3.792466 | 5.25953 | 2.19E-07 | 1.91E-06 | 6.13996 |
| CD74 | 0.70006 | 9.048877 | 5.25902 | 2.19E-07 | 1.92E-06 | 6.13745 |
| LINC00499 | -0.502 | 0.942501 | -5.2564 | 2.22E-07 | 1.94E-06 | 6.12445 |
| SST | -0.898 | 4.30203 | -5.2498 | 2.30E-07 | 2.00E-06 | 6.0917 |
| LINC02488 | 0.6103 | 1.21056 | 5.24921 | 2.31E-07 | 2.01E-06 | 6.08888 |
| EMILIN3 | 0.88197 | 2.07 | 5.24877 | 2.31E-07 | 2.01E-06 | 6.08667 |
| MPPED1 | -0.7614 | 2.108876 | -5.2457 | 2.35E-07 | 2.04E-06 | 6.0716 |
| CXCL5 | -0.5147 | 1.828323 | -5.2443 | 2.36E-07 | 2.05E-06 | 6.06464 |
| CABLES1 | -0.5415 | 4.010756 | -5.2367 | 2.46E-07 | 2.12E-06 | 6.02721 |
| MTND6P4 | -0.541 | 2.567678 | -5.2332 | 2.50E-07 | 2.16E-06 | 6.00964 |
| AC008780.1 | -0.5835 | 1.188654 | -5.2284 | 2.57E-07 | 2.21E-06 | 5.98593 |
| HHATL | -0.6505 | 4.520328 | -5.2236 | 2.63E-07 | 2.26E-06 | 5.9623 |
| RYR2 | -0.5614 | 1.313942 | -5.218 | 2.70E-07 | 2.32E-06 | 5.93508 |
| SIGLEC1 | 0.55446 | 1.877044 | 5.21704 | 2.72E-07 | 2.33E-06 | 5.93013 |
| AC023421.1 | -0.6493 | 1.686189 | -5.2086 | 2.84E-07 | 2.42E-06 | 5.88874 |
| MT-TE | -0.5021 | 1.447388 | -5.2073 | 2.86E-07 | 2.43E-06 | 5.8824 |
| TNFRSF19 | 0.55808 | 3.729485 | 5.20605 | 2.88E-07 | 2.45E-06 | 5.87614 |
| SLC11A1 | 0.63788 | 2.618899 | 5.19922 | 2.98E-07 | 2.53E-06 | 5.84258 |
| SNAP91 | -0.6307 | 5.164439 | -5.19 | 3.12E-07 | 2.64E-06 | 5.79752 |
| SEL1L3 | 0.64719 | 2.4282 | 5.18834 | 3.15E-07 | 2.66E-06 | 5.7893 |
| CARTPT | -0.7046 | 0.854107 | -5.1859 | 3.19E-07 | 2.69E-06 | 5.77751 |
| CPNE9 | -0.5406 | 1.576323 | -5.1827 | 3.24E-07 | 2.73E-06 | 5.76161 |
| SYP | -0.5248 | 6.459307 | -5.1809 | 3.27E-07 | 2.76E-06 | 5.75292 |
| TMEM130 | -0.9111 | 4.29666 | -5.1742 | 3.38E-07 | 2.85E-06 | 5.72005 |
| MATK | -0.5446 | 2.632876 | -5.1716 | 3.43E-07 | 2.88E-06 | 5.70729 |
| TMEM158 | 0.60847 | 4.514852 | 5.17153 | 3.43E-07 | 2.88E-06 | 5.70711 |
| STUM | -0.5546 | 4.606373 | -5.1715 | 3.43E-07 | 2.88E-06 | 5.70705 |
| SOWAHA | -0.6597 | 3.09552 | -5.1619 | 3.60E-07 | 3.01E-06 | 5.66007 |
| HLA-DOA | 0.69887 | 3.467159 | 5.15981 | 3.64E-07 | 3.04E-06 | 5.64993 |
| NPM2 | -0.6311 | 2.771898 | -5.1543 | 3.74E-07 | 3.12E-06 | 5.62293 |
| PDGFA | 0.50939 | 4.277021 | 5.15109 | 3.80E-07 | 3.17E-06 | 5.6075 |
| HLA-DMB | 0.56987 | 3.977446 | 5.14856 | 3.85E-07 | 3.20E-06 | 5.59519 |
| FCER1G | 0.55404 | 6.051402 | 5.14731 | 3.87E-07 | 3.22E-06 | 5.58913 |
| CABP1 | -0.6448 | 3.017664 | -5.1429 | 3.96E-07 | 3.28E-06 | 5.56776 |
| TUBB4A | -0.5564 | 8.092545 | -5.1381 | 4.06E-07 | 3.36E-06 | 5.54458 |
| AC092162.3 | -0.5937 | 1.938989 | -5.1223 | 4.39E-07 | 3.60E-06 | 5.46782 |
| LGI3 | -0.6484 | 4.921313 | -5.1173 | 4.50E-07 | 3.68E-06 | 5.44377 |
| SRPX2 | 0.6235 | 2.214176 | 5.10822 | 4.71E-07 | 3.84E-06 | 5.39974 |
| TBR1 | -0.5798 | 1.408239 | -5.0952 | 5.03E-07 | 4.08E-06 | 5.33698 |
| PNMT | -0.5405 | 1.827794 | -5.0949 | 5.04E-07 | 4.08E-06 | 5.33561 |
| SV2B | -0.8321 | 2.463699 | -5.0845 | 5.31E-07 | 4.29E-06 | 5.28561 |
| C1QTNF4 | -0.5347 | 3.099248 | -5.0758 | 5.54E-07 | 4.46E-06 | 5.24382 |
| SLC6A7 | -0.7037 | 1.489402 | -5.0745 | 5.58E-07 | 4.49E-06 | 5.23748 |
| AIFM3 | -0.6588 | 2.935457 | -5.065 | 5.85E-07 | 4.68E-06 | 5.19183 |
| ASIC2 | -0.5049 | 1.61334 | -5.0612 | 5.96E-07 | 4.77E-06 | 5.17369 |
| SYTL4 | -0.5348 | 4.858964 | -5.0603 | 5.99E-07 | 4.78E-06 | 5.16918 |
| ASPA | -0.5086 | 3.211317 | -5.0585 | 6.04E-07 | 4.82E-06 | 5.16076 |
| BST2 | 0.5185 | 6.491625 | 5.05717 | 6.08E-07 | 4.85E-06 | 5.15435 |
| CHRNA1 | 0.55829 | 0.941423 | 5.05393 | 6.18E-07 | 4.92E-06 | 5.13884 |
| ATP2B3 | -0.6709 | 2.256356 | -5.0499 | 6.31E-07 | 5.01E-06 | 5.11961 |
| JAKMIP1 | -0.5836 | 3.428747 | -5.0497 | 6.32E-07 | 5.02E-06 | 5.11856 |
| VSNL1 | -1.1113 | 4.581628 | -5.0486 | 6.35E-07 | 5.04E-06 | 5.11344 |
| PEX5L | -0.5665 | 3.34298 | -5.0479 | 6.37E-07 | 5.06E-06 | 5.10984 |
| PACSIN1 | -1.0277 | 4.143944 | -5.0364 | 6.75E-07 | 5.33E-06 | 5.05512 |
| NEUROD2 | -0.624 | 1.706113 | -5.0328 | 6.87E-07 | 5.42E-06 | 5.03785 |
| AC021613.1 | -0.5001 | 0.936789 | -5.0321 | 6.89E-07 | 5.44E-06 | 5.03466 |
| NGEF | -0.7463 | 4.162487 | -5.0312 | 6.92E-07 | 5.46E-06 | 5.03028 |
| ABCC3 | 0.65223 | 1.130626 | 5.0268 | 7.07E-07 | 5.57E-06 | 5.0094 |
| AL445250.1 | 0.51189 | 1.789692 | 5.02575 | 7.11E-07 | 5.60E-06 | 5.00443 |
| STOX1 | -0.602 | 4.229203 | -5.0251 | 7.13E-07 | 5.61E-06 | 5.00124 |
| ABCC8 | -0.6654 | 3.759673 | -5.0095 | 7.71E-07 | 6.01E-06 | 4.92713 |
| UNC13C | -0.5288 | 1.303108 | -4.9967 | 8.21E-07 | 6.38E-06 | 4.86634 |
| KBTBD11-OT1 | -0.5689 | 3.39052 | -4.9897 | 8.49E-07 | 6.59E-06 | 4.83357 |
| HTR5A | -0.528 | 1.175706 | -4.9854 | 8.68E-07 | 6.71E-06 | 4.81306 |
| CACNG3 | -0.8332 | 2.441276 | -4.9815 | 8.85E-07 | 6.83E-06 | 4.79456 |
| SNCB | -0.9003 | 5.719118 | -4.9747 | 9.14E-07 | 7.04E-06 | 4.76283 |
| FOSL1 | 0.51426 | 1.883727 | 4.96946 | 9.38E-07 | 7.22E-06 | 4.73789 |
| TCEAL6 | -0.7264 | 3.521157 | -4.9685 | 9.43E-07 | 7.24E-06 | 4.7332 |
| CD163 | 0.84116 | 3.178005 | 4.9684 | 9.43E-07 | 7.25E-06 | 4.73291 |
| ALOX5AP | 0.63647 | 4.593455 | 4.94782 | 1.04E-06 | 7.96E-06 | 4.63616 |
| SOHLH1 | -0.5673 | 1.431313 | -4.9372 | 1.10E-06 | 8.34E-06 | 4.58641 |
| IBSP | 0.56891 | 0.610226 | 4.93719 | 1.10E-06 | 8.34E-06 | 4.58637 |
| RNASE2 | 0.63117 | 2.515737 | 4.91046 | 1.25E-06 | 9.41E-06 | 4.46147 |
| SULT4A1 | -0.9414 | 4.083634 | -4.9046 | 1.29E-06 | 9.66E-06 | 4.43433 |
| RCAN2 | -0.5763 | 4.733683 | -4.9043 | 1.29E-06 | 9.67E-06 | 4.43303 |
| GPR22 | -0.5282 | 1.251421 | -4.8959 | 1.34E-06 | 1.00E-05 | 4.39384 |
| GABRA5 | -0.7567 | 2.421527 | -4.8822 | 1.43E-06 | 1.07E-05 | 4.32995 |
| PPP1R16B | -0.5405 | 4.519265 | -4.8804 | 1.45E-06 | 1.08E-05 | 4.32159 |
| LINC02058 | -0.5374 | 1.96535 | -4.8782 | 1.46E-06 | 1.09E-05 | 4.31145 |
| C1R | 0.63124 | 4.910229 | 4.87383 | 1.49E-06 | 1.11E-05 | 4.29135 |
| SYNPR | -0.7864 | 2.66604 | -4.8721 | 1.51E-06 | 1.12E-05 | 4.28342 |
| CCK | -0.9158 | 3.519326 | -4.865 | 1.56E-06 | 1.15E-05 | 4.25068 |
| CFI | 0.61378 | 3.349632 | 4.86189 | 1.58E-06 | 1.17E-05 | 4.23615 |
| VIM | 0.70103 | 8.379418 | 4.85389 | 1.64E-06 | 1.21E-05 | 4.19924 |
| WSCD2 | -0.6 | 2.092049 | -4.8475 | 1.70E-06 | 1.25E-05 | 4.16972 |
| MAL | -0.8379 | 5.333544 | -4.8399 | 1.76E-06 | 1.29E-05 | 4.13477 |
| SYN2 | -0.7513 | 4.598127 | -4.8384 | 1.77E-06 | 1.30E-05 | 4.12801 |
| GRIA1 | -0.6286 | 5.738317 | -4.8355 | 1.80E-06 | 1.31E-05 | 4.11444 |
| SPP1 | 0.74587 | 8.954714 | 4.83395 | 1.81E-06 | 1.32E-05 | 4.10747 |
| TNFSF13B | 0.65493 | 3.11249 | 4.8333 | 1.81E-06 | 1.32E-05 | 4.10448 |
| DLGAP1-AS4 | -0.5832 | 1.591031 | -4.8288 | 1.85E-06 | 1.35E-05 | 4.08376 |
| CNNM1 | -0.5151 | 2.004629 | -4.8283 | 1.86E-06 | 1.35E-05 | 4.0817 |
| LRFN5 | -0.5122 | 2.15376 | -4.8036 | 2.09E-06 | 1.51E-05 | 3.96864 |
| TF | -0.6059 | 6.660295 | -4.7972 | 2.16E-06 | 1.55E-05 | 3.93938 |
| HSPA7 | 0.5526 | 1.531783 | 4.7954 | 2.17E-06 | 1.56E-05 | 3.93101 |
| TLR2 | 0.54923 | 3.261778 | 4.79277 | 2.20E-06 | 1.58E-05 | 3.91904 |
| GOLGA7B | -0.5108 | 3.954704 | -4.7775 | 2.37E-06 | 1.68E-05 | 3.84946 |
| CACNA1B | -0.5277 | 2.039884 | -4.7744 | 2.40E-06 | 1.71E-05 | 3.83526 |
| FBXL16 | -0.5284 | 6.56774 | -4.766 | 2.50E-06 | 1.77E-05 | 3.79734 |
| MS4A4A | 0.52089 | 3.443363 | 4.75656 | 2.61E-06 | 1.84E-05 | 3.75449 |
| NECAB1 | -0.5673 | 3.550301 | -4.7558 | 2.62E-06 | 1.85E-05 | 3.75106 |
| MFSD4A | -0.5202 | 3.283627 | -4.7453 | 2.76E-06 | 1.93E-05 | 3.70366 |
| PRKCG | -0.7812 | 2.967166 | -4.7367 | 2.87E-06 | 2.00E-05 | 3.66489 |
| SEC14L5 | -0.6053 | 2.872475 | -4.7366 | 2.87E-06 | 2.00E-05 | 3.66418 |
| SSTR2 | -0.6005 | 3.670205 | -4.7163 | 3.16E-06 | 2.19E-05 | 3.57315 |
| NEFM | -0.9541 | 3.430654 | -4.716 | 3.17E-06 | 2.19E-05 | 3.57156 |
| SPOCK3 | -0.6074 | 4.496098 | -4.7125 | 3.22E-06 | 2.22E-05 | 3.556 |
| HOXD8 | 0.68502 | 1.664381 | 4.7091 | 3.27E-06 | 2.25E-05 | 3.54064 |
| C7orf57 | 0.56003 | 0.917365 | 4.70702 | 3.30E-06 | 2.27E-05 | 3.53127 |
| GPNMB | 0.71364 | 3.645654 | 4.70592 | 3.32E-06 | 2.28E-05 | 3.52637 |
| NGB | -0.6471 | 1.480308 | -4.6962 | 3.47E-06 | 2.38E-05 | 3.48259 |
| CBLN2 | -0.5419 | 1.938325 | -4.6824 | 3.70E-06 | 2.53E-05 | 3.42136 |
| OGDHL | -0.6103 | 2.85729 | -4.6774 | 3.79E-06 | 2.58E-05 | 3.39867 |
| SPRY4 | 0.67955 | 3.298591 | 4.67617 | 3.81E-06 | 2.59E-05 | 3.39337 |
| CAMK2A | -1.0159 | 4.698479 | -4.676 | 3.82E-06 | 2.60E-05 | 3.39256 |
| NAPB | -0.556 | 5.398428 | -4.665 | 4.02E-06 | 2.72E-05 | 3.3438 |
| MAL2 | -0.7215 | 2.210172 | -4.6646 | 4.02E-06 | 2.72E-05 | 3.34181 |
| AC090204.1 | 0.55517 | 2.825728 | 4.65816 | 4.15E-06 | 2.80E-05 | 3.31321 |
| VIP | -0.5591 | 1.728085 | -4.6544 | 4.22E-06 | 2.84E-05 | 3.29653 |
| FABP5 | 0.61269 | 2.748321 | 4.65158 | 4.27E-06 | 2.88E-05 | 3.284 |
| PRKCB | -0.5701 | 4.281533 | -4.6402 | 4.51E-06 | 3.02E-05 | 3.23356 |
| FBLN5 | 0.50417 | 3.248421 | 4.63967 | 4.52E-06 | 3.03E-05 | 3.23121 |
| TMEM155 | -0.6482 | 1.942578 | -4.6285 | 4.76E-06 | 3.17E-05 | 3.18181 |
| CYP2J2 | -0.5081 | 3.98005 | -4.6279 | 4.77E-06 | 3.18E-05 | 3.17906 |
| HRH3 | -0.6599 | 3.02902 | -4.6084 | 5.22E-06 | 3.45E-05 | 3.09336 |
| GPX3 | -0.7606 | 6.992094 | -4.608 | 5.23E-06 | 3.46E-05 | 3.09134 |
| DUSP5 | 0.55299 | 2.770853 | 4.60594 | 5.28E-06 | 3.49E-05 | 3.08244 |
| CNTN3 | -0.5907 | 2.859198 | -4.5996 | 5.43E-06 | 3.58E-05 | 3.05471 |
| RAB3A | -0.5995 | 5.917121 | -4.5947 | 5.56E-06 | 3.66E-05 | 3.03307 |
| SLC17A7 | -1.0415 | 4.603526 | -4.5913 | 5.64E-06 | 3.71E-05 | 3.01826 |
| JPH3 | -0.5768 | 4.811673 | -4.5778 | 6.01E-06 | 3.92E-05 | 2.95902 |
| SCIN | 0.56743 | 2.56451 | 4.57309 | 6.14E-06 | 3.99E-05 | 2.93846 |
| FRRS1L | -0.5437 | 3.790901 | -4.5729 | 6.14E-06 | 4.00E-05 | 2.93753 |
| TMEM151A | -0.5402 | 4.830281 | -4.5691 | 6.25E-06 | 4.06E-05 | 2.92097 |
| NGFR | 0.5943 | 3.224327 | 4.56511 | 6.36E-06 | 4.13E-05 | 2.90367 |
| ITGB2 | 0.5065 | 4.912263 | 4.55055 | 6.80E-06 | 4.39E-05 | 2.84023 |
| GDA | -0.7356 | 2.521455 | -4.5394 | 7.15E-06 | 4.60E-05 | 2.79197 |
| GABRA1 | -0.8163 | 2.769563 | -4.5315 | 7.42E-06 | 4.75E-05 | 2.75753 |
| TGFB2 | 0.65673 | 3.319504 | 4.52928 | 7.49E-06 | 4.80E-05 | 2.74795 |
| CHD5 | -0.6461 | 3.019613 | -4.5277 | 7.55E-06 | 4.83E-05 | 2.74092 |
| SLC26A4-AS1 | -0.5955 | 1.574125 | -4.5225 | 7.72E-06 | 4.93E-05 | 2.71873 |
| RASAL1 | -0.5965 | 2.043987 | -4.505 | 8.36E-06 | 5.31E-05 | 2.64327 |
| HAMP | 0.54396 | 1.889638 | 4.50315 | 8.43E-06 | 5.35E-05 | 2.63512 |
| NWD1 | -0.5006 | 2.309174 | -4.5013 | 8.50E-06 | 5.39E-05 | 2.6272 |
| CCKBR | -0.5608 | 1.657781 | -4.497 | 8.67E-06 | 5.48E-05 | 2.60855 |
| CNGA3 | 0.68121 | 2.667483 | 4.48581 | 9.12E-06 | 5.74E-05 | 2.56058 |
| GABRB2 | -0.6951 | 2.185867 | -4.4838 | 9.20E-06 | 5.78E-05 | 2.55194 |
| ENTPD3 | -0.5042 | 1.569036 | -4.4826 | 9.25E-06 | 5.81E-05 | 2.54687 |
| SYT13 | -0.7572 | 3.281219 | -4.4816 | 9.29E-06 | 5.84E-05 | 2.54239 |
| CRTAC1 | -0.6081 | 5.545226 | -4.4808 | 9.33E-06 | 5.86E-05 | 2.53897 |
| DES | 0.54969 | 1.10099 | 4.4779 | 9.45E-06 | 5.93E-05 | 2.52665 |
| GRIN1 | -0.8326 | 4.335483 | -4.4668 | 9.93E-06 | 6.21E-05 | 2.47905 |
| KCNH3 | -0.5875 | 3.27266 | -4.4666 | 9.94E-06 | 6.21E-05 | 2.47833 |
| RNA5SP118 | 0.52447 | 3.137364 | 4.40856 | 1.29E-05 | 7.85E-05 | 2.23172 |
| LINC01338 | -0.583 | 3.591184 | -4.4069 | 1.30E-05 | 7.90E-05 | 2.22455 |
| IGF2 | 0.53967 | 3.455319 | 4.4022 | 1.32E-05 | 8.05E-05 | 2.20485 |
| CHRM1 | -0.6572 | 3.404036 | -4.3986 | 1.35E-05 | 8.17E-05 | 2.18949 |
| FABP7 | 0.61 | 5.983055 | 4.39676 | 1.36E-05 | 8.22E-05 | 2.18192 |
| TMEM235 | -0.6568 | 2.645181 | -4.3965 | 1.36E-05 | 8.23E-05 | 2.18098 |
| SNAP25 | -0.8217 | 7.091278 | -4.3744 | 1.50E-05 | 8.99E-05 | 2.08789 |
| CAMK2B | -0.565 | 4.38013 | -4.3725 | 1.51E-05 | 9.06E-05 | 2.07996 |
| RBFOX1 | -0.6453 | 2.498488 | -4.3605 | 1.59E-05 | 9.52E-05 | 2.02986 |
| ID3 | 0.53586 | 8.168403 | 4.34795 | 1.68E-05 | 0.0001 | 1.97737 |
| AQP5 | 0.51335 | 0.912133 | 4.34679 | 1.69E-05 | 0.000101 | 1.97252 |
| GAP43 | 0.52402 | 7.89634 | 4.34073 | 1.74E-05 | 0.000103 | 1.94725 |
| CREG2 | -0.7651 | 2.837636 | -4.3337 | 1.79E-05 | 0.000106 | 1.91782 |
| KCNK1 | -0.5268 | 3.538694 | -4.3311 | 1.81E-05 | 0.000107 | 1.90738 |
| THBS1 | 0.51285 | 1.945703 | 4.32171 | 1.89E-05 | 0.000111 | 1.86823 |
| SNCA | -0.5684 | 4.426722 | -4.3068 | 2.01E-05 | 0.000118 | 1.80641 |
| GRM3 | -0.542 | 3.894917 | -4.3063 | 2.02E-05 | 0.000118 | 1.80443 |
| CLEC2L | -0.5883 | 2.132832 | -4.3032 | 2.04E-05 | 0.000119 | 1.79171 |
| HLA-DQB2 | 0.55605 | 1.721505 | 4.29716 | 2.10E-05 | 0.000122 | 1.76671 |
| GRIN3A | -0.5635 | 2.210003 | -4.2968 | 2.10E-05 | 0.000123 | 1.76533 |
| RBP4 | -0.5903 | 2.338123 | -4.2933 | 2.13E-05 | 0.000124 | 1.75072 |
| OLFM3 | -0.5279 | 1.564697 | -4.2893 | 2.17E-05 | 0.000126 | 1.73428 |
| SLC24A2 | -0.5116 | 3.617955 | -4.2889 | 2.18E-05 | 0.000126 | 1.73287 |
| TGFB2-AS1 | 0.52122 | 2.650242 | 4.28791 | 2.18E-05 | 0.000127 | 1.7286 |
| NSG2 | -0.6927 | 6.766044 | -4.2861 | 2.20E-05 | 0.000128 | 1.72129 |
| G0S2 | 0.58245 | 1.738457 | 4.27893 | 2.27E-05 | 0.000131 | 1.69166 |
| SHISA6 | -0.6623 | 3.094166 | -4.2645 | 2.42E-05 | 0.000139 | 1.63246 |
| PCDHB7 | 0.50205 | 2.357128 | 4.26193 | 2.44E-05 | 0.00014 | 1.62196 |
| GBP3 | 0.54958 | 3.427299 | 4.25983 | 2.47E-05 | 0.000142 | 1.61337 |
| PLA2G2A | 0.51943 | 0.584454 | 4.25352 | 2.53E-05 | 0.000145 | 1.58755 |
| CYBB | 0.50899 | 4.897517 | 4.25283 | 2.54E-05 | 0.000145 | 1.58474 |
| SYT1 | -0.828 | 4.895078 | -4.2484 | 2.59E-05 | 0.000148 | 1.56677 |
| DDN | -0.7881 | 3.825831 | -4.2478 | 2.60E-05 | 0.000148 | 1.56435 |
| PSD | -0.5139 | 5.445245 | -4.2447 | 2.63E-05 | 0.00015 | 1.5516 |
| GALNT9 | -0.5275 | 3.145149 | -4.234 | 2.76E-05 | 0.000156 | 1.50798 |
| SLC12A5 | -0.6462 | 3.235115 | -4.2317 | 2.78E-05 | 0.000158 | 1.49877 |
| ATP8A2 | -0.5241 | 2.273198 | -4.2154 | 2.98E-05 | 0.000168 | 1.43257 |
| HCN1 | -0.5477 | 1.593619 | -4.198 | 3.21E-05 | 0.00018 | 1.36197 |
| FPR1 | 0.55415 | 3.922619 | 4.19544 | 3.25E-05 | 0.000181 | 1.35176 |
| ACY3 | -0.6293 | 3.5572 | -4.1919 | 3.30E-05 | 0.000184 | 1.33767 |
| RBFOX3 | -0.566 | 2.132831 | -4.1744 | 3.55E-05 | 0.000197 | 1.26716 |
| ELFN2 | -0.5117 | 4.798884 | -4.171 | 3.60E-05 | 0.0002 | 1.25339 |
| NEFL | -0.944 | 4.277055 | -4.1465 | 4.00E-05 | 0.000219 | 1.15559 |
| GEM | 0.50457 | 4.202539 | 4.14266 | 4.06E-05 | 0.000222 | 1.14006 |
| SHISAL1 | -0.592 | 3.323926 | -4.1398 | 4.11E-05 | 0.000224 | 1.12861 |
| OPALIN | -0.833 | 3.34358 | -4.1293 | 4.30E-05 | 0.000233 | 1.08695 |
| FAM183A | 0.50699 | 0.730819 | 4.12151 | 4.44E-05 | 0.00024 | 1.05595 |
| COL11A1 | 0.5608 | 2.706451 | 4.12112 | 4.45E-05 | 0.000241 | 1.05441 |
| AL121827.2 | -0.5101 | 6.052915 | -4.0996 | 4.87E-05 | 0.000261 | 0.96924 |
| CD14 | 0.50606 | 6.385504 | 4.09349 | 4.99E-05 | 0.000267 | 0.94513 |
| TAFA1 | -0.522 | 2.003405 | -4.0851 | 5.17E-05 | 0.000276 | 0.91217 |
| CNTNAP4 | -0.5429 | 3.710427 | -4.0839 | 5.20E-05 | 0.000277 | 0.90726 |
| SLC6A17 | -0.6928 | 3.300621 | -4.0755 | 5.38E-05 | 0.000285 | 0.87434 |
| DOC2A | -0.5087 | 2.436234 | -4.061 | 5.71E-05 | 0.000301 | 0.81765 |
| BEX5 | -0.5733 | 4.358412 | -4.046 | 6.08E-05 | 0.000319 | 0.75896 |
| C1orf115 | -0.5169 | 4.011284 | -4.0431 | 6.15E-05 | 0.000322 | 0.74746 |
| GLT1D1 | -0.5084 | 2.190503 | -4.0411 | 6.20E-05 | 0.000325 | 0.73986 |
| GNG3 | -0.7676 | 5.157287 | -4.024 | 6.66E-05 | 0.000346 | 0.67313 |
| CRYM | -0.7058 | 2.825982 | -4.0233 | 6.67E-05 | 0.000347 | 0.67061 |
| NKAIN2 | -0.5184 | 3.56651 | -4.0131 | 6.96E-05 | 0.000361 | 0.63094 |
| IGFBPL1 | 0.56695 | 2.61897 | 3.99836 | 7.39E-05 | 0.000381 | 0.57407 |
| S100A1 | -0.5004 | 5.352966 | -3.9957 | 7.47E-05 | 0.000385 | 0.56365 |
| SYT4 | -0.7095 | 3.556194 | -3.9926 | 7.57E-05 | 0.000389 | 0.55173 |
| C3 | 0.56732 | 7.019144 | 3.98598 | 7.78E-05 | 0.000399 | 0.52641 |
| AL022313.4 | -0.6717 | 3.945487 | -3.9744 | 8.15E-05 | 0.000416 | 0.48184 |
| TESPA1 | -0.6633 | 1.629096 | -3.9527 | 8.90E-05 | 0.00045 | 0.399 |
| VSTM2A | -0.6182 | 3.376336 | -3.9527 | 8.90E-05 | 0.00045 | 0.39894 |
| IFI44L | 0.51004 | 3.874662 | 3.92193 | 0.0001 | 0.000504 | 0.28193 |
| IGFBP3 | 0.61139 | 4.637248 | 3.91236 | 0.0001 | 0.000522 | 0.24568 |
| CAMKV | -0.5808 | 4.158688 | -3.9038 | 0.00011 | 0.000538 | 0.21343 |
| LY6H | -0.5414 | 5.11528 | -3.8953 | 0.00011 | 0.000555 | 0.18127 |
| SVOP | -0.6316 | 3.244809 | -3.8945 | 0.00011 | 0.000557 | 0.17832 |
| COL22A1 | 0.51935 | 1.802242 | 3.89354 | 0.00011 | 0.000559 | 0.17474 |
| PVALB | -0.6104 | 1.929257 | -3.8821 | 0.00012 | 0.000581 | 0.13182 |
| IGSF1 | -0.522 | 3.191715 | -3.8803 | 0.00012 | 0.000585 | 0.12483 |
| FLNC | 0.57105 | 3.533048 | 3.87798 | 0.00012 | 0.00059 | 0.1163 |
| C21orf62 | 0.52526 | 2.900641 | 3.85434 | 0.00013 | 0.000642 | 0.02793 |
| LTF | 0.77305 | 1.663225 | 3.84929 | 0.00013 | 0.000653 | 0.00913 |
| CAMK1G | -0.6574 | 2.540346 | -3.8461 | 0.00014 | 0.000661 | -0.0027 |
| MAP7D2 | -0.6108 | 2.40291 | -3.8361 | 0.00014 | 0.000684 | -0.0399 |
| NPTX1 | -0.659 | 4.03567 | -3.8137 | 0.00015 | 0.000741 | -0.1229 |
| FAM163B | -0.6589 | 4.506696 | -3.802 | 0.00016 | 0.000771 | -0.166 |
| MT-TF | -0.545 | 4.783588 | -3.7954 | 0.00017 | 0.000789 | -0.1902 |
| OR4N2 | 0.51812 | 1.355673 | 3.79134 | 0.00017 | 0.0008 | -0.2051 |
| SYNGR3 | -0.555 | 3.590571 | -3.79 | 0.00017 | 0.000804 | -0.21 |
| GBP2 | 0.52288 | 3.738614 | 3.7756 | 0.00018 | 0.000845 | -0.2627 |
| C1QL3 | -0.5754 | 1.719492 | -3.7751 | 0.00018 | 0.000846 | -0.2647 |
| RBP1 | 0.55023 | 3.253077 | 3.77037 | 0.00018 | 0.00086 | -0.2818 |
| NRGN | -0.8296 | 6.868153 | -3.7653 | 0.00019 | 0.000876 | -0.3004 |
| SYN1 | -0.5611 | 6.005985 | -3.7318 | 0.00021 | 0.000983 | -0.4221 |
| TRH | 0.5916 | 1.839834 | 3.72455 | 0.00022 | 0.001007 | -0.4481 |
| MT-TM | -0.5013 | 2.800556 | -3.6944 | 0.00025 | 0.00112 | -0.5565 |
| PHF24 | -0.5818 | 3.228791 | -3.6854 | 0.00025 | 0.001155 | -0.5887 |
| GAD2 | -0.5616 | 2.27765 | -3.6649 | 0.00028 | 0.001237 | -0.6616 |
| WIF1 | -0.6171 | 1.783799 | -3.6593 | 0.00028 | 0.00126 | -0.6816 |
| RTP5 | -0.5593 | 4.694043 | -3.6505 | 0.00029 | 0.001297 | -0.7127 |
| GSX1 | 0.54949 | 2.690894 | 3.64061 | 0.0003 | 0.001344 | -0.7478 |
| EPHB6 | -0.5265 | 3.517703 | -3.6201 | 0.00033 | 0.001441 | -0.8202 |
| CELF4 | -0.5274 | 3.514282 | -3.6194 | 0.00033 | 0.001443 | -0.8225 |
| SLC30A3 | -0.5251 | 1.700292 | -3.613 | 0.00033 | 0.001475 | -0.8452 |
| TMEM125 | -0.5593 | 3.209537 | -3.6119 | 0.00034 | 0.00148 | -0.8488 |
| INA | -0.6285 | 5.606853 | -3.5495 | 0.00042 | 0.001822 | -1.0659 |
| KCNJ4 | -0.5304 | 3.381792 | -3.5489 | 0.00043 | 0.001825 | -1.0679 |
| SPHKAP | -0.5208 | 3.358695 | -3.5359 | 0.00045 | 0.001904 | -1.1126 |
| CARNS1 | -0.6106 | 4.625117 | -3.4688 | 0.00057 | 0.002369 | -1.3412 |
| CPLX2 | -0.657 | 5.510399 | -3.4551 | 0.0006 | 0.002476 | -1.3873 |
| S100A9 | 0.55395 | 4.202804 | 3.44337 | 0.00063 | 0.002572 | -1.4266 |
| CHGA | -0.6152 | 5.197903 | -3.4402 | 0.00063 | 0.002597 | -1.4372 |
| GABRG2 | -0.6266 | 3.597076 | -3.4299 | 0.00066 | 0.002685 | -1.4715 |
| SLC32A1 | -0.5518 | 2.459061 | -3.4299 | 0.00066 | 0.002685 | -1.4716 |
| RGS4 | -0.639 | 3.56924 | -3.4205 | 0.00068 | 0.002768 | -1.5029 |
| GALNT17 | -0.5188 | 3.663615 | -3.4014 | 0.00073 | 0.002941 | -1.5663 |
| AC104072.1 | -0.5284 | 2.699444 | -3.3469 | 0.00088 | 0.003497 | -1.7454 |
| RGS1 | 0.51119 | 4.749352 | 3.29154 | 0.00107 | 0.004145 | -1.9245 |
| HLA-DRB6 | 0.50468 | 3.655266 | 3.28926 | 0.00108 | 0.004175 | -1.9319 |
| AEBP1 | 0.59515 | 5.530841 | 3.25923 | 0.0012 | 0.00458 | -2.0277 |
| MOXD1 | 0.54632 | 1.856609 | 3.21956 | 0.00137 | 0.005166 | -2.1531 |
| MBP | -0.6527 | 7.448929 | -3.2133 | 0.0014 | 0.005265 | -2.1726 |
| MOBP | -0.6087 | 4.674174 | -3.1508 | 0.00173 | 0.006341 | -2.3669 |
| ERMN | -0.5863 | 5.360558 | -3.0899 | 0.00212 | 0.007588 | -2.5528 |
| HPCA | -0.6032 | 4.4652 | -3.0798 | 0.00219 | 0.007816 | -2.5832 |
| MAG | -0.5997 | 6.086627 | -3.0032 | 0.00281 | 0.009754 | -2.811 |
| KLK6 | -0.5418 | 4.799185 | -2.9974 | 0.00287 | 0.009922 | -2.828 |
| HLA-DQA2 | 0.50831 | 2.445144 | 2.88224 | 0.00413 | 0.013655 | -3.1596 |
| MOG | -0.5119 | 5.511717 | -2.7697 | 0.00583 | 0.018435 | -3.4716 |
| VGF | 0.50941 | 4.482159 | 2.63985 | 0.00857 | 0.025752 | -3.8165 |
| AC062021.1 | -0.5365 | 3.592077 | -2.6024 | 0.00955 | 0.028307 | -3.913 |
