# Supplementary material for: CIA‐II is associated with lower‐grade glioma survival and cell proliferation
Source: CNS Neurosci Ther. 2023 Jul 14;30(2):e14340. doi: 10.1111/cns.14340 (PMC10848044; doi:10.1111/cns.14340)
Supplement: Supplementary file 9 — Table S4. [file CNS-30-e14340-s007.docx]

**Table S4.** DEGs in CGGA LGG cohort.

| **id** | **logFC** | **AveExpr** | **t** | **P.Value** | **adj.P.Val** | **B** |
| --- | --- | --- | --- | --- | --- | --- |
| ASF1B | 1.66244 | 2.113359 | 15.8838 | 1.76E-35 | 4.28E-31 | 69.8358 |
| BUB1 | 1.4814 | 1.881746 | 13.7717 | 1.65E-29 | 2.00E-25 | 56.3552 |
| NDC80 | 1.55384 | 1.764727 | 13.589 | 5.46E-29 | 3.87E-25 | 55.1783 |
| RRM2 | 1.96303 | 2.786204 | 13.5655 | 6.37E-29 | 3.87E-25 | 55.0268 |
| KIF20A | 1.50108 | 1.535565 | 13.4282 | 1.57E-28 | 7.64E-25 | 54.1415 |
| MLF1IP | 1.62137 | 2.521636 | 13.3689 | 2.32E-28 | 9.39E-25 | 53.7593 |
| CCNB2 | 1.86089 | 2.231905 | 13.2305 | 5.75E-28 | 1.80E-24 | 52.8661 |
| BIRC5 | 1.88968 | 2.400216 | 13.2259 | 5.93E-28 | 1.80E-24 | 52.8366 |
| UBE2C | 2.19862 | 3.332977 | 13.2029 | 6.90E-28 | 1.86E-24 | 52.688 |
| DLGAP5 | 1.39891 | 1.21058 | 13.1861 | 7.70E-28 | 1.87E-24 | 52.5799 |
| CDCA8 | 1.41707 | 1.66064 | 13.1548 | 9.46E-28 | 2.04E-24 | 52.3781 |
| TPX2 | 1.72475 | 2.843904 | 13.1451 | 1.01E-27 | 2.04E-24 | 52.3152 |
| CDK1 | 1.78186 | 2.86748 | 13.0786 | 1.56E-27 | 2.92E-24 | 51.8859 |
| CDK2 | 1.43769 | 3.781396 | 13.0208 | 2.28E-27 | 3.97E-24 | 51.5129 |
| AURKB | 1.70889 | 2.412418 | 12.9833 | 2.92E-27 | 4.74E-24 | 51.2708 |
| PTTG1 | 1.78679 | 3.909567 | 12.9023 | 4.98E-27 | 7.57E-24 | 50.7478 |
| MYBL2 | 1.97958 | 2.140075 | 12.8594 | 6.60E-27 | 9.23E-24 | 50.471 |
| AURKA | 1.20693 | 2.015732 | 12.8541 | 6.83E-27 | 9.23E-24 | 50.4369 |
| CENPA | 1.45333 | 1.540672 | 12.8427 | 7.36E-27 | 9.28E-24 | 50.3632 |
| NCAPG | 1.43613 | 1.680513 | 12.8369 | 7.65E-27 | 9.28E-24 | 50.3256 |
| CCNA2 | 1.37886 | 2.069102 | 12.8299 | 8.01E-27 | 9.28E-24 | 50.2805 |
| MELK | 1.54506 | 1.716496 | 12.8219 | 8.44E-27 | 9.33E-24 | 50.2291 |
| TROAP | 1.68223 | 2.086473 | 12.6972 | 1.92E-26 | 2.03E-23 | 49.4242 |
| HJURP | 1.44263 | 1.872924 | 12.6811 | 2.13E-26 | 2.16E-23 | 49.3202 |
| KIF2C | 1.59317 | 1.998765 | 12.6504 | 2.61E-26 | 2.54E-23 | 49.1219 |
| CDC6 | 1.16352 | 1.747452 | 12.6358 | 2.87E-26 | 2.68E-23 | 49.0277 |
| TK1 | 1.40912 | 2.303619 | 12.5883 | 3.92E-26 | 3.53E-23 | 48.7212 |
| CLSPN | 1.18049 | 1.333174 | 12.5299 | 5.75E-26 | 5.00E-23 | 48.3442 |
| FOXM1 | 1.7463 | 3.060054 | 12.4269 | 1.13E-25 | 9.50E-23 | 47.6794 |
| CEP55 | 1.28737 | 1.283838 | 12.4042 | 1.31E-25 | 1.07E-22 | 47.5328 |
| ASPM | 1.24376 | 1.088986 | 12.3717 | 1.63E-25 | 1.28E-22 | 47.3236 |
| MKI67 | 1.5096 | 2.14158 | 12.2902 | 2.78E-25 | 2.09E-22 | 46.7974 |
| RAD51AP1 | 1.26324 | 2.402346 | 12.2873 | 2.83E-25 | 2.09E-22 | 46.7787 |
| IQGAP3 | 1.32529 | 1.479577 | 12.2545 | 3.51E-25 | 2.51E-22 | 46.5675 |
| CENPK | 1.37785 | 1.992222 | 12.2261 | 4.23E-25 | 2.94E-22 | 46.3844 |
| FAM111B | 1.22442 | 1.391274 | 12.1933 | 5.25E-25 | 3.55E-22 | 46.1728 |
| FAM64A | 1.83759 | 2.592021 | 12.175 | 5.92E-25 | 3.89E-22 | 46.0553 |
| NUF2 | 1.42129 | 2.186247 | 12.1598 | 6.54E-25 | 4.18E-22 | 45.9567 |
| KIFC1 | 1.68981 | 2.390402 | 12.1561 | 6.70E-25 | 4.18E-22 | 45.9331 |
| KIF14 | 0.89254 | 0.844673 | 12.0973 | 9.86E-25 | 5.99E-22 | 45.5539 |
| KIF23 | 1.40561 | 1.814443 | 12.0928 | 1.02E-24 | 6.02E-22 | 45.5249 |
| GTSE1 | 1.41944 | 1.869195 | 12.0827 | 1.08E-24 | 6.28E-22 | 45.4598 |
| CDCA3 | 1.25361 | 2.80464 | 12.0683 | 1.19E-24 | 6.74E-22 | 45.3675 |
| CENPF | 1.75116 | 2.670919 | 12.0371 | 1.46E-24 | 8.09E-22 | 45.1661 |
| SHCBP1 | 1.16112 | 1.845382 | 12.0332 | 1.50E-24 | 8.11E-22 | 45.141 |
| OIP5 | 1.04746 | 1.483068 | 12.0157 | 1.68E-24 | 8.90E-22 | 45.0285 |
| SGOL2 | 0.87925 | 1.857449 | 12.0122 | 1.72E-24 | 8.91E-22 | 45.0061 |
| SGOL1 | 1.1926 | 1.287471 | 12.0063 | 1.79E-24 | 9.07E-22 | 44.9678 |
| NCAPH | 1.34049 | 2.036158 | 11.9606 | 2.42E-24 | 1.20E-21 | 44.6736 |
| CCNB1 | 1.35562 | 3.403788 | 11.7835 | 7.70E-24 | 3.75E-21 | 43.5342 |
| TCF19 | 0.95908 | 2.325866 | 11.7426 | 1.01E-23 | 4.80E-21 | 43.2713 |
| TOP2A | 2.00603 | 3.175507 | 11.725 | 1.13E-23 | 5.29E-21 | 43.1579 |
| ORC1 | 0.94439 | 0.992368 | 11.7198 | 1.17E-23 | 5.37E-21 | 43.1245 |
| CDC20 | 1.67154 | 2.518984 | 11.7047 | 1.29E-23 | 5.75E-21 | 43.0276 |
| ZNF367 | 1.07655 | 2.005825 | 11.7036 | 1.30E-23 | 5.75E-21 | 43.0204 |
| NUSAP1 | 1.87623 | 3.513829 | 11.6536 | 1.80E-23 | 7.83E-21 | 42.6988 |
| ECT2 | 1.13094 | 2.797805 | 11.6197 | 2.25E-23 | 9.61E-21 | 42.481 |
| PLK1 | 1.29518 | 2.689978 | 11.5843 | 2.84E-23 | 1.19E-20 | 42.2538 |
| RAD51 | 1.13163 | 1.691091 | 11.5322 | 3.99E-23 | 1.64E-20 | 41.9196 |
| PARPBP | 0.91765 | 1.591146 | 11.4481 | 6.91E-23 | 2.76E-20 | 41.3799 |
| CDCA5 | 1.27042 | 2.800487 | 11.4478 | 6.92E-23 | 2.76E-20 | 41.3783 |
| NEIL3 | 0.86291 | 0.739738 | 11.4319 | 7.68E-23 | 3.01E-20 | 41.276 |
| LRR1 | 0.83076 | 2.408896 | 11.4297 | 7.78E-23 | 3.01E-20 | 41.2623 |
| CDC45 | 1.38095 | 1.898529 | 11.3906 | 1.00E-22 | 3.78E-20 | 41.0115 |
| CDCA2 | 1.00616 | 1.007062 | 11.3897 | 1.01E-22 | 3.78E-20 | 41.0061 |
| TACC3 | 1.42397 | 3.244663 | 11.3607 | 1.22E-22 | 4.50E-20 | 40.82 |
| SKA3 | 1.161 | 1.590461 | 11.2963 | 1.86E-22 | 6.74E-20 | 40.4078 |
| TTK | 1.18359 | 1.380913 | 11.2925 | 1.91E-22 | 6.81E-20 | 40.3832 |
| KIF4A | 1.30379 | 1.585638 | 11.2354 | 2.76E-22 | 9.74E-20 | 40.0182 |
| GINS1 | 0.95624 | 2.157287 | 11.2175 | 3.11E-22 | 1.08E-19 | 39.9031 |
| HAUS8 | 0.84401 | 2.787581 | 11.2139 | 3.18E-22 | 1.09E-19 | 39.8801 |
| DEPDC1 | 0.96127 | 0.771468 | 11.1968 | 3.55E-22 | 1.18E-19 | 39.7713 |
| ZWINT | 1.348 | 3.358475 | 11.1968 | 3.55E-22 | 1.18E-19 | 39.7711 |
| KPNA2 | 0.91207 | 5.108523 | 11.1727 | 4.16E-22 | 1.37E-19 | 39.6167 |
| CKAP2L | 1.2153 | 1.465128 | 11.1379 | 5.21E-22 | 1.69E-19 | 39.3944 |
| PRC1 | 1.3206 | 3.826196 | 11.117 | 5.97E-22 | 1.91E-19 | 39.261 |
| CDCA4 | 0.82576 | 2.076554 | 11.0767 | 7.76E-22 | 2.45E-19 | 39.0038 |
| CENPM | 1.20314 | 2.199647 | 11.0405 | 9.81E-22 | 3.06E-19 | 38.773 |
| PCNA | 0.8737 | 5.895946 | 11.0376 | 1.00E-21 | 3.08E-19 | 38.7544 |
| CHEK1 | 1.10658 | 2.66505 | 10.9993 | 1.28E-21 | 3.90E-19 | 38.5096 |
| GAS2L3 | 1.22717 | 1.61266 | 10.985 | 1.41E-21 | 4.23E-19 | 38.4186 |
| GSG2 | 0.85748 | 0.818857 | 10.9821 | 1.43E-21 | 4.25E-19 | 38.4 |
| HMMR | 1.08251 | 1.530698 | 10.9803 | 1.45E-21 | 4.25E-19 | 38.3885 |
| SPC24 | 1.43909 | 1.981125 | 10.9673 | 1.58E-21 | 4.57E-19 | 38.3061 |
| CASC5 | 0.97745 | 1.209233 | 10.9549 | 1.71E-21 | 4.90E-19 | 38.2269 |
| KIF11 | 1.15581 | 2.045272 | 10.9061 | 2.35E-21 | 6.63E-19 | 37.9164 |
| TIMELESS | 1.09521 | 3.212754 | 10.9046 | 2.37E-21 | 6.63E-19 | 37.9064 |
| TRIP13 | 1.01282 | 1.909919 | 10.8952 | 2.52E-21 | 6.97E-19 | 37.8468 |
| WDHD1 | 0.79091 | 1.866431 | 10.8705 | 2.96E-21 | 8.09E-19 | 37.6892 |
| C11orf82 | 0.9442 | 1.468463 | 10.8685 | 3.00E-21 | 8.10E-19 | 37.6767 |
| CDKN3 | 1.21643 | 2.673778 | 10.8476 | 3.43E-21 | 9.12E-19 | 37.5435 |
| BRIP1 | 0.81204 | 0.830166 | 10.8467 | 3.45E-21 | 9.12E-19 | 37.5382 |
| CHEK2 | 0.98827 | 2.080122 | 10.8424 | 3.55E-21 | 9.28E-19 | 37.5109 |
| GINS2 | 1.00556 | 2.09265 | 10.8254 | 3.96E-21 | 1.02E-18 | 37.4026 |
| DTL | 1.44477 | 2.161639 | 10.8163 | 4.20E-21 | 1.08E-18 | 37.3444 |
| MCM10 | 1.15921 | 1.364927 | 10.7194 | 7.86E-21 | 1.99E-18 | 36.7289 |
| KIAA0101 | 1.53179 | 2.523579 | 10.6921 | 9.38E-21 | 2.35E-18 | 36.5557 |
| EZH2 | 1.37495 | 2.994425 | 10.6663 | 1.11E-20 | 2.75E-18 | 36.3917 |
| SMC4 | 1.2565 | 3.626267 | 10.6585 | 1.17E-20 | 2.86E-18 | 36.3428 |
| SKA1 | 0.95355 | 0.996618 | 10.6362 | 1.35E-20 | 3.27E-18 | 36.2009 |
| BUB1B | 1.35231 | 1.980067 | 10.6309 | 1.39E-20 | 3.35E-18 | 36.1676 |
| NEK2 | 1.25624 | 1.545277 | 10.61 | 1.59E-20 | 3.80E-18 | 36.0351 |
| MCM8 | 0.81132 | 1.883764 | 10.6071 | 1.62E-20 | 3.83E-18 | 36.0169 |
| FAM83D | 1.23283 | 1.468539 | 10.5138 | 2.96E-20 | 6.93E-18 | 35.426 |
| CENPE | 1.02617 | 1.164981 | 10.49 | 3.46E-20 | 8.00E-18 | 35.2754 |
| PBK | 1.68252 | 2.456885 | 10.4883 | 3.49E-20 | 8.01E-18 | 35.265 |
| CDC25C | 0.93573 | 1.040391 | 10.4715 | 3.89E-20 | 8.85E-18 | 35.1584 |
| WDR62 | 0.97778 | 1.345126 | 10.4498 | 4.47E-20 | 1.01E-17 | 35.0217 |
| POC1A | 0.89828 | 1.97013 | 10.4411 | 4.73E-20 | 1.06E-17 | 34.9667 |
| STIL | 0.78709 | 1.434597 | 10.4357 | 4.90E-20 | 1.08E-17 | 34.9325 |
| ESCO2 | 1.15776 | 1.444097 | 10.4008 | 6.13E-20 | 1.34E-17 | 34.7119 |
| TMEM194A | 0.76324 | 2.861097 | 10.3477 | 8.62E-20 | 1.87E-17 | 34.3775 |
| LMNB1 | 1.40321 | 3.961558 | 10.3413 | 8.98E-20 | 1.93E-17 | 34.3371 |
| BRCA2 | 0.87624 | 1.218056 | 10.2758 | 1.37E-19 | 2.92E-17 | 33.9243 |
| MAD2L1 | 1.15946 | 3.240887 | 10.2597 | 1.52E-19 | 3.18E-17 | 33.823 |
| MCM3 | 0.79299 | 4.633805 | 10.2596 | 1.52E-19 | 3.18E-17 | 33.8227 |
| PLK4 | 1.00809 | 2.167221 | 10.2528 | 1.58E-19 | 3.29E-17 | 33.78 |
| EXO1 | 1.04712 | 1.388145 | 10.2431 | 1.69E-19 | 3.48E-17 | 33.7187 |
| KIF20B | 0.5972 | 1.513228 | 10.2259 | 1.88E-19 | 3.85E-17 | 33.6104 |
| RNASEH2A | 0.88292 | 4.317264 | 10.192 | 2.34E-19 | 4.74E-17 | 33.3972 |
| PRIM2 | 0.62913 | 2.314933 | 10.19 | 2.37E-19 | 4.76E-17 | 33.3846 |
| RCC2 | 0.91279 | 4.989423 | 10.1711 | 2.67E-19 | 5.33E-17 | 33.2665 |
| ORC6 | 0.98982 | 2.564585 | 10.1506 | 3.05E-19 | 6.03E-17 | 33.1378 |
| ESPL1 | 1.35 | 1.603656 | 10.1436 | 3.19E-19 | 6.25E-17 | 33.0933 |
| IKBIP | 0.99941 | 2.883769 | 10.1391 | 3.28E-19 | 6.38E-17 | 33.0655 |
| FANCD2 | 1.06649 | 2.569767 | 10.1045 | 4.09E-19 | 7.90E-17 | 32.8481 |
| DSN1 | 0.69049 | 2.939204 | 10.0487 | 5.84E-19 | 1.12E-16 | 32.4987 |
| PRR11 | 1.10068 | 2.689446 | 10.0463 | 5.93E-19 | 1.13E-16 | 32.4838 |
| FANCI | 1.23496 | 2.838625 | 10.0407 | 6.15E-19 | 1.16E-16 | 32.4485 |
| DBF4 | 0.96249 | 2.955623 | 9.99146 | 8.41E-19 | 1.57E-16 | 32.1406 |
| E2F2 | 1.01356 | 0.962346 | 9.99016 | 8.48E-19 | 1.58E-16 | 32.1325 |
| POLD1 | 0.81212 | 3.337255 | 9.96883 | 9.72E-19 | 1.79E-16 | 31.9992 |
| DCLRE1B | 0.66351 | 2.530428 | 9.94306 | 1.14E-18 | 2.09E-16 | 31.8382 |
| WEE1 | 1.41357 | 3.29982 | 9.88863 | 1.62E-18 | 2.94E-16 | 31.4986 |
| CENPI | 0.71831 | 1.137142 | 9.88504 | 1.66E-18 | 2.98E-16 | 31.4762 |
| RCC1 | 1.07639 | 3.547855 | 9.88329 | 1.67E-18 | 2.99E-16 | 31.4652 |
| IPO4 | 0.77015 | 3.602232 | 9.8644 | 1.89E-18 | 3.35E-16 | 31.3475 |
| BORA | 0.64446 | 1.737736 | 9.85061 | 2.06E-18 | 3.63E-16 | 31.2616 |
| XRCC2 | 0.81172 | 1.263282 | 9.80507 | 2.75E-18 | 4.81E-16 | 30.9781 |
| E2F7 | 0.80753 | 0.855393 | 9.80387 | 2.77E-18 | 4.81E-16 | 30.9707 |
| CENPN | 0.96861 | 2.939405 | 9.80239 | 2.80E-18 | 4.83E-16 | 30.9615 |
| CENPW | 1.04241 | 2.016842 | 9.80021 | 2.84E-18 | 4.86E-16 | 30.9479 |
| CD276 | 1.16753 | 5.291652 | 9.79168 | 2.99E-18 | 5.09E-16 | 30.8948 |
| MXD3 | 1.13122 | 3.21413 | 9.78503 | 3.12E-18 | 5.25E-16 | 30.8535 |
| PPP4C | 0.6129 | 5.968638 | 9.78451 | 3.13E-18 | 5.25E-16 | 30.8503 |
| TICRR | 0.77499 | 1.380221 | 9.75967 | 3.67E-18 | 6.11E-16 | 30.6958 |
| SNRPB | 0.71492 | 6.118112 | 9.73605 | 4.26E-18 | 7.04E-16 | 30.5491 |
| KDELC1 | 0.76139 | 2.32059 | 9.73315 | 4.34E-18 | 7.13E-16 | 30.5312 |
| STK38 | 0.71859 | 3.491708 | 9.72951 | 4.44E-18 | 7.24E-16 | 30.5085 |
| FBXO5 | 0.93236 | 2.881645 | 9.71118 | 4.98E-18 | 8.08E-16 | 30.3947 |
| CHAF1A | 0.84081 | 3.205365 | 9.70321 | 5.24E-18 | 8.44E-16 | 30.3453 |
| HEATR3 | 0.62563 | 3.111281 | 9.69872 | 5.39E-18 | 8.63E-16 | 30.3175 |
| FRMD8 | 0.69146 | 3.785957 | 9.68919 | 5.73E-18 | 9.10E-16 | 30.2584 |
| HELLS | 0.88915 | 1.731338 | 9.67925 | 6.10E-18 | 9.63E-16 | 30.1967 |
| HNRNPAB | 0.67366 | 6.251443 | 9.67476 | 6.27E-18 | 9.84E-16 | 30.1689 |
| FANCC | 0.80942 | 2.037982 | 9.65946 | 6.91E-18 | 1.08E-15 | 30.0741 |
| SYDE1 | 0.95811 | 3.32908 | 9.65484 | 7.11E-18 | 1.10E-15 | 30.0455 |
| ARHGEF39 | 0.77308 | 1.329968 | 9.64605 | 7.52E-18 | 1.15E-15 | 29.991 |
| FAM126A | 0.9872 | 3.145834 | 9.6457 | 7.54E-18 | 1.15E-15 | 29.9888 |
| BRCA1 | 0.77953 | 2.579187 | 9.62903 | 8.37E-18 | 1.27E-15 | 29.8856 |
| LMNB2 | 0.87973 | 4.322817 | 9.62762 | 8.45E-18 | 1.28E-15 | 29.8769 |
| NCAPD2 | 0.85358 | 4.266964 | 9.62457 | 8.61E-18 | 1.29E-15 | 29.858 |
| TYMS | 1.40279 | 4.352697 | 9.60899 | 9.50E-18 | 1.42E-15 | 29.7616 |
| NCAPG2 | 0.80511 | 3.315877 | 9.59097 | 1.06E-17 | 1.58E-15 | 29.6502 |
| TXNDC5 | 0.70548 | 5.029056 | 9.58401 | 1.11E-17 | 1.64E-15 | 29.6071 |
| NDC1 | 0.66682 | 2.810808 | 9.57514 | 1.18E-17 | 1.72E-15 | 29.5523 |
| CENPL | 0.65775 | 2.364172 | 9.57166 | 1.20E-17 | 1.75E-15 | 29.5308 |
| RFC2 | 0.63693 | 4.531418 | 9.5389 | 1.48E-17 | 2.14E-15 | 29.3285 |
| ZNF90 | 0.7122 | 1.55308 | 9.5041 | 1.84E-17 | 2.65E-15 | 29.1137 |
| DESI2 | 0.68954 | 3.619297 | 9.49738 | 1.92E-17 | 2.73E-15 | 29.0723 |
| DSCC1 | 0.67406 | 2.277743 | 9.49722 | 1.92E-17 | 2.73E-15 | 29.0713 |
| RECQL4 | 0.94274 | 3.19521 | 9.48702 | 2.05E-17 | 2.89E-15 | 29.0084 |
| RFWD3 | 0.66538 | 3.017653 | 9.47991 | 2.14E-17 | 3.01E-15 | 28.9647 |
| POLQ | 0.65769 | 0.642497 | 9.46403 | 2.36E-17 | 3.29E-15 | 28.8668 |
| FANCA | 0.8785 | 2.988082 | 9.46391 | 2.37E-17 | 3.29E-15 | 28.8661 |
| CNIH4 | 0.66284 | 4.454488 | 9.43488 | 2.84E-17 | 3.92E-15 | 28.6874 |
| DEPDC1B | 0.97948 | 1.338878 | 9.42799 | 2.96E-17 | 4.07E-15 | 28.645 |
| NRM | 0.97816 | 3.377153 | 9.42497 | 3.02E-17 | 4.13E-15 | 28.6264 |
| POLA2 | 0.67947 | 3.414583 | 9.42007 | 3.11E-17 | 4.23E-15 | 28.5963 |
| DDOST | 0.67873 | 6.315798 | 9.41002 | 3.32E-17 | 4.48E-15 | 28.5345 |
| SERPINH1 | 1.61155 | 4.631601 | 9.40866 | 3.35E-17 | 4.50E-15 | 28.5261 |
| ARHGAP11A | 1.08362 | 2.354968 | 9.4062 | 3.40E-17 | 4.54E-15 | 28.511 |
| NUP93 | 0.5814 | 4.786076 | 9.38433 | 3.90E-17 | 5.17E-15 | 28.3766 |
| RAD54L | 0.87296 | 1.652756 | 9.38381 | 3.91E-17 | 5.17E-15 | 28.3734 |
| FKBP7 | 0.83338 | 2.993936 | 9.37679 | 4.08E-17 | 5.37E-15 | 28.3303 |
| NRAS | 0.66694 | 4.311792 | 9.36976 | 4.27E-17 | 5.58E-15 | 28.2871 |
| VANGL1 | 0.71587 | 1.615481 | 9.36224 | 4.47E-17 | 5.82E-15 | 28.241 |
| KDELR2 | 0.73243 | 5.655354 | 9.36121 | 4.50E-17 | 5.83E-15 | 28.2346 |
| TONSL | 0.75401 | 1.915821 | 9.33935 | 5.16E-17 | 6.64E-15 | 28.1005 |
| PDIA4 | 1.00256 | 5.490358 | 9.31507 | 6.01E-17 | 7.69E-15 | 27.9517 |
| CENPH | 0.98061 | 2.969659 | 9.31155 | 6.14E-17 | 7.82E-15 | 27.9301 |
| RFC4 | 0.76147 | 4.231918 | 9.30696 | 6.32E-17 | 8.01E-15 | 27.902 |
| FEN1 | 0.66126 | 4.073967 | 9.24948 | 9.05E-17 | 1.13E-14 | 27.5501 |
| SLC30A7 | 0.70735 | 2.167007 | 9.24013 | 9.59E-17 | 1.20E-14 | 27.493 |
| CKS2 | 1.1729 | 4.510552 | 9.2362 | 9.83E-17 | 1.22E-14 | 27.469 |
| EME1 | 0.95104 | 2.194161 | 9.22712 | 1.04E-16 | 1.28E-14 | 27.4135 |
| E2F1 | 0.98394 | 2.851759 | 9.22078 | 1.08E-16 | 1.33E-14 | 27.3747 |
| KIF15 | 1.03354 | 2.091962 | 9.21181 | 1.14E-16 | 1.40E-14 | 27.3199 |
| LEPRE1 | 0.82395 | 3.696698 | 9.20236 | 1.21E-16 | 1.48E-14 | 27.2623 |
| CCDC77 | 0.72657 | 3.102207 | 9.18505 | 1.35E-16 | 1.64E-14 | 27.1567 |
| CASP2 | 0.74309 | 3.329017 | 9.17335 | 1.45E-16 | 1.75E-14 | 27.0853 |
| EML4 | 0.61008 | 3.560108 | 9.15763 | 1.60E-16 | 1.92E-14 | 26.9895 |
| MCM6 | 0.6923 | 3.717492 | 9.15134 | 1.67E-16 | 1.99E-14 | 26.9511 |
| VKORC1 | 0.70503 | 5.717233 | 9.14376 | 1.75E-16 | 2.07E-14 | 26.905 |
| SCLT1 | 0.55127 | 2.432126 | 9.13055 | 1.90E-16 | 2.24E-14 | 26.8245 |
| DPAGT1 | 0.55748 | 4.426746 | 9.09128 | 2.42E-16 | 2.84E-14 | 26.5856 |
| COL4A1 | 2.28125 | 3.979236 | 9.07285 | 2.71E-16 | 3.16E-14 | 26.4736 |
| RAD54B | 0.77919 | 2.01055 | 9.06873 | 2.78E-16 | 3.22E-14 | 26.4485 |
| GPX7 | 0.94851 | 3.378019 | 9.06213 | 2.90E-16 | 3.34E-14 | 26.4085 |
| PLEKHG2 | 1.07835 | 3.856474 | 9.05688 | 2.99E-16 | 3.43E-14 | 26.3765 |
| MGME1 | 0.60673 | 3.535419 | 9.02471 | 3.65E-16 | 4.15E-14 | 26.1813 |
| POLE3 | 0.50647 | 5.44607 | 9.00226 | 4.20E-16 | 4.75E-14 | 26.0453 |
| NADK | 0.68883 | 5.081068 | 8.98626 | 4.63E-16 | 5.22E-14 | 25.9483 |
| HSPG2 | 1.48317 | 3.10715 | 8.98517 | 4.66E-16 | 5.23E-14 | 25.9418 |
| E2F8 | 0.67709 | 0.808284 | 8.97523 | 4.96E-16 | 5.53E-14 | 25.8816 |
| GLA | 0.65557 | 4.02368 | 8.97065 | 5.10E-16 | 5.67E-14 | 25.8539 |
| NUP85 | 0.53248 | 4.684479 | 8.96232 | 5.37E-16 | 5.94E-14 | 25.8034 |
| CDKN2C | 1.38024 | 4.332685 | 8.95433 | 5.64E-16 | 6.21E-14 | 25.7551 |
| PFN1 | 0.76343 | 8.220854 | 8.95053 | 5.78E-16 | 6.33E-14 | 25.7321 |
| MND1 | 0.98835 | 1.639887 | 8.94775 | 5.88E-16 | 6.41E-14 | 25.7153 |
| SLC4A7 | 0.7704 | 2.73269 | 8.93381 | 6.40E-16 | 6.95E-14 | 25.631 |
| KIF18A | 0.85424 | 1.022442 | 8.92907 | 6.59E-16 | 7.13E-14 | 25.6024 |
| CEP135 | 0.68485 | 1.754561 | 8.9263 | 6.71E-16 | 7.22E-14 | 25.5857 |
| TGFB1I1 | 1.13019 | 3.779152 | 8.9128 | 7.29E-16 | 7.81E-14 | 25.5042 |
| KIF18B | 1.20526 | 2.03446 | 8.9105 | 7.39E-16 | 7.89E-14 | 25.4903 |
| CTPS1 | 0.78542 | 3.995908 | 8.90664 | 7.57E-16 | 8.04E-14 | 25.4669 |
| GABPB1 | 0.59895 | 3.235739 | 8.89797 | 7.98E-16 | 8.44E-14 | 25.4146 |
| TMEM8A | 0.58587 | 4.813012 | 8.89219 | 8.27E-16 | 8.71E-14 | 25.3798 |
| PUS7 | 0.68848 | 2.748233 | 8.88571 | 8.61E-16 | 9.03E-14 | 25.3407 |
| ITGA5 | 1.40043 | 3.388858 | 8.8691 | 9.54E-16 | 9.96E-14 | 25.2405 |
| LYPLA1 | 0.62884 | 4.585774 | 8.86802 | 9.60E-16 | 9.98E-14 | 25.234 |
| RECQL | 0.68839 | 4.089409 | 8.86707 | 9.66E-16 | 9.99E-14 | 25.2283 |
| POLE2 | 0.76968 | 1.496783 | 8.85956 | 1.01E-15 | 1.04E-13 | 25.183 |
| SIKE1 | 0.59504 | 4.220471 | 8.84551 | 1.10E-15 | 1.13E-13 | 25.0984 |
| ABCC1 | 0.71724 | 2.926077 | 8.81604 | 1.32E-15 | 1.35E-13 | 24.9211 |
| MTFR2 | 0.59382 | 1.035277 | 8.8067 | 1.40E-15 | 1.42E-13 | 24.8649 |
| BTN2A2 | 0.92063 | 3.196916 | 8.80242 | 1.44E-15 | 1.45E-13 | 24.8392 |
| NXT1 | 0.63515 | 3.758527 | 8.80226 | 1.44E-15 | 1.45E-13 | 24.8382 |
| SDC1 | 1.05597 | 1.609251 | 8.79326 | 1.52E-15 | 1.53E-13 | 24.7842 |
| KNTC1 | 0.88581 | 3.004474 | 8.79277 | 1.52E-15 | 1.53E-13 | 24.7812 |
| RPN2 | 0.58893 | 6.700716 | 8.77653 | 1.68E-15 | 1.68E-13 | 24.6837 |
| CCNF | 0.63009 | 1.858742 | 8.77501 | 1.70E-15 | 1.69E-13 | 24.6745 |
| RNF122 | 1.04755 | 2.946433 | 8.75882 | 1.88E-15 | 1.85E-13 | 24.5773 |
| CDC7 | 0.78476 | 2.918265 | 8.75738 | 1.89E-15 | 1.86E-13 | 24.5687 |
| RMI2 | 0.81891 | 2.107125 | 8.75372 | 1.94E-15 | 1.90E-13 | 24.5467 |
| GJC1 | 1.12357 | 2.315505 | 8.75038 | 1.98E-15 | 1.93E-13 | 24.5267 |
| PMM2 | 0.64671 | 3.146344 | 8.74517 | 2.04E-15 | 1.98E-13 | 24.4955 |
| C1orf112 | 0.62991 | 2.207691 | 8.73949 | 2.11E-15 | 2.05E-13 | 24.4614 |
| TRAIP | 0.84079 | 2.152771 | 8.73603 | 2.16E-15 | 2.08E-13 | 24.4407 |
| C5orf34 | 0.66037 | 1.430187 | 8.73309 | 2.20E-15 | 2.11E-13 | 24.423 |
| UBE2T | 1.01977 | 3.341559 | 8.73272 | 2.20E-15 | 2.11E-13 | 24.4209 |
| C19orf48 | 0.68607 | 4.232348 | 8.726 | 2.29E-15 | 2.19E-13 | 24.3806 |
| TMEM106C | 0.81129 | 5.39636 | 8.7254 | 2.30E-15 | 2.19E-13 | 24.377 |
| PGM2 | 0.73125 | 3.735173 | 8.7211 | 2.36E-15 | 2.24E-13 | 24.3512 |
| RBBP8 | 0.78511 | 3.697503 | 8.72013 | 2.38E-15 | 2.24E-13 | 24.3454 |
| BARD1 | 0.94762 | 2.401109 | 8.71492 | 2.45E-15 | 2.30E-13 | 24.3142 |
| CANT1 | 0.61111 | 4.307974 | 8.71215 | 2.50E-15 | 2.33E-13 | 24.2976 |
| AK2 | 0.69216 | 5.99171 | 8.68314 | 2.98E-15 | 2.77E-13 | 24.124 |
| TUBB | 0.69749 | 8.30386 | 8.67408 | 3.15E-15 | 2.91E-13 | 24.0699 |
| NOP2 | 0.71477 | 4.706653 | 8.67046 | 3.22E-15 | 2.97E-13 | 24.0482 |
| ZWILCH | 0.67972 | 2.562058 | 8.65327 | 3.57E-15 | 3.28E-13 | 23.9455 |
| SMARCE1 | 0.6602 | 6.330756 | 8.64825 | 3.69E-15 | 3.37E-13 | 23.9155 |
| KIAA1524 | 0.67538 | 1.990047 | 8.64212 | 3.83E-15 | 3.49E-13 | 23.8789 |
| OSTC | 0.79618 | 5.183605 | 8.6315 | 4.08E-15 | 3.70E-13 | 23.8156 |
| DTYMK | 0.64254 | 4.50742 | 8.61972 | 4.38E-15 | 3.95E-13 | 23.7453 |
| CTNNAL1 | 0.62299 | 3.608572 | 8.61564 | 4.49E-15 | 4.03E-13 | 23.721 |
| CALU | 1.06622 | 5.676656 | 8.61146 | 4.61E-15 | 4.12E-13 | 23.6961 |
| NOL11 | 0.57311 | 4.416431 | 8.60747 | 4.72E-15 | 4.21E-13 | 23.6722 |
| CKLF | 0.6695 | 5.413849 | 8.59539 | 5.08E-15 | 4.50E-13 | 23.6003 |
| ITPRIPL1 | 1.0041 | 1.683942 | 8.59378 | 5.13E-15 | 4.52E-13 | 23.5907 |
| LOXL2 | 1.49604 | 2.833214 | 8.59316 | 5.15E-15 | 4.53E-13 | 23.587 |
| TPM4 | 0.90513 | 6.885808 | 8.56631 | 6.07E-15 | 5.31E-13 | 23.4272 |
| DPH2 | 0.55473 | 4.243651 | 8.56086 | 6.27E-15 | 5.47E-13 | 23.3948 |
| CARHSP1 | 0.73774 | 5.358336 | 8.55967 | 6.32E-15 | 5.49E-13 | 23.3877 |
| BTN2A3P | 0.76876 | 1.50149 | 8.55881 | 6.35E-15 | 5.50E-13 | 23.3825 |
| CHPF2 | 0.73138 | 3.944642 | 8.55759 | 6.40E-15 | 5.52E-13 | 23.3753 |
| ADAM9 | 0.89348 | 5.551352 | 8.55606 | 6.46E-15 | 5.55E-13 | 23.3662 |
| RPF1 | 0.57933 | 4.62315 | 8.54926 | 6.73E-15 | 5.76E-13 | 23.3258 |
| XRN2 | 0.64705 | 4.85163 | 8.54548 | 6.88E-15 | 5.88E-13 | 23.3033 |
| ZMYM1 | 0.61767 | 2.290377 | 8.53987 | 7.12E-15 | 6.06E-13 | 23.27 |
| COL4A2 | 1.97921 | 4.250267 | 8.53571 | 7.30E-15 | 6.19E-13 | 23.2453 |
| SEC61A1 | 0.67683 | 6.654414 | 8.52833 | 7.64E-15 | 6.42E-13 | 23.2014 |
| PDIA5 | 1.09238 | 2.448531 | 8.52801 | 7.65E-15 | 6.42E-13 | 23.1995 |
| DAXX | 0.54134 | 5.314428 | 8.52799 | 7.65E-15 | 6.42E-13 | 23.1994 |
| NIP7 | 0.54474 | 3.75443 | 8.52736 | 7.68E-15 | 6.42E-13 | 23.1957 |
| BCL10 | 0.51559 | 2.514112 | 8.5249 | 7.80E-15 | 6.49E-13 | 23.181 |
| MCM2 | 1.05092 | 3.41881 | 8.5245 | 7.82E-15 | 6.49E-13 | 23.1787 |
| RACGAP1 | 0.8588 | 3.8874 | 8.51778 | 8.14E-15 | 6.74E-13 | 23.1388 |
| P4HB | 0.72317 | 7.561599 | 8.49159 | 9.54E-15 | 7.85E-13 | 22.9834 |
| TARBP2 | 0.57289 | 4.63584 | 8.49089 | 9.58E-15 | 7.85E-13 | 22.9793 |
| C19orf40 | 0.58373 | 1.375548 | 8.49088 | 9.58E-15 | 7.85E-13 | 22.9792 |
| CMTM6 | 0.75277 | 4.104453 | 8.48547 | 9.90E-15 | 8.08E-13 | 22.9471 |
| CCDC15 | 0.56734 | 1.361933 | 8.4753 | 1.05E-14 | 8.54E-13 | 22.8869 |
| DDX39A | 0.66222 | 5.708854 | 8.47059 | 1.08E-14 | 8.76E-13 | 22.859 |
| KIRREL | 0.95414 | 2.87079 | 8.46387 | 1.13E-14 | 9.09E-13 | 22.8192 |
| EXOSC3 | 0.50475 | 3.988493 | 8.45509 | 1.19E-14 | 9.55E-13 | 22.7672 |
| IL10RB | 0.66767 | 3.870337 | 8.44253 | 1.28E-14 | 1.02E-12 | 22.6929 |
| HAUS5 | 0.76862 | 3.606391 | 8.44124 | 1.29E-14 | 1.03E-12 | 22.6853 |
| CLP1 | 0.5136 | 3.006395 | 8.44003 | 1.30E-14 | 1.03E-12 | 22.6781 |
| SZRD1 | 0.68709 | 5.625992 | 8.43806 | 1.32E-14 | 1.04E-12 | 22.6665 |
| IGF2BP3 | 1.46632 | 1.341942 | 8.43591 | 1.34E-14 | 1.05E-12 | 22.6538 |
| MEX3D | 0.69404 | 3.792317 | 8.42622 | 1.42E-14 | 1.11E-12 | 22.5965 |
| DEK | 0.64666 | 5.656803 | 8.42279 | 1.45E-14 | 1.13E-12 | 22.5762 |
| ELAVL1 | 0.51028 | 4.964889 | 8.40707 | 1.59E-14 | 1.23E-12 | 22.4834 |
| ATAD5 | 0.51546 | 1.148395 | 8.40255 | 1.63E-14 | 1.26E-12 | 22.4567 |
| PRMT6 | 0.55779 | 3.407992 | 8.40219 | 1.64E-14 | 1.26E-12 | 22.4546 |
| RNPEP | 0.53328 | 4.549323 | 8.39809 | 1.68E-14 | 1.29E-12 | 22.4304 |
| LRRC42 | 0.7213 | 3.968097 | 8.38824 | 1.78E-14 | 1.36E-12 | 22.3722 |
| NR2C2AP | 0.55352 | 3.959623 | 8.37368 | 1.94E-14 | 1.48E-12 | 22.2864 |
| ITGB1 | 0.98546 | 6.252144 | 8.37258 | 1.96E-14 | 1.48E-12 | 22.2799 |
| KSR2 | -0.8932 | 1.336191 | -8.3724 | 1.96E-14 | 1.48E-12 | 22.2786 |
| NKIRAS2 | 0.71725 | 4.820828 | 8.36664 | 2.03E-14 | 1.53E-12 | 22.2449 |
| RAN | 0.51946 | 7.876165 | 8.36307 | 2.07E-14 | 1.56E-12 | 22.2239 |
| RFC3 | 0.65364 | 2.838114 | 8.35565 | 2.17E-14 | 1.63E-12 | 22.1801 |
| KDM4A | 0.64415 | 4.17879 | 8.34879 | 2.26E-14 | 1.68E-12 | 22.1398 |
| NOP16 | 0.50773 | 4.120101 | 8.34853 | 2.26E-14 | 1.68E-12 | 22.1382 |
| TFPI | 1.68783 | 2.868079 | 8.33969 | 2.38E-14 | 1.77E-12 | 22.0862 |
| DDX20 | 0.574 | 2.693814 | 8.33253 | 2.49E-14 | 1.84E-12 | 22.044 |
| STT3A | 0.60787 | 5.494604 | 8.33234 | 2.49E-14 | 1.84E-12 | 22.0429 |
| MAD2L2 | 0.83572 | 5.6487 | 8.31243 | 2.81E-14 | 2.05E-12 | 21.9259 |
| MCM5 | 0.75377 | 4.402534 | 8.30941 | 2.86E-14 | 2.08E-12 | 21.9081 |
| ALG3 | 0.56356 | 4.451168 | 8.30905 | 2.87E-14 | 2.08E-12 | 21.906 |
| PTBP1 | 0.76032 | 6.316339 | 8.30822 | 2.88E-14 | 2.08E-12 | 21.9011 |
| DPP9 | 0.50679 | 4.134856 | 8.30151 | 3.00E-14 | 2.16E-12 | 21.8617 |
| RPS2 | 0.76029 | 10.04481 | 8.2954 | 3.11E-14 | 2.23E-12 | 21.8258 |
| POLD3 | 0.50821 | 3.214827 | 8.28622 | 3.29E-14 | 2.35E-12 | 21.7719 |
| ITGB3BP | 0.8071 | 3.48047 | 8.28392 | 3.33E-14 | 2.37E-12 | 21.7584 |
| TMEM39B | 0.52932 | 3.46569 | 8.28386 | 3.33E-14 | 2.37E-12 | 21.7581 |
| E2F3 | 0.62423 | 3.229181 | 8.27916 | 3.43E-14 | 2.43E-12 | 21.7305 |
| RTKN2 | 0.77939 | 1.315996 | 8.27368 | 3.54E-14 | 2.51E-12 | 21.6983 |
| H2AFZ | 0.65131 | 7.182272 | 8.26906 | 3.64E-14 | 2.57E-12 | 21.6712 |
| TMPO | 0.72191 | 4.820528 | 8.2666 | 3.70E-14 | 2.60E-12 | 21.6568 |
| CASP6 | 0.77827 | 2.911198 | 8.26103 | 3.82E-14 | 2.68E-12 | 21.6242 |
| FNDC3B | 0.88942 | 3.263736 | 8.21848 | 4.93E-14 | 3.43E-12 | 21.375 |
| ODF2 | 0.61271 | 4.393807 | 8.21494 | 5.04E-14 | 3.48E-12 | 21.3543 |
| TXLNA | 0.73501 | 4.166785 | 8.21475 | 5.04E-14 | 3.48E-12 | 21.3532 |
| CCNJL | 0.67258 | 1.370323 | 8.21175 | 5.13E-14 | 3.54E-12 | 21.3356 |
| SEC24D | 0.8318 | 3.009692 | 8.20211 | 5.44E-14 | 3.73E-12 | 21.2793 |
| YTHDF2 | 0.53133 | 4.970005 | 8.20099 | 5.47E-14 | 3.73E-12 | 21.2727 |
| FLNA | 1.28865 | 6.705289 | 8.19656 | 5.62E-14 | 3.82E-12 | 21.2469 |
| YBX1 | 0.8056 | 8.488766 | 8.1909 | 5.81E-14 | 3.94E-12 | 21.2138 |
| SURF4 | 0.51626 | 6.170356 | 8.1775 | 6.30E-14 | 4.25E-12 | 21.1356 |
| MIS18A | 0.59799 | 2.948324 | 8.16424 | 6.81E-14 | 4.58E-12 | 21.0582 |
| ATAD2 | 0.69833 | 3.040569 | 8.15918 | 7.02E-14 | 4.69E-12 | 21.0287 |
| GLB1 | 0.56328 | 4.371636 | 8.15844 | 7.05E-14 | 4.70E-12 | 21.0244 |
| TRAM2 | 0.82498 | 2.231639 | 8.15311 | 7.28E-14 | 4.84E-12 | 20.9933 |
| ALYREF | 0.59177 | 5.717307 | 8.14949 | 7.44E-14 | 4.93E-12 | 20.9722 |
| KNSTRN | 0.62187 | 3.648957 | 8.13442 | 8.14E-14 | 5.34E-12 | 20.8844 |
| UBALD2 | 0.76139 | 5.641081 | 8.13323 | 8.20E-14 | 5.36E-12 | 20.8775 |
| RBM38 | 0.5951 | 4.337288 | 8.12991 | 8.36E-14 | 5.45E-12 | 20.8582 |
| PHACTR4 | 0.60362 | 3.461321 | 8.12765 | 8.47E-14 | 5.51E-12 | 20.845 |
| NOL8 | 0.5057 | 4.316009 | 8.12624 | 8.54E-14 | 5.54E-12 | 20.8368 |
| RBBP4 | 0.60653 | 6.655534 | 8.11764 | 8.99E-14 | 5.82E-12 | 20.7868 |
| SLC26A2 | 0.73598 | 2.063063 | 8.10884 | 9.48E-14 | 6.10E-12 | 20.7356 |
| ERCC6L | 0.63784 | 0.64844 | 8.10141 | 9.90E-14 | 6.36E-12 | 20.6924 |
| CKAP4 | 0.62855 | 4.68493 | 8.10015 | 9.98E-14 | 6.38E-12 | 20.685 |
| WDR77 | 0.56184 | 4.31573 | 8.0994 | 1.00E-13 | 6.38E-12 | 20.6807 |
| NOP10 | 0.54965 | 6.705044 | 8.09347 | 1.04E-13 | 6.58E-12 | 20.6462 |
| CD93 | 1.41504 | 2.667392 | 8.09111 | 1.05E-13 | 6.65E-12 | 20.6325 |
| GNG5 | 0.98799 | 6.15655 | 8.08434 | 1.10E-13 | 6.91E-12 | 20.5932 |
| FAM111A | 0.89147 | 3.602719 | 8.08156 | 1.11E-13 | 6.98E-12 | 20.5771 |
| C19orf10 | 0.54394 | 6.030098 | 8.07112 | 1.19E-13 | 7.39E-12 | 20.5164 |
| CDH11 | 0.96253 | 5.146812 | 8.05015 | 1.34E-13 | 8.35E-12 | 20.3948 |
| SERBP1 | 0.6062 | 6.786577 | 8.03872 | 1.44E-13 | 8.91E-12 | 20.3286 |
| QTRTD1 | 0.5767 | 3.048624 | 8.03818 | 1.44E-13 | 8.92E-12 | 20.3254 |
| GBE1 | 0.88036 | 3.870017 | 8.02989 | 1.51E-13 | 9.34E-12 | 20.2775 |
| TRIM24 | 0.77991 | 4.473858 | 8.02543 | 1.55E-13 | 9.55E-12 | 20.2516 |
| EFTUD2 | 0.54389 | 5.757989 | 8.02525 | 1.56E-13 | 9.55E-12 | 20.2506 |
| FAM72D | 0.54922 | 0.995469 | 8.02205 | 1.59E-13 | 9.71E-12 | 20.2321 |
| MMS22L | 0.5511 | 1.892771 | 8.01516 | 1.65E-13 | 1.00E-11 | 20.1922 |
| FBLIM1 | 1.22749 | 2.374947 | 8.00166 | 1.79E-13 | 1.08E-11 | 20.1141 |
| DYRK2 | 0.56267 | 2.983662 | 8.00151 | 1.79E-13 | 1.08E-11 | 20.1133 |
| RBM15 | 0.54105 | 2.205873 | 7.99948 | 1.81E-13 | 1.09E-11 | 20.1015 |
| TCEB3 | 0.56491 | 4.150537 | 7.99022 | 1.91E-13 | 1.15E-11 | 20.048 |
| MYL6 | 0.6333 | 9.823688 | 7.98256 | 2.00E-13 | 1.20E-11 | 20.0038 |
| ALDH2 | -0.9245 | 7.579409 | -7.9812 | 2.02E-13 | 1.20E-11 | 19.996 |
| ST14 | 1.09622 | 1.784464 | 7.97746 | 2.06E-13 | 1.22E-11 | 19.9743 |
| SERPINB8 | 0.87485 | 2.771789 | 7.9774 | 2.06E-13 | 1.22E-11 | 19.974 |
| CYB5RL | 0.54779 | 2.080218 | 7.97579 | 2.08E-13 | 1.23E-11 | 19.9647 |
| DNMT1 | 0.60971 | 5.057603 | 7.96982 | 2.16E-13 | 1.27E-11 | 19.9302 |
| BAZ1A | 0.72387 | 3.413813 | 7.96828 | 2.18E-13 | 1.28E-11 | 19.9213 |
| ORAI1 | 0.5993 | 2.88385 | 7.96381 | 2.24E-13 | 1.31E-11 | 19.8955 |
| GNAI3 | 0.71949 | 4.94417 | 7.95824 | 2.31E-13 | 1.35E-11 | 19.8634 |
| COL3A1 | 2.17738 | 3.101433 | 7.9577 | 2.32E-13 | 1.35E-11 | 19.8603 |
| HMGB2 | 0.81276 | 6.0968 | 7.94557 | 2.49E-13 | 1.44E-11 | 19.7903 |
| NAA38 | 0.64564 | 4.580075 | 7.9438 | 2.52E-13 | 1.45E-11 | 19.7802 |
| HM13 | 0.53638 | 6.468462 | 7.9421 | 2.54E-13 | 1.46E-11 | 19.7703 |
| ZNF436 | 0.81375 | 3.884556 | 7.93697 | 2.62E-13 | 1.51E-11 | 19.7408 |
| HAS3 | 0.63927 | 1.274392 | 7.93362 | 2.67E-13 | 1.53E-11 | 19.7215 |
| WDR1 | 0.62528 | 6.718216 | 7.93237 | 2.69E-13 | 1.54E-11 | 19.7143 |
| DNAJB11 | 0.55336 | 5.738716 | 7.92848 | 2.75E-13 | 1.57E-11 | 19.692 |
| WDR34 | 0.62535 | 4.941469 | 7.92307 | 2.84E-13 | 1.61E-11 | 19.6608 |
| PUSL1 | 0.5658 | 3.369711 | 7.92279 | 2.85E-13 | 1.61E-11 | 19.6592 |
| ARPC5 | 0.67693 | 6.335345 | 7.92206 | 2.86E-13 | 1.62E-11 | 19.655 |
| CBX7 | -0.8439 | 4.83926 | -7.91 | 3.07E-13 | 1.73E-11 | 19.5858 |
| PGD | 0.59637 | 6.31083 | 7.90535 | 3.15E-13 | 1.77E-11 | 19.5589 |
| GINS4 | 0.68801 | 1.959586 | 7.90286 | 3.20E-13 | 1.79E-11 | 19.5445 |
| BZW1 | 0.50651 | 6.678261 | 7.89814 | 3.29E-13 | 1.83E-11 | 19.5174 |
| COLGALT1 | 0.73378 | 4.772132 | 7.89102 | 3.43E-13 | 1.91E-11 | 19.4765 |
| PHC2 | 0.67198 | 6.493209 | 7.89056 | 3.44E-13 | 1.91E-11 | 19.4738 |
| NFATC3 | 0.50714 | 3.372076 | 7.88574 | 3.54E-13 | 1.96E-11 | 19.4462 |
| RAD18 | 0.63594 | 2.241755 | 7.88213 | 3.62E-13 | 1.99E-11 | 19.4254 |
| FN1 | 1.44704 | 6.861045 | 7.86252 | 4.06E-13 | 2.23E-11 | 19.3129 |
| C16orf59 | 0.79565 | 2.239477 | 7.86045 | 4.11E-13 | 2.25E-11 | 19.3011 |
| KDM1A | 0.72404 | 5.475129 | 7.85323 | 4.28E-13 | 2.34E-11 | 19.2597 |
| HAUS1 | 0.62317 | 4.208888 | 7.83188 | 4.85E-13 | 2.63E-11 | 19.1373 |
| LAMC1 | 1.16469 | 3.873673 | 7.82137 | 5.16E-13 | 2.78E-11 | 19.0772 |
| TGFBI | 1.64454 | 4.714059 | 7.81921 | 5.23E-13 | 2.81E-11 | 19.0648 |
| SRSF10 | 0.53185 | 5.516552 | 7.81526 | 5.35E-13 | 2.87E-11 | 19.0422 |
| TRAF7 | 0.52466 | 4.936912 | 7.80913 | 5.54E-13 | 2.97E-11 | 19.0072 |
| LMAN2 | 0.55624 | 6.232158 | 7.80284 | 5.75E-13 | 3.07E-11 | 18.9713 |
| CCDC18 | 0.67335 | 1.64389 | 7.79787 | 5.92E-13 | 3.14E-11 | 18.9429 |
| MRPL42 | 0.56446 | 4.767315 | 7.79627 | 5.98E-13 | 3.16E-11 | 18.9337 |
| CDC25A | 0.77392 | 1.57484 | 7.79115 | 6.16E-13 | 3.23E-11 | 18.9044 |
| PLOD1 | 0.90909 | 5.384702 | 7.78056 | 6.55E-13 | 3.43E-11 | 18.844 |
| SLC39A1 | 0.70198 | 5.685618 | 7.77326 | 6.84E-13 | 3.57E-11 | 18.8024 |
| HIST1H2AG | 0.57087 | 0.70574 | 7.77296 | 6.85E-13 | 3.57E-11 | 18.8007 |
| ERI1 | 0.62426 | 2.873087 | 7.77245 | 6.87E-13 | 3.57E-11 | 18.7977 |
| FSTL1 | 1.26911 | 4.991492 | 7.77094 | 6.93E-13 | 3.59E-11 | 18.7891 |
| NUP62 | 0.58994 | 4.72851 | 7.76781 | 7.06E-13 | 3.65E-11 | 18.7713 |
| REEP4 | 0.72857 | 3.36644 | 7.76512 | 7.17E-13 | 3.70E-11 | 18.7559 |
| MSANTD3 | 0.56051 | 4.356619 | 7.76063 | 7.36E-13 | 3.79E-11 | 18.7303 |
| IQGAP2 | 1.26719 | 2.863809 | 7.75281 | 7.70E-13 | 3.95E-11 | 18.6858 |
| BYSL | 0.55159 | 3.829866 | 7.74676 | 7.98E-13 | 4.08E-11 | 18.6513 |
| ACTL6A | 0.65535 | 4.701625 | 7.7413 | 8.24E-13 | 4.18E-11 | 18.6202 |
| APOL4 | 1.49655 | 2.238833 | 7.74026 | 8.29E-13 | 4.19E-11 | 18.6143 |
| HLF | -1.1271 | 3.941616 | -7.738 | 8.40E-13 | 4.23E-11 | 18.6015 |
| PRDX4 | 0.72964 | 5.833065 | 7.73584 | 8.50E-13 | 4.27E-11 | 18.5891 |
| SKP2 | 0.53243 | 2.880153 | 7.72703 | 8.95E-13 | 4.46E-11 | 18.539 |
| CKAP2 | 0.76272 | 3.859183 | 7.72629 | 8.99E-13 | 4.47E-11 | 18.5348 |
| SBNO2 | 0.72777 | 3.576173 | 7.72587 | 9.01E-13 | 4.47E-11 | 18.5324 |
| TMEM256-PLSCR3 | 0.57683 | 4.330703 | 7.72442 | 9.09E-13 | 4.49E-11 | 18.5241 |
| FBXW9 | 0.5255 | 3.173764 | 7.7226 | 9.18E-13 | 4.53E-11 | 18.5138 |
| FADD | 0.597 | 3.796286 | 7.71967 | 9.34E-13 | 4.60E-11 | 18.4972 |
| SDF4 | 0.54698 | 6.323754 | 7.715 | 9.60E-13 | 4.72E-11 | 18.4706 |
| REXO2 | 0.62844 | 5.073833 | 7.71152 | 9.79E-13 | 4.80E-11 | 18.4508 |
| HPCAL4 | -1.7687 | 4.209952 | -7.7107 | 9.84E-13 | 4.81E-11 | 18.4464 |
| SAMD9 | 0.86597 | 2.323477 | 7.70982 | 9.89E-13 | 4.83E-11 | 18.4411 |
| ACTR3 | 0.59296 | 6.349234 | 7.70932 | 9.92E-13 | 4.84E-11 | 18.4383 |
| ALG6 | 0.58582 | 2.958352 | 7.70623 | 1.01E-12 | 4.91E-11 | 18.4207 |
| F2R | 1.18863 | 4.555331 | 7.7056 | 1.01E-12 | 4.92E-11 | 18.4172 |
| PDLIM7 | 0.91901 | 4.812212 | 7.70544 | 1.01E-12 | 4.92E-11 | 18.4163 |
| AJUBA | 0.78529 | 1.906967 | 7.69891 | 1.05E-12 | 5.10E-11 | 18.3792 |
| H2AFX | 0.67403 | 5.77387 | 7.69522 | 1.08E-12 | 5.19E-11 | 18.3582 |
| GDAP2 | 0.53971 | 2.367338 | 7.69503 | 1.08E-12 | 5.19E-11 | 18.3572 |
| SENP1 | 0.50432 | 2.805942 | 7.68881 | 1.12E-12 | 5.35E-11 | 18.3219 |
| TGFBR1 | 0.82725 | 4.162221 | 7.68829 | 1.12E-12 | 5.35E-11 | 18.3189 |
| COL5A2 | 1.4478 | 2.832701 | 7.68803 | 1.12E-12 | 5.35E-11 | 18.3174 |
| NOX4 | 0.91062 | 1.253803 | 7.68465 | 1.14E-12 | 5.45E-11 | 18.2982 |
| DCAF13 | 0.608 | 4.739606 | 7.67933 | 1.18E-12 | 5.61E-11 | 18.2681 |
| MASTL | 0.53423 | 2.312392 | 7.67585 | 1.20E-12 | 5.70E-11 | 18.2483 |
| SF3A3 | 0.61113 | 5.58087 | 7.67373 | 1.22E-12 | 5.75E-11 | 18.2363 |
| WDR76 | 0.78316 | 2.44769 | 7.66861 | 1.26E-12 | 5.90E-11 | 18.2073 |
| SMC5 | 0.68777 | 3.595266 | 7.66674 | 1.27E-12 | 5.93E-11 | 18.1967 |
| ARHGAP11B | 0.76914 | 1.633649 | 7.66109 | 1.31E-12 | 6.12E-11 | 18.1647 |
| LYPLA2 | 0.56326 | 4.782177 | 7.65735 | 1.34E-12 | 6.24E-11 | 18.1435 |
| BTG3 | 0.75096 | 4.700308 | 7.65602 | 1.35E-12 | 6.27E-11 | 18.1359 |
| RP11-285F16.1 | -1.0295 | 1.931954 | -7.6445 | 1.45E-12 | 6.68E-11 | 18.0705 |
| RIT1 | 0.6117 | 4.260233 | 7.6415 | 1.47E-12 | 6.79E-11 | 18.0538 |
| FEM1C | 0.5877 | 3.716289 | 7.63148 | 1.56E-12 | 7.18E-11 | 17.9971 |
| TTF2 | 0.66245 | 2.437919 | 7.62822 | 1.59E-12 | 7.27E-11 | 17.9786 |
| C8orf76 | 0.50804 | 4.024013 | 7.62254 | 1.64E-12 | 7.50E-11 | 17.9466 |
| FZD2 | 0.78114 | 1.896301 | 7.62099 | 1.66E-12 | 7.55E-11 | 17.9378 |
| DKC1 | 0.51147 | 4.84486 | 7.61117 | 1.75E-12 | 7.97E-11 | 17.8823 |
| MGAT2 | 0.6066 | 3.791728 | 7.60765 | 1.79E-12 | 8.10E-11 | 17.8624 |
| ETV6 | 0.58863 | 2.631203 | 7.6048 | 1.82E-12 | 8.22E-11 | 17.8464 |
| HIST1H3H | 0.75343 | 0.806826 | 7.60383 | 1.83E-12 | 8.25E-11 | 17.8409 |
| CAPN5 | 1.02373 | 4.430068 | 7.59129 | 1.97E-12 | 8.84E-11 | 17.7701 |
| HN1L | 0.7469 | 3.904409 | 7.59053 | 1.97E-12 | 8.86E-11 | 17.7658 |
| TMEM45A | 1.0408 | 3.37967 | 7.58106 | 2.08E-12 | 9.32E-11 | 17.7125 |
| ITGA4 | 0.99163 | 1.75956 | 7.56972 | 2.23E-12 | 9.91E-11 | 17.6485 |
| CCDC102A | 0.68933 | 2.219284 | 7.56903 | 2.23E-12 | 9.94E-11 | 17.6446 |
| MMP11 | 1.08123 | 1.569522 | 7.56547 | 2.28E-12 | 1.01E-10 | 17.6246 |
| PKN3 | 0.53596 | 1.829561 | 7.56204 | 2.33E-12 | 1.03E-10 | 17.6053 |
| DCBLD2 | 0.78315 | 3.128492 | 7.55798 | 2.38E-12 | 1.05E-10 | 17.5824 |
| RPAP2 | 0.58139 | 2.932748 | 7.55626 | 2.41E-12 | 1.06E-10 | 17.5727 |
| DPEP1 | 1.2428 | 1.355057 | 7.55526 | 2.42E-12 | 1.06E-10 | 17.5671 |
| GNL2 | 0.6447 | 4.512314 | 7.55129 | 2.47E-12 | 1.08E-10 | 17.5448 |
| ZNF124 | 0.50432 | 2.28961 | 7.55002 | 2.49E-12 | 1.09E-10 | 17.5377 |
| LSM7 | 0.57645 | 6.823961 | 7.54919 | 2.50E-12 | 1.09E-10 | 17.533 |
| NUP205 | 0.57833 | 4.442065 | 7.54362 | 2.59E-12 | 1.13E-10 | 17.5016 |
| FAM131C | -0.9543 | 2.09796 | -7.5353 | 2.71E-12 | 1.18E-10 | 17.4549 |
| SMC2 | 0.58515 | 3.498128 | 7.53473 | 2.72E-12 | 1.18E-10 | 17.4517 |
| CDCA7L | 1.02527 | 3.775344 | 7.53293 | 2.75E-12 | 1.19E-10 | 17.4416 |
| GEN1 | 0.65064 | 1.735118 | 7.53116 | 2.78E-12 | 1.20E-10 | 17.4316 |
| MMP14 | 1.40004 | 4.577119 | 7.53099 | 2.78E-12 | 1.20E-10 | 17.4307 |
| CASP3 | 0.69976 | 4.230981 | 7.52351 | 2.90E-12 | 1.25E-10 | 17.3887 |
| HLX | 0.84627 | 2.252427 | 7.51941 | 2.97E-12 | 1.28E-10 | 17.3656 |
| C17orf53 | 0.68472 | 1.401339 | 7.51775 | 3.00E-12 | 1.29E-10 | 17.3563 |
| VMP1 | 0.74768 | 5.685894 | 7.51585 | 3.03E-12 | 1.30E-10 | 17.3456 |
| SNRNP40 | 0.54561 | 4.61845 | 7.51175 | 3.11E-12 | 1.33E-10 | 17.3227 |
| KDELR1 | 0.63228 | 5.918448 | 7.51125 | 3.12E-12 | 1.33E-10 | 17.3199 |
| MTBP | 0.55192 | 1.813215 | 7.50897 | 3.16E-12 | 1.34E-10 | 17.3071 |
| C8orf4 | 1.50847 | 3.553357 | 7.50688 | 3.19E-12 | 1.36E-10 | 17.2954 |
| ETNPPL | -2.1585 | 5.699047 | -7.505 | 3.23E-12 | 1.36E-10 | 17.2847 |
| PTPN12 | 0.57113 | 5.372725 | 7.50497 | 3.23E-12 | 1.36E-10 | 17.2846 |
| PAXIP1 | 0.51591 | 2.916033 | 7.50351 | 3.26E-12 | 1.37E-10 | 17.2764 |
| TMED2 | 0.52038 | 6.364403 | 7.50096 | 3.31E-12 | 1.39E-10 | 17.2621 |
| ADARB2 | -1.3771 | 3.23184 | -7.4987 | 3.35E-12 | 1.40E-10 | 17.2497 |
| HIST1H2BG | 0.74955 | 0.987483 | 7.4984 | 3.35E-12 | 1.40E-10 | 17.2478 |
| NUP107 | 0.56713 | 4.160281 | 7.49748 | 3.37E-12 | 1.41E-10 | 17.2426 |
| HMG20B | 0.64536 | 5.728257 | 7.48932 | 3.53E-12 | 1.47E-10 | 17.1969 |
| SNHG16 | 0.59388 | 4.681323 | 7.48752 | 3.57E-12 | 1.48E-10 | 17.1868 |
| TANC1 | 0.68857 | 4.073277 | 7.48034 | 3.72E-12 | 1.54E-10 | 17.1466 |
| PDIA6 | 0.61272 | 6.530837 | 7.47657 | 3.80E-12 | 1.57E-10 | 17.1255 |
| STK40 | 0.8143 | 4.20289 | 7.4762 | 3.81E-12 | 1.57E-10 | 17.1234 |
| TNFAIP8 | 0.90002 | 1.822133 | 7.47592 | 3.82E-12 | 1.57E-10 | 17.1218 |
| HIST1H2AE | 0.89493 | 1.676307 | 7.47346 | 3.87E-12 | 1.59E-10 | 17.1081 |
| HNRNPF | 0.50944 | 6.184223 | 7.47313 | 3.88E-12 | 1.59E-10 | 17.1062 |
| PPIH | 0.54036 | 4.806197 | 7.46666 | 4.02E-12 | 1.64E-10 | 17.07 |
| HIST1H2BO | 0.61724 | 0.586207 | 7.46463 | 4.07E-12 | 1.65E-10 | 17.0587 |
| CNTRL | 0.55689 | 2.897313 | 7.4567 | 4.26E-12 | 1.72E-10 | 17.0143 |
| GGH | 0.72988 | 3.850737 | 7.45143 | 4.39E-12 | 1.77E-10 | 16.9849 |
| SLC4A2 | 0.71101 | 5.570482 | 7.44736 | 4.49E-12 | 1.81E-10 | 16.9622 |
| SLC24A4 | -1.1721 | 2.411354 | -7.4464 | 4.52E-12 | 1.81E-10 | 16.9567 |
| PPP2R2C | -1.6192 | 4.663759 | -7.4444 | 4.57E-12 | 1.83E-10 | 16.9457 |
| CTTNBP2NL | 0.59025 | 3.432221 | 7.43661 | 4.78E-12 | 1.90E-10 | 16.9021 |
| VCL | 0.85626 | 4.187394 | 7.43626 | 4.79E-12 | 1.90E-10 | 16.9002 |
| COQ2 | 0.50232 | 3.058711 | 7.43281 | 4.88E-12 | 1.93E-10 | 16.8809 |
| ZYX | 0.90201 | 6.330055 | 7.43082 | 4.94E-12 | 1.95E-10 | 16.8698 |
| INTS8 | 0.50192 | 4.267418 | 7.41717 | 5.34E-12 | 2.10E-10 | 16.7937 |
| SGSM1 | -0.9839 | 2.321614 | -7.4095 | 5.58E-12 | 2.19E-10 | 16.7507 |
| LBR | 0.63951 | 4.357762 | 7.40069 | 5.87E-12 | 2.28E-10 | 16.7018 |
| EMX2 | -1.11 | 2.798995 | -7.3981 | 5.95E-12 | 2.31E-10 | 16.6871 |
| C4orf46 | 0.54582 | 2.356929 | 7.39317 | 6.12E-12 | 2.37E-10 | 16.66 |
| AAED1 | 0.54962 | 2.875427 | 7.39307 | 6.13E-12 | 2.37E-10 | 16.6594 |
| NUP37 | 0.54971 | 3.412498 | 7.38692 | 6.34E-12 | 2.43E-10 | 16.6252 |
| CAPZA1 | 0.61589 | 5.114191 | 7.38656 | 6.36E-12 | 2.44E-10 | 16.6232 |
| TNFRSF12A | 1.704 | 3.614044 | 7.38066 | 6.57E-12 | 2.51E-10 | 16.5903 |
| RP11-124N14.3 | 1.00299 | 1.246036 | 7.37981 | 6.61E-12 | 2.52E-10 | 16.5856 |
| KIF1A | -0.9421 | 6.872781 | -7.3676 | 7.08E-12 | 2.69E-10 | 16.5175 |
| USP1 | 0.5577 | 4.214685 | 7.36567 | 7.16E-12 | 2.72E-10 | 16.507 |
| CDT1 | 0.88875 | 2.373843 | 7.35549 | 7.59E-12 | 2.87E-10 | 16.4505 |
| MT-RNR1 | -0.7954 | 12.55027 | -7.3519 | 7.75E-12 | 2.92E-10 | 16.4303 |
| CASP4 | 1.09458 | 3.35074 | 7.35005 | 7.83E-12 | 2.95E-10 | 16.4203 |
| IDH1 | 0.73124 | 5.726471 | 7.34328 | 8.13E-12 | 3.05E-10 | 16.3828 |
| GNS | 0.67867 | 5.07485 | 7.34165 | 8.21E-12 | 3.07E-10 | 16.3737 |
| TTC26 | 0.69387 | 2.046129 | 7.33928 | 8.32E-12 | 3.11E-10 | 16.3606 |
| PLEKHA8P1 | 0.80648 | 2.077716 | 7.33725 | 8.42E-12 | 3.14E-10 | 16.3493 |
| LAMA4 | 0.98958 | 4.017175 | 7.33655 | 8.45E-12 | 3.15E-10 | 16.3455 |
| PNRC2 | 0.54917 | 5.608773 | 7.33469 | 8.54E-12 | 3.17E-10 | 16.3351 |
| TES | 0.82537 | 2.004184 | 7.32903 | 8.82E-12 | 3.26E-10 | 16.3038 |
| DR1 | 0.58246 | 4.333998 | 7.32459 | 9.04E-12 | 3.33E-10 | 16.2792 |
| CBX3 | 0.61301 | 6.90727 | 7.32285 | 9.13E-12 | 3.35E-10 | 16.2695 |
| CALR | 0.58059 | 8.499436 | 7.32226 | 9.16E-12 | 3.36E-10 | 16.2662 |
| NTSR2 | -1.8482 | 3.910072 | -7.3209 | 9.24E-12 | 3.37E-10 | 16.2586 |
| HDAC7 | 0.5477 | 4.283393 | 7.31856 | 9.36E-12 | 3.41E-10 | 16.2457 |
| TEAD4 | 1.18411 | 1.879162 | 7.31319 | 9.65E-12 | 3.51E-10 | 16.216 |
| CLIC4 | 0.92982 | 6.753965 | 7.29817 | 1.05E-11 | 3.81E-10 | 16.133 |
| CEP89 | 0.62386 | 3.427291 | 7.29657 | 1.06E-11 | 3.83E-10 | 16.1241 |
| ADAM17 | 0.57966 | 4.134028 | 7.29358 | 1.08E-11 | 3.89E-10 | 16.1076 |
| IFNGR2 | 0.65889 | 5.265018 | 7.28725 | 1.12E-11 | 4.03E-10 | 16.0726 |
| EIF4A1 | 0.53433 | 8.366128 | 7.27574 | 1.19E-11 | 4.28E-10 | 16.009 |
| SHOX2 | 1.2077 | 0.874902 | 7.26482 | 1.27E-11 | 4.52E-10 | 15.9488 |
| CBX8 | 0.64228 | 2.935911 | 7.2623 | 1.29E-11 | 4.58E-10 | 15.9349 |
| REST | 0.68896 | 2.526291 | 7.26172 | 1.29E-11 | 4.59E-10 | 15.9317 |
| VGLL4 | 0.58715 | 5.663734 | 7.25999 | 1.30E-11 | 4.61E-10 | 15.9221 |
| POFUT1 | 0.54717 | 4.339897 | 7.25095 | 1.37E-11 | 4.82E-10 | 15.8723 |
| COL1A1 | 1.90581 | 3.28128 | 7.24435 | 1.42E-11 | 4.96E-10 | 15.8359 |
| HEATR1 | 0.50652 | 3.008152 | 7.2413 | 1.45E-11 | 5.04E-10 | 15.8191 |
| C11orf24 | 0.51627 | 4.374888 | 7.23537 | 1.50E-11 | 5.21E-10 | 15.7865 |
| CD248 | 1.34983 | 2.206192 | 7.23388 | 1.51E-11 | 5.24E-10 | 15.7783 |
| RNFT1 | 0.58593 | 3.41477 | 7.22093 | 1.62E-11 | 5.61E-10 | 15.7071 |
| TMX1 | 0.71533 | 4.831816 | 7.21815 | 1.65E-11 | 5.69E-10 | 15.6918 |
| WASF3 | -0.8359 | 5.663227 | -7.2166 | 1.66E-11 | 5.73E-10 | 15.6833 |
| CHIC2 | 0.85417 | 3.753711 | 7.21611 | 1.67E-11 | 5.74E-10 | 15.6806 |
| FAM91A1 | 0.56981 | 4.176776 | 7.21098 | 1.72E-11 | 5.86E-10 | 15.6523 |
| ENPEP | 0.8902 | 1.372064 | 7.20947 | 1.73E-11 | 5.90E-10 | 15.644 |
| PCDH18 | 0.82751 | 2.752124 | 7.20547 | 1.77E-11 | 6.03E-10 | 15.6221 |
| PCOLCE | 1.11036 | 3.813269 | 7.2039 | 1.79E-11 | 6.07E-10 | 15.6135 |
| B4GALT5 | 0.63773 | 4.925484 | 7.19971 | 1.83E-11 | 6.19E-10 | 15.5905 |
| KBTBD11 | -0.9279 | 4.482275 | -7.1916 | 1.92E-11 | 6.45E-10 | 15.5459 |
| ICMT | 0.50752 | 4.323147 | 7.19152 | 1.92E-11 | 6.45E-10 | 15.5455 |
| CHST14 | 0.62897 | 3.760754 | 7.18249 | 2.02E-11 | 6.74E-10 | 15.496 |
| ADAM19 | 0.75776 | 1.901728 | 7.18144 | 2.03E-11 | 6.77E-10 | 15.4902 |
| BCL2L12 | 0.77667 | 2.477573 | 7.18089 | 2.04E-11 | 6.78E-10 | 15.4872 |
| HMGN4 | 0.52323 | 5.423783 | 7.18069 | 2.04E-11 | 6.78E-10 | 15.4861 |
| SDCCAG3 | 0.57478 | 5.316275 | 7.18023 | 2.04E-11 | 6.79E-10 | 15.4836 |
| GPRC5A | 1.15705 | 1.141325 | 7.17377 | 2.12E-11 | 7.02E-10 | 15.4482 |
| CMTM3 | 0.82558 | 5.3337 | 7.17177 | 2.14E-11 | 7.09E-10 | 15.4372 |
| FSBP | 0.71139 | 1.525181 | 7.16744 | 2.20E-11 | 7.25E-10 | 15.4135 |
| SH3GLB1 | 0.63764 | 5.276078 | 7.16728 | 2.20E-11 | 7.25E-10 | 15.4126 |
| HIST1H2BN | 0.5949 | 1.269235 | 7.16529 | 2.22E-11 | 7.32E-10 | 15.4017 |
| BST1 | 0.73729 | 1.895792 | 7.16422 | 2.24E-11 | 7.36E-10 | 15.3959 |
| RASGRF1 | -1.3497 | 3.428987 | -7.1624 | 2.26E-11 | 7.42E-10 | 15.3857 |
| ST8SIA4 | 0.71686 | 1.979849 | 7.16198 | 2.26E-11 | 7.42E-10 | 15.3836 |
| TXNDC17 | 0.51459 | 5.92432 | 7.16097 | 2.28E-11 | 7.45E-10 | 15.3781 |
| COL5A1 | 1.35963 | 1.934041 | 7.15611 | 2.34E-11 | 7.65E-10 | 15.3515 |
| MT-ND6 | -0.6234 | 13.10905 | -7.1557 | 2.34E-11 | 7.65E-10 | 15.3495 |
| CHSY1 | 0.62068 | 3.891767 | 7.15554 | 2.35E-11 | 7.65E-10 | 15.3484 |
| PTMS | 0.74916 | 8.89086 | 7.15322 | 2.38E-11 | 7.74E-10 | 15.3357 |
| NEDD1 | 0.65475 | 3.217646 | 7.15245 | 2.39E-11 | 7.76E-10 | 15.3314 |
| MIS18BP1 | 0.62962 | 2.736596 | 7.14486 | 2.49E-11 | 8.09E-10 | 15.2899 |
| PLOD3 | 0.68015 | 5.148426 | 7.13397 | 2.65E-11 | 8.54E-10 | 15.2304 |
| EFNB1 | 0.61783 | 3.166972 | 7.13295 | 2.66E-11 | 8.58E-10 | 15.2248 |
| C5orf15 | 0.53104 | 4.997091 | 7.12971 | 2.71E-11 | 8.73E-10 | 15.2071 |
| NUP188 | 0.57233 | 4.536937 | 7.12192 | 2.83E-11 | 9.08E-10 | 15.1646 |
| SUV39H2 | 0.5809 | 2.920797 | 7.11945 | 2.87E-11 | 9.18E-10 | 15.1511 |
| EIF4EBP1 | 0.88769 | 4.832405 | 7.11914 | 2.88E-11 | 9.19E-10 | 15.1495 |
| ADPGK | 0.50169 | 4.559017 | 7.11445 | 2.95E-11 | 9.40E-10 | 15.1239 |
| NLRC5 | 0.91134 | 3.443355 | 7.1137 | 2.97E-11 | 9.43E-10 | 15.1197 |
| RP11-303E16.2 | 0.64818 | 1.919017 | 7.11161 | 3.00E-11 | 9.53E-10 | 15.1084 |
| GRAMD1B | -0.7425 | 4.002135 | -7.1086 | 3.05E-11 | 9.65E-10 | 15.0919 |
| OLFML2B | 1.12253 | 3.099197 | 7.10285 | 3.15E-11 | 9.94E-10 | 15.0606 |
| SLFN12 | 0.7065 | 1.674793 | 7.10181 | 3.17E-11 | 9.98E-10 | 15.0549 |
| ZNF486 | 0.50968 | 2.051244 | 7.1011 | 3.18E-11 | 1.00E-09 | 15.0511 |
| RP11-245J24.1 | -1.0302 | 1.169882 | -7.0979 | 3.24E-11 | 1.01E-09 | 15.0338 |
| MMRN1 | 0.72285 | 1.172191 | 7.0972 | 3.25E-11 | 1.02E-09 | 15.0298 |
| C1orf109 | 0.5559 | 4.306249 | 7.097 | 3.26E-11 | 1.02E-09 | 15.0287 |
| PTK7 | 0.72082 | 4.119393 | 7.09046 | 3.38E-11 | 1.05E-09 | 14.9931 |
| RP11-227B21.2 | -1.1541 | 1.633883 | -7.0878 | 3.43E-11 | 1.07E-09 | 14.9788 |
| MAN2B1 | 0.67584 | 4.997569 | 7.08576 | 3.47E-11 | 1.08E-09 | 14.9675 |
| CDC25B | 0.58143 | 5.105845 | 7.08538 | 3.47E-11 | 1.08E-09 | 14.9654 |
| PPP1R18 | 0.74737 | 5.089812 | 7.08513 | 3.48E-11 | 1.08E-09 | 14.9641 |
| LAMB1 | 1.22729 | 3.960049 | 7.0775 | 3.63E-11 | 1.12E-09 | 14.9226 |
| LIN9 | 0.50001 | 1.880519 | 7.075 | 3.68E-11 | 1.13E-09 | 14.909 |
| LXN | 0.94084 | 2.224345 | 7.0724 | 3.74E-11 | 1.15E-09 | 14.8948 |
| ITPKC | 0.72391 | 3.171491 | 7.06717 | 3.85E-11 | 1.18E-09 | 14.8664 |
| TCF3 | 0.69162 | 5.667357 | 7.06274 | 3.94E-11 | 1.20E-09 | 14.8423 |
| SLC25A18 | -1.0691 | 5.673509 | -7.0621 | 3.96E-11 | 1.21E-09 | 14.8386 |
| GRWD1 | 0.55837 | 3.543656 | 7.06177 | 3.96E-11 | 1.21E-09 | 14.837 |
| CHD1 | 0.50205 | 3.953427 | 7.05946 | 4.02E-11 | 1.22E-09 | 14.8245 |
| CDK6 | 1.0184 | 2.950574 | 7.05794 | 4.05E-11 | 1.23E-09 | 14.8163 |
| GALNT2 | 0.62146 | 5.179421 | 7.05788 | 4.05E-11 | 1.23E-09 | 14.8159 |
| 9-Sep | 0.52283 | 6.866553 | 7.05206 | 4.18E-11 | 1.27E-09 | 14.7843 |
| CALD1 | 0.88685 | 5.791407 | 7.05137 | 4.20E-11 | 1.27E-09 | 14.7806 |
| RUNX1 | 1.0788 | 2.288644 | 7.04996 | 4.23E-11 | 1.28E-09 | 14.7729 |
| SNRPG | 0.51633 | 6.371591 | 7.04252 | 4.41E-11 | 1.33E-09 | 14.7326 |
| SLC16A1 | 0.70976 | 5.242406 | 7.03977 | 4.48E-11 | 1.34E-09 | 14.7176 |
| PTPN7 | 0.87551 | 1.440329 | 7.03908 | 4.50E-11 | 1.35E-09 | 14.7139 |
| ARF6 | 0.51702 | 4.787354 | 7.03708 | 4.55E-11 | 1.36E-09 | 14.703 |
| ZNF480 | 0.52848 | 2.505305 | 7.03463 | 4.61E-11 | 1.38E-09 | 14.6897 |
| MT-RNR2 | -0.6916 | 13.90149 | -7.031 | 4.70E-11 | 1.40E-09 | 14.6702 |
| BACH1 | 0.5299 | 3.591599 | 7.02959 | 4.74E-11 | 1.41E-09 | 14.6624 |
| SLC34A2 | 0.69269 | 0.623786 | 7.02917 | 4.75E-11 | 1.41E-09 | 14.6602 |
| MYO1C | 0.60406 | 3.590313 | 7.02682 | 4.81E-11 | 1.43E-09 | 14.6474 |
| TMEM43 | 0.52005 | 5.02585 | 7.02671 | 4.82E-11 | 1.43E-09 | 14.6468 |
| STK17A | 0.70206 | 4.067121 | 7.02533 | 4.85E-11 | 1.43E-09 | 14.6393 |
| PXDNL | 0.61088 | 0.683787 | 7.02313 | 4.91E-11 | 1.45E-09 | 14.6275 |
| CCDC150 | 0.66406 | 1.544294 | 7.02046 | 4.99E-11 | 1.47E-09 | 14.613 |
| TMEM71 | 0.88189 | 1.362547 | 7.02038 | 4.99E-11 | 1.47E-09 | 14.6125 |
| ATAD3A | 0.55245 | 4.045761 | 7.01817 | 5.05E-11 | 1.49E-09 | 14.6006 |
| FAM129A | 1.02855 | 2.285228 | 7.01707 | 5.08E-11 | 1.49E-09 | 14.5946 |
| PHTF1 | 0.65516 | 3.189174 | 7.01572 | 5.12E-11 | 1.50E-09 | 14.5873 |
| SPIN4 | 0.50508 | 1.674486 | 7.01316 | 5.19E-11 | 1.52E-09 | 14.5734 |
| UBTD2 | 0.52598 | 3.929249 | 7.01188 | 5.23E-11 | 1.53E-09 | 14.5665 |
| CD151 | 0.77566 | 6.23741 | 7.00519 | 5.43E-11 | 1.58E-09 | 14.5303 |
| CPLX3 | -1.1288 | 1.509001 | -7.0049 | 5.44E-11 | 1.58E-09 | 14.5286 |
| KIAA0922 | 0.60525 | 2.312216 | 7.00397 | 5.47E-11 | 1.59E-09 | 14.5237 |
| UBE2S | 0.66998 | 5.549575 | 7.00393 | 5.47E-11 | 1.59E-09 | 14.5235 |
| TMEM255B | 0.73175 | 1.990236 | 7.00342 | 5.48E-11 | 1.59E-09 | 14.5208 |
| CREB5 | 0.84076 | 3.720792 | 6.9999 | 5.59E-11 | 1.62E-09 | 14.5017 |
| CNN3 | 0.92572 | 8.161936 | 6.99964 | 5.60E-11 | 1.62E-09 | 14.5003 |
| CDK4 | 1.12307 | 7.126828 | 6.99712 | 5.68E-11 | 1.64E-09 | 14.4867 |
| IRAK1 | 0.5234 | 5.617159 | 6.99614 | 5.71E-11 | 1.65E-09 | 14.4814 |
| MIR4435-1HG | 1.45416 | 3.174591 | 6.99058 | 5.89E-11 | 1.69E-09 | 14.4513 |
| HAS2 | 1.09846 | 1.913122 | 6.99055 | 5.89E-11 | 1.69E-09 | 14.4511 |
| MAGOH | 0.5746 | 5.112671 | 6.98495 | 6.07E-11 | 1.74E-09 | 14.4209 |
| SPON2 | 1.37928 | 2.664025 | 6.98422 | 6.10E-11 | 1.74E-09 | 14.417 |
| CYS1 | -0.6831 | 1.890896 | -6.9838 | 6.11E-11 | 1.75E-09 | 14.4147 |
| MAP7D3 | 0.60339 | 2.153155 | 6.97658 | 6.36E-11 | 1.81E-09 | 14.3757 |
| CLIC1 | 1.17846 | 5.587265 | 6.96534 | 6.77E-11 | 1.91E-09 | 14.3151 |
| HIST1H2BJ | 0.78302 | 1.133392 | 6.9645 | 6.80E-11 | 1.92E-09 | 14.3106 |
| VIM | 1.65035 | 9.142917 | 6.96085 | 6.94E-11 | 1.95E-09 | 14.2909 |
| NASP | 0.58918 | 6.309215 | 6.95932 | 7.00E-11 | 1.97E-09 | 14.2826 |
| ZNF473 | 0.5289 | 2.575066 | 6.95657 | 7.11E-11 | 1.99E-09 | 14.2678 |
| NRP1 | 0.9899 | 4.149699 | 6.95543 | 7.15E-11 | 2.00E-09 | 14.2617 |
| GABRD | -1.4606 | 3.658146 | -6.9548 | 7.18E-11 | 2.01E-09 | 14.2582 |
| NDRG2 | -1.0829 | 9.91658 | -6.9542 | 7.20E-11 | 2.01E-09 | 14.2551 |
| CAV1 | 1.30726 | 4.036287 | 6.94941 | 7.39E-11 | 2.06E-09 | 14.2292 |
| SHKBP1 | 0.64568 | 4.388555 | 6.94669 | 7.51E-11 | 2.08E-09 | 14.2146 |
| GUSB | 0.5605 | 4.885398 | 6.94519 | 7.57E-11 | 2.10E-09 | 14.2065 |
| PLXNA3 | 0.63831 | 4.39607 | 6.9443 | 7.61E-11 | 2.11E-09 | 14.2017 |
| SEPN1 | 0.71835 | 5.808786 | 6.93927 | 7.82E-11 | 2.16E-09 | 14.1747 |
| ORMDL2 | 0.5424 | 4.338826 | 6.9367 | 7.93E-11 | 2.19E-09 | 14.1608 |
| RHOC | 0.78464 | 7.584755 | 6.92447 | 8.48E-11 | 2.33E-09 | 14.0951 |
| ALDH18A1 | 0.51862 | 4.737415 | 6.92385 | 8.51E-11 | 2.33E-09 | 14.0917 |
| NID2 | 0.77151 | 3.177957 | 6.92263 | 8.57E-11 | 2.35E-09 | 14.0852 |
| GPX8 | 1.12714 | 1.429142 | 6.92177 | 8.61E-11 | 2.35E-09 | 14.0805 |
| HSPA5 | 0.61551 | 6.906496 | 6.91552 | 8.91E-11 | 2.43E-09 | 14.0469 |
| CDC42 | 0.50162 | 6.974199 | 6.91379 | 9.00E-11 | 2.45E-09 | 14.0376 |
| CPSF4 | 0.56247 | 5.452945 | 6.91369 | 9.00E-11 | 2.45E-09 | 14.0371 |
| TGIF1 | 0.97885 | 4.108725 | 6.91252 | 9.06E-11 | 2.46E-09 | 14.0309 |
| LAG3 | 0.72703 | 1.22589 | 6.90949 | 9.22E-11 | 2.50E-09 | 14.0146 |
| CCDC142 | 0.56093 | 3.054265 | 6.90572 | 9.41E-11 | 2.55E-09 | 13.9943 |
| POLE | 0.77403 | 3.477447 | 6.90344 | 9.53E-11 | 2.57E-09 | 13.9821 |
| CNPY4 | 0.62 | 4.469988 | 6.89956 | 9.73E-11 | 2.62E-09 | 13.9613 |
| AFAP1L1 | 0.82517 | 2.288324 | 6.89869 | 9.78E-11 | 2.63E-09 | 13.9566 |
| EMX2OS | -1.2475 | 3.008292 | -6.8969 | 9.87E-11 | 2.65E-09 | 13.9472 |
| IFI16 | 0.90029 | 5.717244 | 6.89542 | 9.96E-11 | 2.67E-09 | 13.9391 |
| LDHD | -0.6892 | 3.453682 | -6.8944 | 1.00E-10 | 2.68E-09 | 13.9334 |
| ISL2 | 0.7111 | 0.620047 | 6.89341 | 1.01E-10 | 2.69E-09 | 13.9283 |
| ATP6V1G2 | -1.0172 | 6.575126 | -6.8923 | 1.01E-10 | 2.71E-09 | 13.9222 |
| NUDT1 | 0.56164 | 5.127371 | 6.89171 | 1.02E-10 | 2.71E-09 | 13.9192 |
| UGDH | 0.71045 | 3.951616 | 6.89004 | 1.03E-10 | 2.73E-09 | 13.9102 |
| INTS7 | 0.55669 | 3.277188 | 6.88657 | 1.05E-10 | 2.78E-09 | 13.8916 |
| RAI14 | 0.63742 | 3.478416 | 6.88559 | 1.05E-10 | 2.79E-09 | 13.8864 |
| ADAMTS7 | 0.73668 | 1.235213 | 6.88508 | 1.05E-10 | 2.79E-09 | 13.8837 |
| PDIA3 | 0.51608 | 7.375061 | 6.88429 | 1.06E-10 | 2.80E-09 | 13.8794 |
| PANX1 | 0.52741 | 3.771701 | 6.8833 | 1.06E-10 | 2.81E-09 | 13.8741 |
| DAPK3 | 0.51247 | 5.832433 | 6.88193 | 1.07E-10 | 2.83E-09 | 13.8668 |
| TMED9 | 0.51064 | 6.569572 | 6.88074 | 1.08E-10 | 2.84E-09 | 13.8604 |
| ZNF28 | 0.6238 | 2.782298 | 6.87843 | 1.09E-10 | 2.87E-09 | 13.848 |
| GRN | 0.66454 | 6.544349 | 6.8781 | 1.10E-10 | 2.87E-09 | 13.8463 |
| BAX | 0.55091 | 6.387838 | 6.87787 | 1.10E-10 | 2.87E-09 | 13.845 |
| C7orf49 | 0.50852 | 4.964456 | 6.86712 | 1.16E-10 | 3.03E-09 | 13.7875 |
| CCDC50 | 0.57341 | 4.547486 | 6.86131 | 1.20E-10 | 3.12E-09 | 13.7564 |
| GALNT1 | 0.57546 | 5.261688 | 6.85879 | 1.22E-10 | 3.16E-09 | 13.7429 |
| STAT3 | 0.5623 | 5.896513 | 6.85518 | 1.24E-10 | 3.22E-09 | 13.7237 |
| TMEM37 | 0.71295 | 1.928635 | 6.85479 | 1.24E-10 | 3.22E-09 | 13.7216 |
| ATP6V0A2 | 0.53488 | 3.243402 | 6.8544 | 1.25E-10 | 3.22E-09 | 13.7195 |
| SLAIN2 | 0.57943 | 4.09032 | 6.85339 | 1.25E-10 | 3.24E-09 | 13.7141 |
| NFIL3 | 0.74302 | 3.959668 | 6.85325 | 1.26E-10 | 3.24E-09 | 13.7133 |
| PKN2 | 0.57254 | 3.779383 | 6.8505 | 1.27E-10 | 3.27E-09 | 13.6986 |
| PROS1 | 0.92142 | 3.936527 | 6.84776 | 1.29E-10 | 3.31E-09 | 13.684 |
| PIM1 | 0.82706 | 3.606291 | 6.8475 | 1.30E-10 | 3.31E-09 | 13.6826 |
| MOB1A | 0.57923 | 4.702706 | 6.84706 | 1.30E-10 | 3.32E-09 | 13.6803 |
| GABRG1 | -1.3495 | 2.706246 | -6.8403 | 1.35E-10 | 3.44E-09 | 13.6441 |
| PSMC3IP | 0.51976 | 3.173664 | 6.8394 | 1.35E-10 | 3.45E-09 | 13.6394 |
| NMI | 0.8242 | 2.926395 | 6.8373 | 1.37E-10 | 3.48E-09 | 13.6282 |
| HOXD9 | 1.03301 | 0.821004 | 6.83601 | 1.38E-10 | 3.49E-09 | 13.6213 |
| COL1A2 | 1.57706 | 4.255514 | 6.83598 | 1.38E-10 | 3.49E-09 | 13.6211 |
| SLC35F5 | 0.51965 | 4.176783 | 6.83588 | 1.38E-10 | 3.49E-09 | 13.6206 |
| HDAC2 | 0.53909 | 5.973241 | 6.83552 | 1.38E-10 | 3.49E-09 | 13.6187 |
| HIST1H2BK | 0.94271 | 4.172816 | 6.83191 | 1.41E-10 | 3.56E-09 | 13.5994 |
| SLC25A19 | 0.6191 | 3.16526 | 6.828 | 1.44E-10 | 3.63E-09 | 13.5786 |
| SOAT1 | 0.68807 | 3.73744 | 6.81775 | 1.52E-10 | 3.82E-09 | 13.5239 |
| DISP1 | 0.63565 | 2.629005 | 6.81556 | 1.54E-10 | 3.86E-09 | 13.5123 |
| PSORS1C1 | 1.14462 | 1.659565 | 6.81462 | 1.55E-10 | 3.87E-09 | 13.5072 |
| ADAM12 | 1.06845 | 1.391426 | 6.81447 | 1.55E-10 | 3.87E-09 | 13.5065 |
| CDCA7 | 1.02336 | 2.713208 | 6.81368 | 1.56E-10 | 3.89E-09 | 13.5022 |
| IGFBP2 | 1.87083 | 5.09478 | 6.81326 | 1.56E-10 | 3.89E-09 | 13.5 |
| PIF1 | 0.76015 | 1.813866 | 6.81162 | 1.58E-10 | 3.91E-09 | 13.4913 |
| CTD-2562J17.7 | -1.0171 | 3.03736 | -6.8049 | 1.64E-10 | 4.06E-09 | 13.4552 |
| HYAL2 | 0.62855 | 4.867927 | 6.80259 | 1.66E-10 | 4.10E-09 | 13.4432 |
| TXNDC12 | 0.51288 | 5.098082 | 6.80141 | 1.67E-10 | 4.12E-09 | 13.4369 |
| LEPREL4 | 0.5863 | 3.699646 | 6.79247 | 1.75E-10 | 4.31E-09 | 13.3893 |
| CTD-2380F24.1 | -1.0027 | 1.810608 | -6.788 | 1.79E-10 | 4.40E-09 | 13.3655 |
| DDX11 | 0.82134 | 3.465813 | 6.78707 | 1.80E-10 | 4.42E-09 | 13.3607 |
| BMP1 | 0.72143 | 3.932118 | 6.7862 | 1.81E-10 | 4.44E-09 | 13.356 |
| SLC22A6 | -0.91 | 1.56043 | -6.7855 | 1.82E-10 | 4.45E-09 | 13.3522 |
| DRAXIN | 0.99682 | 1.551566 | 6.78527 | 1.82E-10 | 4.45E-09 | 13.3511 |
| MANF | 0.61572 | 5.806306 | 6.77635 | 1.91E-10 | 4.65E-09 | 13.3037 |
| TRAM1 | 0.61364 | 5.770394 | 6.77453 | 1.93E-10 | 4.70E-09 | 13.294 |
| CYP46A1 | -0.996 | 4.104516 | -6.7728 | 1.95E-10 | 4.74E-09 | 13.2848 |
| PRPF40A | 0.56938 | 5.165926 | 6.77244 | 1.95E-10 | 4.74E-09 | 13.2829 |
| MYB | 0.57209 | 0.698612 | 6.77188 | 1.96E-10 | 4.75E-09 | 13.28 |
| TP53I3 | 0.79901 | 4.082098 | 6.77098 | 1.97E-10 | 4.77E-09 | 13.2752 |
| RP11-61L23.2 | 1.07946 | 1.665118 | 6.77084 | 1.97E-10 | 4.77E-09 | 13.2745 |
| ACSL6 | -1.1528 | 4.806652 | -6.7695 | 1.98E-10 | 4.79E-09 | 13.2673 |
| ANKRD24 | -0.6955 | 2.653549 | -6.7624 | 2.06E-10 | 4.96E-09 | 13.2298 |
| IGFBP4 | 1.03671 | 4.76742 | 6.76112 | 2.08E-10 | 4.99E-09 | 13.2229 |
| CEP152 | 0.51867 | 1.397483 | 6.75568 | 2.14E-10 | 5.13E-09 | 13.1941 |
| MGP | 1.80406 | 4.974617 | 6.75161 | 2.19E-10 | 5.24E-09 | 13.1725 |
| ANTXR2 | 0.82983 | 3.373891 | 6.7508 | 2.20E-10 | 5.26E-09 | 13.1682 |
| PLEKHA4 | 1.2505 | 3.763422 | 6.74388 | 2.28E-10 | 5.45E-09 | 13.1315 |
| H19 | 1.80865 | 1.970663 | 6.74271 | 2.30E-10 | 5.47E-09 | 13.1253 |
| RP5-1119A7.17 | -1.4799 | 2.901983 | -6.7427 | 2.30E-10 | 5.47E-09 | 13.1251 |
| DCBLD1 | 0.54007 | 2.824309 | 6.73309 | 2.42E-10 | 5.73E-09 | 13.0744 |
| DNA2 | 0.53297 | 1.7083 | 6.73138 | 2.44E-10 | 5.78E-09 | 13.0654 |
| ZFP36L2 | 0.76278 | 5.763395 | 6.73003 | 2.46E-10 | 5.81E-09 | 13.0583 |
| ITPK1 | -0.6342 | 6.938931 | -6.7241 | 2.54E-10 | 5.97E-09 | 13.0268 |
| HOXA5 | 1.16493 | 0.878511 | 6.72208 | 2.57E-10 | 6.03E-09 | 13.0162 |
| DDX12P | 0.71917 | 1.564679 | 6.71941 | 2.61E-10 | 6.11E-09 | 13.0021 |
| HSD3B7 | 0.72217 | 2.511865 | 6.71832 | 2.62E-10 | 6.14E-09 | 12.9963 |
| ITPRIPL2 | 0.66799 | 2.653234 | 6.71624 | 2.65E-10 | 6.21E-09 | 12.9854 |
| ZNF468 | 0.60288 | 2.305088 | 6.71418 | 2.68E-10 | 6.27E-09 | 12.9744 |
| AIFM3 | -1.3881 | 3.288755 | -6.7125 | 2.70E-10 | 6.32E-09 | 12.9658 |
| NID1 | 1.06237 | 3.91059 | 6.71165 | 2.72E-10 | 6.34E-09 | 12.9611 |
| MCM7 | 0.7913 | 6.213522 | 6.71141 | 2.72E-10 | 6.35E-09 | 12.9598 |
| TULP3 | 0.57373 | 3.881988 | 6.70754 | 2.78E-10 | 6.47E-09 | 12.9394 |
| BEX2 | -1.041 | 6.107167 | -6.7074 | 2.78E-10 | 6.47E-09 | 12.9388 |
| RPL11 | 0.54051 | 10.12512 | 6.70494 | 2.82E-10 | 6.52E-09 | 12.9257 |
| HMGA1 | 0.65893 | 5.424615 | 6.70203 | 2.86E-10 | 6.61E-09 | 12.9103 |
| SPAG5 | 0.78355 | 3.385191 | 6.70183 | 2.87E-10 | 6.61E-09 | 12.9093 |
| S100A3 | 1.21065 | 1.550208 | 6.69892 | 2.91E-10 | 6.69E-09 | 12.8939 |
| CCDC102B | 0.51112 | 3.507167 | 6.6984 | 2.92E-10 | 6.70E-09 | 12.8912 |
| ZNF790 | 0.52248 | 2.689429 | 6.69738 | 2.94E-10 | 6.73E-09 | 12.8858 |
| RP11-565P22.6 | 0.95854 | 2.011027 | 6.69103 | 3.04E-10 | 6.93E-09 | 12.8523 |
| RBMS1 | 0.80562 | 3.455785 | 6.68952 | 3.06E-10 | 6.97E-09 | 12.8444 |
| PLEKHB1 | -0.8907 | 8.721017 | -6.685 | 3.14E-10 | 7.12E-09 | 12.8206 |
| PPP1R3E | -0.5399 | 4.060218 | -6.6817 | 3.20E-10 | 7.25E-09 | 12.803 |
| SPPL2A | 0.58532 | 4.206679 | 6.67568 | 3.30E-10 | 7.46E-09 | 12.7714 |
| SLC1A2 | -1.2114 | 6.52622 | -6.6739 | 3.33E-10 | 7.52E-09 | 12.762 |
| DZIP1L | 0.60121 | 2.107778 | 6.67261 | 3.36E-10 | 7.57E-09 | 12.7553 |
| BZW2 | 0.62028 | 4.750837 | 6.67133 | 3.38E-10 | 7.61E-09 | 12.7486 |
| HOXA7 | 1.28734 | 1.068412 | 6.67075 | 3.39E-10 | 7.63E-09 | 12.7455 |
| CCT6A | 0.5359 | 6.712876 | 6.66999 | 3.41E-10 | 7.66E-09 | 12.7415 |
| PTBP3 | 0.51897 | 2.966149 | 6.6695 | 3.41E-10 | 7.66E-09 | 12.7389 |
| HMGN2 | 0.57156 | 8.181606 | 6.66892 | 3.42E-10 | 7.67E-09 | 12.7359 |
| WDR3 | 0.64664 | 3.401846 | 6.66814 | 3.44E-10 | 7.70E-09 | 12.7317 |
| BICD1 | 0.6069 | 3.74419 | 6.66427 | 3.51E-10 | 7.84E-09 | 12.7114 |
| RNF138 | 0.51101 | 4.264518 | 6.66026 | 3.59E-10 | 8.00E-09 | 12.6903 |
| SLC35D1 | 0.51227 | 2.463213 | 6.65745 | 3.64E-10 | 8.11E-09 | 12.6755 |
| PHF13 | 0.51381 | 3.260473 | 6.65646 | 3.66E-10 | 8.15E-09 | 12.6703 |
| EXOSC10 | 0.50054 | 5.134562 | 6.65476 | 3.70E-10 | 8.20E-09 | 12.6614 |
| DRAM2 | 0.5548 | 5.094259 | 6.65265 | 3.74E-10 | 8.27E-09 | 12.6503 |
| ANO6 | 0.84295 | 4.067712 | 6.65044 | 3.78E-10 | 8.35E-09 | 12.6387 |
| S100PBP | 0.51685 | 3.694061 | 6.64643 | 3.87E-10 | 8.51E-09 | 12.6177 |
| MAPKAPK2 | 0.54086 | 5.241079 | 6.64529 | 3.89E-10 | 8.56E-09 | 12.6117 |
| KIAA0020 | 0.52483 | 3.363614 | 6.64377 | 3.92E-10 | 8.60E-09 | 12.6037 |
| VASP | 0.71802 | 4.238628 | 6.64338 | 3.93E-10 | 8.61E-09 | 12.6017 |
| SPOCD1 | 1.9453 | 2.712142 | 6.64275 | 3.94E-10 | 8.64E-09 | 12.5983 |
| MYCBP | 0.70182 | 2.725701 | 6.63941 | 4.02E-10 | 8.79E-09 | 12.5808 |
| CHTF18 | 0.68387 | 3.571284 | 6.63789 | 4.05E-10 | 8.84E-09 | 12.5729 |
| MYH7B | -0.6359 | 1.938313 | -6.6364 | 4.08E-10 | 8.90E-09 | 12.5651 |
| HOXA6 | 0.88286 | 0.630373 | 6.63636 | 4.08E-10 | 8.90E-09 | 12.5648 |
| ZBTB42 | 0.64436 | 1.401064 | 6.63577 | 4.10E-10 | 8.92E-09 | 12.5617 |
| PRF1 | 0.67301 | 1.085329 | 6.63288 | 4.16E-10 | 9.05E-09 | 12.5466 |
| SPINK8 | 0.86126 | 0.937775 | 6.63093 | 4.20E-10 | 9.14E-09 | 12.5364 |
| ESM1 | 1.47449 | 1.402349 | 6.61899 | 4.48E-10 | 9.69E-09 | 12.4738 |
| MCAM | 0.99761 | 4.916521 | 6.6164 | 4.55E-10 | 9.81E-09 | 12.4602 |
| LYNX1 | -0.8571 | 4.86767 | -6.6159 | 4.56E-10 | 9.83E-09 | 12.4574 |
| FHL3 | 0.67392 | 3.902859 | 6.61472 | 4.59E-10 | 9.88E-09 | 12.4514 |
| GPR65 | 1.03966 | 1.694969 | 6.61327 | 4.62E-10 | 9.95E-09 | 12.4438 |
| FAM46A | 0.81459 | 2.17258 | 6.60998 | 4.71E-10 | 1.01E-08 | 12.4266 |
| LRRC36 | 0.61464 | 0.97799 | 6.60589 | 4.81E-10 | 1.03E-08 | 12.4052 |
| PPP1R14B | 0.69003 | 6.856641 | 6.60179 | 4.92E-10 | 1.05E-08 | 12.3838 |
| LIMA1 | 0.84533 | 5.620885 | 6.60117 | 4.93E-10 | 1.06E-08 | 12.3805 |
| KIAA0040 | 0.97114 | 2.432694 | 6.60071 | 4.95E-10 | 1.06E-08 | 12.3781 |
| GJB6 | -1.5422 | 2.146628 | -6.5925 | 5.17E-10 | 1.10E-08 | 12.3352 |
| MECOM | 0.65113 | 2.128183 | 6.59229 | 5.18E-10 | 1.10E-08 | 12.3341 |
| TMED7-TICAM2 | 0.69913 | 0.941829 | 6.58539 | 5.37E-10 | 1.14E-08 | 12.2981 |
| PPP4R4 | -1.0713 | 2.116835 | -6.5806 | 5.51E-10 | 1.17E-08 | 12.2729 |
| TNC | 1.41214 | 5.434789 | 6.58017 | 5.52E-10 | 1.17E-08 | 12.2708 |
| ARHGEF19 | 0.64471 | 1.279496 | 6.57546 | 5.66E-10 | 1.20E-08 | 12.2462 |
| ENG | 0.78929 | 4.7048 | 6.57529 | 5.67E-10 | 1.20E-08 | 12.2453 |
| IL15RA | 0.63401 | 1.960575 | 6.57441 | 5.70E-10 | 1.20E-08 | 12.2407 |
| DSE | 0.66852 | 3.035608 | 6.57429 | 5.70E-10 | 1.20E-08 | 12.2401 |
| CCDC109B | 1.07055 | 2.652807 | 6.57068 | 5.81E-10 | 1.22E-08 | 12.2213 |
| CHRNA9 | 1.08373 | 0.885935 | 6.56958 | 5.85E-10 | 1.23E-08 | 12.2155 |
| MXRA5 | 0.88445 | 1.281793 | 6.56822 | 5.89E-10 | 1.23E-08 | 12.2084 |
| COMMD2 | 0.54934 | 4.684168 | 6.56598 | 5.96E-10 | 1.25E-08 | 12.1967 |
| TPST1 | 0.74951 | 5.223035 | 6.56307 | 6.05E-10 | 1.26E-08 | 12.1816 |
| METTL21B | 0.89524 | 2.724044 | 6.56284 | 6.06E-10 | 1.26E-08 | 12.1804 |
| RGS7 | -1.1438 | 2.898169 | -6.5596 | 6.17E-10 | 1.28E-08 | 12.1634 |
| C12orf39 | -1.4242 | 3.384881 | -6.5591 | 6.18E-10 | 1.29E-08 | 12.161 |
| HSPA12A | -0.8974 | 3.75311 | -6.5541 | 6.35E-10 | 1.32E-08 | 12.135 |
| RP11-145M9.4 | 0.67433 | 4.809068 | 6.54899 | 6.53E-10 | 1.35E-08 | 12.1082 |
| PHYHIP | -1.529 | 4.38193 | -6.5463 | 6.62E-10 | 1.37E-08 | 12.094 |
| PIK3R3 | 0.62614 | 3.582158 | 6.54585 | 6.64E-10 | 1.37E-08 | 12.0919 |
| TMEM51 | 0.60161 | 3.143424 | 6.54547 | 6.65E-10 | 1.37E-08 | 12.0899 |
| MT-ND2 | -0.629 | 13.46061 | -6.5447 | 6.68E-10 | 1.37E-08 | 12.0861 |
| NCAPD3 | 0.59635 | 3.877304 | 6.54087 | 6.82E-10 | 1.40E-08 | 12.066 |
| KCNIP2 | -1.5687 | 4.920182 | -6.5406 | 6.83E-10 | 1.40E-08 | 12.0645 |
| LIG1 | 0.57513 | 4.045681 | 6.53829 | 6.91E-10 | 1.42E-08 | 12.0526 |
| PTAR1 | 0.53823 | 3.882576 | 6.53333 | 7.10E-10 | 1.45E-08 | 12.0268 |
| MICB | 0.56044 | 1.162591 | 6.53217 | 7.14E-10 | 1.46E-08 | 12.0207 |
| KDM5A | 0.52743 | 3.700489 | 6.53091 | 7.19E-10 | 1.46E-08 | 12.0142 |
| TSPAN9 | 0.50311 | 3.285557 | 6.52947 | 7.25E-10 | 1.47E-08 | 12.0067 |
| MMP2 | 0.97214 | 4.625278 | 6.52898 | 7.26E-10 | 1.48E-08 | 12.0042 |
| CABP1 | -1.6048 | 3.820338 | -6.5273 | 7.33E-10 | 1.49E-08 | 11.9955 |
| C1orf86 | 0.5576 | 5.255961 | 6.52707 | 7.34E-10 | 1.49E-08 | 11.9943 |
| FAIM2 | -0.9029 | 6.918898 | -6.5227 | 7.51E-10 | 1.52E-08 | 11.9717 |
| ZNF561 | 0.53041 | 4.134557 | 6.51996 | 7.62E-10 | 1.54E-08 | 11.9573 |
| CA4 | -1.0679 | 3.364087 | -6.5186 | 7.68E-10 | 1.55E-08 | 11.9504 |
| H3F3A | 0.50815 | 8.629763 | 6.51535 | 7.81E-10 | 1.57E-08 | 11.9334 |
| BRINP1 | -1.2381 | 4.209195 | -6.5137 | 7.88E-10 | 1.58E-08 | 11.9249 |
| ZIK1 | 0.52416 | 2.609016 | 6.51183 | 7.96E-10 | 1.60E-08 | 11.9151 |
| PNMAL2 | -0.936 | 3.301666 | -6.5089 | 8.09E-10 | 1.62E-08 | 11.8998 |
| RELL1 | 0.70359 | 3.092238 | 6.50754 | 8.15E-10 | 1.63E-08 | 11.8929 |
| HEATR2 | 0.61352 | 3.953188 | 6.50265 | 8.36E-10 | 1.67E-08 | 11.8675 |
| NT5DC2 | 0.74967 | 5.476423 | 6.50111 | 8.43E-10 | 1.68E-08 | 11.8596 |
| VAT1 | 0.55903 | 6.287151 | 6.49874 | 8.54E-10 | 1.70E-08 | 11.8473 |
| ALDH5A1 | -0.6925 | 5.188278 | -6.4944 | 8.74E-10 | 1.74E-08 | 11.8246 |
| NAT1 | 0.61917 | 1.529131 | 6.49345 | 8.78E-10 | 1.74E-08 | 11.8199 |
| TMED5 | 0.62829 | 5.099016 | 6.49181 | 8.86E-10 | 1.76E-08 | 11.8114 |
| RP11-434B12.1 | -0.8098 | 1.809511 | -6.4889 | 9.00E-10 | 1.78E-08 | 11.7963 |
| GBP1 | 1.36282 | 3.91951 | 6.4878 | 9.05E-10 | 1.79E-08 | 11.7906 |
| FCGBP | 1.55544 | 2.750295 | 6.48323 | 9.27E-10 | 1.83E-08 | 11.767 |
| SRF | 0.50313 | 4.275447 | 6.48246 | 9.31E-10 | 1.83E-08 | 11.763 |
| HTRA3 | 0.88306 | 1.091975 | 6.47943 | 9.46E-10 | 1.86E-08 | 11.7473 |
| ACTG1 | 0.50559 | 10.77653 | 6.47878 | 9.49E-10 | 1.86E-08 | 11.744 |
| ANXA1 | 1.80845 | 5.210158 | 6.47588 | 9.64E-10 | 1.89E-08 | 11.729 |
| TP73 | 0.76219 | 1.068581 | 6.4747 | 9.70E-10 | 1.89E-08 | 11.7228 |
| MT-ND1 | -0.6763 | 13.24071 | -6.4746 | 9.71E-10 | 1.89E-08 | 11.7225 |
| YBX3 | 0.85792 | 5.274688 | 6.47425 | 9.73E-10 | 1.89E-08 | 11.7205 |
| ANGPT2 | 1.19215 | 2.369444 | 6.47058 | 9.92E-10 | 1.92E-08 | 11.7016 |
| PODXL | 0.57972 | 4.893512 | 6.46645 | 1.01E-09 | 1.96E-08 | 11.6802 |
| MT-CO1 | -0.5018 | 14.65839 | -6.4637 | 1.03E-09 | 1.98E-08 | 11.666 |
| MYOF | 0.93198 | 2.908509 | 6.46206 | 1.04E-09 | 2.00E-08 | 11.6575 |
| C1orf95 | -0.9937 | 3.567842 | -6.4615 | 1.04E-09 | 2.00E-08 | 11.6548 |
| C1QTNF6 | 0.7366 | 2.456918 | 6.45653 | 1.07E-09 | 2.05E-08 | 11.629 |
| DTX3L | 0.76145 | 3.238476 | 6.44718 | 1.12E-09 | 2.14E-08 | 11.5808 |
| SNAPC2 | 0.57322 | 3.959068 | 6.4432 | 1.15E-09 | 2.18E-08 | 11.5603 |
| PANX2 | -0.9005 | 2.52856 | -6.443 | 1.15E-09 | 2.18E-08 | 11.5594 |
| RAB27A | 0.60356 | 2.414806 | 6.43932 | 1.17E-09 | 2.23E-08 | 11.5402 |
| TUBB6 | 1.01243 | 4.326747 | 6.43907 | 1.17E-09 | 2.23E-08 | 11.539 |
| AC005330.2 | -0.6534 | 1.944337 | -6.439 | 1.17E-09 | 2.23E-08 | 11.5384 |
| GEM | 1.14269 | 3.636533 | 6.43794 | 1.18E-09 | 2.24E-08 | 11.5331 |
| MFAP2 | 1.14373 | 1.540682 | 6.43399 | 1.20E-09 | 2.28E-08 | 11.5128 |
| PCDH12 | 0.71947 | 2.055293 | 6.43388 | 1.20E-09 | 2.28E-08 | 11.5122 |
| MSH5 | 0.69362 | 3.463151 | 6.43105 | 1.22E-09 | 2.31E-08 | 11.4977 |
| NRSN1 | -1.2505 | 4.668718 | -6.4287 | 1.24E-09 | 2.33E-08 | 11.4856 |
| C7orf41 | -0.897 | 7.300984 | -6.4284 | 1.24E-09 | 2.33E-08 | 11.4838 |
| RP11-690D19.3 | -0.5326 | 2.363741 | -6.4283 | 1.24E-09 | 2.33E-08 | 11.4837 |
| MT-ND5 | -0.5844 | 12.49035 | -6.4282 | 1.24E-09 | 2.33E-08 | 11.4827 |
| IFI30 | 1.44501 | 4.990131 | 6.42379 | 1.27E-09 | 2.38E-08 | 11.4603 |
| RP11-640M9.2 | 0.67174 | 3.459727 | 6.41508 | 1.33E-09 | 2.48E-08 | 11.4155 |
| HAR1A | -0.794 | 1.332086 | -6.4139 | 1.34E-09 | 2.49E-08 | 11.4096 |
| SLC12A5 | -1.1586 | 4.193866 | -6.4118 | 1.35E-09 | 2.51E-08 | 11.3984 |
| WNT5A | 0.87184 | 2.941971 | 6.41093 | 1.36E-09 | 2.51E-08 | 11.3942 |
| DYRK3 | 0.70093 | 2.257231 | 6.40713 | 1.39E-09 | 2.56E-08 | 11.3746 |
| MYO1G | 0.73161 | 1.137031 | 6.40592 | 1.40E-09 | 2.57E-08 | 11.3684 |
| MSH6 | 0.53567 | 4.613409 | 6.40387 | 1.41E-09 | 2.59E-08 | 11.3579 |
| MCM4 | 0.68957 | 5.098487 | 6.39881 | 1.45E-09 | 2.66E-08 | 11.3319 |
| RPS19 | 0.53948 | 10.22505 | 6.39414 | 1.49E-09 | 2.72E-08 | 11.308 |
| BTG1 | 0.53803 | 5.639436 | 6.39129 | 1.51E-09 | 2.76E-08 | 11.2933 |
| FAM132B | 0.8023 | 1.682768 | 6.39123 | 1.51E-09 | 2.76E-08 | 11.293 |
| KNDC1 | -0.956 | 3.308179 | -6.3763 | 1.63E-09 | 2.96E-08 | 11.2164 |
| OLFML3 | 0.86087 | 4.758784 | 6.37497 | 1.64E-09 | 2.97E-08 | 11.2097 |
| ECE1 | 0.7244 | 4.640213 | 6.37281 | 1.66E-09 | 3.00E-08 | 11.1986 |
| EXOC3L2 | 0.62255 | 0.486738 | 6.37225 | 1.67E-09 | 3.01E-08 | 11.1958 |
| SEC11A | 0.51236 | 6.630492 | 6.37038 | 1.69E-09 | 3.04E-08 | 11.1862 |
| HIST1H4H | 0.72999 | 2.058711 | 6.36906 | 1.70E-09 | 3.05E-08 | 11.1794 |
| DOCK11 | 0.57697 | 2.40358 | 6.36873 | 1.70E-09 | 3.05E-08 | 11.1777 |
| MTMR11 | 0.73404 | 3.255247 | 6.36582 | 1.73E-09 | 3.09E-08 | 11.1628 |
| RHOH | 0.64283 | 1.341451 | 6.36294 | 1.75E-09 | 3.14E-08 | 11.1481 |
| FLT4 | 0.66897 | 2.026188 | 6.36126 | 1.77E-09 | 3.16E-08 | 11.1395 |
| PRIM1 | 0.5284 | 3.183808 | 6.35996 | 1.78E-09 | 3.18E-08 | 11.1328 |
| LINC00641 | -0.6197 | 4.347481 | -6.3596 | 1.78E-09 | 3.18E-08 | 11.1308 |
| NR5A2 | 0.61284 | 0.815969 | 6.35826 | 1.80E-09 | 3.20E-08 | 11.1241 |
| TCF7 | 0.68729 | 2.147291 | 6.35776 | 1.80E-09 | 3.21E-08 | 11.1216 |
| AC099522.1 | 0.96242 | 1.944706 | 6.35531 | 1.82E-09 | 3.24E-08 | 11.1091 |
| NODAL | 0.74693 | 1.03747 | 6.35123 | 1.86E-09 | 3.31E-08 | 11.0882 |
| PLAT | 1.22618 | 3.39052 | 6.35103 | 1.87E-09 | 3.31E-08 | 11.0872 |
| PSME2 | 0.55928 | 6.259104 | 6.35037 | 1.87E-09 | 3.32E-08 | 11.0838 |
| SIPA1L3 | 0.61407 | 3.187557 | 6.3467 | 1.91E-09 | 3.37E-08 | 11.0651 |
| LAP3 | 0.65728 | 6.171507 | 6.34589 | 1.92E-09 | 3.38E-08 | 11.061 |
| DHFR | 0.59613 | 4.893773 | 6.34563 | 1.92E-09 | 3.39E-08 | 11.0596 |
| GATAD2A | 0.56263 | 5.174371 | 6.34069 | 1.97E-09 | 3.46E-08 | 11.0344 |
| CLCF1 | 0.95873 | 1.157595 | 6.33938 | 1.98E-09 | 3.49E-08 | 11.0277 |
| CACNA2D3 | -0.942 | 2.087042 | -6.3372 | 2.01E-09 | 3.52E-08 | 11.0167 |
| PTPRT | -1.1786 | 2.747056 | -6.3362 | 2.02E-09 | 3.53E-08 | 11.0115 |
| SLC6A1 | -0.8669 | 6.11378 | -6.3339 | 2.04E-09 | 3.57E-08 | 10.9997 |
| PLSCR1 | 0.94624 | 4.693449 | 6.33382 | 2.04E-09 | 3.57E-08 | 10.9993 |
| CD63 | 0.60194 | 9.146719 | 6.33369 | 2.04E-09 | 3.57E-08 | 10.9987 |
| MPZL1 | 0.67945 | 5.923383 | 6.33342 | 2.05E-09 | 3.57E-08 | 10.9973 |
| FAM115C | 0.70781 | 1.010876 | 6.33014 | 2.08E-09 | 3.62E-08 | 10.9806 |
| XPR1 | 0.54111 | 4.099046 | 6.32831 | 2.10E-09 | 3.65E-08 | 10.9713 |
| TMEM159 | 0.70059 | 2.516803 | 6.32668 | 2.12E-09 | 3.67E-08 | 10.9629 |
| FBXO2 | -1.3747 | 5.225041 | -6.3251 | 2.14E-09 | 3.70E-08 | 10.9548 |
| LRRN4CL | 1.02336 | 1.464111 | 6.32055 | 2.19E-09 | 3.79E-08 | 10.9317 |
| SNRPGP10 | 0.66264 | 4.549881 | 6.31862 | 2.21E-09 | 3.82E-08 | 10.9219 |
| FN3K | -0.5517 | 5.372357 | -6.3184 | 2.21E-09 | 3.82E-08 | 10.9208 |
| FZD1 | 0.56722 | 2.799616 | 6.31068 | 2.31E-09 | 3.96E-08 | 10.8815 |
| EDNRA | 0.94518 | 3.043336 | 6.30609 | 2.36E-09 | 4.05E-08 | 10.8581 |
| MFNG | 0.77083 | 3.445224 | 6.30356 | 2.39E-09 | 4.09E-08 | 10.8452 |
| CAP1 | 0.50071 | 7.037948 | 6.30148 | 2.42E-09 | 4.13E-08 | 10.8347 |
| ABCB4 | 0.58537 | 1.266687 | 6.30016 | 2.44E-09 | 4.16E-08 | 10.8279 |
| TIMP1 | 1.88427 | 5.293564 | 6.29969 | 2.44E-09 | 4.16E-08 | 10.8256 |
| TUBA1C | 1.11483 | 4.734267 | 6.29904 | 2.45E-09 | 4.17E-08 | 10.8222 |
| RFPL1S | -1.1785 | 2.473881 | -6.2969 | 2.48E-09 | 4.21E-08 | 10.8113 |
| NEFM | -1.9301 | 3.66429 | -6.294 | 2.52E-09 | 4.27E-08 | 10.7968 |
| MNX1 | 0.83469 | 0.799651 | 6.29215 | 2.54E-09 | 4.31E-08 | 10.7872 |
| MYO9B | 0.52771 | 5.647118 | 6.29112 | 2.55E-09 | 4.32E-08 | 10.782 |
| TREM1 | 1.32924 | 1.633343 | 6.28561 | 2.63E-09 | 4.44E-08 | 10.754 |
| CPEB1 | -0.6268 | 3.655037 | -6.2754 | 2.77E-09 | 4.66E-08 | 10.7021 |
| AGRN | 0.58662 | 5.852042 | 6.27247 | 2.82E-09 | 4.73E-08 | 10.6873 |
| GNG12 | 1.17742 | 3.943041 | 6.27198 | 2.82E-09 | 4.74E-08 | 10.6848 |
| RPS2P46 | 0.69846 | 2.816421 | 6.2673 | 2.89E-09 | 4.83E-08 | 10.6611 |
| C19orf55 | 0.64686 | 3.12558 | 6.26706 | 2.90E-09 | 4.84E-08 | 10.6599 |
| TP53INP1 | 0.56511 | 3.277325 | 6.26484 | 2.93E-09 | 4.88E-08 | 10.6487 |
| CYP27B1 | 0.89528 | 1.117915 | 6.26428 | 2.94E-09 | 4.89E-08 | 10.6458 |
| PHLDA2 | 0.90498 | 1.130199 | 6.26348 | 2.95E-09 | 4.91E-08 | 10.6418 |
| SCN2B | -1.0432 | 2.852433 | -6.262 | 2.97E-09 | 4.94E-08 | 10.6343 |
| PTX3 | 1.32946 | 1.8316 | 6.26086 | 2.99E-09 | 4.96E-08 | 10.6285 |
| HS2ST1 | 0.6021 | 4.045435 | 6.26073 | 2.99E-09 | 4.96E-08 | 10.6278 |
| GNB4 | 0.76393 | 4.447448 | 6.26026 | 3.00E-09 | 4.97E-08 | 10.6255 |
| SHANK2 | -0.9961 | 3.111016 | -6.2591 | 3.02E-09 | 5.00E-08 | 10.6195 |
| NEBL | -0.87 | 5.063613 | -6.2575 | 3.05E-09 | 5.04E-08 | 10.6113 |
| BTN3A1 | 0.66756 | 4.339171 | 6.25496 | 3.09E-09 | 5.10E-08 | 10.5986 |
| ZC3HAV1 | 0.54565 | 3.655368 | 6.25457 | 3.09E-09 | 5.10E-08 | 10.5966 |
| PRSS23 | 1.11211 | 4.372343 | 6.25263 | 3.12E-09 | 5.14E-08 | 10.5868 |
| AKAP2 | 0.70848 | 3.55485 | 6.25141 | 3.14E-09 | 5.17E-08 | 10.5807 |
| STC1 | 1.01628 | 0.943824 | 6.25035 | 3.16E-09 | 5.19E-08 | 10.5753 |
| COL6A3 | 1.06928 | 1.518257 | 6.24773 | 3.20E-09 | 5.25E-08 | 10.562 |
| EMR1 | 0.80124 | 0.929915 | 6.24543 | 3.24E-09 | 5.31E-08 | 10.5504 |
| PRR16 | 0.76596 | 1.45426 | 6.24516 | 3.25E-09 | 5.31E-08 | 10.549 |
| MRO | -0.9665 | 4.087582 | -6.2436 | 3.27E-09 | 5.35E-08 | 10.5413 |
| H6PD | 0.55903 | 3.283421 | 6.24325 | 3.28E-09 | 5.36E-08 | 10.5394 |
| JAKMIP1 | -1.1319 | 2.970803 | -6.2431 | 3.28E-09 | 5.36E-08 | 10.5384 |
| TM4SF1 | 1.0896 | 3.840698 | 6.24097 | 3.32E-09 | 5.42E-08 | 10.5279 |
| DOT1L | 0.66132 | 3.929553 | 6.23771 | 3.38E-09 | 5.50E-08 | 10.5114 |
| RP11-47I22.1 | -1.4494 | 2.178892 | -6.2358 | 3.41E-09 | 5.55E-08 | 10.5019 |
| TOR4A | 0.55177 | 1.643515 | 6.22737 | 3.56E-09 | 5.78E-08 | 10.4591 |
| PYGL | 0.98866 | 3.889086 | 6.22708 | 3.57E-09 | 5.78E-08 | 10.4577 |
| HN1 | 0.65954 | 6.836876 | 6.22509 | 3.60E-09 | 5.84E-08 | 10.4477 |
| PDLIM1 | 1.20868 | 2.995162 | 6.22174 | 3.67E-09 | 5.93E-08 | 10.4308 |
| CBX2 | 0.75317 | 2.439265 | 6.22135 | 3.68E-09 | 5.94E-08 | 10.4288 |
| SLC29A1 | 0.59114 | 4.315223 | 6.22109 | 3.68E-09 | 5.94E-08 | 10.4275 |
| COL6A2 | 1.38216 | 3.967817 | 6.21975 | 3.71E-09 | 5.97E-08 | 10.4207 |
| SNCG | -1.4869 | 5.056028 | -6.2186 | 3.73E-09 | 6.00E-08 | 10.4148 |
| FOXS1 | 0.75216 | 1.529206 | 6.21853 | 3.73E-09 | 6.00E-08 | 10.4145 |
| CRY2 | -0.6588 | 4.988714 | -6.2173 | 3.75E-09 | 6.02E-08 | 10.4082 |
| DHRSX | 0.50957 | 3.09086 | 6.21231 | 3.85E-09 | 6.15E-08 | 10.3832 |
| SLC24A2 | -1.1306 | 3.074331 | -6.2078 | 3.94E-09 | 6.29E-08 | 10.3604 |
| RTN1 | -1.1622 | 7.730623 | -6.1989 | 4.13E-09 | 6.57E-08 | 10.3158 |
| EN1 | 1.00948 | 0.821862 | 6.19816 | 4.15E-09 | 6.59E-08 | 10.3119 |
| IL1RAP | 0.9883 | 3.076469 | 6.19793 | 4.15E-09 | 6.60E-08 | 10.3108 |
| IGIP | -0.5922 | 4.568158 | -6.1935 | 4.25E-09 | 6.74E-08 | 10.2885 |
| SERPINI1 | -1.4121 | 5.111087 | -6.193 | 4.26E-09 | 6.75E-08 | 10.2861 |
| SRPX2 | 1.16984 | 1.668883 | 6.18906 | 4.35E-09 | 6.88E-08 | 10.2662 |
| AK5 | -1.4859 | 4.183829 | -6.1843 | 4.45E-09 | 7.04E-08 | 10.2422 |
| CIITA | 1.03518 | 2.432616 | 6.18306 | 4.48E-09 | 7.08E-08 | 10.236 |
| SMC6 | 0.52789 | 3.303231 | 6.18112 | 4.53E-09 | 7.14E-08 | 10.2262 |
| ING3 | 0.50923 | 4.208706 | 6.17324 | 4.72E-09 | 7.43E-08 | 10.1867 |
| FAM20C | 0.9036 | 4.872547 | 6.1718 | 4.75E-09 | 7.47E-08 | 10.1795 |
| EHD4 | 0.6644 | 3.02868 | 6.17039 | 4.79E-09 | 7.52E-08 | 10.1724 |
| PTPN9 | 0.50399 | 4.128204 | 6.16994 | 4.80E-09 | 7.53E-08 | 10.1701 |
| SMIM4 | 0.59914 | 4.423352 | 6.16787 | 4.85E-09 | 7.61E-08 | 10.1597 |
| JAKMIP3 | -0.707 | 1.945563 | -6.1677 | 4.85E-09 | 7.61E-08 | 10.159 |
| GMIP | 0.54685 | 3.5771 | 6.1612 | 5.02E-09 | 7.84E-08 | 10.1263 |
| ANXA2 | 1.42573 | 5.968817 | 6.15829 | 5.10E-09 | 7.94E-08 | 10.1117 |
| EMP1 | 1.30024 | 5.016288 | 6.15568 | 5.16E-09 | 8.04E-08 | 10.0986 |
| SP140L | 0.71335 | 2.185487 | 6.15551 | 5.17E-09 | 8.04E-08 | 10.0978 |
| MYL3 | -0.7755 | 2.396983 | -6.1554 | 5.17E-09 | 8.04E-08 | 10.0972 |
| PTP4A3 | 0.76693 | 4.764263 | 6.15414 | 5.21E-09 | 8.08E-08 | 10.0909 |
| SH3PXD2B | 0.59759 | 3.860217 | 6.1514 | 5.28E-09 | 8.19E-08 | 10.0772 |
| DOK3 | 0.74222 | 2.963151 | 6.14814 | 5.37E-09 | 8.31E-08 | 10.0608 |
| PLAU | 1.36639 | 2.631763 | 6.14719 | 5.40E-09 | 8.35E-08 | 10.0561 |
| RP1-257A7.5 | -0.8288 | 4.485872 | -6.1467 | 5.41E-09 | 8.36E-08 | 10.0538 |
| STRA6 | 0.70326 | 1.282906 | 6.14505 | 5.46E-09 | 8.43E-08 | 10.0454 |
| MATK | -0.9926 | 2.605586 | -6.1445 | 5.47E-09 | 8.44E-08 | 10.0425 |
| RPS2P5 | 0.86496 | 5.136816 | 6.14342 | 5.50E-09 | 8.48E-08 | 10.0372 |
| METTL1 | 0.94524 | 4.114492 | 6.14116 | 5.57E-09 | 8.56E-08 | 10.0259 |
| SPRY1 | 1.02523 | 3.427493 | 6.14101 | 5.57E-09 | 8.56E-08 | 10.0252 |
| BCL3 | 0.87254 | 2.69863 | 6.14029 | 5.59E-09 | 8.59E-08 | 10.0215 |
| DONSON | 0.50597 | 3.828134 | 6.13977 | 5.61E-09 | 8.60E-08 | 10.0189 |
| TUBA1A | 0.55868 | 10.56789 | 6.13946 | 5.62E-09 | 8.61E-08 | 10.0174 |
| ENHO | -1.1238 | 7.000636 | -6.1394 | 5.62E-09 | 8.61E-08 | 10.0172 |
| RBFOX3 | -1.4538 | 2.813533 | -6.1391 | 5.63E-09 | 8.62E-08 | 10.0154 |
| AIM1 | 0.65546 | 0.974432 | 6.13827 | 5.65E-09 | 8.65E-08 | 10.0114 |
| FBL | 0.52643 | 6.671911 | 6.1368 | 5.69E-09 | 8.70E-08 | 10.0041 |
| PARP9 | 0.77615 | 4.263996 | 6.13384 | 5.78E-09 | 8.82E-08 | 9.98927 |
| RP2 | 0.53012 | 3.25803 | 6.13279 | 5.81E-09 | 8.86E-08 | 9.98402 |
| PTGDS | -1.3661 | 9.478504 | -6.1318 | 5.84E-09 | 8.89E-08 | 9.97902 |
| MAPK8IP2 | -0.905 | 5.314444 | -6.1293 | 5.92E-09 | 8.98E-08 | 9.96654 |
| HOXD4 | 0.84606 | 0.964041 | 6.12864 | 5.94E-09 | 9.01E-08 | 9.9633 |
| ELK3 | 0.68924 | 3.427154 | 6.12629 | 6.01E-09 | 9.11E-08 | 9.95154 |
| TMEM151B | -1.2254 | 3.874583 | -6.1234 | 6.10E-09 | 9.23E-08 | 9.93691 |
| EXOC3L1 | 0.5253 | 1.519321 | 6.12203 | 6.14E-09 | 9.28E-08 | 9.93026 |
| FES | 0.59233 | 2.804177 | 6.12071 | 6.18E-09 | 9.34E-08 | 9.92369 |
| METTL7B | 1.44152 | 3.019085 | 6.11761 | 6.28E-09 | 9.48E-08 | 9.90822 |
| PNMA3 | -0.966 | 2.738867 | -6.1167 | 6.31E-09 | 9.51E-08 | 9.90389 |
| AC123886.2 | -0.768 | 1.028519 | -6.1155 | 6.35E-09 | 9.57E-08 | 9.89768 |
| HAND2 | 0.98394 | 0.906908 | 6.11072 | 6.51E-09 | 9.80E-08 | 9.87382 |
| GRIN1 | -1.7969 | 4.041773 | -6.1084 | 6.59E-09 | 9.91E-08 | 9.86234 |
| TEF | -0.714 | 4.454099 | -6.1072 | 6.63E-09 | 9.96E-08 | 9.8562 |
| RUNX2 | 0.56553 | 1.529911 | 6.10217 | 6.80E-09 | 1.02E-07 | 9.83119 |
| CACNA1I | -0.6969 | 1.196906 | -6.1016 | 6.82E-09 | 1.02E-07 | 9.82835 |
| PPP1R1B | -1.234 | 5.162504 | -6.0993 | 6.90E-09 | 1.03E-07 | 9.81709 |
| CRYZ | 0.66945 | 4.17554 | 6.09823 | 6.94E-09 | 1.04E-07 | 9.81157 |
| P2RY6 | 0.77438 | 1.753757 | 6.0979 | 6.95E-09 | 1.04E-07 | 9.80991 |
| TMEM39A | 0.51 | 3.468103 | 6.09706 | 6.98E-09 | 1.04E-07 | 9.80574 |
| VAV2 | 0.53987 | 3.979006 | 6.09534 | 7.05E-09 | 1.05E-07 | 9.79719 |
| CAMK2G | -0.7083 | 5.917931 | -6.0931 | 7.13E-09 | 1.06E-07 | 9.78626 |
| FAM60A | 0.80069 | 3.707004 | 6.09142 | 7.19E-09 | 1.07E-07 | 9.77769 |
| RAP1GAP2 | -0.9059 | 2.914664 | -6.0908 | 7.21E-09 | 1.07E-07 | 9.77477 |
| EFNA4 | 0.54956 | 1.690606 | 6.09018 | 7.24E-09 | 1.07E-07 | 9.77149 |
| CDK11A | 0.51277 | 4.857029 | 6.08993 | 7.24E-09 | 1.07E-07 | 9.77024 |
| ALDOC | -1.1385 | 9.163057 | -6.0883 | 7.31E-09 | 1.08E-07 | 9.76198 |
| WISP1 | 0.79393 | 1.085053 | 6.0869 | 7.36E-09 | 1.09E-07 | 9.75516 |
| HOXA4 | 1.04932 | 0.730779 | 6.08664 | 7.37E-09 | 1.09E-07 | 9.75387 |
| CYP17A1-AS1 | -0.8294 | 1.847767 | -6.0847 | 7.44E-09 | 1.10E-07 | 9.74439 |
| NDE1 | 0.67277 | 4.133844 | 6.08123 | 7.58E-09 | 1.12E-07 | 9.72696 |
| ZNF93 | 0.62361 | 2.675105 | 6.08057 | 7.60E-09 | 1.12E-07 | 9.72367 |
| PPP4R1L | 0.54464 | 2.163012 | 6.08053 | 7.60E-09 | 1.12E-07 | 9.72347 |
| EPS8 | 0.54241 | 4.49424 | 6.07989 | 7.63E-09 | 1.12E-07 | 9.72032 |
| C4orf21 | 0.52187 | 2.047646 | 6.07933 | 7.65E-09 | 1.12E-07 | 9.71751 |
| LINC00634 | -0.8531 | 4.463891 | -6.0784 | 7.69E-09 | 1.13E-07 | 9.71275 |
| ARAP3 | 0.83946 | 2.54844 | 6.0778 | 7.71E-09 | 1.13E-07 | 9.7099 |
| PRRT1 | -0.6891 | 4.597154 | -6.0776 | 7.72E-09 | 1.13E-07 | 9.70868 |
| ANG | 0.79335 | 1.877923 | 6.07477 | 7.83E-09 | 1.15E-07 | 9.69487 |
| HOXA10 | 1.15466 | 0.964724 | 6.07405 | 7.86E-09 | 1.15E-07 | 9.69127 |
| RAC2 | 0.845 | 2.793571 | 6.07391 | 7.86E-09 | 1.15E-07 | 9.69056 |
| RP11-423G4.7 | -0.7728 | 1.139269 | -6.0736 | 7.88E-09 | 1.15E-07 | 9.68882 |
| SNAP25 | -1.8252 | 6.832573 | -6.072 | 7.94E-09 | 1.16E-07 | 9.68128 |
| CCR5 | 0.78915 | 1.339655 | 6.072 | 7.94E-09 | 1.16E-07 | 9.68108 |
| NECAP2 | 0.60618 | 5.449165 | 6.06918 | 8.06E-09 | 1.17E-07 | 9.66708 |
| LHPP | -1.0096 | 6.235385 | -6.0683 | 8.09E-09 | 1.18E-07 | 9.66289 |
| HTR2A | -0.9116 | 1.877723 | -6.0681 | 8.10E-09 | 1.18E-07 | 9.66181 |
| HIST1H3E | 0.73561 | 1.042982 | 6.06788 | 8.11E-09 | 1.18E-07 | 9.66065 |
| MEST | 0.92389 | 5.497567 | 6.06567 | 8.20E-09 | 1.19E-07 | 9.64964 |
| ZNF217 | 0.61175 | 2.411667 | 6.06505 | 8.23E-09 | 1.19E-07 | 9.64661 |
| FOXD1 | 0.7401 | 0.985834 | 6.06132 | 8.39E-09 | 1.21E-07 | 9.62807 |
| TPPP | -1.194 | 5.199933 | -6.0583 | 8.52E-09 | 1.23E-07 | 9.61311 |
| CD97 | 0.76339 | 3.403825 | 6.05804 | 8.53E-09 | 1.23E-07 | 9.61181 |
| TNPO1 | 0.51937 | 5.326486 | 6.05758 | 8.55E-09 | 1.23E-07 | 9.6095 |
| FAM114A1 | 0.82173 | 3.171046 | 6.05677 | 8.59E-09 | 1.24E-07 | 9.60549 |
| PPP1R13B | -0.5605 | 3.425748 | -6.0559 | 8.62E-09 | 1.24E-07 | 9.60109 |
| EPHX1 | -0.5223 | 6.513062 | -6.0545 | 8.68E-09 | 1.25E-07 | 9.59442 |
| HIST1H3D | 0.63942 | 0.904728 | 6.05166 | 8.81E-09 | 1.26E-07 | 9.58016 |
| ZNF107 | 0.50189 | 2.823088 | 6.04563 | 9.09E-09 | 1.30E-07 | 9.5503 |
| EXT1 | 0.51134 | 3.731287 | 6.04329 | 9.20E-09 | 1.31E-07 | 9.5387 |
| NABP1 | 0.75772 | 2.80123 | 6.039 | 9.40E-09 | 1.34E-07 | 9.51743 |
| GALNT4 | 0.55837 | 1.608741 | 6.03792 | 9.45E-09 | 1.35E-07 | 9.5121 |
| LINC00320 | -1.1478 | 3.586173 | -6.0316 | 9.76E-09 | 1.38E-07 | 9.48107 |
| SHC1 | 0.65164 | 4.894292 | 6.03034 | 9.83E-09 | 1.39E-07 | 9.47462 |
| RAB32 | 0.8538 | 3.034162 | 6.02921 | 9.88E-09 | 1.40E-07 | 9.469 |
| RRM1 | 0.55538 | 4.667401 | 6.02848 | 9.92E-09 | 1.40E-07 | 9.46542 |
| EIF2AK3 | 0.52715 | 3.953541 | 6.02698 | 1.00E-08 | 1.41E-07 | 9.45801 |
| GRAP | 0.56545 | 2.079717 | 6.02444 | 1.01E-08 | 1.43E-07 | 9.44542 |
| RP11-66N24.3 | 0.53487 | 1.728787 | 6.02389 | 1.02E-08 | 1.43E-07 | 9.4427 |
| CREB1 | 0.52987 | 4.298158 | 6.02347 | 1.02E-08 | 1.43E-07 | 9.44063 |
| CPSF6 | 0.54224 | 4.978455 | 6.0199 | 1.04E-08 | 1.46E-07 | 9.42302 |
| CPE | -0.8863 | 9.667947 | -6.0194 | 1.04E-08 | 1.46E-07 | 9.42056 |
| SYN1 | -1.411 | 4.749872 | -6.015 | 1.06E-08 | 1.49E-07 | 9.39893 |
| HOXA3 | 0.90209 | 0.674577 | 6.0148 | 1.06E-08 | 1.49E-07 | 9.39783 |
| APOBEC3B | 0.6359 | 0.97465 | 6.01031 | 1.09E-08 | 1.52E-07 | 9.37566 |
| TMEM255A | 0.92958 | 4.313023 | 6.00616 | 1.11E-08 | 1.55E-07 | 9.35518 |
| STXBP6 | -0.9083 | 3.446302 | -6.0057 | 1.11E-08 | 1.55E-07 | 9.35305 |
| ANXA5 | 0.65802 | 7.723535 | 6.00304 | 1.13E-08 | 1.57E-07 | 9.3398 |
| CD96 | 0.64294 | 0.782207 | 6.00271 | 1.13E-08 | 1.58E-07 | 9.33818 |
| DLL4 | 0.68888 | 2.017396 | 6.00212 | 1.13E-08 | 1.58E-07 | 9.33524 |
| RGS20 | -0.9659 | 2.994252 | -6.001 | 1.14E-08 | 1.59E-07 | 9.32957 |
| STAC3 | 0.60033 | 2.122424 | 6.00066 | 1.14E-08 | 1.59E-07 | 9.32805 |
| TYMP | 1.27059 | 3.813007 | 6.00003 | 1.15E-08 | 1.59E-07 | 9.32494 |
| SP100 | 0.81626 | 3.93773 | 5.99912 | 1.15E-08 | 1.60E-07 | 9.32045 |
| TNFRSF4 | 0.61587 | 1.116036 | 5.99634 | 1.17E-08 | 1.62E-07 | 9.30676 |
| EVA1B | 0.73445 | 3.602761 | 5.99631 | 1.17E-08 | 1.62E-07 | 9.30664 |
| SAMD9L | 0.8517 | 2.868556 | 5.99599 | 1.17E-08 | 1.62E-07 | 9.30503 |
| EMILIN1 | 0.93535 | 3.456164 | 5.99143 | 1.20E-08 | 1.65E-07 | 9.28259 |
| TMEM2 | 0.66731 | 3.319695 | 5.98611 | 1.23E-08 | 1.69E-07 | 9.25636 |
| CROT | 0.59153 | 3.776482 | 5.98565 | 1.23E-08 | 1.69E-07 | 9.25413 |
| MAP7 | -1.0015 | 4.111161 | -5.9835 | 1.25E-08 | 1.71E-07 | 9.24336 |
| ATP9A | -0.6867 | 6.133852 | -5.983 | 1.25E-08 | 1.71E-07 | 9.2412 |
| PLA2G4A | 0.66682 | 2.190503 | 5.98261 | 1.25E-08 | 1.71E-07 | 9.23913 |
| KLHL17 | 0.59873 | 3.347886 | 5.98145 | 1.26E-08 | 1.72E-07 | 9.23345 |
| HDAC11 | -0.646 | 5.182386 | -5.9806 | 1.27E-08 | 1.72E-07 | 9.22923 |
| KCNE3 | 0.74087 | 1.97486 | 5.97967 | 1.27E-08 | 1.73E-07 | 9.22471 |
| MAP2K3 | 0.58301 | 3.377 | 5.97866 | 1.28E-08 | 1.74E-07 | 9.21974 |
| FAM26F | 0.97424 | 2.499095 | 5.97073 | 1.33E-08 | 1.80E-07 | 9.18076 |
| ARRB1 | -0.7434 | 4.461381 | -5.9649 | 1.37E-08 | 1.86E-07 | 9.15187 |
| CARD6 | 0.53058 | 1.740636 | 5.96437 | 1.37E-08 | 1.86E-07 | 9.14951 |
| ODC1 | 0.6218 | 6.111956 | 5.96386 | 1.38E-08 | 1.86E-07 | 9.147 |
| HNRNPH1 | 0.52506 | 7.948905 | 5.9623 | 1.39E-08 | 1.88E-07 | 9.13934 |
| CD58 | 0.82131 | 2.955325 | 5.9585 | 1.42E-08 | 1.91E-07 | 9.12068 |
| APBB1 | -0.5108 | 6.884784 | -5.9554 | 1.44E-08 | 1.93E-07 | 9.10551 |
| ALDH6A1 | -0.6547 | 5.509221 | -5.9538 | 1.45E-08 | 1.95E-07 | 9.09747 |
| PLVAP | 1.03906 | 3.05014 | 5.94921 | 1.48E-08 | 1.99E-07 | 9.07513 |
| TIFA | 0.6375 | 1.914301 | 5.94801 | 1.49E-08 | 2.00E-07 | 9.06924 |
| PACSIN1 | -1.8253 | 3.462276 | -5.948 | 1.49E-08 | 2.00E-07 | 9.06921 |
| RSU1 | 0.53129 | 5.647015 | 5.94619 | 1.51E-08 | 2.01E-07 | 9.0603 |
| DGCR6 | -1.1212 | 5.839821 | -5.9426 | 1.53E-08 | 2.04E-07 | 9.04297 |
| HRH3 | -1.1493 | 2.150226 | -5.9407 | 1.55E-08 | 2.06E-07 | 9.03332 |
| TTYH3 | 0.61833 | 5.718152 | 5.93915 | 1.56E-08 | 2.08E-07 | 9.02581 |
| OSR2 | 0.82116 | 0.817975 | 5.93841 | 1.57E-08 | 2.08E-07 | 9.02221 |
| CREB3L2 | 0.50886 | 3.188232 | 5.93664 | 1.58E-08 | 2.09E-07 | 9.01355 |
| CLEC11A | 0.52356 | 3.097687 | 5.93621 | 1.58E-08 | 2.10E-07 | 9.01142 |
| CFI | 1.31887 | 3.567609 | 5.9345 | 1.60E-08 | 2.11E-07 | 9.00307 |
| KCNK13 | 0.54706 | 1.392356 | 5.93113 | 1.63E-08 | 2.14E-07 | 8.98659 |
| NAMPTL | 0.90647 | 1.763399 | 5.92882 | 1.65E-08 | 2.17E-07 | 8.97525 |
| RELB | 0.67108 | 2.739846 | 5.92845 | 1.65E-08 | 2.17E-07 | 8.97348 |
| GPC1 | 0.58973 | 6.051405 | 5.92293 | 1.69E-08 | 2.23E-07 | 8.94648 |
| ANXA2R | 0.55022 | 1.403407 | 5.9225 | 1.70E-08 | 2.23E-07 | 8.94436 |
| CAMK2A | -1.9166 | 4.250693 | -5.922 | 1.70E-08 | 2.23E-07 | 8.94182 |
| TSKU | 0.53067 | 3.389353 | 5.92011 | 1.72E-08 | 2.25E-07 | 8.93268 |
| ADHFE1 | -0.6938 | 4.607233 | -5.9161 | 1.75E-08 | 2.29E-07 | 8.91328 |
| TNIP2 | 0.57827 | 4.620787 | 5.91394 | 1.77E-08 | 2.32E-07 | 8.90256 |
| HHATL | -1.2782 | 4.6705 | -5.913 | 1.78E-08 | 2.33E-07 | 8.8981 |
| FAM46C | 0.51942 | 0.893216 | 5.91224 | 1.79E-08 | 2.34E-07 | 8.89425 |
| SNCB | -1.6709 | 5.084756 | -5.9115 | 1.80E-08 | 2.34E-07 | 8.89049 |
| EPHB2 | 0.7717 | 3.059289 | 5.90878 | 1.82E-08 | 2.37E-07 | 8.87737 |
| NAP1L2 | -0.9527 | 4.133293 | -5.9083 | 1.82E-08 | 2.37E-07 | 8.87489 |
| PLCE1 | 0.65648 | 2.687993 | 5.90606 | 1.85E-08 | 2.40E-07 | 8.86409 |
| C12orf5 | 0.51705 | 3.791732 | 5.90311 | 1.87E-08 | 2.43E-07 | 8.84971 |
| MAML1 | 0.51879 | 3.864828 | 5.89875 | 1.91E-08 | 2.48E-07 | 8.82844 |
| NOD2 | 0.58018 | 1.130802 | 5.89627 | 1.94E-08 | 2.51E-07 | 8.81634 |
| SEC61G | 0.99572 | 7.028991 | 5.89349 | 1.97E-08 | 2.54E-07 | 8.80279 |
| CAV2 | 0.86275 | 3.174728 | 5.89016 | 2.00E-08 | 2.58E-07 | 8.7866 |
| HIST1H2BD | 0.78848 | 3.19886 | 5.88935 | 2.01E-08 | 2.58E-07 | 8.78265 |
| P4HA3 | 0.68093 | 0.806048 | 5.88859 | 2.01E-08 | 2.59E-07 | 8.77891 |
| VSTM2L | -1.2828 | 3.551544 | -5.8859 | 2.04E-08 | 2.62E-07 | 8.76596 |
| MLKL | 0.53473 | 1.880992 | 5.88567 | 2.04E-08 | 2.62E-07 | 8.76473 |
| SERINC2 | 0.86076 | 1.666317 | 5.88434 | 2.06E-08 | 2.64E-07 | 8.75825 |
| HOXD11 | 0.85219 | 0.585237 | 5.88355 | 2.07E-08 | 2.65E-07 | 8.75438 |
| GADD45A | 1.02058 | 5.163129 | 5.88061 | 2.10E-08 | 2.68E-07 | 8.74011 |
| PPIC | 0.75919 | 2.813765 | 5.87891 | 2.12E-08 | 2.70E-07 | 8.73181 |
| CNTNAP2 | -0.992 | 3.369158 | -5.8775 | 2.13E-08 | 2.72E-07 | 8.72491 |
| TUBB4A | -1.2024 | 7.357855 | -5.8773 | 2.13E-08 | 2.72E-07 | 8.72384 |
| PAQR6 | -0.9165 | 6.949248 | -5.8771 | 2.13E-08 | 2.72E-07 | 8.72325 |
| ADAT3 | 0.5059 | 1.926825 | 5.87702 | 2.14E-08 | 2.72E-07 | 8.72261 |
| SLCO1A2 | -1.1249 | 3.840788 | -5.877 | 2.14E-08 | 2.72E-07 | 8.72238 |
| UBASH3B | 0.55736 | 1.908436 | 5.87381 | 2.17E-08 | 2.76E-07 | 8.70703 |
| UACA | 0.60797 | 3.627981 | 5.87209 | 2.19E-08 | 2.78E-07 | 8.69864 |
| TRPM8 | 0.84554 | 0.918273 | 5.86716 | 2.24E-08 | 2.84E-07 | 8.67472 |
| MTATP6P1 | -0.5177 | 11.4808 | -5.8657 | 2.26E-08 | 2.86E-07 | 8.6674 |
| USH1C | -1.6147 | 4.197406 | -5.8652 | 2.27E-08 | 2.86E-07 | 8.66522 |
| FILIP1L | 0.91033 | 3.38575 | 5.86292 | 2.29E-08 | 2.89E-07 | 8.65411 |
| CES4A | -0.9838 | 2.745109 | -5.8624 | 2.30E-08 | 2.90E-07 | 8.65177 |
| FSTL5 | -1.0976 | 2.287161 | -5.8582 | 2.35E-08 | 2.95E-07 | 8.63138 |
| MRVI1 | -0.8362 | 4.533006 | -5.856 | 2.37E-08 | 2.98E-07 | 8.62038 |
| CTHRC1 | 1.05234 | 2.048322 | 5.85322 | 2.41E-08 | 3.02E-07 | 8.60702 |
| GPR84 | 0.70024 | 1.02423 | 5.85248 | 2.41E-08 | 3.03E-07 | 8.60347 |
| CD72 | 0.63174 | 2.001884 | 5.85113 | 2.43E-08 | 3.04E-07 | 8.59689 |
| EPS8L1 | 0.55551 | 1.196457 | 5.85069 | 2.44E-08 | 3.05E-07 | 8.59478 |
| C1orf85 | 0.57803 | 4.494759 | 5.84962 | 2.45E-08 | 3.06E-07 | 8.5896 |
| RP11-617F23.1 | -0.5467 | 3.882482 | -5.8489 | 2.46E-08 | 3.07E-07 | 8.58629 |
| AC007620.3 | 0.72548 | 4.285449 | 5.84693 | 2.48E-08 | 3.09E-07 | 8.57653 |
| FLJ30594 | -0.9522 | 3.366121 | -5.8427 | 2.54E-08 | 3.15E-07 | 8.55593 |
| PLEKHS1 | 0.79352 | 0.609002 | 5.84115 | 2.56E-08 | 3.17E-07 | 8.54855 |
| FAM81A | -0.8501 | 3.354225 | -5.8399 | 2.57E-08 | 3.19E-07 | 8.54226 |
| SCD | -0.8895 | 8.258599 | -5.8395 | 2.58E-08 | 3.19E-07 | 8.5405 |
| CASP8 | 0.65815 | 2.399818 | 5.83856 | 2.59E-08 | 3.20E-07 | 8.53599 |
| VEGFA | 1.32965 | 5.546828 | 5.83827 | 2.59E-08 | 3.20E-07 | 8.53459 |
| LAMB2 | 0.65463 | 6.595452 | 5.83319 | 2.66E-08 | 3.28E-07 | 8.51001 |
| ATF5 | 0.67767 | 3.932052 | 5.83314 | 2.66E-08 | 3.28E-07 | 8.50975 |
| RAB42 | 0.74343 | 1.414496 | 5.83283 | 2.66E-08 | 3.28E-07 | 8.50824 |
| MYRIP | -0.7712 | 2.84998 | -5.8326 | 2.67E-08 | 3.28E-07 | 8.50722 |
| MMP19 | 0.95352 | 1.467977 | 5.83085 | 2.69E-08 | 3.31E-07 | 8.49866 |
| CORO6 | -0.875 | 2.981519 | -5.8306 | 2.69E-08 | 3.31E-07 | 8.49765 |
| PLAC8 | 0.86077 | 1.340893 | 5.83016 | 2.70E-08 | 3.31E-07 | 8.49534 |
| SYP | -1.0107 | 6.355822 | -5.8278 | 2.73E-08 | 3.35E-07 | 8.48376 |
| TAP1 | 0.73063 | 5.050285 | 5.82734 | 2.74E-08 | 3.35E-07 | 8.48171 |
| RP11-153M7.1 | 0.591 | 0.85678 | 5.82489 | 2.77E-08 | 3.39E-07 | 8.46985 |
| KLHL32 | -0.8121 | 3.695432 | -5.8189 | 2.86E-08 | 3.48E-07 | 8.44103 |
| GRIN2C | -0.8519 | 2.947278 | -5.8176 | 2.87E-08 | 3.50E-07 | 8.43463 |
| HGF | 0.87933 | 1.97216 | 5.81644 | 2.89E-08 | 3.52E-07 | 8.42902 |
| PPP1R1A | -1.0654 | 3.992779 | -5.8151 | 2.91E-08 | 3.53E-07 | 8.42266 |
| ARTN | 0.5524 | 0.993956 | 5.80853 | 3.01E-08 | 3.64E-07 | 8.39083 |
| CASP1 | 0.82615 | 3.748814 | 5.80671 | 3.04E-08 | 3.66E-07 | 8.38204 |
| CTDSP2 | 0.653 | 6.192753 | 5.80435 | 3.07E-08 | 3.70E-07 | 8.37069 |
| IGFLR1 | 0.63877 | 3.391506 | 5.80347 | 3.08E-08 | 3.72E-07 | 8.36644 |
| CCND1 | 0.93281 | 5.120446 | 5.80088 | 3.12E-08 | 3.76E-07 | 8.35393 |
| CMTM1 | 0.61967 | 2.917334 | 5.80079 | 3.13E-08 | 3.76E-07 | 8.35351 |
| CA11 | -0.964 | 5.829693 | -5.8004 | 3.13E-08 | 3.76E-07 | 8.35177 |
| UGCG | 0.59053 | 3.55677 | 5.80038 | 3.13E-08 | 3.76E-07 | 8.35151 |
| MSN | 0.9738 | 5.797573 | 5.79731 | 3.18E-08 | 3.81E-07 | 8.33671 |
| IRF1 | 0.87742 | 3.805533 | 5.79564 | 3.21E-08 | 3.83E-07 | 8.3287 |
| CARD16 | 0.96231 | 3.098061 | 5.79502 | 3.22E-08 | 3.84E-07 | 8.32569 |
| CITED1 | 0.95154 | 3.648243 | 5.79321 | 3.25E-08 | 3.88E-07 | 8.31698 |
| FANCG | 0.50199 | 4.076317 | 5.79295 | 3.25E-08 | 3.88E-07 | 8.31571 |
| CTD-2510F5.4 | 0.66904 | 1.272511 | 5.7924 | 3.26E-08 | 3.89E-07 | 8.31305 |
| NPY | -1.578 | 4.584362 | -5.7919 | 3.27E-08 | 3.90E-07 | 8.31046 |
| RP11-513M16.7 | -0.7608 | 2.558318 | -5.7903 | 3.29E-08 | 3.92E-07 | 8.30298 |
| LY6H | -1.3053 | 5.141616 | -5.7891 | 3.31E-08 | 3.94E-07 | 8.29709 |
| DPY19L1 | 0.66973 | 4.449761 | 5.78723 | 3.34E-08 | 3.97E-07 | 8.28817 |
| CALN1 | -1.2063 | 3.050463 | -5.7864 | 3.36E-08 | 3.99E-07 | 8.28414 |
| MREG | 0.7198 | 2.985374 | 5.78618 | 3.36E-08 | 3.99E-07 | 8.28312 |
| KCNN1 | -0.9256 | 3.157784 | -5.7858 | 3.37E-08 | 3.99E-07 | 8.28142 |
| LIF | 1.00433 | 1.36166 | 5.78543 | 3.37E-08 | 4.00E-07 | 8.27951 |
| WSCD2 | -0.9904 | 1.958589 | -5.7854 | 3.38E-08 | 4.00E-07 | 8.2792 |
| ALDH1A1 | -1.1408 | 4.767388 | -5.7835 | 3.41E-08 | 4.03E-07 | 8.27012 |
| TMEM217 | 0.59098 | 1.395336 | 5.78285 | 3.42E-08 | 4.04E-07 | 8.26712 |
| MNS1 | 0.57816 | 2.685882 | 5.78234 | 3.43E-08 | 4.05E-07 | 8.26466 |
| HIC1 | 0.57337 | 1.820358 | 5.78033 | 3.46E-08 | 4.08E-07 | 8.255 |
| ACPL2 | 0.63536 | 4.821747 | 5.77861 | 3.49E-08 | 4.11E-07 | 8.24669 |
| COL12A1 | 0.67021 | 1.741445 | 5.77196 | 3.61E-08 | 4.24E-07 | 8.21475 |
| PDZD4 | -0.8238 | 6.870894 | -5.7708 | 3.63E-08 | 4.26E-07 | 8.20924 |
| RASL10A | -1.2667 | 4.887235 | -5.7687 | 3.67E-08 | 4.30E-07 | 8.19924 |
| BMF | 0.66777 | 2.157509 | 5.76861 | 3.67E-08 | 4.30E-07 | 8.19865 |
| ZFR2 | -1.0268 | 2.277839 | -5.7678 | 3.68E-08 | 4.32E-07 | 8.19476 |
| LOX | 1.13608 | 1.434911 | 5.76751 | 3.69E-08 | 4.32E-07 | 8.19337 |
| ZSWIM4 | 0.54462 | 2.849051 | 5.7625 | 3.78E-08 | 4.42E-07 | 8.16933 |
| DUSP16 | 0.52 | 3.822237 | 5.7576 | 3.87E-08 | 4.52E-07 | 8.14579 |
| CSDC2 | -1.1915 | 4.37838 | -5.7529 | 3.97E-08 | 4.61E-07 | 8.12333 |
| CPNE5 | -0.9931 | 4.320395 | -5.7523 | 3.98E-08 | 4.63E-07 | 8.12037 |
| AVPI1 | -0.5018 | 3.161954 | -5.7518 | 3.99E-08 | 4.63E-07 | 8.11811 |
| ARHGDIG | -1.1694 | 4.591288 | -5.7516 | 3.99E-08 | 4.64E-07 | 8.11683 |
| S100A11 | 1.07747 | 6.494501 | 5.75075 | 4.01E-08 | 4.65E-07 | 8.11295 |
| PSMB9 | 0.79295 | 5.125261 | 5.75005 | 4.02E-08 | 4.67E-07 | 8.10958 |
| CYTIP | 0.64484 | 1.818335 | 5.7468 | 4.09E-08 | 4.74E-07 | 8.09402 |
| C1orf226 | 0.79619 | 3.278978 | 5.74588 | 4.11E-08 | 4.76E-07 | 8.08959 |
| SECTM1 | 0.78495 | 1.964753 | 5.74406 | 4.14E-08 | 4.79E-07 | 8.08089 |
| PALLD | 0.60336 | 5.319375 | 5.7433 | 4.16E-08 | 4.80E-07 | 8.07727 |
| SNHG12 | 0.81787 | 4.766165 | 5.74192 | 4.19E-08 | 4.83E-07 | 8.07062 |
| NAMPT | 1.10508 | 5.616427 | 5.74089 | 4.21E-08 | 4.86E-07 | 8.0657 |
| LIMS1 | 0.57595 | 4.805387 | 5.74037 | 4.22E-08 | 4.87E-07 | 8.06321 |
| NFKBIZ | 0.77266 | 2.315847 | 5.73843 | 4.26E-08 | 4.91E-07 | 8.05391 |
| IQGAP1 | 0.74143 | 4.933636 | 5.73653 | 4.30E-08 | 4.96E-07 | 8.04486 |
| SNX20 | 0.6587 | 1.350029 | 5.73568 | 4.32E-08 | 4.97E-07 | 8.04078 |
| GPR160 | 0.56198 | 1.933864 | 5.73512 | 4.33E-08 | 4.98E-07 | 8.03811 |
| MCF2L2 | -0.7742 | 3.316525 | -5.7346 | 4.34E-08 | 4.98E-07 | 8.03578 |
| CTSC | 0.94592 | 4.849493 | 5.73458 | 4.34E-08 | 4.98E-07 | 8.03552 |
| SH2B3 | 0.55088 | 3.318688 | 5.73454 | 4.34E-08 | 4.98E-07 | 8.03533 |
| CARD8 | 0.5247 | 4.469876 | 5.73034 | 4.43E-08 | 5.07E-07 | 8.01522 |
| ZC3H12A | 0.65142 | 1.774415 | 5.7302 | 4.44E-08 | 5.07E-07 | 8.01457 |
| GAPT | 0.79635 | 1.582859 | 5.72821 | 4.48E-08 | 5.12E-07 | 8.00504 |
| ABCC8 | -1.272 | 4.420348 | -5.7276 | 4.49E-08 | 5.12E-07 | 8.0023 |
| NAGA | 0.5301 | 3.955574 | 5.72738 | 4.50E-08 | 5.13E-07 | 8.00106 |
| FAM76B | 0.50914 | 4.079379 | 5.72707 | 4.51E-08 | 5.13E-07 | 7.99958 |
| NAT2 | 0.57241 | 0.731148 | 5.72701 | 4.51E-08 | 5.13E-07 | 7.9993 |
| AC034193.5 | 0.58584 | 1.533508 | 5.7248 | 4.56E-08 | 5.17E-07 | 7.98877 |
| PLEK2 | 0.52794 | 0.770581 | 5.72355 | 4.59E-08 | 5.20E-07 | 7.98275 |
| IGFBP7 | 0.82132 | 8.263386 | 5.71833 | 4.71E-08 | 5.32E-07 | 7.95782 |
| DOC2A | -1.2085 | 3.286954 | -5.7175 | 4.72E-08 | 5.34E-07 | 7.95406 |
| CD2 | 0.74693 | 1.163329 | 5.717 | 4.74E-08 | 5.35E-07 | 7.95149 |
| CRLF1 | -1.4885 | 4.433426 | -5.7153 | 4.78E-08 | 5.39E-07 | 7.94332 |
| MOV10 | 0.60564 | 4.498513 | 5.71512 | 4.78E-08 | 5.39E-07 | 7.94251 |
| HLA-DRA | 1.33175 | 7.349821 | 5.71033 | 4.90E-08 | 5.50E-07 | 7.91966 |
| COL6A1 | 0.75107 | 6.157932 | 5.70944 | 4.92E-08 | 5.52E-07 | 7.91543 |
| C15orf59 | -0.8644 | 6.047147 | -5.7085 | 4.94E-08 | 5.55E-07 | 7.91073 |
| 4-Sep | -1.0544 | 7.214252 | -5.7075 | 4.97E-08 | 5.57E-07 | 7.90595 |
| KIAA1045 | -1.3294 | 2.602281 | -5.7048 | 5.03E-08 | 5.63E-07 | 7.89322 |
| SERPINA1 | 1.13522 | 3.87548 | 5.70294 | 5.08E-08 | 5.68E-07 | 7.88441 |
| CTD-2396E7.10 | -1.0675 | 3.685089 | -5.7028 | 5.08E-08 | 5.69E-07 | 7.88367 |
| CHGA | -1.5759 | 4.531999 | -5.699 | 5.18E-08 | 5.79E-07 | 7.86585 |
| ORAI2 | 0.51227 | 4.766604 | 5.69425 | 5.30E-08 | 5.91E-07 | 7.84301 |
| GLIPR1 | 0.8707 | 3.668384 | 5.68775 | 5.47E-08 | 6.07E-07 | 7.81209 |
| GBP2 | 1.38202 | 4.1352 | 5.68722 | 5.49E-08 | 6.09E-07 | 7.80954 |
| HLA-DPA1 | 1.32394 | 6.894804 | 5.6859 | 5.52E-08 | 6.12E-07 | 7.80326 |
| CLEC2L | -1.1722 | 2.053615 | -5.6858 | 5.52E-08 | 6.12E-07 | 7.80274 |
| PEX5L | -1.0951 | 3.332556 | -5.6844 | 5.56E-08 | 6.16E-07 | 7.7962 |
| CTD-2396E7.9 | -1.1243 | 3.430318 | -5.6838 | 5.58E-08 | 6.17E-07 | 7.79319 |
| C1QTNF4 | -1.0682 | 3.503335 | -5.682 | 5.63E-08 | 6.22E-07 | 7.78475 |
| HOXD3 | 0.95028 | 1.249643 | 5.68189 | 5.63E-08 | 6.22E-07 | 7.78419 |
| SEMA3F | 0.76815 | 1.746265 | 5.68041 | 5.67E-08 | 6.26E-07 | 7.77716 |
| FPGT | 0.54429 | 2.8119 | 5.68012 | 5.68E-08 | 6.26E-07 | 7.77578 |
| HELZ2 | 0.64739 | 3.067938 | 5.68009 | 5.68E-08 | 6.26E-07 | 7.77563 |
| TSPYL2 | -0.6659 | 6.170222 | -5.6799 | 5.69E-08 | 6.26E-07 | 7.77477 |
| CPEB3 | -0.5552 | 2.440205 | -5.6796 | 5.70E-08 | 6.27E-07 | 7.77337 |
| MAP3K1 | 0.5483 | 2.673632 | 5.67701 | 5.77E-08 | 6.33E-07 | 7.76102 |
| ACTG2 | 1.19806 | 1.855334 | 5.67621 | 5.79E-08 | 6.35E-07 | 7.75721 |
| GAS1 | 0.91299 | 3.343724 | 5.67617 | 5.79E-08 | 6.35E-07 | 7.75704 |
| RNASE2 | 1.01743 | 1.853046 | 5.67481 | 5.83E-08 | 6.39E-07 | 7.75055 |
| SSFA2 | 0.51048 | 6.006336 | 5.6738 | 5.86E-08 | 6.41E-07 | 7.74575 |
| LEPREL2 | 0.5357 | 3.485547 | 5.66959 | 5.98E-08 | 6.54E-07 | 7.72576 |
| HLA-DPB1 | 1.27597 | 5.485305 | 5.66846 | 6.02E-08 | 6.56E-07 | 7.72043 |
| CSTA | 0.89099 | 1.473407 | 5.66623 | 6.08E-08 | 6.63E-07 | 7.7098 |
| RHOJ | 0.68151 | 3.179688 | 5.66558 | 6.10E-08 | 6.65E-07 | 7.70675 |
| TCIRG1 | 0.71683 | 4.028911 | 5.6648 | 6.13E-08 | 6.67E-07 | 7.70302 |
| MYO1B | 0.76293 | 3.340329 | 5.6631 | 6.18E-08 | 6.72E-07 | 7.69495 |
| CTC-260E6.6 | 0.50012 | 1.167689 | 5.66277 | 6.19E-08 | 6.73E-07 | 7.69341 |
| RP11-253E3.3 | 0.54068 | 0.674208 | 5.66115 | 6.24E-08 | 6.77E-07 | 7.68571 |
| SHISA6 | -1.3391 | 2.63262 | -5.6602 | 6.26E-08 | 6.80E-07 | 7.68143 |
| SLC8A2 | -1.1139 | 3.042215 | -5.6572 | 6.36E-08 | 6.88E-07 | 7.66685 |
| C1R | 1.24218 | 6.157131 | 5.65665 | 6.37E-08 | 6.89E-07 | 7.66438 |
| GAL3ST4 | 0.68546 | 4.228741 | 5.65599 | 6.40E-08 | 6.91E-07 | 7.66127 |
| XBP1 | 0.5675 | 5.09969 | 5.65587 | 6.40E-08 | 6.91E-07 | 7.66071 |
| CELSR1 | 0.72846 | 1.107515 | 5.65463 | 6.44E-08 | 6.95E-07 | 7.6548 |
| SERP2 | -0.6477 | 5.119348 | -5.6544 | 6.44E-08 | 6.95E-07 | 7.65382 |
| LCTL | 0.59768 | 1.156202 | 5.6514 | 6.54E-08 | 7.05E-07 | 7.63953 |
| LINC00152 | 1.15661 | 3.010157 | 5.65133 | 6.54E-08 | 7.05E-07 | 7.6392 |
| HIST1H3B | 0.60297 | 0.585855 | 5.64778 | 6.66E-08 | 7.16E-07 | 7.62238 |
| MMP9 | 1.42251 | 1.330538 | 5.644 | 6.78E-08 | 7.28E-07 | 7.60446 |
| ATP1A2 | -1.1057 | 7.658459 | -5.6434 | 6.80E-08 | 7.30E-07 | 7.6015 |
| HMGCLL1 | -0.8729 | 2.224476 | -5.6429 | 6.82E-08 | 7.31E-07 | 7.59949 |
| RP1-239B22.5 | -0.604 | 1.517476 | -5.6417 | 6.86E-08 | 7.35E-07 | 7.59352 |
| LTB4R | 0.50764 | 2.047345 | 5.6408 | 6.89E-08 | 7.37E-07 | 7.58933 |
| HK3 | 0.7328 | 1.707333 | 5.63958 | 6.93E-08 | 7.41E-07 | 7.58358 |
| FUT9 | -0.7847 | 3.288401 | -5.639 | 6.95E-08 | 7.43E-07 | 7.58085 |
| KANSL1L | 0.6354 | 3.152534 | 5.63661 | 7.03E-08 | 7.51E-07 | 7.56951 |
| PLAUR | 1.01561 | 2.897381 | 5.63401 | 7.12E-08 | 7.60E-07 | 7.5572 |
| TMEM173 | 0.65417 | 3.667062 | 5.63293 | 7.16E-08 | 7.63E-07 | 7.55209 |
| HOXD10 | 1.02062 | 0.777407 | 5.63243 | 7.18E-08 | 7.64E-07 | 7.54973 |
| RP11-118K6.3 | -0.5915 | 1.826966 | -5.6322 | 7.19E-08 | 7.64E-07 | 7.54846 |
| SULT4A1 | -1.6113 | 3.362996 | -5.6297 | 7.27E-08 | 7.73E-07 | 7.53688 |
| RP13-514E23.1 | -0.7198 | 2.830565 | -5.6289 | 7.30E-08 | 7.76E-07 | 7.53299 |
| LINC00087 | -0.8951 | 2.521558 | -5.6278 | 7.34E-08 | 7.79E-07 | 7.52798 |
| HLA-DOA | 1.04997 | 2.9718 | 5.62755 | 7.35E-08 | 7.79E-07 | 7.52671 |
| PTPN13 | 0.56044 | 4.050527 | 5.62707 | 7.37E-08 | 7.81E-07 | 7.52443 |
| TNFAIP3 | 0.68873 | 2.856025 | 5.6199 | 7.63E-08 | 8.06E-07 | 7.49057 |
| IL4I1 | 0.74447 | 1.401977 | 5.61844 | 7.69E-08 | 8.11E-07 | 7.48369 |
| MAL | -1.6548 | 4.652252 | -5.6182 | 7.69E-08 | 8.12E-07 | 7.4827 |
| RAB11FIP4 | -0.8654 | 4.303108 | -5.6171 | 7.74E-08 | 8.15E-07 | 7.47729 |
| HAPLN3 | 0.66035 | 2.176936 | 5.61283 | 7.90E-08 | 8.30E-07 | 7.45721 |
| GNAO1 | -0.6358 | 6.555464 | -5.6121 | 7.93E-08 | 8.33E-07 | 7.45366 |
| DNMBP-AS1 | -0.6725 | 1.020653 | -5.6115 | 7.95E-08 | 8.35E-07 | 7.45105 |
| LINC00086 | -0.7146 | 2.830823 | -5.611 | 7.97E-08 | 8.36E-07 | 7.44867 |
| ITGB7 | 0.56039 | 1.53497 | 5.61094 | 7.97E-08 | 8.36E-07 | 7.44831 |
| CHAF1B | 0.55073 | 1.311841 | 5.61076 | 7.98E-08 | 8.37E-07 | 7.44749 |
| SOWAHA | -1.0443 | 2.51242 | -5.6092 | 8.04E-08 | 8.42E-07 | 7.44024 |
| PIK3CG | 0.52694 | 1.393448 | 5.60812 | 8.08E-08 | 8.45E-07 | 7.43503 |
| LMO3 | -1.0143 | 5.430724 | -5.6048 | 8.21E-08 | 8.58E-07 | 7.41954 |
| TMEM151A | -1.117 | 3.986855 | -5.603 | 8.29E-08 | 8.64E-07 | 7.411 |
| PABPC1L2B | -0.7049 | 0.968016 | -5.6029 | 8.29E-08 | 8.64E-07 | 7.41031 |
| HS3ST1 | 0.82368 | 2.100897 | 5.60282 | 8.29E-08 | 8.64E-07 | 7.41007 |
| CLDN7 | 0.53838 | 1.348432 | 5.60189 | 8.33E-08 | 8.68E-07 | 7.4057 |
| BTN3A2 | 0.77746 | 4.682553 | 5.60178 | 8.34E-08 | 8.68E-07 | 7.40516 |
| SH3GL3 | -1.0977 | 3.970187 | -5.5973 | 8.52E-08 | 8.84E-07 | 7.38427 |
| STAT1 | 0.6706 | 5.666209 | 5.59657 | 8.55E-08 | 8.87E-07 | 7.38065 |
| RBM12 | 0.51481 | 4.734197 | 5.59581 | 8.58E-08 | 8.89E-07 | 7.37705 |
| SCP2 | 0.50005 | 6.779744 | 5.59413 | 8.65E-08 | 8.95E-07 | 7.36916 |
| SNAP91 | -1.1101 | 4.812966 | -5.5926 | 8.72E-08 | 9.01E-07 | 7.36216 |
| RP11-93B14.5 | 0.70042 | 0.811701 | 5.59151 | 8.76E-08 | 9.05E-07 | 7.35684 |
| TMSB15A | 1.24757 | 3.045928 | 5.59142 | 8.77E-08 | 9.05E-07 | 7.3564 |
| TWIST1 | 0.73686 | 1.449041 | 5.59065 | 8.80E-08 | 9.08E-07 | 7.35277 |
| CHST1 | -0.8488 | 4.811537 | -5.5904 | 8.81E-08 | 9.09E-07 | 7.35183 |
| PPP1R3B | 0.56774 | 1.951587 | 5.58979 | 8.84E-08 | 9.11E-07 | 7.34876 |
| EN2 | 0.58039 | 0.816381 | 5.58859 | 8.89E-08 | 9.15E-07 | 7.34313 |
| COL14A1 | 0.84096 | 1.676492 | 5.58771 | 8.93E-08 | 9.18E-07 | 7.33898 |
| SIGLEC7 | 0.6737 | 1.748785 | 5.5871 | 8.95E-08 | 9.20E-07 | 7.33611 |
| LCN12 | -0.7607 | 2.449777 | -5.5853 | 9.03E-08 | 9.26E-07 | 7.32766 |
| UNC13C | -1.0296 | 1.697964 | -5.5844 | 9.07E-08 | 9.30E-07 | 7.32344 |
| MRC2 | 1.03338 | 4.399468 | 5.58406 | 9.09E-08 | 9.31E-07 | 7.32184 |
| GLI3 | 0.56141 | 2.087344 | 5.58295 | 9.14E-08 | 9.34E-07 | 7.3166 |
| SH3GL2 | -1.1981 | 5.284383 | -5.5829 | 9.14E-08 | 9.34E-07 | 7.31645 |
| ASPA | -0.988 | 3.028802 | -5.5815 | 9.20E-08 | 9.40E-07 | 7.30993 |
| HIST1H3J | 0.5328 | 0.441163 | 5.58069 | 9.24E-08 | 9.44E-07 | 7.30598 |
| HMOX1 | 1.15194 | 4.836268 | 5.58026 | 9.26E-08 | 9.45E-07 | 7.30397 |
| TFDP2 | 0.52708 | 4.278873 | 5.57959 | 9.29E-08 | 9.48E-07 | 7.30084 |
| LINC00263 | -0.6002 | 2.796657 | -5.5763 | 9.44E-08 | 9.62E-07 | 7.28526 |
| MPZL2 | 0.70825 | 1.176528 | 5.57261 | 9.61E-08 | 9.76E-07 | 7.26806 |
| ECSCR | 1.01924 | 3.942808 | 5.56692 | 9.88E-08 | 1.00E-06 | 7.24139 |
| SNTA1 | -0.7957 | 4.990143 | -5.5652 | 9.96E-08 | 1.01E-06 | 7.23341 |
| SLC16A3 | 0.86918 | 3.680673 | 5.56484 | 9.98E-08 | 1.01E-06 | 7.23163 |
| FAM107A | -0.9863 | 8.359784 | -5.5628 | 1.01E-07 | 1.02E-06 | 7.22196 |
| BTBD19 | 0.60799 | 2.078818 | 5.56222 | 1.01E-07 | 1.02E-06 | 7.21933 |
| ENAH | 0.65265 | 6.160805 | 5.56199 | 1.01E-07 | 1.02E-06 | 7.21828 |
| MYH9 | 0.51393 | 5.636417 | 5.55832 | 1.03E-07 | 1.04E-06 | 7.20106 |
| SNRPN | -0.5924 | 5.508824 | -5.5576 | 1.03E-07 | 1.04E-06 | 7.19768 |
| CABP4 | 0.52649 | 1.028298 | 5.55671 | 1.04E-07 | 1.05E-06 | 7.19353 |
| TMEM59L | -0.668 | 7.059496 | -5.5564 | 1.04E-07 | 1.05E-06 | 7.19199 |
| ADAP1 | -0.9741 | 4.649067 | -5.5564 | 1.04E-07 | 1.05E-06 | 7.19196 |
| RP11-513I15.6 | -0.8719 | 3.686921 | -5.5459 | 1.09E-07 | 1.10E-06 | 7.14311 |
| DNAJB1 | 0.61838 | 6.218741 | 5.54317 | 1.11E-07 | 1.11E-06 | 7.13019 |
| FAM129B | 0.60374 | 4.924218 | 5.54035 | 1.12E-07 | 1.12E-06 | 7.117 |
| HOXA2 | 0.72419 | 0.566146 | 5.53888 | 1.13E-07 | 1.13E-06 | 7.11012 |
| HILPDA | 0.80994 | 4.018579 | 5.5373 | 1.14E-07 | 1.14E-06 | 7.10273 |
| ZNF738 | 0.54712 | 3.68208 | 5.53685 | 1.14E-07 | 1.14E-06 | 7.10063 |
| EPHB6 | -1.1865 | 3.278163 | -5.5361 | 1.15E-07 | 1.14E-06 | 7.09697 |
| SVOP | -1.5064 | 3.450028 | -5.5344 | 1.16E-07 | 1.15E-06 | 7.08911 |
| MSR1 | 1.1496 | 3.591883 | 5.53167 | 1.17E-07 | 1.16E-06 | 7.07645 |
| AP003039.3 | -0.9309 | 1.721598 | -5.5303 | 1.18E-07 | 1.17E-06 | 7.07001 |
| SOX11 | 0.94194 | 2.871817 | 5.52947 | 1.18E-07 | 1.18E-06 | 7.0662 |
| CD80 | 0.50863 | 0.582145 | 5.52606 | 1.20E-07 | 1.19E-06 | 7.05029 |
| PELI3 | -0.5097 | 3.63011 | -5.5246 | 1.21E-07 | 1.20E-06 | 7.04367 |
| ELTD1 | 0.71364 | 2.977629 | 5.52435 | 1.21E-07 | 1.20E-06 | 7.04231 |
| ACBD7 | -1.2159 | 3.610405 | -5.5228 | 1.22E-07 | 1.21E-06 | 7.03526 |
| PKIB | 1.03789 | 2.70817 | 5.52217 | 1.23E-07 | 1.21E-06 | 7.03213 |
| CA3 | 1.19497 | 1.47207 | 5.52186 | 1.23E-07 | 1.21E-06 | 7.03071 |
| GRM3 | -1.0719 | 3.936438 | -5.5195 | 1.24E-07 | 1.23E-06 | 7.01956 |
| CKMT1A | -1.2159 | 2.987888 | -5.5177 | 1.25E-07 | 1.24E-06 | 7.01134 |
| MYO1F | 0.71554 | 3.773027 | 5.51612 | 1.26E-07 | 1.24E-06 | 7.00397 |
| TM4SF18 | 0.61625 | 2.662486 | 5.51522 | 1.27E-07 | 1.25E-06 | 6.99976 |
| BGN | 1.01674 | 5.34997 | 5.51401 | 1.28E-07 | 1.25E-06 | 6.99414 |
| IL13RA2 | 1.27503 | 1.796205 | 5.51141 | 1.29E-07 | 1.27E-06 | 6.982 |
| NFE2L3 | 0.55907 | 2.091156 | 5.51124 | 1.29E-07 | 1.27E-06 | 6.98121 |
| EMR2 | 0.6087 | 1.446042 | 5.51002 | 1.30E-07 | 1.27E-06 | 6.97554 |
| TRPC6 | 0.58384 | 1.021384 | 5.50921 | 1.31E-07 | 1.28E-06 | 6.97178 |
| LEFTY2 | 1.08389 | 1.270486 | 5.50873 | 1.31E-07 | 1.28E-06 | 6.96956 |
| FOSL1 | 0.76629 | 1.446056 | 5.50682 | 1.32E-07 | 1.29E-06 | 6.96067 |
| TAGLN2 | 1.03118 | 6.120961 | 5.50574 | 1.33E-07 | 1.30E-06 | 6.95561 |
| DLGAP1-AS4 | -0.8559 | 1.022159 | -5.5043 | 1.34E-07 | 1.30E-06 | 6.9487 |
| KDELR3 | 0.65042 | 1.316439 | 5.50347 | 1.34E-07 | 1.31E-06 | 6.94504 |
| SEMA4A | -0.6599 | 3.191717 | -5.503 | 1.35E-07 | 1.31E-06 | 6.94274 |
| CNTNAP4 | -1.1999 | 3.557511 | -5.5017 | 1.35E-07 | 1.32E-06 | 6.93662 |
| SYN2 | -1.5883 | 3.699184 | -5.5016 | 1.35E-07 | 1.32E-06 | 6.93653 |
| CTB-175P5.4 | 0.52071 | 0.595219 | 5.50162 | 1.35E-07 | 1.32E-06 | 6.93647 |
| GPRC5B | -0.5726 | 8.264581 | -5.4961 | 1.39E-07 | 1.35E-06 | 6.91102 |
| RP11-791G15.2 | 0.54157 | 0.831657 | 5.49602 | 1.39E-07 | 1.35E-06 | 6.91042 |
| ERAP1 | 0.5335 | 4.350321 | 5.49438 | 1.40E-07 | 1.36E-06 | 6.90281 |
| DOCK3 | -0.6188 | 3.329608 | -5.4938 | 1.41E-07 | 1.36E-06 | 6.9003 |
| TMEM130 | -1.5239 | 3.923067 | -5.4934 | 1.41E-07 | 1.36E-06 | 6.89838 |
| SYNGR1 | -0.6966 | 5.303262 | -5.4934 | 1.41E-07 | 1.36E-06 | 6.89807 |
| BATF | 0.63315 | 1.072966 | 5.49254 | 1.42E-07 | 1.37E-06 | 6.89424 |
| HSD11B2 | 0.62088 | 1.307259 | 5.49139 | 1.42E-07 | 1.37E-06 | 6.88892 |
| PDGFD | 0.81452 | 1.458165 | 5.49075 | 1.43E-07 | 1.38E-06 | 6.88595 |
| KCNB1 | -0.7645 | 3.331087 | -5.4889 | 1.44E-07 | 1.39E-06 | 6.87734 |
| ARHGDIB | 0.73071 | 6.295091 | 5.48736 | 1.45E-07 | 1.40E-06 | 6.87022 |
| ROR1 | 0.60034 | 1.077661 | 5.48665 | 1.46E-07 | 1.40E-06 | 6.86691 |
| PPP1R16B | -1.0102 | 3.674138 | -5.4863 | 1.46E-07 | 1.40E-06 | 6.86535 |
| SCN3B | -1.1894 | 4.301502 | -5.4852 | 1.47E-07 | 1.41E-06 | 6.86036 |
| PLP2 | 1.01142 | 3.841222 | 5.48502 | 1.47E-07 | 1.41E-06 | 6.85936 |
| CACNG2 | -1.1206 | 2.306814 | -5.4845 | 1.47E-07 | 1.41E-06 | 6.85694 |
| ECM2 | 0.94759 | 3.210491 | 5.48413 | 1.47E-07 | 1.42E-06 | 6.85522 |
| IQSEC3 | -1.1482 | 2.862402 | -5.4839 | 1.47E-07 | 1.42E-06 | 6.85424 |
| C1orf54 | 0.68348 | 4.532479 | 5.48385 | 1.48E-07 | 1.42E-06 | 6.85391 |
| NEK6 | 0.6756 | 5.005855 | 5.48343 | 1.48E-07 | 1.42E-06 | 6.85198 |
| MYT1L | -1.2942 | 3.047237 | -5.4811 | 1.49E-07 | 1.43E-06 | 6.84125 |
| DMRTC1B | -0.6606 | 2.947569 | -5.4804 | 1.50E-07 | 1.44E-06 | 6.83776 |
| MIDN | 0.56824 | 5.870348 | 5.48013 | 1.50E-07 | 1.44E-06 | 6.83667 |
| PTGFRN | 0.77149 | 3.856632 | 5.47965 | 1.51E-07 | 1.44E-06 | 6.83442 |
| ABCC3 | 1.21082 | 1.927852 | 5.47948 | 1.51E-07 | 1.44E-06 | 6.83365 |
| DUSP10 | 0.61616 | 2.591331 | 5.47905 | 1.51E-07 | 1.44E-06 | 6.83167 |
| OGDHL | -1.0161 | 2.672018 | -5.4748 | 1.54E-07 | 1.47E-06 | 6.81208 |
| OSR1 | 0.83104 | 1.120672 | 5.4726 | 1.56E-07 | 1.48E-06 | 6.80177 |
| HSPA6 | 0.92308 | 1.661549 | 5.47037 | 1.57E-07 | 1.50E-06 | 6.79145 |
| RASGRF2 | -0.814 | 2.55376 | -5.4699 | 1.58E-07 | 1.50E-06 | 6.78911 |
| OLFM1 | -1.123 | 6.868118 | -5.4692 | 1.58E-07 | 1.50E-06 | 6.78597 |
| CPA4 | 0.59393 | 0.782357 | 5.4669 | 1.60E-07 | 1.52E-06 | 6.77535 |
| FRRS1L | -0.9416 | 3.100655 | -5.465 | 1.62E-07 | 1.53E-06 | 6.76643 |
| FAAH | -0.6004 | 4.080576 | -5.465 | 1.62E-07 | 1.53E-06 | 6.76638 |
| SEC14L5 | -1.0787 | 2.210196 | -5.4644 | 1.62E-07 | 1.53E-06 | 6.7637 |
| FCGR3A | 1.22567 | 5.45506 | 5.46399 | 1.62E-07 | 1.53E-06 | 6.76188 |
| CD300A | 0.70898 | 3.207822 | 5.46255 | 1.63E-07 | 1.54E-06 | 6.7552 |
| B2M | 0.73014 | 11.70391 | 5.46246 | 1.64E-07 | 1.54E-06 | 6.75479 |
| CD36 | 0.83421 | 1.744024 | 5.461 | 1.65E-07 | 1.55E-06 | 6.74804 |
| SPSB1 | 0.5975 | 4.002579 | 5.46071 | 1.65E-07 | 1.55E-06 | 6.74672 |
| CD7 | 0.53314 | 1.082275 | 5.46018 | 1.65E-07 | 1.56E-06 | 6.74426 |
| IRX5 | 0.67674 | 0.81708 | 5.45928 | 1.66E-07 | 1.56E-06 | 6.7401 |
| YBX1P1 | 0.51051 | 1.319337 | 5.45908 | 1.66E-07 | 1.56E-06 | 6.73917 |
| CRISPLD1 | 0.95016 | 4.755015 | 5.45885 | 1.66E-07 | 1.56E-06 | 6.7381 |
| CDH22 | -0.9212 | 2.491492 | -5.4565 | 1.68E-07 | 1.58E-06 | 6.72737 |
| LAPTM4B | 0.53459 | 6.210505 | 5.45559 | 1.69E-07 | 1.59E-06 | 6.72303 |
| CACNA2D2 | -0.6081 | 1.994392 | -5.4551 | 1.69E-07 | 1.59E-06 | 6.72096 |
| RDX | 0.51682 | 6.672606 | 5.45474 | 1.70E-07 | 1.59E-06 | 6.71912 |
| TMEM235 | -1.2837 | 2.548392 | -5.4546 | 1.70E-07 | 1.59E-06 | 6.71832 |
| KCNJ11 | -1.0943 | 3.323206 | -5.4493 | 1.74E-07 | 1.63E-06 | 6.69413 |
| RGS19 | 0.55175 | 3.982168 | 5.44851 | 1.75E-07 | 1.63E-06 | 6.69032 |
| MAP3K6 | 0.69991 | 3.167152 | 5.44675 | 1.76E-07 | 1.64E-06 | 6.68218 |
| ANXA3 | -0.6546 | 1.674801 | -5.4461 | 1.77E-07 | 1.65E-06 | 6.679 |
| MPZ | 0.55633 | 2.075633 | 5.44462 | 1.78E-07 | 1.66E-06 | 6.67234 |
| ULBP2 | 0.50229 | 1.162477 | 5.44275 | 1.80E-07 | 1.67E-06 | 6.66375 |
| ADAMTS15 | 0.97927 | 1.776727 | 5.44227 | 1.80E-07 | 1.67E-06 | 6.66151 |
| LUM | 1.16334 | 2.692765 | 5.44073 | 1.81E-07 | 1.69E-06 | 6.65439 |
| RP1-152L7.5 | 0.52336 | 2.721962 | 5.43894 | 1.83E-07 | 1.70E-06 | 6.64613 |
| TWSG1 | 0.54746 | 4.181607 | 5.43842 | 1.83E-07 | 1.70E-06 | 6.64375 |
| IGF2BP2 | 0.88507 | 1.048186 | 5.43341 | 1.88E-07 | 1.74E-06 | 6.62063 |
| TLR1 | 0.74346 | 2.727867 | 5.43287 | 1.88E-07 | 1.74E-06 | 6.61816 |
| ID3 | 1.06002 | 7.347878 | 5.4314 | 1.90E-07 | 1.75E-06 | 6.61136 |
| CD8BP | -0.525 | 0.865202 | -5.4312 | 1.90E-07 | 1.75E-06 | 6.61031 |
| ITK | 0.63724 | 0.809762 | 5.43014 | 1.91E-07 | 1.76E-06 | 6.60557 |
| PHYHD1 | -0.8091 | 4.521462 | -5.4268 | 1.94E-07 | 1.78E-06 | 6.59029 |
| NNMT | 1.55953 | 2.972084 | 5.42521 | 1.95E-07 | 1.80E-06 | 6.58285 |
| ITGAL | 0.71645 | 2.346583 | 5.42216 | 1.98E-07 | 1.82E-06 | 6.56883 |
| PSMB8 | 0.66892 | 5.430706 | 5.42141 | 1.99E-07 | 1.83E-06 | 6.56536 |
| CCDC167 | 0.52391 | 5.64751 | 5.41915 | 2.01E-07 | 1.85E-06 | 6.55494 |
| PTGER4 | 0.6829 | 1.689014 | 5.4189 | 2.01E-07 | 1.85E-06 | 6.55383 |
| CD163 | 1.37375 | 3.85104 | 5.41879 | 2.01E-07 | 1.85E-06 | 6.55332 |
| CALY | -1.5163 | 3.789204 | -5.4091 | 2.11E-07 | 1.93E-06 | 6.50875 |
| IQSEC2 | -0.5574 | 3.851083 | -5.407 | 2.13E-07 | 1.94E-06 | 6.49911 |
| LIMD2 | 0.52563 | 4.298852 | 5.40627 | 2.14E-07 | 1.95E-06 | 6.49576 |
| GNAL | -0.8788 | 3.39352 | -5.4061 | 2.14E-07 | 1.95E-06 | 6.49496 |
| LGALS3BP | 0.63343 | 6.900739 | 5.40278 | 2.17E-07 | 1.98E-06 | 6.47972 |
| RP11-5P18.10 | -0.5602 | 0.926407 | -5.4022 | 2.18E-07 | 1.98E-06 | 6.47689 |
| HIST2H4A | 0.63939 | 2.979159 | 5.40178 | 2.19E-07 | 1.99E-06 | 6.47511 |
| ADPRH | 0.55976 | 2.043061 | 5.39662 | 2.24E-07 | 2.03E-06 | 6.45146 |
| OS9 | 0.61098 | 7.366322 | 5.3964 | 2.24E-07 | 2.03E-06 | 6.45042 |
| HDAC1 | 0.57203 | 5.034835 | 5.39595 | 2.25E-07 | 2.03E-06 | 6.44836 |
| EPHA2 | 0.65851 | 1.880031 | 5.39567 | 2.25E-07 | 2.04E-06 | 6.4471 |
| COL8A1 | 1.02265 | 1.267577 | 5.39408 | 2.27E-07 | 2.05E-06 | 6.43978 |
| NECAB1 | -1.0625 | 3.244425 | -5.3936 | 2.27E-07 | 2.05E-06 | 6.4374 |
| C1orf162 | 0.80595 | 3.74505 | 5.39309 | 2.28E-07 | 2.06E-06 | 6.43526 |
| TSPAN7 | -0.6246 | 8.41225 | -5.3928 | 2.28E-07 | 2.06E-06 | 6.43397 |
| OSMR | 0.87463 | 2.762114 | 5.39163 | 2.29E-07 | 2.07E-06 | 6.42857 |
| GABBR1 | -0.8443 | 7.423438 | -5.3901 | 2.31E-07 | 2.08E-06 | 6.42171 |
| KIAA1644 | -1.0871 | 2.560369 | -5.3899 | 2.31E-07 | 2.08E-06 | 6.42056 |
| RP11-355I22.7 | -0.941 | 2.506254 | -5.3886 | 2.33E-07 | 2.10E-06 | 6.41457 |
| ACRBP | 0.57053 | 1.751117 | 5.38727 | 2.34E-07 | 2.11E-06 | 6.40858 |
| AC112229.7 | -1.2142 | 1.899446 | -5.3841 | 2.38E-07 | 2.14E-06 | 6.39396 |
| AC093673.5 | 0.64356 | 3.163435 | 5.38381 | 2.38E-07 | 2.14E-06 | 6.39275 |
| RPA3 | 0.50805 | 4.448986 | 5.38198 | 2.40E-07 | 2.15E-06 | 6.38433 |
| NAPB | -1.0467 | 4.756962 | -5.3794 | 2.43E-07 | 2.18E-06 | 6.37253 |
| SLC25A24 | 0.56828 | 2.115289 | 5.37907 | 2.43E-07 | 2.18E-06 | 6.37101 |
| ARRDC3 | 0.54557 | 4.440656 | 5.37759 | 2.45E-07 | 2.19E-06 | 6.36424 |
| GABRG2 | -1.4309 | 3.167448 | -5.3775 | 2.45E-07 | 2.19E-06 | 6.36365 |
| ANXA2P2 | 0.51543 | 0.582225 | 5.37703 | 2.46E-07 | 2.20E-06 | 6.36168 |
| OR51E1 | 0.52271 | 0.448588 | 5.37473 | 2.48E-07 | 2.22E-06 | 6.35116 |
| SNAI2 | 0.75659 | 1.822219 | 5.37304 | 2.50E-07 | 2.24E-06 | 6.34341 |
| FHOD1 | 0.6039 | 3.504568 | 5.37175 | 2.52E-07 | 2.25E-06 | 6.33752 |
| MS4A6A | 1.36601 | 5.127183 | 5.37017 | 2.54E-07 | 2.26E-06 | 6.33031 |
| AATK | -0.9698 | 5.166714 | -5.3686 | 2.56E-07 | 2.28E-06 | 6.32293 |
| LGI3 | -1.1998 | 4.160886 | -5.3681 | 2.56E-07 | 2.28E-06 | 6.32083 |
| ITGB1P1 | 0.88637 | 2.148439 | 5.3673 | 2.57E-07 | 2.29E-06 | 6.31718 |
| NFKB2 | 0.5436 | 3.24078 | 5.36661 | 2.58E-07 | 2.29E-06 | 6.31402 |
| DOK2 | 0.6207 | 1.25047 | 5.36656 | 2.58E-07 | 2.29E-06 | 6.31383 |
| HLA-DOB | 0.55483 | 0.968255 | 5.36583 | 2.59E-07 | 2.30E-06 | 6.31047 |
| RGS16 | 1.02634 | 2.689216 | 5.36511 | 2.60E-07 | 2.31E-06 | 6.3072 |
| SYT5 | -1.2275 | 3.611046 | -5.3622 | 2.64E-07 | 2.34E-06 | 6.29379 |
| PVRL2 | 0.51667 | 4.58506 | 5.36064 | 2.66E-07 | 2.35E-06 | 6.28677 |
| LINC01018 | -0.5674 | 0.844226 | -5.3606 | 2.66E-07 | 2.35E-06 | 6.28672 |
| LY96 | 0.88149 | 3.564362 | 5.35773 | 2.69E-07 | 2.38E-06 | 6.27348 |
| CXCR6 | 0.54244 | 0.704097 | 5.35706 | 2.70E-07 | 2.39E-06 | 6.27041 |
| P4HA1 | 0.56476 | 4.630334 | 5.3568 | 2.71E-07 | 2.39E-06 | 6.26924 |
| STXBP5-AS1 | -0.5701 | 1.370077 | -5.3559 | 2.72E-07 | 2.40E-06 | 6.26502 |
| MCL1 | 0.52668 | 6.59161 | 5.35547 | 2.72E-07 | 2.40E-06 | 6.26316 |
| MMP25 | 0.53866 | 1.581947 | 5.35292 | 2.76E-07 | 2.42E-06 | 6.25153 |
| HIST2H2AA4 | 0.83054 | 4.322575 | 5.35129 | 2.78E-07 | 2.44E-06 | 6.24409 |
| LRRK2 | 0.7571 | 2.617609 | 5.35102 | 2.78E-07 | 2.44E-06 | 6.24287 |
| HSD17B6 | -0.8122 | 3.491572 | -5.348 | 2.82E-07 | 2.47E-06 | 6.22929 |
| ANKRD29 | -0.6895 | 2.016803 | -5.3476 | 2.83E-07 | 2.48E-06 | 6.22711 |
| MYBPC1 | -1.0618 | 3.689233 | -5.3467 | 2.84E-07 | 2.49E-06 | 6.22308 |
| MAP1A | -0.6032 | 5.963572 | -5.3461 | 2.85E-07 | 2.49E-06 | 6.22049 |
| RP11-421L21.3 | 0.51372 | 2.703353 | 5.34551 | 2.85E-07 | 2.50E-06 | 6.21773 |
| PRKCB | -0.9982 | 3.975006 | -5.3434 | 2.88E-07 | 2.52E-06 | 6.20794 |
| LBH | 0.79015 | 3.846491 | 5.3427 | 2.89E-07 | 2.52E-06 | 6.20493 |
| ST8SIA3 | -1.1017 | 3.011894 | -5.3423 | 2.90E-07 | 2.53E-06 | 6.20327 |
| APOL1 | 0.78667 | 2.796108 | 5.34094 | 2.92E-07 | 2.54E-06 | 6.19693 |
| CELF4 | -1.3006 | 3.848054 | -5.3398 | 2.93E-07 | 2.55E-06 | 6.19173 |
| KYNU | 0.61232 | 1.376867 | 5.3396 | 2.93E-07 | 2.55E-06 | 6.19081 |
| SOCS3 | 1.38283 | 2.981677 | 5.33633 | 2.98E-07 | 2.59E-06 | 6.17596 |
| AVIL | 0.73799 | 2.739116 | 5.33588 | 2.99E-07 | 2.59E-06 | 6.17387 |
| RBM47 | 0.66785 | 2.168815 | 5.33379 | 3.02E-07 | 2.62E-06 | 6.16437 |
| PWAR6 | -0.736 | 4.060323 | -5.332 | 3.04E-07 | 2.64E-06 | 6.15646 |
| THBS3 | 0.5079 | 4.200134 | 5.33105 | 3.06E-07 | 2.64E-06 | 6.1519 |
| FOSL2 | 0.81147 | 3.823256 | 5.3307 | 3.06E-07 | 2.64E-06 | 6.1503 |
| FAM20A | 0.8778 | 1.803 | 5.33068 | 3.06E-07 | 2.64E-06 | 6.15024 |
| TP53 | 0.71237 | 4.731501 | 5.32866 | 3.09E-07 | 2.66E-06 | 6.14106 |
| DYNC1I1 | -0.9511 | 3.787969 | -5.3265 | 3.12E-07 | 2.69E-06 | 6.13114 |
| CNRIP1 | -0.6282 | 5.995508 | -5.3214 | 3.20E-07 | 2.75E-06 | 6.10792 |
| CLEC2B | 0.70431 | 2.143598 | 5.31941 | 3.23E-07 | 2.77E-06 | 6.09903 |
| GPR82 | 0.51752 | 0.755402 | 5.31765 | 3.25E-07 | 2.79E-06 | 6.09101 |
| STK38L | 0.54157 | 4.555522 | 5.31754 | 3.26E-07 | 2.79E-06 | 6.09051 |
| HOXC6 | 0.65741 | 0.639486 | 5.31559 | 3.29E-07 | 2.82E-06 | 6.08167 |
| TLR10 | 0.59915 | 1.48792 | 5.313 | 3.33E-07 | 2.85E-06 | 6.06991 |
| NRG3 | -0.7893 | 3.558415 | -5.3113 | 3.35E-07 | 2.87E-06 | 6.06206 |
| SST | -1.4495 | 3.730963 | -5.3101 | 3.37E-07 | 2.88E-06 | 6.05662 |
| MPPED1 | -1.1885 | 2.298881 | -5.3064 | 3.43E-07 | 2.92E-06 | 6.04 |
| RP1-293L6.1 | -0.9041 | 1.576856 | -5.306 | 3.44E-07 | 2.92E-06 | 6.038 |
| AC006126.4 | 0.50072 | 0.354402 | 5.3057 | 3.44E-07 | 2.93E-06 | 6.03679 |
| GRIN3A | -0.9096 | 1.764228 | -5.3046 | 3.46E-07 | 2.94E-06 | 6.03167 |
| KIAA1671 | -0.5108 | 3.271249 | -5.3009 | 3.52E-07 | 2.99E-06 | 6.01509 |
| SIPA1L2 | 0.52383 | 4.450581 | 5.3004 | 3.53E-07 | 2.99E-06 | 6.01281 |
| SV2B | -1.2766 | 2.512257 | -5.2999 | 3.54E-07 | 2.99E-06 | 6.01065 |
| OR52K3P | 0.62355 | 0.908341 | 5.29833 | 3.56E-07 | 3.01E-06 | 6.00343 |
| SNX7 | 0.56549 | 4.224346 | 5.29822 | 3.57E-07 | 3.01E-06 | 6.0029 |
| INPPL1 | 0.53507 | 6.36464 | 5.29651 | 3.60E-07 | 3.03E-06 | 5.99519 |
| NHLH1 | 0.60569 | 0.84109 | 5.29477 | 3.62E-07 | 3.05E-06 | 5.98728 |
| RP11-834C11.4 | 0.82576 | 2.869874 | 5.29257 | 3.66E-07 | 3.08E-06 | 5.97734 |
| 3-Sep | -0.7176 | 6.761369 | -5.2922 | 3.67E-07 | 3.09E-06 | 5.9755 |
| RP11-361F15.2 | -0.7459 | 2.288154 | -5.2918 | 3.68E-07 | 3.09E-06 | 5.97368 |
| SOHLH1 | -0.8223 | 1.14455 | -5.2915 | 3.68E-07 | 3.10E-06 | 5.97252 |
| PDCD1LG2 | 0.60111 | 1.156295 | 5.28674 | 3.76E-07 | 3.16E-06 | 5.95097 |
| HPSE2 | -1.0166 | 2.015973 | -5.2863 | 3.77E-07 | 3.16E-06 | 5.94919 |
| RP11-580I16.2 | 0.57356 | 1.271855 | 5.28404 | 3.81E-07 | 3.20E-06 | 5.93875 |
| APOBEC3C | 0.75331 | 2.807077 | 5.28282 | 3.83E-07 | 3.21E-06 | 5.93324 |
| FEZF2 | -0.6992 | 1.78216 | -5.2808 | 3.87E-07 | 3.24E-06 | 5.92419 |
| CISH | 0.63316 | 1.305711 | 5.27961 | 3.89E-07 | 3.25E-06 | 5.91876 |
| FAM19A5 | -0.6648 | 5.716293 | -5.2796 | 3.89E-07 | 3.25E-06 | 5.9185 |
| THBD | 0.81569 | 1.508994 | 5.27769 | 3.93E-07 | 3.28E-06 | 5.91009 |
| NAPSB | 1.1948 | 3.280889 | 5.27437 | 3.99E-07 | 3.32E-06 | 5.89508 |
| HS3ST4 | -0.8731 | 1.998065 | -5.2727 | 4.02E-07 | 3.35E-06 | 5.88747 |
| ACTN1 | 1.04455 | 5.158549 | 5.27229 | 4.03E-07 | 3.35E-06 | 5.8857 |
| DDN | -1.5006 | 3.288028 | -5.2717 | 4.04E-07 | 3.36E-06 | 5.88308 |
| CPLX1 | -1.1561 | 4.756808 | -5.2692 | 4.09E-07 | 3.40E-06 | 5.87173 |
| RAPGEF3 | -0.6388 | 4.168776 | -5.2687 | 4.10E-07 | 3.40E-06 | 5.86948 |
| CACNA2D4 | 0.6445 | 1.898514 | 5.26669 | 4.14E-07 | 3.43E-06 | 5.86043 |
| TPD52L1 | -0.9979 | 4.602244 | -5.266 | 4.15E-07 | 3.44E-06 | 5.85743 |
| SPATA6 | 0.76703 | 3.207363 | 5.26553 | 4.16E-07 | 3.45E-06 | 5.85523 |
| FAM196B | -0.6836 | 1.918425 | -5.2651 | 4.17E-07 | 3.45E-06 | 5.85329 |
| PABPC1L2A | -0.6698 | 0.945026 | -5.2646 | 4.18E-07 | 3.46E-06 | 5.85102 |
| ARHGAP30 | 0.62636 | 2.940936 | 5.26338 | 4.20E-07 | 3.48E-06 | 5.84553 |
| IQCA1 | -0.8346 | 2.060511 | -5.261 | 4.25E-07 | 3.51E-06 | 5.835 |
| PROCR | 0.53115 | 2.598164 | 5.25973 | 4.27E-07 | 3.52E-06 | 5.82906 |
| CCNL2 | 0.58235 | 6.460286 | 5.2597 | 4.27E-07 | 3.52E-06 | 5.82893 |
| TMEM56 | -0.6636 | 2.117764 | -5.2557 | 4.35E-07 | 3.58E-06 | 5.81101 |
| TCEAL6 | -1.2178 | 3.042301 | -5.2552 | 4.36E-07 | 3.59E-06 | 5.80879 |
| CD300LF | 0.64991 | 1.657603 | 5.25306 | 4.41E-07 | 3.62E-06 | 5.79905 |
| CBLN2 | -1.0971 | 2.272038 | -5.2529 | 4.41E-07 | 3.62E-06 | 5.7985 |
| NEURL1B | 0.59133 | 3.068928 | 5.25278 | 4.41E-07 | 3.62E-06 | 5.79776 |
| UBE2QL1 | -0.9065 | 2.915069 | -5.2503 | 4.47E-07 | 3.66E-06 | 5.78653 |
| CHI3L2 | 1.90389 | 4.561093 | 5.25002 | 4.47E-07 | 3.67E-06 | 5.78535 |
| GALNT16 | -0.6063 | 4.18047 | -5.2494 | 4.48E-07 | 3.67E-06 | 5.78272 |
| VSNL1 | -1.8908 | 4.646095 | -5.2487 | 4.50E-07 | 3.68E-06 | 5.7795 |
| ZNF683 | 0.64193 | 0.704576 | 5.24765 | 4.52E-07 | 3.70E-06 | 5.7747 |
| ELF4 | 0.54463 | 1.48708 | 5.24715 | 4.53E-07 | 3.70E-06 | 5.77246 |
| FREM2 | 0.64334 | 1.21826 | 5.24703 | 4.53E-07 | 3.70E-06 | 5.77192 |
| IBSP | 1.18098 | 0.936232 | 5.24609 | 4.55E-07 | 3.72E-06 | 5.76767 |
| MTMR7 | -0.6886 | 2.929371 | -5.2459 | 4.56E-07 | 3.72E-06 | 5.76693 |
| CASKIN1 | -0.8502 | 4.194484 | -5.2447 | 4.58E-07 | 3.74E-06 | 5.76149 |
| RPLP0 | 0.59055 | 10.24359 | 5.24089 | 4.67E-07 | 3.80E-06 | 5.74434 |
| HOXD-AS2 | 0.78083 | 1.044689 | 5.2378 | 4.73E-07 | 3.84E-06 | 5.73045 |
| RDH10 | 0.9958 | 3.577179 | 5.23343 | 4.83E-07 | 3.92E-06 | 5.71082 |
| CAMK2B | -1.1344 | 4.504531 | -5.2323 | 4.86E-07 | 3.93E-06 | 5.70572 |
| CPLX2 | -1.5635 | 5.079459 | -5.2321 | 4.86E-07 | 3.94E-06 | 5.70463 |
| MUC1 | 0.71707 | 3.028684 | 5.23126 | 4.88E-07 | 3.95E-06 | 5.70107 |
| LINC00882 | -0.5331 | 3.135025 | -5.2298 | 4.91E-07 | 3.97E-06 | 5.69431 |
| LHFPL2 | 0.65892 | 4.119595 | 5.22808 | 4.95E-07 | 4.00E-06 | 5.6868 |
| TRIM21 | 0.56781 | 3.243742 | 5.22776 | 4.96E-07 | 4.00E-06 | 5.68537 |
| STAB1 | 0.89559 | 5.002352 | 5.22552 | 5.01E-07 | 4.04E-06 | 5.67532 |
| EMILIN2 | 0.81409 | 2.162951 | 5.22538 | 5.02E-07 | 4.04E-06 | 5.67472 |
| PRR18 | -1.0449 | 3.519294 | -5.2245 | 5.04E-07 | 4.06E-06 | 5.67059 |
| ATP8A1 | -0.7903 | 4.556942 | -5.2243 | 5.04E-07 | 4.06E-06 | 5.66973 |
| B3GAT1 | -0.6406 | 6.334326 | -5.2231 | 5.07E-07 | 4.08E-06 | 5.66439 |
| CDH5 | 0.54338 | 3.106345 | 5.22175 | 5.10E-07 | 4.10E-06 | 5.65844 |
| FPR2 | 0.62464 | 0.884297 | 5.22158 | 5.10E-07 | 4.10E-06 | 5.65767 |
| HIST1H4A | 0.58878 | 0.647142 | 5.2205 | 5.13E-07 | 4.12E-06 | 5.65284 |
| SPOCK3 | -1.2237 | 4.709346 | -5.2196 | 5.15E-07 | 4.13E-06 | 5.64869 |
| GPR4 | 0.58015 | 1.912572 | 5.21827 | 5.18E-07 | 4.15E-06 | 5.64282 |
| ETS1 | 0.70389 | 4.459001 | 5.21211 | 5.33E-07 | 4.26E-06 | 5.61527 |
| LINC00844 | -1.0568 | 6.708219 | -5.2118 | 5.34E-07 | 4.26E-06 | 5.61367 |
| HOXC9 | 0.64082 | 0.503542 | 5.21 | 5.39E-07 | 4.29E-06 | 5.60584 |
| GDF15 | 1.00419 | 1.648848 | 5.20953 | 5.40E-07 | 4.30E-06 | 5.60373 |
| TRIO | 0.59562 | 5.856449 | 5.20824 | 5.43E-07 | 4.31E-06 | 5.59794 |
| BAI3 | -0.6551 | 4.665126 | -5.2075 | 5.45E-07 | 4.33E-06 | 5.59441 |
| OPALIN | -1.6042 | 3.072768 | -5.2072 | 5.46E-07 | 4.33E-06 | 5.59342 |
| MMP7 | 0.93887 | 0.761974 | 5.2069 | 5.47E-07 | 4.33E-06 | 5.59195 |
| SYT1 | -1.664 | 4.405864 | -5.2068 | 5.47E-07 | 4.33E-06 | 5.59164 |
| SMS | 0.53243 | 5.404086 | 5.2052 | 5.51E-07 | 4.36E-06 | 5.58436 |
| C14orf132 | -0.6679 | 5.676417 | -5.205 | 5.51E-07 | 4.36E-06 | 5.5836 |
| CHGB | -1.3044 | 5.306336 | -5.2026 | 5.58E-07 | 4.41E-06 | 5.57281 |
| RP11-143K11.1 | -0.9623 | 2.847811 | -5.2025 | 5.58E-07 | 4.41E-06 | 5.57244 |
| LCNL1 | -0.904 | 3.043849 | -5.2015 | 5.60E-07 | 4.42E-06 | 5.56789 |
| BACE2 | 0.7285 | 3.028246 | 5.20129 | 5.61E-07 | 4.43E-06 | 5.56687 |
| SPATS2L | 0.50498 | 5.474013 | 5.19862 | 5.68E-07 | 4.48E-06 | 5.55495 |
| DUSP26 | -0.8365 | 4.764065 | -5.1979 | 5.70E-07 | 4.49E-06 | 5.55155 |
| CASP7 | 0.53033 | 2.834987 | 5.19646 | 5.74E-07 | 4.51E-06 | 5.54529 |
| LTBR | 0.65694 | 3.479922 | 5.19544 | 5.76E-07 | 4.53E-06 | 5.54075 |
| CARNS1 | -1.457 | 4.306103 | -5.1913 | 5.88E-07 | 4.61E-06 | 5.52242 |
| ARPP21 | -0.9106 | 4.56856 | -5.1911 | 5.88E-07 | 4.62E-06 | 5.52133 |
| FRMPD2P1 | -0.7649 | 1.42296 | -5.1894 | 5.93E-07 | 4.65E-06 | 5.5136 |
| SLC25A48 | -1.2421 | 4.524534 | -5.1872 | 5.99E-07 | 4.69E-06 | 5.50399 |
| ITGAV | 0.52119 | 5.817047 | 5.18679 | 6.00E-07 | 4.69E-06 | 5.50215 |
| MOBP | -1.8404 | 5.647565 | -5.1853 | 6.04E-07 | 4.72E-06 | 5.49559 |
| CNNM1 | -0.7528 | 1.599395 | -5.1849 | 6.05E-07 | 4.72E-06 | 5.49366 |
| RAVER1 | 0.55652 | 4.544191 | 5.18419 | 6.07E-07 | 4.74E-06 | 5.49056 |
| LDLRAD3 | 0.54988 | 4.887567 | 5.18387 | 6.08E-07 | 4.74E-06 | 5.48914 |
| NGEF | -1.1942 | 4.099454 | -5.1835 | 6.09E-07 | 4.75E-06 | 5.4875 |
| ATP2B3 | -0.905 | 1.868457 | -5.1834 | 6.09E-07 | 4.75E-06 | 5.48714 |
| ATP2B2 | -0.8693 | 4.060551 | -5.1816 | 6.15E-07 | 4.78E-06 | 5.47887 |
| AHR | 0.68009 | 2.956387 | 5.18131 | 6.15E-07 | 4.79E-06 | 5.47773 |
| ISG20 | 0.7823 | 2.374849 | 5.17889 | 6.22E-07 | 4.84E-06 | 5.46691 |
| IL2RA | 0.60686 | 0.665264 | 5.17737 | 6.27E-07 | 4.86E-06 | 5.46018 |
| MAP3K7CL | 0.87825 | 2.219088 | 5.17586 | 6.31E-07 | 4.89E-06 | 5.45346 |
| TEAD2 | 0.70327 | 2.551079 | 5.17399 | 6.37E-07 | 4.93E-06 | 5.44511 |
| VSTM2A | -1.3243 | 3.859622 | -5.1732 | 6.39E-07 | 4.94E-06 | 5.44144 |
| SPTBN4 | -0.6319 | 4.016487 | -5.1732 | 6.39E-07 | 4.94E-06 | 5.44142 |
| RP3-395M20.12 | -0.8214 | 1.62462 | -5.1726 | 6.41E-07 | 4.95E-06 | 5.43899 |
| S100A4 | 1.1624 | 3.598466 | 5.17259 | 6.41E-07 | 4.95E-06 | 5.43887 |
| NKAIN2 | -1.0981 | 3.334599 | -5.1725 | 6.41E-07 | 4.96E-06 | 5.43832 |
| RP11-482M8.3 | -0.5318 | 1.022184 | -5.172 | 6.43E-07 | 4.96E-06 | 5.43627 |
| ARL4C | 0.87693 | 4.135567 | 5.17093 | 6.46E-07 | 4.99E-06 | 5.43148 |
| RP11-22P6.3 | 0.50238 | 1.467415 | 5.16842 | 6.53E-07 | 5.04E-06 | 5.42031 |
| CARD9 | 0.546 | 2.319712 | 5.1668 | 6.58E-07 | 5.07E-06 | 5.41312 |
| PNP | 0.54054 | 4.774144 | 5.16408 | 6.67E-07 | 5.13E-06 | 5.40104 |
| CBLB | 0.51526 | 3.264586 | 5.16056 | 6.77E-07 | 5.21E-06 | 5.38538 |
| TNFRSF1A | 0.74865 | 5.451821 | 5.16007 | 6.79E-07 | 5.22E-06 | 5.38322 |
| KCNK12 | -0.6727 | 1.529929 | -5.156 | 6.92E-07 | 5.30E-06 | 5.36533 |
| TMSB4X | 0.63744 | 11.46548 | 5.15557 | 6.93E-07 | 5.31E-06 | 5.3632 |
| RAPGEF4 | -0.8937 | 5.185885 | -5.1548 | 6.96E-07 | 5.33E-06 | 5.35967 |
| GDA | -1.2619 | 2.461325 | -5.1529 | 7.02E-07 | 5.37E-06 | 5.35127 |
| GSTM5 | -0.9591 | 2.269996 | -5.1528 | 7.02E-07 | 5.37E-06 | 5.35071 |
| CD101 | 0.57222 | 1.290456 | 5.15242 | 7.03E-07 | 5.38E-06 | 5.34922 |
| SIX1 | 0.86429 | 1.852524 | 5.15226 | 7.04E-07 | 5.38E-06 | 5.34853 |
| ENTPD3 | -0.7286 | 1.250949 | -5.1521 | 7.04E-07 | 5.38E-06 | 5.34789 |
| WDR90 | 0.50836 | 4.142039 | 5.15076 | 7.09E-07 | 5.41E-06 | 5.34189 |
| DOC2B | -0.7041 | 1.636341 | -5.1497 | 7.12E-07 | 5.44E-06 | 5.33714 |
| AEN | 0.60905 | 3.416456 | 5.14477 | 7.29E-07 | 5.55E-06 | 5.31532 |
| ABLIM2 | -0.701 | 3.311607 | -5.1435 | 7.33E-07 | 5.58E-06 | 5.3095 |
| MARS | 0.57966 | 6.48951 | 5.14196 | 7.38E-07 | 5.61E-06 | 5.30286 |
| ARHGEF15 | 0.55137 | 1.6953 | 5.14076 | 7.42E-07 | 5.64E-06 | 5.29752 |
| STEAP3 | 0.99815 | 2.552304 | 5.14074 | 7.42E-07 | 5.64E-06 | 5.29743 |
| PKMYT1 | 0.58791 | 3.477409 | 5.14063 | 7.43E-07 | 5.64E-06 | 5.29694 |
| SLC18A3 | 0.62231 | 0.513649 | 5.14044 | 7.43E-07 | 5.64E-06 | 5.29614 |
| KIAA0930 | -0.5002 | 6.192103 | -5.1395 | 7.47E-07 | 5.66E-06 | 5.2921 |
| NKX6-1 | 0.55337 | 0.636775 | 5.13928 | 7.47E-07 | 5.67E-06 | 5.291 |
| LATS2 | 0.51948 | 1.7684 | 5.13804 | 7.52E-07 | 5.69E-06 | 5.28547 |
| TF | -1.2802 | 7.780961 | -5.136 | 7.59E-07 | 5.74E-06 | 5.27654 |
| HOXB7 | 0.90619 | 1.393703 | 5.13546 | 7.61E-07 | 5.75E-06 | 5.27407 |
| CLVS2 | -0.8607 | 2.078719 | -5.1351 | 7.62E-07 | 5.76E-06 | 5.2724 |
| RPH3A | -1.29 | 3.871721 | -5.1331 | 7.69E-07 | 5.81E-06 | 5.26375 |
| ZAP70 | 0.54027 | 1.39719 | 5.13307 | 7.69E-07 | 5.81E-06 | 5.2635 |
| APLP1 | -0.818 | 8.236796 | -5.132 | 7.73E-07 | 5.83E-06 | 5.25857 |
| RP11-869B15.1 | -0.5137 | 2.34325 | -5.1317 | 7.74E-07 | 5.84E-06 | 5.25734 |
| BCL6B | 0.61036 | 2.193501 | 5.13127 | 7.75E-07 | 5.85E-06 | 5.25551 |
| HRSP12 | -0.6896 | 6.076253 | -5.1298 | 7.81E-07 | 5.88E-06 | 5.24908 |
| DDX60L | 0.56448 | 2.698691 | 5.12908 | 7.83E-07 | 5.90E-06 | 5.24583 |
| AC093616.4 | 0.51797 | 2.257535 | 5.1278 | 7.88E-07 | 5.93E-06 | 5.24017 |
| PBX3 | 0.61979 | 3.911592 | 5.12768 | 7.88E-07 | 5.93E-06 | 5.23965 |
| CTD-2210P24.4 | -0.6505 | 2.058799 | -5.1263 | 7.93E-07 | 5.96E-06 | 5.23367 |
| SUSD2 | 0.65081 | 1.506289 | 5.12522 | 7.97E-07 | 5.99E-06 | 5.22876 |
| IL18BP | 0.59705 | 3.784264 | 5.12301 | 8.05E-07 | 6.04E-06 | 5.21899 |
| MBP | -1.9956 | 9.78837 | -5.1221 | 8.09E-07 | 6.06E-06 | 5.21482 |
| RUNDC3A | -0.9172 | 6.440221 | -5.1198 | 8.17E-07 | 6.12E-06 | 5.20492 |
| APOD | -0.8618 | 7.726228 | -5.1193 | 8.19E-07 | 6.13E-06 | 5.20243 |
| CCK | -1.6828 | 4.051708 | -5.1181 | 8.24E-07 | 6.16E-06 | 5.1973 |
| OR4N2 | 1.04623 | 1.335657 | 5.11796 | 8.24E-07 | 6.16E-06 | 5.19668 |
| RIPK3 | 0.62978 | 1.861791 | 5.11659 | 8.30E-07 | 6.19E-06 | 5.19063 |
| ERP27 | 0.54333 | 0.752529 | 5.1163 | 8.31E-07 | 6.19E-06 | 5.18936 |
| ITGA1 | 0.70746 | 2.263086 | 5.1159 | 8.32E-07 | 6.20E-06 | 5.18759 |
| TSPAN12 | 0.83172 | 4.172515 | 5.11573 | 8.33E-07 | 6.20E-06 | 5.18685 |
| CRNDE | 1.24514 | 2.400982 | 5.11566 | 8.33E-07 | 6.21E-06 | 5.1865 |
| FXYD7 | -1.3249 | 4.555761 | -5.1146 | 8.37E-07 | 6.23E-06 | 5.18204 |
| ASIC2 | -0.742 | 1.390683 | -5.1112 | 8.50E-07 | 6.32E-06 | 5.16685 |
| BARX1 | 0.51872 | 0.417346 | 5.10492 | 8.75E-07 | 6.48E-06 | 5.13916 |
| ATL3 | 0.50783 | 3.586974 | 5.10208 | 8.87E-07 | 6.56E-06 | 5.12661 |
| CHRM1 | -1.0595 | 2.448056 | -5.1014 | 8.90E-07 | 6.57E-06 | 5.12353 |
| NTN1 | 0.71133 | 4.56119 | 5.10038 | 8.94E-07 | 6.60E-06 | 5.11913 |
| COL11A1 | 1.02094 | 2.679649 | 5.0993 | 8.98E-07 | 6.63E-06 | 5.11438 |
| ARHGAP44 | -0.7794 | 2.78785 | -5.0987 | 9.00E-07 | 6.64E-06 | 5.11189 |
| HOXA9 | 0.75743 | 0.625446 | 5.09768 | 9.05E-07 | 6.67E-06 | 5.10725 |
| GNLY | 0.75716 | 1.607417 | 5.09737 | 9.06E-07 | 6.68E-06 | 5.10586 |
| CYP19A1 | 0.61654 | 1.164829 | 5.09699 | 9.08E-07 | 6.69E-06 | 5.10423 |
| DNAJC6 | -0.711 | 5.01788 | -5.0939 | 9.20E-07 | 6.77E-06 | 5.09082 |
| SLAMF8 | 0.74121 | 1.719794 | 5.09087 | 9.33E-07 | 6.86E-06 | 5.07728 |
| WNK2 | -0.9279 | 3.623329 | -5.09 | 9.37E-07 | 6.88E-06 | 5.07336 |
| CHRNA7 | -0.585 | 1.622427 | -5.0884 | 9.44E-07 | 6.92E-06 | 5.06654 |
| GNG3 | -1.5885 | 4.452132 | -5.0876 | 9.48E-07 | 6.95E-06 | 5.06278 |
| S100A1 | -1.1118 | 7.067408 | -5.0848 | 9.60E-07 | 7.04E-06 | 5.05041 |
| LZTS3 | -0.5227 | 4.929495 | -5.0846 | 9.60E-07 | 7.04E-06 | 5.04991 |
| NALCN | -0.648 | 3.684814 | -5.0844 | 9.61E-07 | 7.04E-06 | 5.04904 |
| CD3E | 0.67044 | 1.245265 | 5.08385 | 9.64E-07 | 7.06E-06 | 5.04638 |
| CHD5 | -1.088 | 2.473199 | -5.0827 | 9.69E-07 | 7.09E-06 | 5.0414 |
| PRLHR | -1.2545 | 2.169208 | -5.0812 | 9.76E-07 | 7.14E-06 | 5.03464 |
| PLOD2 | 0.766 | 4.255785 | 5.08103 | 9.76E-07 | 7.14E-06 | 5.034 |
| RP11-416I2.1 | -0.6685 | 0.706214 | -5.076 | 9.99E-07 | 7.30E-06 | 5.0121 |
| CCRL2 | 0.55138 | 1.73947 | 5.07448 | 1.01E-06 | 7.34E-06 | 5.00527 |
| SMAD4 | 0.51076 | 5.253445 | 5.07328 | 1.01E-06 | 7.38E-06 | 4.99998 |
| KANK2 | 0.61607 | 4.390596 | 5.07321 | 1.01E-06 | 7.38E-06 | 4.99968 |
| PAIP2B | -0.8006 | 3.37735 | -5.0696 | 1.03E-06 | 7.49E-06 | 4.98402 |
| NRGN | -1.7589 | 6.065147 | -5.0688 | 1.03E-06 | 7.51E-06 | 4.98045 |
| CDKL2 | -0.621 | 1.264893 | -5.0673 | 1.04E-06 | 7.55E-06 | 4.97392 |
| FAM65B | -0.6024 | 2.747696 | -5.067 | 1.04E-06 | 7.56E-06 | 4.97264 |
| JPH3 | -1.0692 | 4.292849 | -5.0665 | 1.04E-06 | 7.57E-06 | 4.97018 |
| CACNG3 | -1.1464 | 1.825826 | -5.0653 | 1.05E-06 | 7.61E-06 | 4.96499 |
| FPR3 | 0.85936 | 2.133437 | 5.06484 | 1.05E-06 | 7.62E-06 | 4.96295 |
| SYT4 | -1.2176 | 3.214615 | -5.0628 | 1.06E-06 | 7.69E-06 | 4.95391 |
| PDPN | 1.39098 | 3.680038 | 5.06253 | 1.06E-06 | 7.70E-06 | 4.95287 |
| TRAF3IP3 | 0.54841 | 2.261779 | 5.06153 | 1.07E-06 | 7.73E-06 | 4.94845 |
| RCAN1 | 0.82726 | 6.109936 | 5.06131 | 1.07E-06 | 7.73E-06 | 4.94752 |
| MVP | 0.62795 | 4.975176 | 5.05971 | 1.08E-06 | 7.78E-06 | 4.94049 |
| RP1-269M15.3 | -0.6378 | 0.893758 | -5.0592 | 1.08E-06 | 7.79E-06 | 4.93811 |
| RP3-406A7.7 | -0.8196 | 2.04877 | -5.0566 | 1.09E-06 | 7.87E-06 | 4.92679 |
| MAP3K8 | 0.5758 | 2.464926 | 5.05585 | 1.10E-06 | 7.90E-06 | 4.92359 |
| MLTK | 0.65112 | 3.063202 | 5.0533 | 1.11E-06 | 7.97E-06 | 4.91242 |
| REEP6 | -0.5642 | 3.738286 | -5.0526 | 1.11E-06 | 7.99E-06 | 4.90954 |
| STX1B | -0.7665 | 4.596512 | -5.0504 | 1.12E-06 | 8.07E-06 | 4.89982 |
| AGAP2-AS1 | 0.92982 | 1.827849 | 5.04967 | 1.13E-06 | 8.09E-06 | 4.89656 |
| NGB | -0.9201 | 1.104482 | -5.0457 | 1.15E-06 | 8.22E-06 | 4.87909 |
| MAST3 | -0.6527 | 3.840299 | -5.0456 | 1.15E-06 | 8.22E-06 | 4.87858 |
| PSD | -0.9657 | 5.562866 | -5.0455 | 1.15E-06 | 8.22E-06 | 4.87818 |
| RP11-100M12.3 | -0.519 | 0.606637 | -5.0451 | 1.15E-06 | 8.23E-06 | 4.87653 |
| EIF4E3 | -0.5118 | 3.370701 | -5.0444 | 1.15E-06 | 8.25E-06 | 4.87359 |
| LRRC46 | 0.50824 | 1.499066 | 5.04409 | 1.16E-06 | 8.26E-06 | 4.87218 |
| VCAM1 | 1.23253 | 4.04329 | 5.04177 | 1.17E-06 | 8.34E-06 | 4.86201 |
| CTB-25B13.12 | 0.51806 | 2.548174 | 5.04171 | 1.17E-06 | 8.34E-06 | 4.86178 |
| RP11-143K11.5 | -0.5226 | 0.955498 | -5.0402 | 1.18E-06 | 8.38E-06 | 4.8552 |
| NPNT | 1.04143 | 2.85467 | 5.04017 | 1.18E-06 | 8.38E-06 | 4.85506 |
| CD44 | 1.2183 | 6.097367 | 5.03895 | 1.18E-06 | 8.42E-06 | 4.84972 |
| SRRM4 | -0.7676 | 1.678401 | -5.0388 | 1.18E-06 | 8.43E-06 | 4.84894 |
| C1QA | 0.86714 | 6.954405 | 5.03749 | 1.19E-06 | 8.47E-06 | 4.84334 |
| KIF6 | -0.9283 | 2.655743 | -5.0354 | 1.20E-06 | 8.55E-06 | 4.83409 |
| GABRA5 | -1.2438 | 2.450676 | -5.0313 | 1.22E-06 | 8.69E-06 | 4.81644 |
| RNASE3 | 0.56311 | 0.891514 | 5.03006 | 1.23E-06 | 8.73E-06 | 4.81094 |
| CHPF | 0.57827 | 5.672926 | 5.02925 | 1.24E-06 | 8.75E-06 | 4.8074 |
| SOCS1 | 0.55268 | 1.37481 | 5.02821 | 1.24E-06 | 8.79E-06 | 4.80288 |
| SLC7A14 | -0.8806 | 2.816379 | -5.0275 | 1.25E-06 | 8.82E-06 | 4.79964 |
| CTBS | 0.56311 | 3.189336 | 5.0267 | 1.25E-06 | 8.85E-06 | 4.79626 |
| FAM19A2 | -0.854 | 2.64432 | -5.0267 | 1.25E-06 | 8.85E-06 | 4.79608 |
| E2F5 | 0.53201 | 3.961008 | 5.02608 | 1.25E-06 | 8.87E-06 | 4.79356 |
| STXBP2 | 0.59161 | 3.632534 | 5.02586 | 1.26E-06 | 8.87E-06 | 4.79264 |
| RBP4 | -1.0569 | 2.133748 | -5.0253 | 1.26E-06 | 8.89E-06 | 4.79035 |
| LPAR6 | 0.72967 | 4.046207 | 5.02198 | 1.28E-06 | 9.01E-06 | 4.77573 |
| WWTR1 | 0.84128 | 4.534098 | 5.02105 | 1.28E-06 | 9.04E-06 | 4.77165 |
| CLEC5A | 0.84857 | 1.061476 | 5.02046 | 1.29E-06 | 9.06E-06 | 4.76909 |
| DUSP5 | 0.78767 | 2.082784 | 5.02006 | 1.29E-06 | 9.07E-06 | 4.76734 |
| IER5L | 0.73173 | 3.060533 | 5.0191 | 1.29E-06 | 9.10E-06 | 4.76317 |
| SLC7A7 | 0.64914 | 3.184346 | 5.0186 | 1.30E-06 | 9.12E-06 | 4.76101 |
| TMEM106A | 0.52856 | 1.894065 | 5.01691 | 1.31E-06 | 9.18E-06 | 4.75365 |
| GIMAP2 | 0.56196 | 3.075852 | 5.01598 | 1.31E-06 | 9.22E-06 | 4.74961 |
| HIST1H2AK | 0.50921 | 1.303079 | 5.01579 | 1.31E-06 | 9.22E-06 | 4.74875 |
| SCARF2 | 0.53295 | 2.930755 | 5.0139 | 1.33E-06 | 9.29E-06 | 4.74054 |
| ALPK2 | 0.76566 | 0.822565 | 5.00799 | 1.36E-06 | 9.51E-06 | 4.71484 |
| PAQR8 | -0.8161 | 5.305572 | -5.0074 | 1.37E-06 | 9.53E-06 | 4.71222 |
| UTS2 | 0.5083 | 0.698967 | 5.00671 | 1.37E-06 | 9.56E-06 | 4.70929 |
| CTB-43E15.4 | -0.8917 | 1.705798 | -5.0064 | 1.37E-06 | 9.56E-06 | 4.70809 |
| EPB41 | 0.61946 | 3.551928 | 5.00508 | 1.38E-06 | 9.60E-06 | 4.7022 |
| DPYD | 0.75935 | 2.576165 | 5.00429 | 1.38E-06 | 9.63E-06 | 4.69878 |
| KCNN4 | 0.57761 | 1.292989 | 5.00164 | 1.40E-06 | 9.72E-06 | 4.68728 |
| HIST1H1D | 0.51786 | 0.468182 | 5.00074 | 1.41E-06 | 9.76E-06 | 4.68336 |
| RCN3 | 0.61733 | 3.183625 | 5.00012 | 1.41E-06 | 9.77E-06 | 4.68068 |
| FBXL16 | -0.9798 | 5.597293 | -4.9922 | 1.46E-06 | 1.01E-05 | 4.64611 |
| BSN | -0.7308 | 2.769321 | -4.9912 | 1.47E-06 | 1.02E-05 | 4.64206 |
| AC018647.3 | -0.9226 | 4.107542 | -4.9872 | 1.50E-06 | 1.03E-05 | 4.62457 |
| MYL12A | 0.65849 | 5.944865 | 4.98708 | 1.50E-06 | 1.03E-05 | 4.6241 |
| RLTPR | -0.8239 | 2.388813 | -4.9865 | 1.50E-06 | 1.03E-05 | 4.62173 |
| DDX25 | -0.8202 | 3.914252 | -4.9856 | 1.51E-06 | 1.04E-05 | 4.61763 |
| AJAP1 | -0.6127 | 1.918794 | -4.9855 | 1.51E-06 | 1.04E-05 | 4.61728 |
| SH3BGRL2 | -0.5312 | 3.47225 | -4.9833 | 1.52E-06 | 1.05E-05 | 4.60785 |
| NTNG2 | -0.548 | 3.892032 | -4.9789 | 1.55E-06 | 1.06E-05 | 4.58882 |
| PXDN | 0.7667 | 4.422478 | 4.97853 | 1.56E-06 | 1.07E-05 | 4.5871 |
| TNFAIP6 | 0.78401 | 1.521055 | 4.97712 | 1.57E-06 | 1.07E-05 | 4.581 |
| ARHGAP25 | 0.54354 | 2.973319 | 4.97601 | 1.57E-06 | 1.08E-05 | 4.57617 |
| LEPREL1 | 0.75427 | 2.431196 | 4.97401 | 1.59E-06 | 1.08E-05 | 4.56753 |
| TRIM5 | 0.54668 | 3.034703 | 4.97204 | 1.60E-06 | 1.09E-05 | 4.55903 |
| CRTAC1 | -1.0672 | 4.633322 | -4.9708 | 1.61E-06 | 1.10E-05 | 4.5535 |
| GBP5 | 0.69166 | 1.252719 | 4.97007 | 1.62E-06 | 1.10E-05 | 4.55052 |
| CLEC12A | 0.62113 | 0.898133 | 4.96823 | 1.63E-06 | 1.11E-05 | 4.54257 |
| MOAP1 | -0.5026 | 5.671392 | -4.9656 | 1.65E-06 | 1.12E-05 | 4.53112 |
| CPNE6 | -1.207 | 3.302634 | -4.9655 | 1.65E-06 | 1.12E-05 | 4.53055 |
| SP110 | 0.53059 | 3.961436 | 4.96531 | 1.65E-06 | 1.12E-05 | 4.52996 |
| SLC6A17 | -1.1902 | 2.530222 | -4.9644 | 1.66E-06 | 1.13E-05 | 4.52599 |
| AC034220.3 | -0.5998 | 2.908457 | -4.9643 | 1.66E-06 | 1.13E-05 | 4.52539 |
| SLC1A4 | -0.7062 | 5.606783 | -4.9642 | 1.66E-06 | 1.13E-05 | 4.52529 |
| SNCA | -1.0731 | 5.334613 | -4.9617 | 1.68E-06 | 1.14E-05 | 4.51454 |
| HOXA11 | 0.53073 | 0.520845 | 4.96098 | 1.68E-06 | 1.14E-05 | 4.51123 |
| EIF4E1B | -0.5667 | 0.840958 | -4.961 | 1.68E-06 | 1.14E-05 | 4.51118 |
| KLF10 | 0.66545 | 3.617585 | 4.96078 | 1.68E-06 | 1.14E-05 | 4.51037 |
| ARPC1B | 0.67981 | 5.798019 | 4.9605 | 1.69E-06 | 1.14E-05 | 4.50918 |
| STEAP2 | -0.6279 | 2.135924 | -4.9598 | 1.69E-06 | 1.14E-05 | 4.50614 |
| WI2-1896O14.1 | -0.8827 | 3.957073 | -4.9585 | 1.70E-06 | 1.15E-05 | 4.50072 |
| C1RL | 0.79136 | 2.985247 | 4.95339 | 1.74E-06 | 1.17E-05 | 4.4785 |
| RP11-297M9.2 | -0.8508 | 1.824531 | -4.9477 | 1.79E-06 | 1.20E-05 | 4.4538 |
| ERMN | -1.5509 | 5.183846 | -4.9464 | 1.80E-06 | 1.21E-05 | 4.44826 |
| NKX2-5 | 0.61836 | 0.564469 | 4.94632 | 1.80E-06 | 1.21E-05 | 4.44804 |
| RAB3A | -1.0763 | 4.957154 | -4.945 | 1.81E-06 | 1.21E-05 | 4.44254 |
| CALHM2 | 0.50173 | 3.18402 | 4.94196 | 1.83E-06 | 1.23E-05 | 4.42925 |
| SYT13 | -1.233 | 2.657985 | -4.9418 | 1.84E-06 | 1.23E-05 | 4.42839 |
| GPIHBP1 | -0.8449 | 2.797785 | -4.9417 | 1.84E-06 | 1.23E-05 | 4.4281 |
| SSTR2 | -0.9221 | 3.486178 | -4.9402 | 1.85E-06 | 1.24E-05 | 4.42174 |
| TCEAL5 | -0.7456 | 5.138677 | -4.9398 | 1.85E-06 | 1.24E-05 | 4.42013 |
| TRIP10 | 0.53201 | 3.923199 | 4.93895 | 1.86E-06 | 1.24E-05 | 4.4163 |
| ARHGAP15 | 0.56809 | 2.30512 | 4.93669 | 1.88E-06 | 1.26E-05 | 4.4066 |
| SH2D5 | -0.964 | 1.816285 | -4.9328 | 1.91E-06 | 1.28E-05 | 4.38968 |
| MADCAM1 | -0.5287 | 1.535003 | -4.9291 | 1.94E-06 | 1.29E-05 | 4.37408 |
| CCNE2 | 0.56802 | 2.643639 | 4.92898 | 1.94E-06 | 1.29E-05 | 4.37347 |
| COL18A1 | 0.69235 | 4.517108 | 4.9283 | 1.95E-06 | 1.30E-05 | 4.37057 |
| C2orf82 | -1.1179 | 3.59522 | -4.9257 | 1.97E-06 | 1.31E-05 | 4.35928 |
| MAGEE1 | -0.6549 | 3.095549 | -4.9225 | 2.00E-06 | 1.33E-05 | 4.34569 |
| RFTN1 | 0.76825 | 4.113098 | 4.92247 | 2.00E-06 | 1.33E-05 | 4.34554 |
| CHN1 | -0.8894 | 6.539984 | -4.9215 | 2.01E-06 | 1.33E-05 | 4.34149 |
| RP11-84C10.2 | 0.62786 | 0.878098 | 4.92004 | 2.02E-06 | 1.34E-05 | 4.33507 |
| EPHA1 | 0.53977 | 1.292887 | 4.91912 | 2.03E-06 | 1.35E-05 | 4.33115 |
| RAB13 | 0.57627 | 6.627661 | 4.91633 | 2.06E-06 | 1.36E-05 | 4.31917 |
| LINC00957 | -0.799 | 2.276424 | -4.9154 | 2.07E-06 | 1.36E-05 | 4.31514 |
| SOX12 | 0.50885 | 4.520921 | 4.91359 | 2.08E-06 | 1.37E-05 | 4.30746 |
| KCNH3 | -1.0064 | 2.782304 | -4.9116 | 2.10E-06 | 1.38E-05 | 4.29881 |
| SNORD3B-2 | 0.75512 | 0.847815 | 4.91115 | 2.10E-06 | 1.39E-05 | 4.297 |
| LINC00960 | 0.67586 | 1.576389 | 4.90994 | 2.12E-06 | 1.39E-05 | 4.29182 |
| RDH5 | 0.55602 | 2.86607 | 4.90878 | 2.13E-06 | 1.40E-05 | 4.28684 |
| PLB1 | 0.58655 | 2.164067 | 4.90815 | 2.13E-06 | 1.40E-05 | 4.28414 |
| ATP2A3 | 0.51675 | 1.521338 | 4.90606 | 2.15E-06 | 1.41E-05 | 4.27521 |
| ARHGEF26-AS1 | -0.6959 | 4.537017 | -4.9055 | 2.16E-06 | 1.42E-05 | 4.27259 |
| KIAA0513 | -0.61 | 4.543876 | -4.9045 | 2.17E-06 | 1.42E-05 | 4.26833 |
| LOXL3 | 0.57758 | 3.506222 | 4.90345 | 2.18E-06 | 1.43E-05 | 4.26403 |
| SLC17A7 | -1.8235 | 4.455761 | -4.9009 | 2.20E-06 | 1.44E-05 | 4.25333 |
| HAPLN2 | -1.3311 | 5.269041 | -4.8973 | 2.24E-06 | 1.46E-05 | 4.23787 |
| CDC42EP3 | 0.52023 | 2.643921 | 4.89643 | 2.25E-06 | 1.47E-05 | 4.23402 |
| IRF7 | 0.68286 | 3.603753 | 4.8964 | 2.25E-06 | 1.47E-05 | 4.23389 |
| RP11-826N14.2 | -0.5135 | 0.619207 | -4.8963 | 2.25E-06 | 1.47E-05 | 4.23354 |
| S1PR3 | 0.93775 | 3.658468 | 4.89615 | 2.25E-06 | 1.47E-05 | 4.23282 |
| FAM163B | -1.2797 | 3.421795 | -4.8939 | 2.27E-06 | 1.48E-05 | 4.22335 |
| PCDHB16 | 0.73877 | 2.190426 | 4.89204 | 2.29E-06 | 1.49E-05 | 4.21526 |
| STAC | 0.72317 | 0.951325 | 4.89161 | 2.30E-06 | 1.50E-05 | 4.21343 |
| NSG2 | -1.3448 | 6.079631 | -4.8904 | 2.31E-06 | 1.50E-05 | 4.20839 |
| EVA1A | 0.58603 | 1.510373 | 4.88914 | 2.32E-06 | 1.51E-05 | 4.20285 |
| CDK5R2 | -1.0173 | 3.201755 | -4.8889 | 2.32E-06 | 1.51E-05 | 4.20195 |
| PCED1B | 0.52817 | 1.826367 | 4.88825 | 2.33E-06 | 1.52E-05 | 4.19907 |
| CXCL9 | 0.68956 | 0.931502 | 4.88779 | 2.34E-06 | 1.52E-05 | 4.19709 |
| HOXD13 | 0.54998 | 0.40051 | 4.8875 | 2.34E-06 | 1.52E-05 | 4.19587 |
| ADIRF | -0.9747 | 4.893713 | -4.8871 | 2.34E-06 | 1.52E-05 | 4.19397 |
| SNORD3D | 0.79839 | 0.919759 | 4.88699 | 2.34E-06 | 1.52E-05 | 4.1937 |
| VAMP1 | -0.6722 | 4.178383 | -4.8858 | 2.36E-06 | 1.53E-05 | 4.18874 |
| DCTN1-AS1 | -0.652 | 1.238256 | -4.8858 | 2.36E-06 | 1.53E-05 | 4.18871 |
| FAM153B | -0.7891 | 1.22382 | -4.8847 | 2.37E-06 | 1.54E-05 | 4.18396 |
| CEND1 | -0.75 | 5.556285 | -4.8842 | 2.37E-06 | 1.54E-05 | 4.18173 |
| ABRACL | 0.58922 | 3.45081 | 4.88403 | 2.38E-06 | 1.54E-05 | 4.18106 |
| AC010441.1 | 0.70359 | 3.615197 | 4.88353 | 2.38E-06 | 1.54E-05 | 4.17893 |
| PRRX1 | 0.68285 | 5.412149 | 4.88105 | 2.41E-06 | 1.56E-05 | 4.16834 |
| PEBP4 | -0.658 | 2.255767 | -4.8789 | 2.43E-06 | 1.57E-05 | 4.1592 |
| HOXD8 | 1.09502 | 1.565447 | 4.87825 | 2.44E-06 | 1.57E-05 | 4.15641 |
| SLC40A1 | 0.63446 | 4.486727 | 4.87641 | 2.46E-06 | 1.58E-05 | 4.14856 |
| SLC26A4-AS1 | -1.0882 | 1.82397 | -4.8759 | 2.46E-06 | 1.59E-05 | 4.14649 |
| FCGR2A | 0.88102 | 4.384453 | 4.87452 | 2.48E-06 | 1.60E-05 | 4.14051 |
| TRDC | 0.78918 | 1.021665 | 4.86848 | 2.55E-06 | 1.63E-05 | 4.11482 |
| KDELC2 | 0.58584 | 2.970651 | 4.86618 | 2.57E-06 | 1.65E-05 | 4.10501 |
| KIF5A | -0.9757 | 6.354168 | -4.8614 | 2.63E-06 | 1.68E-05 | 4.08489 |
| NKG7 | 0.67844 | 1.727182 | 4.8578 | 2.67E-06 | 1.70E-05 | 4.0694 |
| VIPR1 | -0.6538 | 1.960654 | -4.8572 | 2.68E-06 | 1.71E-05 | 4.0669 |
| ASAP3 | 0.63642 | 4.366824 | 4.85635 | 2.69E-06 | 1.71E-05 | 4.06325 |
| HIST1H1C | 0.71089 | 3.828142 | 4.85503 | 2.70E-06 | 1.72E-05 | 4.05764 |
| VWA5B2 | -0.7222 | 2.702173 | -4.8512 | 2.75E-06 | 1.75E-05 | 4.04142 |
| ARID5A | 0.60994 | 4.168329 | 4.85097 | 2.75E-06 | 1.75E-05 | 4.04039 |
| SNORD3B-1 | 0.79044 | 1.221953 | 4.85073 | 2.75E-06 | 1.75E-05 | 4.03939 |
| CNN2 | 0.73512 | 3.658754 | 4.84812 | 2.79E-06 | 1.77E-05 | 4.0283 |
| GMFG | 0.60051 | 4.535339 | 4.84677 | 2.80E-06 | 1.78E-05 | 4.02257 |
| EFHD1 | -0.8157 | 6.458608 | -4.8438 | 2.84E-06 | 1.80E-05 | 4.0098 |
| KLHDC8A | 0.95956 | 4.301458 | 4.84134 | 2.87E-06 | 1.82E-05 | 3.99956 |
| NOTCH3 | 0.58504 | 3.984062 | 4.84099 | 2.88E-06 | 1.82E-05 | 3.99807 |
| HLA-B | 0.88241 | 7.81547 | 4.84049 | 2.88E-06 | 1.82E-05 | 3.99595 |
| C1QB | 0.84074 | 7.61579 | 4.8391 | 2.90E-06 | 1.83E-05 | 3.99006 |
| PRICKLE3 | 0.51181 | 1.391526 | 4.83832 | 2.91E-06 | 1.84E-05 | 3.98677 |
| MUSTN1 | -0.7658 | 2.53193 | -4.8368 | 2.93E-06 | 1.85E-05 | 3.98027 |
| IL7R | 0.60111 | 1.16508 | 4.83557 | 2.94E-06 | 1.86E-05 | 3.97514 |
| ABI3 | 0.65484 | 3.160977 | 4.83452 | 2.96E-06 | 1.86E-05 | 3.97066 |
| FERMT3 | 0.66792 | 3.555059 | 4.83239 | 2.99E-06 | 1.88E-05 | 3.96166 |
| C15orf48 | 0.56287 | 0.769905 | 4.83215 | 2.99E-06 | 1.88E-05 | 3.96064 |
| GOT1 | -0.6288 | 5.337108 | -4.831 | 3.00E-06 | 1.89E-05 | 3.95587 |
| TP53TG5 | -0.7255 | 1.504821 | -4.8287 | 3.04E-06 | 1.91E-05 | 3.9461 |
| HOXC10 | 0.93195 | 0.929533 | 4.82799 | 3.05E-06 | 1.91E-05 | 3.94303 |
| SLC43A3 | 0.76587 | 3.228175 | 4.82544 | 3.08E-06 | 1.93E-05 | 3.93227 |
| DNAJC22 | 0.51862 | 1.344714 | 4.82358 | 3.11E-06 | 1.95E-05 | 3.92439 |
| NEFH | -1.0435 | 2.532883 | -4.8228 | 3.12E-06 | 1.95E-05 | 3.92112 |
| FRMPD4 | -0.7063 | 1.127732 | -4.8218 | 3.13E-06 | 1.96E-05 | 3.91669 |
| SELL | -1.4504 | 4.733082 | -4.8207 | 3.14E-06 | 1.96E-05 | 3.91228 |
| GRIA1 | -0.9121 | 5.401623 | -4.8203 | 3.15E-06 | 1.97E-05 | 3.91073 |
| PITX1 | 0.62597 | 0.7358 | 4.81885 | 3.17E-06 | 1.98E-05 | 3.90441 |
| PRKCDBP | 0.65923 | 2.206519 | 4.81576 | 3.21E-06 | 2.00E-05 | 3.89136 |
| CD244 | 0.51275 | 0.772909 | 4.81501 | 3.22E-06 | 2.01E-05 | 3.88823 |
| RIMS3 | -0.9041 | 3.540133 | -4.8134 | 3.25E-06 | 2.02E-05 | 3.88155 |
| ADM | 0.97373 | 2.954205 | 4.8133 | 3.25E-06 | 2.02E-05 | 3.88098 |
| CAB39L | -0.5034 | 4.690883 | -4.8105 | 3.29E-06 | 2.05E-05 | 3.8693 |
| GPR1 | 0.53129 | 0.527396 | 4.8102 | 3.29E-06 | 2.05E-05 | 3.86793 |
| SYNPR | -1.2833 | 2.692825 | -4.8101 | 3.29E-06 | 2.05E-05 | 3.86771 |
| STK36 | 0.54646 | 5.087533 | 4.80898 | 3.31E-06 | 2.06E-05 | 3.86277 |
| FRMD3 | 0.70787 | 3.510507 | 4.80611 | 3.35E-06 | 2.08E-05 | 3.85069 |
| SIX5 | 0.50399 | 1.984716 | 4.80598 | 3.36E-06 | 2.08E-05 | 3.85011 |
| OTUD7A | -0.5136 | 2.620846 | -4.8052 | 3.37E-06 | 2.09E-05 | 3.84667 |
| POU3F2 | 0.52409 | 4.513012 | 4.80512 | 3.37E-06 | 2.09E-05 | 3.84652 |
| RP11-624M8.1 | -0.6844 | 1.924221 | -4.8048 | 3.37E-06 | 2.09E-05 | 3.84519 |
| AGTRAP | 0.58219 | 4.692718 | 4.80466 | 3.37E-06 | 2.09E-05 | 3.84458 |
| IL4R | 0.50929 | 3.194894 | 4.80348 | 3.39E-06 | 2.10E-05 | 3.83961 |
| RBFOX1 | -1.2008 | 2.917698 | -4.803 | 3.40E-06 | 2.10E-05 | 3.83766 |
| MARVELD1 | 0.65715 | 2.540149 | 4.80172 | 3.42E-06 | 2.11E-05 | 3.83219 |
| IFI44 | 0.72256 | 4.698855 | 4.80132 | 3.42E-06 | 2.12E-05 | 3.83052 |
| WDFY3-AS2 | -0.5279 | 3.525232 | -4.8008 | 3.43E-06 | 2.12E-05 | 3.82832 |
| CABLES1 | -0.7117 | 3.631411 | -4.7996 | 3.45E-06 | 2.13E-05 | 3.8232 |
| PAPLN | -0.788 | 3.200376 | -4.7994 | 3.45E-06 | 2.13E-05 | 3.82224 |
| PAX3 | 0.73315 | 0.60763 | 4.7992 | 3.46E-06 | 2.13E-05 | 3.8216 |
| STXBP1 | -0.7503 | 5.951553 | -4.7971 | 3.49E-06 | 2.15E-05 | 3.81287 |
| BZRAP1 | -0.679 | 5.278567 | -4.7946 | 3.53E-06 | 2.17E-05 | 3.8024 |
| ACN9 | 0.55034 | 4.119057 | 4.79096 | 3.58E-06 | 2.20E-05 | 3.78691 |
| FAM201A | -0.63 | 1.598608 | -4.7903 | 3.59E-06 | 2.20E-05 | 3.7843 |
| ANPEP | 0.59333 | 1.738196 | 4.78912 | 3.61E-06 | 2.21E-05 | 3.77918 |
| RP11-2E11.9 | -0.8382 | 2.965637 | -4.7889 | 3.62E-06 | 2.21E-05 | 3.77834 |
| ADRA1A | -0.6894 | 2.45748 | -4.7875 | 3.64E-06 | 2.22E-05 | 3.7724 |
| NRIP2 | -0.5173 | 2.249102 | -4.7847 | 3.68E-06 | 2.25E-05 | 3.7606 |
| COL15A1 | 0.66659 | 1.235701 | 4.78383 | 3.70E-06 | 2.26E-05 | 3.75697 |
| DSEL | 0.54748 | 3.461293 | 4.78313 | 3.71E-06 | 2.26E-05 | 3.75404 |
| ESAM | 0.53315 | 4.404958 | 4.78277 | 3.72E-06 | 2.26E-05 | 3.75251 |
| TUB | -0.6529 | 4.898297 | -4.7827 | 3.72E-06 | 2.26E-05 | 3.75233 |
| HIST1H1B | 0.64894 | 0.621506 | 4.7827 | 3.72E-06 | 2.26E-05 | 3.75223 |
| DENND1C | 0.55454 | 2.084041 | 4.77968 | 3.77E-06 | 2.29E-05 | 3.73953 |
| SLC29A2 | -0.6623 | 2.676239 | -4.7795 | 3.77E-06 | 2.29E-05 | 3.73876 |
| CTD-2081K17.2 | -1.0009 | 2.24979 | -4.7758 | 3.83E-06 | 2.33E-05 | 3.72328 |
| KCNJ9 | -0.7782 | 4.38409 | -4.7742 | 3.86E-06 | 2.34E-05 | 3.71643 |
| GPC2 | 0.73848 | 3.603353 | 4.77152 | 3.90E-06 | 2.37E-05 | 3.70531 |
| ADAMTSL4 | 0.56714 | 1.479476 | 4.77135 | 3.91E-06 | 2.37E-05 | 3.7046 |
| RP11-1263C18.1 | -0.8135 | 1.736532 | -4.7702 | 3.93E-06 | 2.38E-05 | 3.69988 |
| GPR61 | -0.5509 | 1.569514 | -4.7624 | 4.06E-06 | 2.45E-05 | 3.66699 |
| MFSD4 | -0.741 | 3.580916 | -4.7622 | 4.07E-06 | 2.45E-05 | 3.66614 |
| HSPA2 | -1.0115 | 5.66171 | -4.762 | 4.07E-06 | 2.46E-05 | 3.66551 |
| ATP1B1 | -0.8329 | 6.861746 | -4.7612 | 4.08E-06 | 2.46E-05 | 3.6622 |
| CD69 | 0.70317 | 1.475078 | 4.76062 | 4.09E-06 | 2.47E-05 | 3.65969 |
| SLC25A43 | 0.57478 | 2.399661 | 4.76017 | 4.10E-06 | 2.47E-05 | 3.65778 |
| LA16c-329F2.1 | -0.5706 | 0.731482 | -4.7557 | 4.18E-06 | 2.52E-05 | 3.63892 |
| HLA-DMA | 0.89238 | 5.219035 | 4.75237 | 4.24E-06 | 2.55E-05 | 3.62517 |
| PDE1A | -0.7878 | 3.037905 | -4.7517 | 4.26E-06 | 2.56E-05 | 3.62255 |
| ADAM11 | -0.7277 | 2.639481 | -4.751 | 4.27E-06 | 2.56E-05 | 3.61951 |
| EVC | 0.55558 | 1.617833 | 4.74367 | 4.41E-06 | 2.64E-05 | 3.58884 |
| RIMBP2 | -0.8359 | 2.12022 | -4.7418 | 4.45E-06 | 2.65E-05 | 3.58087 |
| AC004019.13 | -0.5327 | 1.15438 | -4.7391 | 4.50E-06 | 2.68E-05 | 3.56995 |
| RP11-513M16.8 | -0.5666 | 2.669285 | -4.7374 | 4.53E-06 | 2.70E-05 | 3.56259 |
| LDHA | 0.76558 | 6.987981 | 4.73669 | 4.55E-06 | 2.71E-05 | 3.55973 |
| LRRC7 | -0.7271 | 2.119855 | -4.7345 | 4.59E-06 | 2.73E-05 | 3.55055 |
| KB-1517D11.4 | -0.5497 | 0.641864 | -4.7314 | 4.65E-06 | 2.76E-05 | 3.53792 |
| KCNA1 | -0.7626 | 1.455639 | -4.7314 | 4.65E-06 | 2.76E-05 | 3.53788 |
| FUOM | 0.54876 | 3.00483 | 4.7293 | 4.69E-06 | 2.79E-05 | 3.52896 |
| RP1-261G23.7 | 1.65953 | 4.074161 | 4.72918 | 4.70E-06 | 2.79E-05 | 3.52848 |
| LAMC3 | 0.67607 | 2.527264 | 4.72623 | 4.76E-06 | 2.82E-05 | 3.51618 |
| RIMS2 | -0.8546 | 2.908057 | -4.726 | 4.76E-06 | 2.82E-05 | 3.51541 |
| CA9 | 0.96697 | 1.308972 | 4.72487 | 4.79E-06 | 2.83E-05 | 3.51055 |
| TRHDE-AS1 | -0.5584 | 0.997224 | -4.7244 | 4.79E-06 | 2.84E-05 | 3.50875 |
| HAS2-AS1 | 0.6329 | 1.732139 | 4.72359 | 4.81E-06 | 2.85E-05 | 3.50519 |
| GABRA1 | -1.3217 | 2.636628 | -4.7214 | 4.86E-06 | 2.87E-05 | 3.4961 |
| C2 | 0.72925 | 3.251727 | 4.71868 | 4.92E-06 | 2.90E-05 | 3.48482 |
| DLG2 | -0.6497 | 4.145516 | -4.7186 | 4.92E-06 | 2.90E-05 | 3.48437 |
| BTN3A3 | 0.53329 | 4.294371 | 4.71532 | 4.99E-06 | 2.94E-05 | 3.47085 |
| NCF4 | 0.57873 | 2.961169 | 4.7151 | 4.99E-06 | 2.94E-05 | 3.46991 |
| RP11-977G19.10 | 0.8447 | 1.13279 | 4.71508 | 4.99E-06 | 2.94E-05 | 3.46984 |
| CD74 | 1.01282 | 9.438264 | 4.71466 | 5.00E-06 | 2.95E-05 | 3.46808 |
| NECAB2 | -0.8085 | 3.865005 | -4.7128 | 5.04E-06 | 2.97E-05 | 3.46035 |
| INMT | 0.69113 | 1.119099 | 4.71163 | 5.07E-06 | 2.98E-05 | 3.45553 |
| ZDHHC12 | 0.5515 | 3.265451 | 4.71071 | 5.09E-06 | 2.99E-05 | 3.4517 |
| PI15 | 0.51065 | 0.781927 | 4.7104 | 5.10E-06 | 3.00E-05 | 3.45042 |
| SYT7 | -1.1121 | 3.221298 | -4.71 | 5.10E-06 | 3.00E-05 | 3.44894 |
| SHMT2 | 0.63181 | 6.174058 | 4.70916 | 5.12E-06 | 3.01E-05 | 3.44525 |
| EDIL3 | -0.9899 | 4.895461 | -4.7088 | 5.13E-06 | 3.01E-05 | 3.44392 |
| SNPH | -0.5596 | 3.710465 | -4.708 | 5.15E-06 | 3.02E-05 | 3.44033 |
| RIMS1 | -0.8505 | 2.590464 | -4.7078 | 5.15E-06 | 3.02E-05 | 3.43945 |
| ECM1 | 0.52583 | 3.129441 | 4.70677 | 5.18E-06 | 3.04E-05 | 3.43536 |
| RP1-74M1.3 | 0.54636 | 1.013827 | 4.70521 | 5.21E-06 | 3.05E-05 | 3.42891 |
| RP13-131K19.6 | 0.54873 | 0.969222 | 4.70511 | 5.21E-06 | 3.05E-05 | 3.42846 |
| RP11-698N11.2 | 0.77413 | 1.912201 | 4.70433 | 5.23E-06 | 3.06E-05 | 3.42522 |
| NETO2 | 0.79948 | 3.693765 | 4.70275 | 5.27E-06 | 3.08E-05 | 3.41867 |
| HILS1 | 0.61538 | 0.786987 | 4.70209 | 5.28E-06 | 3.09E-05 | 3.41595 |
| ATP10A | -0.5257 | 2.562992 | -4.7011 | 5.31E-06 | 3.10E-05 | 3.41177 |
| HLA-DQA2 | 0.80582 | 1.125245 | 4.69839 | 5.37E-06 | 3.13E-05 | 3.40064 |
| ZNF560 | 0.74496 | 1.175232 | 4.69666 | 5.41E-06 | 3.16E-05 | 3.39344 |
| GRB14 | 0.81427 | 1.45995 | 4.69581 | 5.43E-06 | 3.17E-05 | 3.38995 |
| AC013268.5 | -0.9053 | 1.354051 | -4.6946 | 5.46E-06 | 3.18E-05 | 3.38477 |
| NEFL | -1.6098 | 3.536503 | -4.6941 | 5.47E-06 | 3.19E-05 | 3.38267 |
| ITGA3 | 0.66292 | 3.683176 | 4.69383 | 5.48E-06 | 3.19E-05 | 3.38175 |
| FBXO41 | -0.657 | 3.536938 | -4.6927 | 5.50E-06 | 3.20E-05 | 3.37723 |
| RP3-428L16.2 | 0.74016 | 3.145138 | 4.69255 | 5.51E-06 | 3.20E-05 | 3.37646 |
| RYR2 | -0.7828 | 1.645039 | -4.6911 | 5.54E-06 | 3.22E-05 | 3.37027 |
| SLC5A11 | -1.026 | 2.677986 | -4.6907 | 5.55E-06 | 3.23E-05 | 3.36881 |
| FKBP10 | 0.67272 | 5.787879 | 4.6892 | 5.59E-06 | 3.24E-05 | 3.36258 |
| RHBDF1 | 0.53789 | 3.491273 | 4.68909 | 5.59E-06 | 3.24E-05 | 3.36214 |
| SVIL | 0.60569 | 2.138625 | 4.68892 | 5.59E-06 | 3.24E-05 | 3.36142 |
| 9-Mar | 0.71589 | 4.41489 | 4.68767 | 5.62E-06 | 3.26E-05 | 3.35624 |
| MAL2 | -1.0893 | 1.937734 | -4.6877 | 5.62E-06 | 3.26E-05 | 3.35622 |
| LRRC25 | 0.60414 | 2.22375 | 4.68638 | 5.65E-06 | 3.27E-05 | 3.35094 |
| TAGLN3 | -0.9281 | 5.906667 | -4.6796 | 5.82E-06 | 3.36E-05 | 3.32289 |
| RP11-127B20.2 | -0.6137 | 2.077335 | -4.6769 | 5.89E-06 | 3.39E-05 | 3.31189 |
| RTN4R | -0.8317 | 3.052352 | -4.6722 | 6.01E-06 | 3.46E-05 | 3.29249 |
| GLT1D1 | -0.8326 | 1.723761 | -4.6712 | 6.04E-06 | 3.47E-05 | 3.28846 |
| KIAA1598 | -0.7945 | 4.945886 | -4.6693 | 6.09E-06 | 3.50E-05 | 3.28044 |
| TNFRSF11B | 0.79391 | 1.409929 | 4.66914 | 6.09E-06 | 3.50E-05 | 3.27976 |
| TGFB1 | 0.56228 | 4.707583 | 4.66889 | 6.10E-06 | 3.50E-05 | 3.27875 |
| BDKRB2 | 0.51942 | 0.818908 | 4.66751 | 6.14E-06 | 3.52E-05 | 3.27303 |
| NRXN1 | -0.782 | 5.670885 | -4.6665 | 6.16E-06 | 3.53E-05 | 3.26899 |
| NAP1L3 | -0.6375 | 5.625472 | -4.6641 | 6.23E-06 | 3.57E-05 | 3.25885 |
| KCNJ4 | -1.0707 | 2.570707 | -4.6636 | 6.24E-06 | 3.57E-05 | 3.25709 |
| KRT18 | 0.70376 | 0.999009 | 4.65907 | 6.36E-06 | 3.64E-05 | 3.2383 |
| OLFML2A | 0.61596 | 1.72751 | 4.65863 | 6.37E-06 | 3.64E-05 | 3.2365 |
| PLXDC1 | 0.6054 | 4.077997 | 4.65763 | 6.40E-06 | 3.66E-05 | 3.23235 |
| CAMSAP3 | -0.7645 | 2.66385 | -4.6564 | 6.44E-06 | 3.67E-05 | 3.22727 |
| SLC25A27 | -0.5478 | 4.587216 | -4.6532 | 6.52E-06 | 3.71E-05 | 3.21433 |
| NUAK2 | 0.51429 | 1.290699 | 4.65039 | 6.60E-06 | 3.76E-05 | 3.20262 |
| BCL2A1 | 0.86735 | 1.967359 | 4.6502 | 6.61E-06 | 3.76E-05 | 3.20182 |
| FGFBP2 | 0.76196 | 1.46881 | 4.64873 | 6.65E-06 | 3.78E-05 | 3.19579 |
| PYGM | -0.7766 | 2.649758 | -4.6474 | 6.69E-06 | 3.80E-05 | 3.19043 |
| RND1 | -0.6529 | 4.207512 | -4.6472 | 6.70E-06 | 3.80E-05 | 3.18956 |
| IL2RG | 0.59555 | 1.928414 | 4.6468 | 6.71E-06 | 3.80E-05 | 3.18786 |
| RASAL1 | -0.9499 | 2.07649 | -4.6457 | 6.74E-06 | 3.82E-05 | 3.18343 |
| UNC80 | -0.6032 | 3.023161 | -4.645 | 6.76E-06 | 3.83E-05 | 3.18038 |
| LIPE | -0.7827 | 4.403629 | -4.6437 | 6.80E-06 | 3.85E-05 | 3.17525 |
| CKMT1B | -1.0337 | 3.479556 | -4.6436 | 6.80E-06 | 3.85E-05 | 3.17459 |
| EMP3 | 1.21992 | 3.952755 | 4.64221 | 6.84E-06 | 3.87E-05 | 3.16901 |
| ELN | 0.73047 | 5.034733 | 4.64192 | 6.85E-06 | 3.87E-05 | 3.1678 |
| PLK3 | 0.54788 | 3.351905 | 4.63727 | 6.99E-06 | 3.94E-05 | 3.14876 |
| RP11-1035H13.3 | 0.70117 | 1.163312 | 4.63667 | 7.01E-06 | 3.95E-05 | 3.14631 |
| TBR1 | -0.9618 | 1.788126 | -4.6351 | 7.05E-06 | 3.97E-05 | 3.13979 |
| UNC5A | -0.8485 | 3.321224 | -4.6325 | 7.13E-06 | 4.01E-05 | 3.12919 |
| UCP2 | 0.82308 | 5.021708 | 4.63116 | 7.17E-06 | 4.03E-05 | 3.12369 |
| TDRD9 | -0.6748 | 1.724039 | -4.6299 | 7.21E-06 | 4.05E-05 | 3.1184 |
| EMX1 | -0.8324 | 1.359463 | -4.6297 | 7.22E-06 | 4.05E-05 | 3.11791 |
| TLR3 | 0.53263 | 2.355468 | 4.62918 | 7.23E-06 | 4.06E-05 | 3.11559 |
| C1QC | 0.78136 | 7.347106 | 4.62885 | 7.25E-06 | 4.06E-05 | 3.11426 |
| GRIN2B | -0.6511 | 1.177191 | -4.6275 | 7.29E-06 | 4.08E-05 | 3.10877 |
| THRB | -0.6018 | 3.232334 | -4.6269 | 7.31E-06 | 4.09E-05 | 3.10611 |
| SHANK1 | -0.9212 | 3.420274 | -4.6261 | 7.33E-06 | 4.11E-05 | 3.10293 |
| RXFP1 | -0.6587 | 1.149105 | -4.6249 | 7.37E-06 | 4.12E-05 | 3.09825 |
| KIAA0226L | 0.60628 | 2.35367 | 4.62375 | 7.41E-06 | 4.14E-05 | 3.09337 |
| RP11-809C18.3 | -0.7653 | 1.349303 | -4.621 | 7.49E-06 | 4.19E-05 | 3.08198 |
| GLUD1 | -0.5502 | 7.891169 | -4.6195 | 7.54E-06 | 4.21E-05 | 3.07585 |
| ZNF300 | 0.626 | 3.23813 | 4.6169 | 7.63E-06 | 4.26E-05 | 3.06536 |
| IPCEF1 | -0.696 | 2.363002 | -4.6168 | 7.63E-06 | 4.26E-05 | 3.06515 |
| BOK | -1.0178 | 3.831678 | -4.6157 | 7.67E-06 | 4.27E-05 | 3.06034 |
| HBQ1 | -0.6001 | 1.267693 | -4.6146 | 7.70E-06 | 4.29E-05 | 3.05613 |
| PRRT2 | -0.6668 | 4.761767 | -4.6137 | 7.73E-06 | 4.30E-05 | 3.05227 |
| NRP2 | 0.59242 | 4.705887 | 4.61341 | 7.74E-06 | 4.31E-05 | 3.05113 |
| SQRDL | 0.60569 | 3.024989 | 4.61334 | 7.74E-06 | 4.31E-05 | 3.05082 |
| PTGES3P1 | 0.91668 | 2.353829 | 4.61217 | 7.78E-06 | 4.33E-05 | 3.04606 |
| CD300LG | -0.6151 | 0.874762 | -4.612 | 7.79E-06 | 4.33E-05 | 3.04548 |
| RP11-498C9.2 | 0.58961 | 1.80741 | 4.61011 | 7.85E-06 | 4.36E-05 | 3.03763 |
| TCEAL2 | -0.6787 | 6.889992 | -4.6098 | 7.86E-06 | 4.37E-05 | 3.03622 |
| DGCR5 | -0.6842 | 3.617828 | -4.6092 | 7.88E-06 | 4.37E-05 | 3.03399 |
| DNAH11 | 0.53808 | 0.747516 | 4.60896 | 7.89E-06 | 4.38E-05 | 3.03293 |
| CST7 | 0.52914 | 1.204123 | 4.60849 | 7.91E-06 | 4.39E-05 | 3.03101 |
| PITX2 | 0.59995 | 0.518397 | 4.59854 | 8.25E-06 | 4.56E-05 | 2.99045 |
| ATP8A2 | -0.7508 | 1.734843 | -4.5976 | 8.28E-06 | 4.57E-05 | 2.98672 |
| STC2 | 0.8307 | 2.664147 | 4.59753 | 8.28E-06 | 4.57E-05 | 2.98636 |
| MT-ATP8 | -0.5054 | 14.89972 | -4.5971 | 8.30E-06 | 4.58E-05 | 2.98443 |
| FST | 0.56298 | 1.480827 | 4.59704 | 8.30E-06 | 4.58E-05 | 2.98437 |
| A2M | 0.59855 | 7.607925 | 4.5962 | 8.33E-06 | 4.59E-05 | 2.98093 |
| DOCK7 | 0.56338 | 4.229225 | 4.59534 | 8.36E-06 | 4.61E-05 | 2.97744 |
| SIPA1 | 0.50617 | 4.433893 | 4.59081 | 8.52E-06 | 4.69E-05 | 2.95902 |
| FGL2 | 0.66305 | 3.066789 | 4.59055 | 8.53E-06 | 4.69E-05 | 2.95794 |
| FA2H | -1.0394 | 3.919437 | -4.59 | 8.55E-06 | 4.70E-05 | 2.95575 |
| MYO5B | 0.65198 | 1.524487 | 4.58906 | 8.59E-06 | 4.72E-05 | 2.95189 |
| AC009005.2 | 0.51212 | 1.436963 | 4.58779 | 8.64E-06 | 4.74E-05 | 2.94673 |
| SLC16A10 | 0.51068 | 1.135793 | 4.58778 | 8.64E-06 | 4.74E-05 | 2.94669 |
| PLP1 | -1.1044 | 9.90019 | -4.5872 | 8.66E-06 | 4.75E-05 | 2.9443 |
| FYB | 0.69796 | 3.384576 | 4.58639 | 8.69E-06 | 4.76E-05 | 2.94104 |
| ASPDH | -0.7159 | 3.309116 | -4.5789 | 8.97E-06 | 4.90E-05 | 2.91057 |
| SPN | 0.52922 | 1.799119 | 4.57839 | 8.99E-06 | 4.90E-05 | 2.90851 |
| EHD2 | 0.59361 | 3.905155 | 4.57736 | 9.03E-06 | 4.92E-05 | 2.90434 |
| CCDC89 | 0.52887 | 1.331041 | 4.57608 | 9.08E-06 | 4.94E-05 | 2.89916 |
| NET1 | -0.5499 | 4.77341 | -4.5747 | 9.13E-06 | 4.97E-05 | 2.89341 |
| NDUFA4L2 | 0.63644 | 4.47487 | 4.57368 | 9.17E-06 | 4.98E-05 | 2.88942 |
| RP11-81K13.1 | -0.7428 | 1.125033 | -4.5694 | 9.34E-06 | 5.06E-05 | 2.87212 |
| KLHDC9 | -0.5995 | 3.772406 | -4.5674 | 9.42E-06 | 5.10E-05 | 2.86399 |
| PLEKHA6 | -0.5284 | 3.565526 | -4.567 | 9.43E-06 | 5.11E-05 | 2.86253 |
| RP6-65G23.3 | -0.5203 | 1.292269 | -4.5663 | 9.46E-06 | 5.12E-05 | 2.85944 |
| SIGLEC9 | 0.6126 | 2.176232 | 4.56489 | 9.52E-06 | 5.14E-05 | 2.85378 |
| ACTA2 | 0.86596 | 5.42833 | 4.56267 | 9.61E-06 | 5.19E-05 | 2.8448 |
| AC131056.3 | -0.6207 | 1.171665 | -4.5613 | 9.66E-06 | 5.21E-05 | 2.83933 |
| CHRNA4 | -0.7694 | 2.337958 | -4.5592 | 9.75E-06 | 5.25E-05 | 2.83072 |
| CSRP2 | 0.85505 | 6.285965 | 4.55737 | 9.83E-06 | 5.29E-05 | 2.82335 |
| C1orf204 | -0.5305 | 1.633556 | -4.5573 | 9.83E-06 | 5.30E-05 | 2.82286 |
| ABAT | -0.5703 | 7.257852 | -4.5551 | 9.92E-06 | 5.34E-05 | 2.81431 |
| F5 | -1.2786 | 2.335026 | -4.5521 | 1.00E-05 | 5.40E-05 | 2.8021 |
| DBNDD2 | -0.8163 | 7.571953 | -4.5492 | 1.02E-05 | 5.47E-05 | 2.79031 |
| GPR27 | -0.8026 | 3.535549 | -4.5483 | 1.02E-05 | 5.48E-05 | 2.78688 |
| SLC6A6 | 0.52286 | 2.513506 | 4.54783 | 1.02E-05 | 5.49E-05 | 2.78478 |
| SERPINE1 | 1.24009 | 3.172314 | 4.54471 | 1.04E-05 | 5.56E-05 | 2.77219 |
| SYT9 | -0.5203 | 2.204169 | -4.5443 | 1.04E-05 | 5.57E-05 | 2.77044 |
| TFAP2B | 0.58467 | 0.560608 | 4.54218 | 1.05E-05 | 5.61E-05 | 2.76196 |
| PDZD2 | -0.5914 | 3.737645 | -4.541 | 1.05E-05 | 5.63E-05 | 2.75736 |
| SOCS2 | 0.83121 | 3.51951 | 4.53852 | 1.06E-05 | 5.69E-05 | 2.74721 |
| RP11-277L2.5 | -0.6594 | 3.72835 | -4.5372 | 1.07E-05 | 5.71E-05 | 2.74181 |
| ROGDI | -0.5015 | 5.949246 | -4.534 | 1.09E-05 | 5.78E-05 | 2.72885 |
| GATA3 | 0.55306 | 0.714458 | 4.53296 | 1.09E-05 | 5.81E-05 | 2.72479 |
| EFNA1 | 0.51564 | 4.500748 | 4.5324 | 1.09E-05 | 5.82E-05 | 2.72255 |
| AC090425.1 | -0.5924 | 0.772751 | -4.5313 | 1.10E-05 | 5.85E-05 | 2.71795 |
| RP11-547D24.1 | -0.5012 | 0.921454 | -4.5296 | 1.11E-05 | 5.88E-05 | 2.71134 |
| RASAL3 | 0.53767 | 2.406638 | 4.52898 | 1.11E-05 | 5.89E-05 | 2.70879 |
| TUBA4A | -0.9657 | 4.848712 | -4.5287 | 1.11E-05 | 5.90E-05 | 2.70748 |
| HOXB6 | 0.52422 | 0.609928 | 4.52569 | 1.12E-05 | 5.96E-05 | 2.69552 |
| PRKAR1B | -0.7672 | 5.719335 | -4.5254 | 1.12E-05 | 5.97E-05 | 2.69457 |
| CLEC18A | 0.57033 | 1.704336 | 4.52413 | 1.13E-05 | 6.00E-05 | 2.68925 |
| RP4-792G4.2 | 0.85268 | 1.453586 | 4.52227 | 1.14E-05 | 6.04E-05 | 2.68179 |
| PAM | 0.55785 | 6.531683 | 4.52148 | 1.14E-05 | 6.06E-05 | 2.67861 |
| AMER2 | -0.6971 | 5.251438 | -4.521 | 1.15E-05 | 6.07E-05 | 2.67666 |
| NWD1 | -0.5702 | 1.754232 | -4.5208 | 1.15E-05 | 6.07E-05 | 2.67607 |
| SOX2 | 0.55422 | 7.777359 | 4.52063 | 1.15E-05 | 6.07E-05 | 2.67518 |
| LRRC32 | 0.66677 | 2.157841 | 4.51893 | 1.16E-05 | 6.11E-05 | 2.66837 |
| CD34 | 0.55976 | 3.853299 | 4.50955 | 1.20E-05 | 6.34E-05 | 2.63069 |
| GPR141 | 0.51056 | 0.733612 | 4.50942 | 1.20E-05 | 6.34E-05 | 2.63019 |
| GABRB2 | -1.0764 | 2.230171 | -4.5078 | 1.21E-05 | 6.38E-05 | 2.62376 |
| CTD-2545M3.8 | -0.6773 | 1.798973 | -4.5077 | 1.21E-05 | 6.38E-05 | 2.62324 |
| TMEM63C | -0.5808 | 2.921626 | -4.5043 | 1.23E-05 | 6.47E-05 | 2.60977 |
| HOXB2 | 0.76866 | 1.167374 | 4.50432 | 1.23E-05 | 6.47E-05 | 2.60973 |
| AF131216.5 | -0.7902 | 2.864563 | -4.5039 | 1.23E-05 | 6.48E-05 | 2.60796 |
| KIAA1107 | -0.5376 | 2.775132 | -4.4982 | 1.26E-05 | 6.62E-05 | 2.58516 |
| PRKCZ | -0.6846 | 5.06598 | -4.4969 | 1.27E-05 | 6.65E-05 | 2.58012 |
| TMEM155 | -1.0094 | 1.890307 | -4.494 | 1.28E-05 | 6.71E-05 | 2.56839 |
| CFH | 0.62163 | 2.734075 | 4.49388 | 1.28E-05 | 6.71E-05 | 2.56791 |
| RAB3C | -0.9351 | 3.040631 | -4.4929 | 1.29E-05 | 6.74E-05 | 2.56383 |
| KIAA1244 | -0.5641 | 3.194323 | -4.4924 | 1.29E-05 | 6.75E-05 | 2.56182 |
| LINC00672 | -0.737 | 3.545562 | -4.4912 | 1.30E-05 | 6.77E-05 | 2.55725 |
| HLA-DMB | 0.85286 | 4.900311 | 4.49011 | 1.31E-05 | 6.80E-05 | 2.55285 |
| REPS2 | -0.6484 | 3.444009 | -4.4897 | 1.31E-05 | 6.81E-05 | 2.55123 |
| RP11-588K22.2 | -0.6254 | 2.724907 | -4.4886 | 1.31E-05 | 6.84E-05 | 2.54665 |
| BCAT1 | 0.75018 | 2.721286 | 4.48781 | 1.32E-05 | 6.86E-05 | 2.54363 |
| TESPA1 | -1.0401 | 1.770063 | -4.4868 | 1.32E-05 | 6.88E-05 | 2.53979 |
| GALNT10 | 0.5678 | 4.504424 | 4.486 | 1.33E-05 | 6.90E-05 | 2.5364 |
| RP11-689K5.3 | 0.50303 | 0.854145 | 4.48349 | 1.34E-05 | 6.97E-05 | 2.52639 |
| LIMD1 | 0.60929 | 2.67532 | 4.48325 | 1.34E-05 | 6.97E-05 | 2.52541 |
| TMEM125 | -1.0439 | 2.724566 | -4.4828 | 1.35E-05 | 6.98E-05 | 2.52374 |
| SH2D4A | 0.583 | 0.948019 | 4.48186 | 1.35E-05 | 7.01E-05 | 2.51988 |
| GZMB | 0.58047 | 1.159994 | 4.48156 | 1.35E-05 | 7.02E-05 | 2.51867 |
| DIRAS3 | 0.82278 | 2.36391 | 4.48136 | 1.35E-05 | 7.02E-05 | 2.51787 |
| SCRT1 | -1.0145 | 3.044112 | -4.4812 | 1.36E-05 | 7.02E-05 | 2.51724 |
| KLF6 | 0.54113 | 4.737188 | 4.48088 | 1.36E-05 | 7.03E-05 | 2.51597 |
| GDF10 | -0.8349 | 2.247942 | -4.4793 | 1.37E-05 | 7.07E-05 | 2.50954 |
| PRODH | -0.8892 | 4.195155 | -4.4792 | 1.37E-05 | 7.07E-05 | 2.50929 |
| RP11-626G11.3 | 0.57904 | 2.144585 | 4.47551 | 1.39E-05 | 7.17E-05 | 2.49454 |
| NPM2 | -0.836 | 2.911759 | -4.4752 | 1.39E-05 | 7.17E-05 | 2.49333 |
| SAT1 | 0.52945 | 7.881195 | 4.47238 | 1.41E-05 | 7.25E-05 | 2.48205 |
| CAMKK1 | -0.8262 | 3.200883 | -4.4668 | 1.44E-05 | 7.40E-05 | 2.45983 |
| PLIN1 | -0.5785 | 1.609803 | -4.4665 | 1.44E-05 | 7.40E-05 | 2.45852 |
| SFN | 0.54419 | 0.708335 | 4.46642 | 1.44E-05 | 7.40E-05 | 2.45833 |
| GPR3 | 0.59068 | 1.382624 | 4.46629 | 1.44E-05 | 7.41E-05 | 2.4578 |
| TAPSAR1 | 0.59736 | 3.655055 | 4.4655 | 1.45E-05 | 7.43E-05 | 2.45467 |
| CD163L1 | 0.59578 | 1.619622 | 4.46368 | 1.46E-05 | 7.48E-05 | 2.44741 |
| NEDD9 | 0.58318 | 3.586126 | 4.46159 | 1.47E-05 | 7.54E-05 | 2.43909 |
| SOD2 | 0.98639 | 7.499022 | 4.45953 | 1.48E-05 | 7.60E-05 | 2.43092 |
| RP11-524D16__A.3 | 0.69393 | 1.071948 | 4.45896 | 1.49E-05 | 7.62E-05 | 2.42863 |
| HOTAIRM1 | 0.97071 | 1.669742 | 4.45756 | 1.50E-05 | 7.65E-05 | 2.42308 |
| CD300E | 0.59741 | 0.706786 | 4.4575 | 1.50E-05 | 7.65E-05 | 2.42283 |
| SLFN13 | 0.53868 | 1.646376 | 4.45516 | 1.51E-05 | 7.72E-05 | 2.41352 |
| CLECL1 | 0.52127 | 1.123629 | 4.45439 | 1.52E-05 | 7.74E-05 | 2.41047 |
| SYCE1 | -0.7774 | 1.337356 | -4.4544 | 1.52E-05 | 7.74E-05 | 2.41043 |
| HLA-A | 0.79267 | 8.010437 | 4.45324 | 1.52E-05 | 7.77E-05 | 2.40592 |
| ELF3 | 0.51118 | 0.934412 | 4.45307 | 1.52E-05 | 7.77E-05 | 2.40521 |
| RNASE6 | 0.57517 | 3.876074 | 4.45125 | 1.54E-05 | 7.83E-05 | 2.398 |
| MEX3A | 0.74629 | 3.451085 | 4.45066 | 1.54E-05 | 7.84E-05 | 2.39566 |
| SYBU | -0.5735 | 5.702032 | -4.4499 | 1.54E-05 | 7.86E-05 | 2.39266 |
| CLEC18B | 0.81462 | 2.155138 | 4.44931 | 1.55E-05 | 7.87E-05 | 2.3903 |
| KCNT1 | -0.8189 | 1.896248 | -4.4491 | 1.55E-05 | 7.88E-05 | 2.38957 |
| ATP1A3 | -0.9581 | 6.133991 | -4.4477 | 1.56E-05 | 7.92E-05 | 2.38407 |
| CPNE9 | -0.6569 | 1.335734 | -4.4476 | 1.56E-05 | 7.92E-05 | 2.38333 |
| KCNC1 | -0.7018 | 2.954283 | -4.4475 | 1.56E-05 | 7.92E-05 | 2.38295 |
| F2RL1 | 0.6235 | 1.716234 | 4.44534 | 1.57E-05 | 7.98E-05 | 2.37454 |
| RBM11 | -0.6108 | 1.823337 | -4.4449 | 1.58E-05 | 7.99E-05 | 2.37268 |
| CD48 | 0.77684 | 1.899504 | 4.4444 | 1.58E-05 | 8.00E-05 | 2.37082 |
| CHADL | -0.7532 | 4.603796 | -4.4409 | 1.60E-05 | 8.11E-05 | 2.35699 |
| FCER1G | 0.7645 | 6.120331 | 4.43912 | 1.62E-05 | 8.17E-05 | 2.34987 |
| ADAMTS1 | 0.71367 | 2.913483 | 4.43881 | 1.62E-05 | 8.18E-05 | 2.34866 |
| OMG | -0.7368 | 5.922166 | -4.4365 | 1.63E-05 | 8.25E-05 | 2.3394 |
| TNFRSF19 | 0.70892 | 3.449208 | 4.43637 | 1.63E-05 | 8.25E-05 | 2.33898 |
| LDB3 | -0.8207 | 2.470748 | -4.4345 | 1.65E-05 | 8.30E-05 | 2.33146 |
| ZNF474 | 0.56492 | 0.81291 | 4.43433 | 1.65E-05 | 8.31E-05 | 2.3309 |
| HPX | 0.50063 | 1.064175 | 4.43355 | 1.65E-05 | 8.33E-05 | 2.32782 |
| DAB2 | 0.55602 | 4.288703 | 4.43212 | 1.66E-05 | 8.37E-05 | 2.32215 |
| INA | -1.228 | 4.516722 | -4.4321 | 1.66E-05 | 8.37E-05 | 2.32214 |
| MOG | -1.3745 | 5.566965 | -4.4302 | 1.68E-05 | 8.44E-05 | 2.31468 |
| LCK | 0.62031 | 1.1612 | 4.42998 | 1.68E-05 | 8.44E-05 | 2.3137 |
| PCOLCE2 | 0.71647 | 2.197675 | 4.42968 | 1.68E-05 | 8.45E-05 | 2.31253 |
| ARHGAP18 | 0.54634 | 2.575032 | 4.42894 | 1.69E-05 | 8.47E-05 | 2.30958 |
| PAXBP1 | 0.53004 | 4.798282 | 4.4287 | 1.69E-05 | 8.48E-05 | 2.30862 |
| TGFB2 | 0.98716 | 3.529529 | 4.42844 | 1.69E-05 | 8.49E-05 | 2.30761 |
| CPVL | 0.66566 | 4.304619 | 4.42796 | 1.69E-05 | 8.50E-05 | 2.30571 |
| PABPC1L | 0.56649 | 4.190901 | 4.42686 | 1.70E-05 | 8.53E-05 | 2.30136 |
| IL32 | 0.78476 | 4.744623 | 4.42655 | 1.70E-05 | 8.54E-05 | 2.30014 |
| MSMP | 0.77664 | 0.894535 | 4.42574 | 1.71E-05 | 8.56E-05 | 2.29693 |
| NELL1 | -0.8307 | 1.978389 | -4.4242 | 1.72E-05 | 8.62E-05 | 2.29084 |
| KCNK1 | -0.8975 | 3.530182 | -4.424 | 1.72E-05 | 8.62E-05 | 2.29019 |
| PRKAG2-AS1 | -0.5864 | 2.484143 | -4.424 | 1.72E-05 | 8.62E-05 | 2.28989 |
| C10orf10 | 0.73124 | 4.4566 | 4.42295 | 1.73E-05 | 8.65E-05 | 2.28589 |
| POSTN | 1.22128 | 1.225387 | 4.42227 | 1.73E-05 | 8.67E-05 | 2.28321 |
| VAMP8 | 0.64657 | 4.737933 | 4.42128 | 1.74E-05 | 8.70E-05 | 2.27929 |
| PNMT | -0.6411 | 1.540158 | -4.421 | 1.74E-05 | 8.71E-05 | 2.27838 |
| MCF2 | -0.6242 | 2.037385 | -4.4207 | 1.74E-05 | 8.72E-05 | 2.277 |
| GS1-72M22.1 | -0.6166 | 1.667005 | -4.4195 | 1.75E-05 | 8.76E-05 | 2.27221 |
| KIAA1161 | -0.5823 | 4.18518 | -4.4172 | 1.77E-05 | 8.83E-05 | 2.26304 |
| DUSP6 | 0.67898 | 4.674329 | 4.41567 | 1.78E-05 | 8.88E-05 | 2.25717 |
| SYNGR3 | -0.9981 | 3.306706 | -4.4154 | 1.78E-05 | 8.88E-05 | 2.25613 |
| CASQ1 | -0.6688 | 3.765485 | -4.4153 | 1.78E-05 | 8.89E-05 | 2.25557 |
| GLIPR2 | 0.51949 | 5.536442 | 4.41523 | 1.78E-05 | 8.89E-05 | 2.25544 |
| KCNC2 | -1.0233 | 2.198105 | -4.4151 | 1.78E-05 | 8.89E-05 | 2.25506 |
| ASPHD1 | -0.7126 | 4.723937 | -4.415 | 1.79E-05 | 8.89E-05 | 2.25458 |
| CHI3L1 | 1.97865 | 5.307812 | 4.41341 | 1.80E-05 | 8.94E-05 | 2.24826 |
| HPCA | -1.2942 | 4.159223 | -4.4119 | 1.81E-05 | 8.99E-05 | 2.24234 |
| PDIA2 | -0.7196 | 3.44451 | -4.4101 | 1.82E-05 | 9.05E-05 | 2.2353 |
| SLC11A1 | 0.94603 | 3.463424 | 4.40886 | 1.83E-05 | 9.08E-05 | 2.23029 |
| AASS | 0.50496 | 4.170879 | 4.40762 | 1.84E-05 | 9.13E-05 | 2.22541 |
| GBP3 | 0.82087 | 3.69046 | 4.40667 | 1.85E-05 | 9.16E-05 | 2.22168 |
| SLC6A7 | -0.8342 | 1.233328 | -4.4062 | 1.85E-05 | 9.17E-05 | 2.21988 |
| RNF135 | 0.57222 | 2.763269 | 4.40617 | 1.85E-05 | 9.17E-05 | 2.21969 |
| CXCL10 | 1.06377 | 1.828334 | 4.40543 | 1.86E-05 | 9.20E-05 | 2.21679 |
| SERPINA3 | 1.60236 | 6.559445 | 4.4037 | 1.87E-05 | 9.25E-05 | 2.20998 |
| MAP7D2 | -1.0403 | 2.162864 | -4.4034 | 1.87E-05 | 9.26E-05 | 2.20888 |
| HCN2 | -0.6601 | 4.599066 | -4.4034 | 1.87E-05 | 9.26E-05 | 2.20866 |
| FCN3 | 0.50571 | 0.640577 | 4.39963 | 1.90E-05 | 9.39E-05 | 2.19397 |
| CAMK4 | -0.779 | 2.450631 | -4.3989 | 1.91E-05 | 9.41E-05 | 2.19118 |
| LMCD1 | 0.53995 | 3.555863 | 4.39839 | 1.91E-05 | 9.43E-05 | 2.1891 |
| MAG | -1.3685 | 5.367379 | -4.3981 | 1.92E-05 | 9.44E-05 | 2.18778 |
| TMSB10 | 0.58169 | 9.839771 | 4.39798 | 1.92E-05 | 9.44E-05 | 2.18746 |
| APOBEC3G | 0.58129 | 2.419589 | 4.39535 | 1.94E-05 | 9.53E-05 | 2.17712 |
| CLCN4 | -0.5489 | 3.603142 | -4.3939 | 1.95E-05 | 9.58E-05 | 2.17142 |
| SORCS2 | -0.5424 | 2.748221 | -4.3909 | 1.97E-05 | 9.68E-05 | 2.15971 |
| CREG2 | -1.1215 | 2.524261 | -4.389 | 1.99E-05 | 9.74E-05 | 2.15223 |
| LYN | 0.56037 | 3.831992 | 4.38804 | 2.00E-05 | 9.78E-05 | 2.14842 |
| CLEC10A | 0.51402 | 0.880191 | 4.38782 | 2.00E-05 | 9.78E-05 | 2.14753 |
| SLC1A5 | 0.63704 | 3.073177 | 4.38753 | 2.00E-05 | 9.79E-05 | 2.14639 |
| ISYNA1 | 0.50752 | 4.192845 | 4.38426 | 2.03E-05 | 9.91E-05 | 2.13358 |
| RP11-862L9.3 | -1.5301 | 3.842493 | -4.3838 | 2.03E-05 | 9.92E-05 | 2.13196 |
| RP11-454P7.3 | -0.632 | 1.094364 | -4.3819 | 2.05E-05 | 1.00E-04 | 2.12416 |
| LYZ | 1.07105 | 3.480157 | 4.3812 | 2.05E-05 | 0.0001 | 2.12157 |
| LINC00617 | -0.7208 | 1.500236 | -4.3779 | 2.08E-05 | 0.000101 | 2.10849 |
| BST2 | 0.70252 | 5.559471 | 4.37766 | 2.08E-05 | 0.000102 | 2.10769 |
| ADCY5 | -0.5062 | 4.038901 | -4.3755 | 2.10E-05 | 0.000102 | 2.09934 |
| PDE1B | -0.6804 | 2.922425 | -4.3747 | 2.11E-05 | 0.000103 | 2.09624 |
| LAMA2 | 0.54741 | 2.135215 | 4.3742 | 2.11E-05 | 0.000103 | 2.09413 |
| LIPG | 0.6282 | 2.154126 | 4.37256 | 2.13E-05 | 0.000103 | 2.08771 |
| RN7SL731P | 0.79529 | 2.539051 | 4.37255 | 2.13E-05 | 0.000103 | 2.08767 |
| UPP1 | 0.64826 | 3.990889 | 4.36895 | 2.16E-05 | 0.000105 | 2.07358 |
| TBX15 | 0.54674 | 1.709751 | 4.36757 | 2.17E-05 | 0.000105 | 2.06819 |
| SERTAD1 | 0.50846 | 2.718616 | 4.36644 | 2.18E-05 | 0.000106 | 2.06378 |
| SPC25 | 0.707 | 2.598893 | 4.36598 | 2.19E-05 | 0.000106 | 2.06197 |
| RNASE4 | 0.61166 | 2.520903 | 4.36529 | 2.19E-05 | 0.000106 | 2.05929 |
| UBD | 0.9534 | 2.204294 | 4.36457 | 2.20E-05 | 0.000106 | 2.05645 |
| SPSB4 | 0.67467 | 2.936597 | 4.36422 | 2.20E-05 | 0.000106 | 2.05511 |
| CDH6 | 0.54196 | 2.615104 | 4.36411 | 2.20E-05 | 0.000106 | 2.05464 |
| RNF219-AS1 | -0.8433 | 2.981693 | -4.3634 | 2.21E-05 | 0.000107 | 2.05192 |
| CTSB | 0.50184 | 8.471338 | 4.36318 | 2.21E-05 | 0.000107 | 2.05104 |
| LINC00943 | -0.5565 | 1.219722 | -4.3627 | 2.22E-05 | 0.000107 | 2.04911 |
| GALR1 | 0.72902 | 1.644414 | 4.36243 | 2.22E-05 | 0.000107 | 2.0481 |
| TFEC | 0.52053 | 1.827749 | 4.36187 | 2.22E-05 | 0.000107 | 2.04589 |
| GABRB3 | -0.9168 | 4.105321 | -4.3617 | 2.23E-05 | 0.000107 | 2.04541 |
| EPHA10 | -0.5772 | 1.70694 | -4.3616 | 2.23E-05 | 0.000107 | 2.04494 |
| C1orf115 | -0.8747 | 3.077725 | -4.3615 | 2.23E-05 | 0.000107 | 2.04458 |
| LY86-AS1 | -0.6453 | 0.844604 | -4.3614 | 2.23E-05 | 0.000107 | 2.04403 |
| RP11-731J8.2 | -0.7185 | 2.859043 | -4.3609 | 2.23E-05 | 0.000107 | 2.04226 |
| JPH4 | -0.7668 | 5.172301 | -4.3598 | 2.24E-05 | 0.000108 | 2.03779 |
| MYD88 | 0.58313 | 3.597574 | 4.35978 | 2.24E-05 | 0.000108 | 2.03773 |
| TMSB15B | 0.51343 | 3.060279 | 4.3547 | 2.29E-05 | 0.00011 | 2.01789 |
| CACNA1B | -0.7146 | 1.585352 | -4.3545 | 2.29E-05 | 0.00011 | 2.01722 |
| RNF144A-AS1 | -0.5532 | 1.05225 | -4.3537 | 2.30E-05 | 0.00011 | 2.01398 |
| RERGL | -0.6075 | 2.653216 | -4.3535 | 2.30E-05 | 0.00011 | 2.01334 |
| ANK3 | -0.6817 | 3.911087 | -4.3532 | 2.30E-05 | 0.00011 | 2.01216 |
| NCKAP1L | 0.61064 | 3.449615 | 4.35313 | 2.31E-05 | 0.00011 | 2.01177 |
| RP11-783K16.5 | 0.62673 | 1.361341 | 4.35198 | 2.32E-05 | 0.000111 | 2.00731 |
| KLK6 | -1.2901 | 4.267262 | -4.3507 | 2.33E-05 | 0.000111 | 2.00211 |
| EPSTI1 | 0.63938 | 2.483793 | 4.35025 | 2.33E-05 | 0.000112 | 2.00056 |
| IL1RN | 0.73561 | 1.233933 | 4.3481 | 2.35E-05 | 0.000112 | 1.99218 |
| ESPNL | 0.61582 | 0.657931 | 4.34777 | 2.36E-05 | 0.000113 | 1.99087 |
| WARS | 0.52504 | 6.124123 | 4.34598 | 2.37E-05 | 0.000113 | 1.98392 |
| MT3 | -0.8062 | 9.377603 | -4.3453 | 2.38E-05 | 0.000114 | 1.98128 |
| NEURL | -0.8791 | 2.603706 | -4.3449 | 2.38E-05 | 0.000114 | 1.97969 |
| B3GNT5 | 0.59661 | 2.483664 | 4.34459 | 2.39E-05 | 0.000114 | 1.97849 |
| TPSB2 | 0.59535 | 0.65608 | 4.34443 | 2.39E-05 | 0.000114 | 1.97787 |
| TMEM191A | -0.6053 | 1.57914 | -4.3435 | 2.40E-05 | 0.000114 | 1.97431 |
| AC011242.6 | 0.54001 | 1.567832 | 4.34125 | 2.42E-05 | 0.000115 | 1.96547 |
| MIR7-3HG | -0.8365 | 2.109871 | -4.3389 | 2.44E-05 | 0.000116 | 1.95635 |
| SPRED3 | 0.52352 | 1.561797 | 4.33623 | 2.47E-05 | 0.000117 | 1.94596 |
| HCLS1 | 0.6254 | 4.839229 | 4.33387 | 2.49E-05 | 0.000118 | 1.93677 |
| CRYM | -1.3138 | 3.161502 | -4.3315 | 2.52E-05 | 0.000119 | 1.92759 |
| FXYD1 | -1.1601 | 4.895684 | -4.3308 | 2.53E-05 | 0.000119 | 1.92502 |
| GALNT3 | 0.51613 | 1.178351 | 4.3305 | 2.53E-05 | 0.000119 | 1.92367 |
| DMTN | -0.9485 | 4.944699 | -4.3299 | 2.54E-05 | 0.00012 | 1.9215 |
| NMNAT2 | -0.7845 | 4.267414 | -4.3295 | 2.54E-05 | 0.00012 | 1.91976 |
| PHACTR3 | -0.6784 | 5.291955 | -4.3283 | 2.55E-05 | 0.00012 | 1.9153 |
| CYR61 | 1.03156 | 4.715581 | 4.32803 | 2.55E-05 | 0.000121 | 1.9141 |
| CCL5 | 0.77971 | 2.146528 | 4.32621 | 2.57E-05 | 0.000121 | 1.907 |
| SRCIN1 | -0.7392 | 4.199002 | -4.325 | 2.59E-05 | 0.000122 | 1.90242 |
| CXCL11 | 0.56702 | 1.044834 | 4.32182 | 2.62E-05 | 0.000123 | 1.88997 |
| JUN | 0.65774 | 6.508865 | 4.31897 | 2.65E-05 | 0.000124 | 1.87895 |
| KRTAP5-AS1 | -0.5803 | 1.252079 | -4.3186 | 2.66E-05 | 0.000124 | 1.87734 |
| FBXO27 | -0.6254 | 2.575564 | -4.3173 | 2.67E-05 | 0.000125 | 1.87265 |
| WNT10B | -0.7104 | 1.482234 | -4.315 | 2.69E-05 | 0.000126 | 1.86369 |
| KCNJ3 | -0.7292 | 2.022336 | -4.3133 | 2.71E-05 | 0.000127 | 1.85704 |
| CAMK1G | -0.9397 | 1.993119 | -4.3114 | 2.73E-05 | 0.000128 | 1.84977 |
| PRKCG | -1.0135 | 2.145461 | -4.3114 | 2.73E-05 | 0.000128 | 1.84965 |
| VSTM2B | -0.7525 | 3.822238 | -4.3108 | 2.74E-05 | 0.000128 | 1.84715 |
| CCDC85A | -0.6234 | 2.364973 | -4.31 | 2.75E-05 | 0.000128 | 1.84416 |
| MTND2P28 | -0.5607 | 7.926592 | -4.3091 | 2.76E-05 | 0.000129 | 1.84078 |
| LCP1 | 0.58381 | 4.006412 | 4.30814 | 2.77E-05 | 0.000129 | 1.837 |
| GLI1 | 0.6985 | 1.162113 | 4.30094 | 2.85E-05 | 0.000133 | 1.80916 |
| LOXL1 | 0.81193 | 2.100873 | 4.30056 | 2.86E-05 | 0.000133 | 1.8077 |
| GSX2 | 0.6863 | 0.925639 | 4.30023 | 2.86E-05 | 0.000133 | 1.80641 |
| CTD-2023N9.3 | -0.6427 | 0.866623 | -4.2982 | 2.89E-05 | 0.000134 | 1.79854 |
| TMEM144 | -1.1277 | 4.885128 | -4.2977 | 2.89E-05 | 0.000134 | 1.7966 |
| LGI1 | -0.8165 | 3.18231 | -4.2976 | 2.89E-05 | 0.000134 | 1.79625 |
| TAC4 | 0.54813 | 1.021398 | 4.29756 | 2.89E-05 | 0.000134 | 1.7961 |
| PCDH9 | -0.6236 | 4.276447 | -4.2968 | 2.90E-05 | 0.000135 | 1.79299 |
| DNALI1 | 0.61261 | 4.62946 | 4.29647 | 2.91E-05 | 0.000135 | 1.79188 |
| CRHR1 | -0.5157 | 1.82546 | -4.2937 | 2.94E-05 | 0.000136 | 1.78134 |
| CD14 | 0.95579 | 5.435732 | 4.29372 | 2.94E-05 | 0.000136 | 1.78129 |
| SCNN1B | 0.55107 | 0.684346 | 4.293 | 2.95E-05 | 0.000136 | 1.77852 |
| FAHD2B | -0.6866 | 3.718323 | -4.292 | 2.96E-05 | 0.000137 | 1.77476 |
| CYBA | 0.70657 | 5.863253 | 4.29191 | 2.96E-05 | 0.000137 | 1.7743 |
| ACTL6B | -1.0054 | 3.938454 | -4.2903 | 2.98E-05 | 0.000138 | 1.76808 |
| WBSCR17 | -0.9805 | 2.950639 | -4.2901 | 2.98E-05 | 0.000138 | 1.76743 |
| TNFRSF10B | 0.52624 | 4.079658 | 4.2878 | 3.01E-05 | 0.000139 | 1.75843 |
| NT5C1A | -0.5274 | 1.478437 | -4.2847 | 3.05E-05 | 0.00014 | 1.74642 |
| ICAM1 | 0.84718 | 2.996396 | 4.28334 | 3.07E-05 | 0.000141 | 1.74128 |
| VWF | 0.59128 | 3.79948 | 4.28194 | 3.08E-05 | 0.000142 | 1.73587 |
| RP11-286B14.1 | -0.8702 | 1.248453 | -4.2799 | 3.11E-05 | 0.000143 | 1.72787 |
| CYTL1 | 0.59514 | 3.062294 | 4.27916 | 3.12E-05 | 0.000143 | 1.72517 |
| ST18 | -0.95 | 2.996324 | -4.2784 | 3.13E-05 | 0.000144 | 1.72234 |
| MLIP | 0.73337 | 2.281106 | 4.27758 | 3.14E-05 | 0.000144 | 1.71909 |
| IL6 | 0.76427 | 1.302963 | 4.27688 | 3.15E-05 | 0.000144 | 1.71642 |
| C11orf96 | 0.6809 | 4.924818 | 4.26969 | 3.24E-05 | 0.000148 | 1.68875 |
| SCG5 | -0.869 | 6.016034 | -4.2689 | 3.25E-05 | 0.000149 | 1.68579 |
| PIK3AP1 | 0.54654 | 2.893288 | 4.26871 | 3.25E-05 | 0.000149 | 1.68498 |
| CRABP2 | 0.53172 | 2.598895 | 4.26339 | 3.32E-05 | 0.000151 | 1.6646 |
| FNBP1L | 0.61872 | 3.939601 | 4.26123 | 3.35E-05 | 0.000153 | 1.6563 |
| LILRB1 | 0.61148 | 2.581569 | 4.26106 | 3.35E-05 | 0.000153 | 1.65565 |
| CCDC64 | -0.7331 | 2.582201 | -4.2594 | 3.38E-05 | 0.000154 | 1.64932 |
| ODF3B | 0.68825 | 3.015488 | 4.25938 | 3.38E-05 | 0.000154 | 1.64922 |
| TTPA | -0.562 | 1.596612 | -4.257 | 3.41E-05 | 0.000155 | 1.6399 |
| KCNIP3 | -0.765 | 5.112217 | -4.2568 | 3.41E-05 | 0.000155 | 1.63914 |
| PSD2 | -0.698 | 5.513765 | -4.2557 | 3.43E-05 | 0.000155 | 1.63524 |
| PVT1 | 0.70592 | 1.939161 | 4.25421 | 3.45E-05 | 0.000156 | 1.62937 |
| APOL2 | 0.53188 | 5.234323 | 4.25369 | 3.46E-05 | 0.000157 | 1.62741 |
| CNKSR2 | -0.6282 | 3.293044 | -4.251 | 3.49E-05 | 0.000158 | 1.61704 |
| IL17D | -0.5961 | 6.708416 | -4.2507 | 3.50E-05 | 0.000158 | 1.61591 |
| FCRLA | 0.51722 | 1.193739 | 4.24811 | 3.53E-05 | 0.00016 | 1.60605 |
| SEL1L3 | 0.74888 | 2.815014 | 4.24743 | 3.54E-05 | 0.00016 | 1.60342 |
| AR | 0.53894 | 1.771113 | 4.24667 | 3.56E-05 | 0.00016 | 1.60054 |
| ACSBG1 | -0.6939 | 5.204777 | -4.2463 | 3.56E-05 | 0.00016 | 1.59909 |
| HSPB8 | -0.8466 | 6.791052 | -4.2461 | 3.56E-05 | 0.000161 | 1.59823 |
| TMCC2 | -0.5126 | 4.301702 | -4.2456 | 3.57E-05 | 0.000161 | 1.59634 |
| MAP3K5 | -0.5111 | 4.701894 | -4.2422 | 3.62E-05 | 0.000163 | 1.58333 |
| SLC26A10 | 0.68357 | 3.471924 | 4.24198 | 3.62E-05 | 0.000163 | 1.58262 |
| GLS2 | -0.5925 | 2.149428 | -4.2408 | 3.64E-05 | 0.000164 | 1.57806 |
| ELOVL2-AS1 | -0.5153 | 1.2768 | -4.2402 | 3.65E-05 | 0.000164 | 1.57574 |
| MSC | 0.54417 | 1.559463 | 4.24013 | 3.65E-05 | 0.000164 | 1.57554 |
| RP11-268P4.5 | -0.5752 | 1.512394 | -4.2382 | 3.68E-05 | 0.000165 | 1.56829 |
| RAP1GAP | -0.6422 | 4.874767 | -4.234 | 3.74E-05 | 0.000168 | 1.5522 |
| RP11-353N4.1 | -0.5399 | 1.47022 | -4.2281 | 3.83E-05 | 0.000171 | 1.52957 |
| F2RL2 | 0.54791 | 0.791645 | 4.22543 | 3.87E-05 | 0.000173 | 1.51946 |
| CNTN2 | -1.0811 | 4.426218 | -4.2248 | 3.88E-05 | 0.000173 | 1.51719 |
| CUX2 | -0.7512 | 2.016993 | -4.2242 | 3.89E-05 | 0.000173 | 1.51495 |
| PRMT8 | -0.7409 | 1.791631 | -4.2232 | 3.91E-05 | 0.000174 | 1.51084 |
| TRHDE | -0.6025 | 1.084107 | -4.2226 | 3.92E-05 | 0.000174 | 1.50884 |
| ADAP2 | 0.54348 | 3.641764 | 4.22248 | 3.92E-05 | 0.000174 | 1.50823 |
| ALOX5 | 0.62066 | 3.480762 | 4.2207 | 3.95E-05 | 0.000176 | 1.50145 |
| BTNL9 | -0.5586 | 2.742428 | -4.2203 | 3.95E-05 | 0.000176 | 1.49988 |
| ABTB2 | -0.5532 | 2.88551 | -4.2196 | 3.96E-05 | 0.000176 | 1.49722 |
| PNCK | -0.6395 | 4.110812 | -4.2155 | 4.03E-05 | 0.000179 | 1.4817 |
| AMICA1 | 0.5655 | 1.497272 | 4.21226 | 4.08E-05 | 0.000181 | 1.46935 |
| FAM46B | 0.57891 | 0.997084 | 4.21224 | 4.08E-05 | 0.000181 | 1.4693 |
| GPC6 | 0.50724 | 2.059582 | 4.21159 | 4.09E-05 | 0.000181 | 1.4668 |
| RP11-490M8.1 | -0.628 | 3.034937 | -4.2105 | 4.11E-05 | 0.000182 | 1.46268 |
| UNC79 | -0.6546 | 2.770898 | -4.2082 | 4.15E-05 | 0.000183 | 1.45402 |
| SHISA7 | -0.6819 | 3.103435 | -4.2082 | 4.15E-05 | 0.000183 | 1.45377 |
| NRXN3 | -0.6955 | 3.209051 | -4.2045 | 4.21E-05 | 0.000186 | 1.44006 |
| AC013268.2 | -0.6182 | 0.909996 | -4.2032 | 4.23E-05 | 0.000187 | 1.43498 |
| IL34 | -0.5519 | 2.72054 | -4.2014 | 4.26E-05 | 0.000188 | 1.42818 |
| LMO1 | 0.69881 | 2.274574 | 4.20134 | 4.27E-05 | 0.000188 | 1.42791 |
| NKX6-2 | -1.2146 | 4.075373 | -4.2004 | 4.28E-05 | 0.000188 | 1.42431 |
| AMZ1 | -0.6741 | 2.459102 | -4.1975 | 4.33E-05 | 0.00019 | 1.41331 |
| RP11-379F4.4 | -0.5032 | 1.223067 | -4.1949 | 4.38E-05 | 0.000192 | 1.40333 |
| GAD2 | -0.9054 | 1.86024 | -4.1938 | 4.40E-05 | 0.000193 | 1.39925 |
| NPTX1 | -1.1162 | 3.34858 | -4.1919 | 4.43E-05 | 0.000194 | 1.39229 |
| TAGLN | 0.83047 | 5.900361 | 4.18953 | 4.47E-05 | 0.000196 | 1.38319 |
| ENPP2 | -1.0306 | 5.042571 | -4.1858 | 4.54E-05 | 0.000198 | 1.36892 |
| KIAA1549L | -0.5051 | 3.234193 | -4.1845 | 4.56E-05 | 0.000199 | 1.36407 |
| HLA-F | 0.61395 | 5.098506 | 4.18263 | 4.60E-05 | 0.0002 | 1.3571 |
| THBS1 | 0.85011 | 2.494998 | 4.18174 | 4.61E-05 | 0.000201 | 1.35377 |
| TXLNB | 0.59232 | 1.281801 | 4.18158 | 4.62E-05 | 0.000201 | 1.35314 |
| FSCN1 | 0.50714 | 6.67349 | 4.18125 | 4.62E-05 | 0.000201 | 1.35191 |
| IGHG1 | 1.56918 | 2.473354 | 4.18024 | 4.64E-05 | 0.000202 | 1.3481 |
| RP11-527D7.1 | -0.5395 | 1.166934 | -4.179 | 4.66E-05 | 0.000203 | 1.34353 |
| SULF1 | 0.95621 | 3.810962 | 4.17843 | 4.67E-05 | 0.000203 | 1.34123 |
| S100A6 | 0.74518 | 8.183906 | 4.17769 | 4.69E-05 | 0.000204 | 1.33845 |
| FZD6 | 0.50075 | 1.909375 | 4.17697 | 4.70E-05 | 0.000204 | 1.33573 |
| TMEM88B | -1.1445 | 3.264412 | -4.1759 | 4.72E-05 | 0.000205 | 1.33169 |
| C11orf87 | -0.7816 | 1.449251 | -4.1717 | 4.80E-05 | 0.000208 | 1.31589 |
| RCAN2 | -0.7794 | 4.396135 | -4.1705 | 4.83E-05 | 0.000209 | 1.31117 |
| CD180 | 0.55265 | 1.852167 | 4.16958 | 4.84E-05 | 0.00021 | 1.30786 |
| NPTXR | -0.7239 | 5.026371 | -4.1693 | 4.85E-05 | 0.00021 | 1.30664 |
| CXXC11 | -0.8526 | 3.067799 | -4.1679 | 4.87E-05 | 0.000211 | 1.30167 |
| HS3ST3B1 | 0.53605 | 0.775234 | 4.16727 | 4.89E-05 | 0.000211 | 1.29918 |
| TPSAB1 | 0.62041 | 0.712927 | 4.16699 | 4.89E-05 | 0.000211 | 1.29813 |
| DLK1 | 0.61132 | 0.631508 | 4.16528 | 4.93E-05 | 0.000212 | 1.29166 |
| CRHBP | -0.6203 | 2.256487 | -4.1649 | 4.93E-05 | 0.000213 | 1.29024 |
| HOXA-AS2 | 0.65552 | 0.531835 | 4.16411 | 4.95E-05 | 0.000213 | 1.28728 |
| ADAM8 | 0.50459 | 2.122819 | 4.16332 | 4.96E-05 | 0.000214 | 1.28431 |
| PTPRC | 0.62802 | 3.353006 | 4.16196 | 4.99E-05 | 0.000215 | 1.27918 |
| FAM19A1 | -0.7167 | 1.728417 | -4.1619 | 4.99E-05 | 0.000215 | 1.27879 |
| HCP5 | 0.60329 | 2.555082 | 4.15992 | 5.03E-05 | 0.000216 | 1.27151 |
| PCDH7 | -0.6724 | 3.832745 | -4.159 | 5.05E-05 | 0.000217 | 1.26788 |
| RP11-392E22.12 | -0.702 | 1.473113 | -4.1588 | 5.05E-05 | 0.000217 | 1.26747 |
| AC008964.1 | 0.64437 | 2.809346 | 4.15876 | 5.06E-05 | 0.000217 | 1.26715 |
| COL27A1 | 0.59594 | 2.384049 | 4.15651 | 5.10E-05 | 0.000219 | 1.2587 |
| FAIM3 | 0.55294 | 1.812244 | 4.15634 | 5.10E-05 | 0.000219 | 1.25805 |
| HBEGF | 0.57521 | 3.517534 | 4.15478 | 5.14E-05 | 0.00022 | 1.25219 |
| TRAF4 | 0.55282 | 5.40926 | 4.15344 | 5.16E-05 | 0.000221 | 1.24716 |
| SASH3 | 0.61028 | 3.149979 | 4.14548 | 5.33E-05 | 0.000227 | 1.21726 |
| RBM12B-AS1 | 0.51935 | 1.509335 | 4.14477 | 5.34E-05 | 0.000227 | 1.21461 |
| EPHB3 | 0.52728 | 3.155286 | 4.14443 | 5.35E-05 | 0.000228 | 1.21335 |
| SNAP25-AS1 | -0.5007 | 1.775992 | -4.1436 | 5.37E-05 | 0.000228 | 1.21032 |
| AC005789.11 | 0.50435 | 1.701002 | 4.14245 | 5.39E-05 | 0.000229 | 1.20591 |
| TSPAN31 | 0.53553 | 5.899246 | 4.14146 | 5.41E-05 | 0.00023 | 1.20222 |
| PLEKHH1 | -0.7007 | 5.287143 | -4.1389 | 5.47E-05 | 0.000232 | 1.19269 |
| SYNC | 0.59504 | 2.886758 | 4.1375 | 5.50E-05 | 0.000233 | 1.18737 |
| HS3ST2 | -0.805 | 2.30223 | -4.1373 | 5.50E-05 | 0.000233 | 1.18657 |
| CAPN3 | -0.9104 | 5.314107 | -4.1366 | 5.52E-05 | 0.000234 | 1.18417 |
| CD4 | 0.58325 | 4.50888 | 4.13627 | 5.53E-05 | 0.000234 | 1.18278 |
| ARSJ | 0.58269 | 1.564963 | 4.13437 | 5.57E-05 | 0.000235 | 1.17565 |
| PART1 | -0.6352 | 1.387069 | -4.133 | 5.60E-05 | 0.000236 | 1.17059 |
| SIM2 | 0.6132 | 1.967575 | 4.13004 | 5.66E-05 | 0.000239 | 1.15947 |
| RASGEF1C | -0.5442 | 3.329317 | -4.1265 | 5.74E-05 | 0.000242 | 1.1464 |
| NCF1 | 0.57144 | 2.418339 | 4.12254 | 5.83E-05 | 0.000245 | 1.13144 |
| SFRP2 | -1.5069 | 4.982293 | -4.1195 | 5.91E-05 | 0.000248 | 1.12002 |
| RP11-192H23.5 | -1.0353 | 3.063614 | -4.1193 | 5.91E-05 | 0.000248 | 1.11951 |
| RASGRP1 | -0.5408 | 2.85349 | -4.1171 | 5.96E-05 | 0.00025 | 1.11121 |
| TRIB2 | 0.56383 | 5.428379 | 4.11567 | 6.00E-05 | 0.000251 | 1.10581 |
| VAV3 | 0.52393 | 1.355552 | 4.11562 | 6.00E-05 | 0.000251 | 1.10563 |
| SLC4A4 | -0.6975 | 5.59825 | -4.1142 | 6.03E-05 | 0.000252 | 1.10019 |
| APLN | 0.72004 | 4.94398 | 4.11396 | 6.04E-05 | 0.000253 | 1.09943 |
| CD68 | 0.74292 | 6.266004 | 4.11341 | 6.05E-05 | 0.000253 | 1.09737 |
| TNFAIP8L2 | 0.50974 | 2.858595 | 4.10962 | 6.14E-05 | 0.000256 | 1.08325 |
| RP11-347E10.1 | 0.59165 | 0.937369 | 4.10857 | 6.17E-05 | 0.000257 | 1.07934 |
| OAS3 | 0.57499 | 3.455839 | 4.10687 | 6.21E-05 | 0.000259 | 1.07304 |
| C5AR1 | 0.69066 | 2.4749 | 4.10431 | 6.27E-05 | 0.000261 | 1.0635 |
| LCP2 | 0.57157 | 4.13471 | 4.10283 | 6.31E-05 | 0.000262 | 1.05801 |
| TNNT1 | -0.8591 | 2.530154 | -4.0989 | 6.40E-05 | 0.000266 | 1.04339 |
| LAIR1 | 0.67368 | 4.296208 | 4.09883 | 6.41E-05 | 0.000266 | 1.04312 |
| MMD2 | -0.8813 | 4.002273 | -4.0987 | 6.41E-05 | 0.000266 | 1.0426 |
| C1S | 0.78261 | 5.263999 | 4.09828 | 6.42E-05 | 0.000266 | 1.04108 |
| LINC00599 | -0.8053 | 3.387069 | -4.0978 | 6.43E-05 | 0.000267 | 1.03926 |
| CAMKV | -0.9998 | 3.880596 | -4.0964 | 6.47E-05 | 0.000268 | 1.03413 |
| ABCA2 | -0.595 | 6.84269 | -4.0964 | 6.47E-05 | 0.000268 | 1.03408 |
| RTN4RL1 | -0.6791 | 1.407737 | -4.0934 | 6.54E-05 | 0.000271 | 1.02293 |
| CCND2 | 0.54594 | 5.447978 | 4.09308 | 6.55E-05 | 0.000271 | 1.02176 |
| RANBP3L | -0.5701 | 4.095883 | -4.0927 | 6.56E-05 | 0.000272 | 1.02027 |
| SLC30A3 | -0.9452 | 1.899984 | -4.0926 | 6.56E-05 | 0.000272 | 1.02007 |
| ZDHHC11B | -0.5453 | 3.727143 | -4.0914 | 6.60E-05 | 0.000273 | 1.0156 |
| TSPO | 0.55221 | 5.5704 | 4.09073 | 6.61E-05 | 0.000273 | 1.01304 |
| OR2I1P | 0.56778 | 0.736678 | 4.08757 | 6.70E-05 | 0.000276 | 1.00135 |
| PTPRN2 | -0.5178 | 5.055728 | -4.0816 | 6.86E-05 | 0.000282 | 0.9791 |
| SLC4A10 | -0.8416 | 2.793361 | -4.0807 | 6.88E-05 | 0.000283 | 0.97592 |
| RP11-701H24.3 | -0.5428 | 2.267366 | -4.0793 | 6.92E-05 | 0.000285 | 0.97084 |
| IGHA1 | 1.1539 | 1.712934 | 4.07887 | 6.93E-05 | 0.000285 | 0.96912 |
| RP11-307B6.3 | -0.7249 | 1.76796 | -4.078 | 6.95E-05 | 0.000286 | 0.96606 |
| S100A2 | 0.59361 | 2.094469 | 4.07788 | 6.96E-05 | 0.000286 | 0.96544 |
| SLN | 1.27413 | 2.301326 | 4.07655 | 6.99E-05 | 0.000287 | 0.9605 |
| VIP | -0.6895 | 1.238898 | -4.074 | 7.06E-05 | 0.000289 | 0.95123 |
| BCYRN1 | -0.7571 | 1.650131 | -4.0732 | 7.09E-05 | 0.00029 | 0.94795 |
| SEMA4D | -0.5553 | 5.160837 | -4.0725 | 7.10E-05 | 0.000291 | 0.94558 |
| SLC1A3 | -0.5312 | 8.583053 | -4.0723 | 7.11E-05 | 0.000291 | 0.94485 |
| MIR219-2 | -0.8074 | 3.720458 | -4.0687 | 7.21E-05 | 0.000295 | 0.93146 |
| CTC-338M12.5 | -0.5093 | 2.901754 | -4.0669 | 7.26E-05 | 0.000297 | 0.92478 |
| NAV3 | -0.5064 | 3.015321 | -4.0645 | 7.33E-05 | 0.000299 | 0.91587 |
| KCNJ16 | -0.8911 | 3.906989 | -4.0626 | 7.38E-05 | 0.000301 | 0.90881 |
| GSDMD | 0.52355 | 3.6623 | 4.0616 | 7.41E-05 | 0.000302 | 0.9053 |
| FAM187A | 0.72805 | 2.800594 | 4.06145 | 7.42E-05 | 0.000302 | 0.90474 |
| RAB40B | -0.5153 | 4.982191 | -4.0614 | 7.42E-05 | 0.000302 | 0.90439 |
| HAMP | 0.9816 | 2.766314 | 4.05726 | 7.54E-05 | 0.000307 | 0.88929 |
| KIAA0319 | -0.5807 | 1.600229 | -4.0556 | 7.59E-05 | 0.000308 | 0.88326 |
| RPS6KA1 | 0.50458 | 3.53816 | 4.05539 | 7.59E-05 | 0.000309 | 0.88242 |
| PRR24 | 0.53744 | 4.144402 | 4.05293 | 7.67E-05 | 0.000312 | 0.87334 |
| RND3 | 0.61242 | 3.983359 | 4.04957 | 7.77E-05 | 0.000315 | 0.86099 |
| PHACTR1 | -0.5265 | 4.569719 | -4.0491 | 7.78E-05 | 0.000316 | 0.85933 |
| RP11-288G11.3 | -0.959 | 2.382158 | -4.049 | 7.78E-05 | 0.000316 | 0.85907 |
| LRAT | 0.58398 | 1.369296 | 4.04862 | 7.80E-05 | 0.000316 | 0.85749 |
| CACNG8 | -0.6333 | 2.387366 | -4.0455 | 7.89E-05 | 0.000319 | 0.84614 |
| ITPKA | -0.7648 | 2.802973 | -4.0449 | 7.91E-05 | 0.00032 | 0.84373 |
| RP11-11N9.4 | 0.65909 | 2.199393 | 4.04476 | 7.92E-05 | 0.00032 | 0.8433 |
| GLIS3 | 0.66253 | 3.541818 | 4.04453 | 7.92E-05 | 0.00032 | 0.84246 |
| GRM5 | -0.7156 | 1.930492 | -4.0366 | 8.17E-05 | 0.000329 | 0.81336 |
| EEF1A2 | -0.9696 | 5.849873 | -4.0363 | 8.18E-05 | 0.000329 | 0.81206 |
| LRRC52 | 0.53926 | 0.557853 | 4.03182 | 8.32E-05 | 0.000335 | 0.79579 |
| SPI1 | 0.60978 | 4.661875 | 4.03057 | 8.36E-05 | 0.000336 | 0.79122 |
| SH2D3A | 0.54318 | 0.710159 | 4.0305 | 8.37E-05 | 0.000336 | 0.79098 |
| GYLTL1B | 0.56488 | 0.642986 | 4.02966 | 8.39E-05 | 0.000337 | 0.78789 |
| SLIT1 | -0.9362 | 4.57104 | -4.0283 | 8.44E-05 | 0.000339 | 0.78299 |
| SDS | -0.7126 | 3.320933 | -4.0267 | 8.49E-05 | 0.00034 | 0.77707 |
| RP4-794H19.4 | 0.60732 | 0.662676 | 4.0226 | 8.63E-05 | 0.000345 | 0.76203 |
| HMCN2 | 0.76821 | 1.425005 | 4.02182 | 8.65E-05 | 0.000346 | 0.75917 |
| CTD-2521M24.5 | 0.57971 | 0.585172 | 4.02143 | 8.67E-05 | 0.000346 | 0.75774 |
| RP11-133F8.2 | -0.6927 | 1.621972 | -4.0194 | 8.74E-05 | 0.000349 | 0.75017 |
| PTPN5 | -0.9643 | 2.848651 | -4.0192 | 8.74E-05 | 0.000349 | 0.74966 |
| SOX4 | 0.82976 | 5.744473 | 4.01415 | 8.91E-05 | 0.000355 | 0.73115 |
| KCNAB1 | -0.5809 | 2.877624 | -4.0127 | 8.96E-05 | 0.000357 | 0.72593 |
| FKBP9 | 0.6195 | 4.634 | 4.01101 | 9.02E-05 | 0.000359 | 0.71967 |
| DLGAP3 | -0.7573 | 2.925447 | -4.0108 | 9.03E-05 | 0.000359 | 0.71895 |
| SDPR | -0.6711 | 3.669736 | -4.0093 | 9.08E-05 | 0.000361 | 0.71335 |
| ANO4 | -0.5313 | 2.131756 | -4.0082 | 9.12E-05 | 0.000362 | 0.70942 |
| NDRG4 | -0.5108 | 7.838737 | -4.004 | 9.27E-05 | 0.000367 | 0.69411 |
| MS4A7 | 0.71192 | 4.42522 | 4.00278 | 9.32E-05 | 0.000368 | 0.68967 |
| ATF3 | 0.78884 | 4.007022 | 4.00273 | 9.32E-05 | 0.000368 | 0.68948 |
| ZDHHC22 | -0.889 | 5.220229 | -4.0017 | 9.35E-05 | 0.00037 | 0.68586 |
| KCNS1 | -0.7468 | 1.194549 | -3.9967 | 9.54E-05 | 0.000376 | 0.66752 |
| FAM153C | -0.6143 | 1.140904 | -3.995 | 9.60E-05 | 0.000378 | 0.66138 |
| TTC9B | -0.8564 | 4.029301 | -3.9946 | 9.61E-05 | 0.000379 | 0.66005 |
| FAM155A | -0.7282 | 3.771329 | -3.9885 | 9.84E-05 | 0.000386 | 0.63771 |
| OSBPL1A | -0.5355 | 5.310787 | -3.9885 | 9.84E-05 | 0.000386 | 0.63756 |
| IGKC | 1.51538 | 3.103965 | 3.98396 | 0.0001 | 0.000392 | 0.62122 |
| HK2 | 0.60853 | 3.020044 | 3.98348 | 0.0001 | 0.000393 | 0.61947 |
| PCP4L1 | -0.9416 | 2.445827 | -3.9808 | 0.0001 | 0.000397 | 0.60974 |
| GRIK3 | 0.60885 | 4.298988 | 3.98015 | 0.0001 | 0.000397 | 0.60739 |
| SPHK1 | 0.55335 | 2.737889 | 3.97849 | 0.0001 | 0.000399 | 0.60136 |
| SCN1B | -0.6746 | 4.726587 | -3.9771 | 0.0001 | 0.000401 | 0.59645 |
| TNNT2 | -0.7901 | 1.459748 | -3.9771 | 0.0001 | 0.000401 | 0.59621 |
| WDR86 | -0.5304 | 3.19733 | -3.9755 | 0.0001 | 0.000403 | 0.59056 |
| KCTD4 | -0.6259 | 2.441777 | -3.9712 | 0.00011 | 0.000409 | 0.575 |
| GSC | 0.52357 | 1.174435 | 3.96945 | 0.00011 | 0.000412 | 0.56861 |
| F13A1 | 0.92467 | 2.925741 | 3.96861 | 0.00011 | 0.000413 | 0.56558 |
| SAA2 | 0.88394 | 0.711552 | 3.96772 | 0.00011 | 0.000414 | 0.56235 |
| CALB1 | -0.7443 | 2.144781 | -3.9673 | 0.00011 | 0.000415 | 0.56082 |
| SLITRK5 | -0.5342 | 3.005297 | -3.9649 | 0.00011 | 0.000418 | 0.55215 |
| OLFM3 | -0.7205 | 1.49825 | -3.9629 | 0.00011 | 0.00042 | 0.54507 |
| ZNF385B | -0.5596 | 1.466757 | -3.9625 | 0.00011 | 0.000421 | 0.54364 |
| CNDP1 | -1.2618 | 4.226484 | -3.9607 | 0.00011 | 0.000423 | 0.53716 |
| DMRTA2 | 0.65154 | 0.853214 | 3.95933 | 0.00011 | 0.000426 | 0.53204 |
| SCIN | 0.8951 | 3.16458 | 3.95829 | 0.00011 | 0.000427 | 0.52828 |
| CTD-2540B15.7 | 0.65662 | 1.197178 | 3.95692 | 0.00011 | 0.000429 | 0.52335 |
| BEX1 | -0.6438 | 7.980304 | -3.9565 | 0.00011 | 0.00043 | 0.52172 |
| TYROBP | 0.63836 | 6.774016 | 3.95372 | 0.00011 | 0.000434 | 0.51181 |
| RP3-460G2.2 | 0.67193 | 0.811438 | 3.95369 | 0.00011 | 0.000434 | 0.51169 |
| CHL1-AS2 | -0.6186 | 1.944889 | -3.9531 | 0.00011 | 0.000434 | 0.50954 |
| PLCH2 | -0.5882 | 2.949155 | -3.9525 | 0.00011 | 0.000435 | 0.50753 |
| VASN | 0.70512 | 1.953028 | 3.95227 | 0.00011 | 0.000436 | 0.50656 |
| LINC00966 | -0.6535 | 2.239189 | -3.9496 | 0.00011 | 0.000439 | 0.49694 |
| GRM5-AS1 | -0.6684 | 1.169584 | -3.9484 | 0.00011 | 0.000441 | 0.49244 |
| EPB41L3 | -0.5594 | 5.593528 | -3.945 | 0.00012 | 0.000447 | 0.48021 |
| NT5DC1 | -0.5212 | 3.382569 | -3.9439 | 0.00012 | 0.000448 | 0.4763 |
| MGAT5B | -0.6582 | 3.345444 | -3.9423 | 0.00012 | 0.000451 | 0.47062 |
| PDXP | -0.6549 | 5.30073 | -3.9417 | 0.00012 | 0.000452 | 0.46834 |
| ACP5 | 0.64705 | 2.344753 | 3.93879 | 0.00012 | 0.000456 | 0.45803 |
| PTGS1 | 0.61002 | 3.02364 | 3.93733 | 0.00012 | 0.000458 | 0.45278 |
| RIIAD1 | -0.7054 | 2.632184 | -3.9347 | 0.00012 | 0.000462 | 0.44331 |
| HLA-DRB6 | 0.50253 | 0.711143 | 3.93253 | 0.00012 | 0.000465 | 0.43554 |
| RP11-566K19.6 | -0.7136 | 1.893229 | -3.9325 | 0.00012 | 0.000465 | 0.43542 |
| CERCAM | -0.6818 | 5.845941 | -3.932 | 0.00012 | 0.000466 | 0.43364 |
| SLITRK1 | -0.6581 | 2.828264 | -3.9304 | 0.00012 | 0.000468 | 0.42784 |
| SLC35F3 | -0.5159 | 1.215385 | -3.9288 | 0.00012 | 0.000471 | 0.42227 |
| CTSS | 0.67426 | 5.11435 | 3.9255 | 0.00013 | 0.000477 | 0.4103 |
| MYC | 0.62338 | 4.944932 | 3.92463 | 0.00013 | 0.000478 | 0.4072 |
| S100A9 | 1.02973 | 4.187713 | 3.91683 | 0.00013 | 0.000491 | 0.37924 |
| HOXB3 | 0.66753 | 0.770633 | 3.91616 | 0.00013 | 0.000492 | 0.37685 |
| LRRC17 | 0.63645 | 3.23608 | 3.91432 | 0.00013 | 0.000495 | 0.37028 |
| KIF5C | -0.5217 | 6.26711 | -3.9139 | 0.00013 | 0.000495 | 0.36892 |
| MIA | 0.67914 | 1.291527 | 3.91314 | 0.00013 | 0.000496 | 0.36605 |
| PHYHIPL | -0.6087 | 7.268042 | -3.9127 | 0.00013 | 0.000497 | 0.36445 |
| PDE2A | -0.7258 | 4.866872 | -3.9114 | 0.00013 | 0.000499 | 0.35999 |
| CYP4X1 | -0.5309 | 1.427926 | -3.9069 | 0.00013 | 0.000506 | 0.34384 |
| PTPRD | -0.5576 | 4.829321 | -3.9065 | 0.00013 | 0.000507 | 0.34243 |
| LINC00403 | -0.6134 | 2.621792 | -3.9056 | 0.00014 | 0.000508 | 0.33918 |
| PTPRZ1 | 0.56576 | 8.627772 | 3.90553 | 0.00014 | 0.000509 | 0.33887 |
| FCGR2C | 0.89829 | 2.165822 | 3.90512 | 0.00014 | 0.000509 | 0.3374 |
| CPXM1 | 0.82124 | 4.705785 | 3.90428 | 0.00014 | 0.000511 | 0.33438 |
| TRAC | 0.54955 | 1.363019 | 3.90397 | 0.00014 | 0.000511 | 0.3333 |
| ZNF204P | -0.6217 | 2.281381 | -3.9036 | 0.00014 | 0.000512 | 0.33214 |
| INHBA | 0.51714 | 1.433077 | 3.90114 | 0.00014 | 0.000516 | 0.3232 |
| OAS2 | 0.59609 | 2.983363 | 3.89909 | 0.00014 | 0.000519 | 0.31587 |
| INSM1 | 0.65633 | 2.549084 | 3.89896 | 0.00014 | 0.000519 | 0.31542 |
| STK32B | 0.54815 | 1.957465 | 3.8963 | 0.00014 | 0.000524 | 0.30593 |
| UNC13A | -0.6709 | 3.96059 | -3.8959 | 0.00014 | 0.000524 | 0.30464 |
| FXYD5 | 0.55791 | 4.838751 | 3.89453 | 0.00014 | 0.000527 | 0.29962 |
| FLNC | 0.89517 | 2.907285 | 3.89422 | 0.00014 | 0.000527 | 0.29852 |
| INHBE | 0.55717 | 0.710759 | 3.89226 | 0.00014 | 0.000531 | 0.29154 |
| RP11-64B16.2 | -0.6886 | 2.678573 | -3.8918 | 0.00014 | 0.000531 | 0.29 |
| GPR101 | 0.54068 | 0.631896 | 3.88858 | 0.00014 | 0.000537 | 0.27844 |
| PRCD | -0.612 | 3.063203 | -3.8872 | 0.00014 | 0.00054 | 0.2737 |
| PLLP | -0.8124 | 5.164526 | -3.8856 | 0.00015 | 0.000543 | 0.26802 |
| RP11-597D13.7 | -0.7135 | 1.694253 | -3.8855 | 0.00015 | 0.000543 | 0.26747 |
| RP11-275H4.1 | -0.6805 | 1.471135 | -3.8852 | 0.00015 | 0.000543 | 0.26654 |
| MKX | -0.5806 | 1.94264 | -3.8775 | 0.00015 | 0.000557 | 0.23914 |
| IGHG2 | 1.23071 | 1.626941 | 3.87724 | 0.00015 | 0.000558 | 0.23815 |
| EFNB2 | 0.52493 | 3.1009 | 3.87615 | 0.00015 | 0.000559 | 0.23428 |
| HECW1 | -0.578 | 1.904373 | -3.8745 | 0.00015 | 0.000563 | 0.2286 |
| COL26A1 | -0.5692 | 1.788582 | -3.8739 | 0.00015 | 0.000564 | 0.22614 |
| MAOA | -0.5293 | 4.044141 | -3.8736 | 0.00015 | 0.000564 | 0.22522 |
| RXRG | -0.5104 | 2.483007 | -3.8722 | 0.00015 | 0.000567 | 0.22016 |
| SHISA9 | -0.7521 | 2.903245 | -3.872 | 0.00015 | 0.000567 | 0.21941 |
| L1CAM | -1.0042 | 4.163544 | -3.8716 | 0.00015 | 0.000568 | 0.21828 |
| SRRM3 | -0.7534 | 3.743207 | -3.8715 | 0.00015 | 0.000568 | 0.21792 |
| PCDHB7 | 0.68626 | 1.903033 | 3.86959 | 0.00015 | 0.000571 | 0.21103 |
| TUBA8 | -0.7046 | 2.57001 | -3.8695 | 0.00015 | 0.000572 | 0.21061 |
| HIST1H2AC | 0.52183 | 4.327986 | 3.86845 | 0.00016 | 0.000574 | 0.20698 |
| FAM153A | -0.5254 | 1.022582 | -3.867 | 0.00016 | 0.000576 | 0.202 |
| FAHD2CP | -0.6046 | 4.474472 | -3.866 | 0.00016 | 0.000578 | 0.19844 |
| SAA1 | 1.07636 | 0.90191 | 3.86503 | 0.00016 | 0.00058 | 0.19487 |
| PTN | 0.5088 | 8.325862 | 3.86477 | 0.00016 | 0.00058 | 0.19396 |
| TLCD1 | -0.5138 | 3.28523 | -3.8644 | 0.00016 | 0.000581 | 0.19259 |
| HSPB1 | 0.67414 | 7.315065 | 3.86424 | 0.00016 | 0.000581 | 0.1921 |
| TEX38 | 0.57086 | 0.697585 | 3.86364 | 0.00016 | 0.000582 | 0.18998 |
| SLC44A5 | 0.62217 | 2.525825 | 3.86098 | 0.00016 | 0.000588 | 0.18055 |
| BMP8B | 0.56688 | 1.83091 | 3.86091 | 0.00016 | 0.000588 | 0.1803 |
| RGS4 | -1.1152 | 3.895463 | -3.858 | 0.00016 | 0.000594 | 0.16992 |
| TMEM132D | -0.6125 | 1.320808 | -3.8561 | 0.00016 | 0.000597 | 0.16347 |
| RP11-116O18.1 | -0.6609 | 1.138646 | -3.8558 | 0.00016 | 0.000598 | 0.16222 |
| PVALB | -0.9878 | 2.086931 | -3.8508 | 0.00017 | 0.000608 | 0.14448 |
| GABRE | 0.5454 | 1.46067 | 3.84634 | 0.00017 | 0.000617 | 0.12887 |
| C1QL3 | -0.8214 | 1.463367 | -3.8457 | 0.00017 | 0.000618 | 0.12657 |
| NES | 0.70823 | 6.559054 | 3.8415 | 0.00017 | 0.000627 | 0.1118 |
| OPCML | -0.7143 | 3.439473 | -3.8403 | 0.00017 | 0.00063 | 0.10759 |
| PTPRR | -0.5986 | 1.507891 | -3.839 | 0.00017 | 0.000632 | 0.10318 |
| RP11-1055B8.3 | -0.6987 | 3.060336 | -3.8377 | 0.00017 | 0.000634 | 0.09851 |
| DAAM2 | -0.8749 | 6.456094 | -3.8374 | 0.00017 | 0.000635 | 0.09739 |
| TULP1 | 0.54095 | 0.553713 | 3.83653 | 0.00018 | 0.000637 | 0.09431 |
| FRMPD2 | -0.519 | 1.462109 | -3.8359 | 0.00018 | 0.000638 | 0.09202 |
| RN7SL138P | 0.54503 | 1.885783 | 3.83401 | 0.00018 | 0.000642 | 0.08545 |
| ZNF536 | -0.6824 | 2.674725 | -3.8321 | 0.00018 | 0.000646 | 0.07866 |
| LRP4 | -0.5112 | 5.893148 | -3.8285 | 0.00018 | 0.000654 | 0.06625 |
| GPR98 | -0.7492 | 4.752873 | -3.8243 | 0.00018 | 0.000663 | 0.0514 |
| WSCD1 | 0.52438 | 5.202354 | 3.82312 | 0.00018 | 0.000666 | 0.04724 |
| FSCN3 | 0.54141 | 0.83439 | 3.82275 | 0.00018 | 0.000666 | 0.04592 |
| ERAP2 | 0.64839 | 2.65123 | 3.81669 | 0.00019 | 0.00068 | 0.02468 |
| IL18 | 0.62017 | 3.513526 | 3.81554 | 0.00019 | 0.000682 | 0.02068 |
| ME1 | -0.603 | 2.990346 | -3.8126 | 0.00019 | 0.000689 | 0.01052 |
| RAPGEF5 | -0.6762 | 4.235604 | -3.8119 | 0.00019 | 0.00069 | 0.00796 |
| AC064875.2 | 0.56238 | 0.805366 | 3.81172 | 0.00019 | 0.00069 | 0.0073 |
| PACSIN3 | -0.5331 | 3.187794 | -3.8088 | 0.00019 | 0.000697 | -0.0029 |
| ZNF365 | -0.504 | 3.512491 | -3.8071 | 0.0002 | 0.000701 | -0.0088 |
| CLVS1 | -0.6182 | 2.034831 | -3.8064 | 0.0002 | 0.000703 | -0.0112 |
| LRTM2 | -0.7088 | 1.806828 | -3.7998 | 0.0002 | 0.000719 | -0.0344 |
| RIPPLY2 | -0.5821 | 3.588966 | -3.7983 | 0.0002 | 0.000723 | -0.0396 |
| PCBP3 | -0.5382 | 3.345148 | -3.7978 | 0.0002 | 0.000724 | -0.0414 |
| CAMK2N1 | -0.5403 | 6.797073 | -3.7968 | 0.0002 | 0.000726 | -0.0447 |
| HIST2H2BE | 0.50324 | 3.879263 | 3.79558 | 0.0002 | 0.000729 | -0.0491 |
| RPRML | -0.7204 | 1.527393 | -3.7935 | 0.00021 | 0.000734 | -0.0563 |
| LAPTM5 | 0.62651 | 6.840655 | 3.79341 | 0.00021 | 0.000734 | -0.0566 |
| SYNJ2 | -0.6826 | 3.021205 | -3.7927 | 0.00021 | 0.000736 | -0.0589 |
| LAMP5 | -0.6092 | 3.981271 | -3.7904 | 0.00021 | 0.000741 | -0.067 |
| IL1R1 | 0.5689 | 2.181516 | 3.78886 | 0.00021 | 0.000745 | -0.0725 |
| PTHLH | 0.54059 | 2.315242 | 3.78852 | 0.00021 | 0.000746 | -0.0737 |
| ANKS1B | -0.6085 | 5.326697 | -3.7858 | 0.00021 | 0.000752 | -0.0832 |
| PPAP2C | -0.7989 | 3.479927 | -3.7857 | 0.00021 | 0.000752 | -0.0834 |
| DDR2 | 0.63648 | 3.637546 | 3.7815 | 0.00022 | 0.000763 | -0.0981 |
| RAB26 | -0.5411 | 3.04084 | -3.7804 | 0.00022 | 0.000765 | -0.102 |
| RP11-155G14.5 | 0.62598 | 1.51807 | 3.78031 | 0.00022 | 0.000765 | -0.1022 |
| C1QL2 | -0.6002 | 1.585065 | -3.7797 | 0.00022 | 0.000767 | -0.1045 |
| AMOT | -0.5447 | 4.552755 | -3.7794 | 0.00022 | 0.000768 | -0.1054 |
| CCR1 | 0.5909 | 2.936433 | 3.77713 | 0.00022 | 0.000773 | -0.1133 |
| HIST2H2BB | 0.52094 | 1.849738 | 3.77278 | 0.00022 | 0.000784 | -0.1283 |
| ZIC1 | 0.68034 | 4.828884 | 3.77238 | 0.00022 | 0.000785 | -0.1297 |
| BTK | 0.51926 | 2.873226 | 3.77152 | 0.00022 | 0.000787 | -0.1327 |
| CRYAA | 0.5116 | 0.782186 | 3.77081 | 0.00022 | 0.000789 | -0.1352 |
| G0S2 | 0.77051 | 1.380487 | 3.76945 | 0.00023 | 0.000792 | -0.1399 |
| AMER3 | -0.5992 | 1.745932 | -3.7689 | 0.00023 | 0.000793 | -0.1416 |
| SCG3 | -0.8191 | 7.472059 | -3.7647 | 0.00023 | 0.000805 | -0.1564 |
| SSTR1 | -0.6798 | 1.965759 | -3.7629 | 0.00023 | 0.00081 | -0.1624 |
| DNM1 | -0.8502 | 5.298269 | -3.7624 | 0.00023 | 0.000811 | -0.1642 |
| IL10RA | 0.50419 | 2.986504 | 3.7609 | 0.00023 | 0.000815 | -0.1695 |
| 4-Mar | -0.6515 | 1.94788 | -3.7599 | 0.00023 | 0.000818 | -0.173 |
| BEX5 | -0.8151 | 3.406192 | -3.7537 | 0.00024 | 0.000834 | -0.1944 |
| HAVCR2 | 0.5652 | 3.718706 | 3.75246 | 0.00024 | 0.000837 | -0.1986 |
| FPR1 | 0.71037 | 3.641668 | 3.75053 | 0.00024 | 0.000843 | -0.2053 |
| MATN2 | 0.64572 | 5.502933 | 3.75017 | 0.00024 | 0.000843 | -0.2066 |
| EFEMP2 | 0.62609 | 4.576914 | 3.75017 | 0.00024 | 0.000843 | -0.2066 |
| IGSF6 | 0.5369 | 3.528459 | 3.74901 | 0.00024 | 0.000847 | -0.2106 |
| STMN2 | -1.273 | 5.333898 | -3.7479 | 0.00024 | 0.000849 | -0.2145 |
| DNAJA4 | -0.6408 | 3.819049 | -3.7451 | 0.00025 | 0.000856 | -0.2239 |
| NR2F2 | 0.5769 | 3.166479 | 3.73697 | 0.00025 | 0.00088 | -0.252 |
| ABHD12B | -0.5012 | 1.412538 | -3.7355 | 0.00026 | 0.000883 | -0.2572 |
| GPR62 | -0.6759 | 2.029697 | -3.7323 | 0.00026 | 0.000893 | -0.2682 |
| PLIN2 | 0.54041 | 4.11174 | 3.73068 | 0.00026 | 0.000897 | -0.2737 |
| RP11-326C3.7 | -0.6204 | 2.33707 | -3.7262 | 0.00026 | 0.00091 | -0.2891 |
| CELF3 | -0.8153 | 4.588002 | -3.7256 | 0.00026 | 0.000912 | -0.291 |
| MS4A4A | 0.72408 | 3.571406 | 3.72113 | 0.00027 | 0.000925 | -0.3064 |
| TNFSF13B | 0.72248 | 3.044662 | 3.71538 | 0.00027 | 0.000943 | -0.3261 |
| CDH20 | -0.5739 | 4.646201 | -3.715 | 0.00027 | 0.000944 | -0.3273 |
| GHRHR | -0.507 | 2.12218 | -3.712 | 0.00028 | 0.000954 | -0.3378 |
| UHRF1 | 0.61532 | 3.929126 | 3.71189 | 0.00028 | 0.000954 | -0.3381 |
| ITGB2 | 0.64192 | 5.24754 | 3.69474 | 0.0003 | 0.001005 | -0.3966 |
| YPEL4 | -0.509 | 3.815935 | -3.6914 | 0.0003 | 0.001017 | -0.4081 |
| TLR2 | 0.62207 | 2.522897 | 3.69046 | 0.0003 | 0.00102 | -0.4112 |
| IGHM | 0.77974 | 1.215157 | 3.69029 | 0.0003 | 0.00102 | -0.4118 |
| SBSN | 0.68373 | 0.745936 | 3.68904 | 0.0003 | 0.001024 | -0.416 |
| LINC00982 | -0.5581 | 2.461458 | -3.6888 | 0.0003 | 0.001024 | -0.4168 |
| SLC16A4 | 0.54142 | 2.960792 | 3.68621 | 0.00031 | 0.001033 | -0.4256 |
| OASL | 0.53222 | 1.539101 | 3.6842 | 0.00031 | 0.00104 | -0.4325 |
| BRSK2 | -0.526 | 4.898284 | -3.6832 | 0.00031 | 0.001043 | -0.436 |
| DIRAS2 | -0.7075 | 3.730292 | -3.6792 | 0.00031 | 0.001056 | -0.4494 |
| CTSZ | 0.54587 | 6.149945 | 3.67543 | 0.00032 | 0.001069 | -0.4623 |
| MGAT4C | -0.681 | 2.815441 | -3.6749 | 0.00032 | 0.00107 | -0.4641 |
| DNAH7 | -0.5166 | 2.322293 | -3.6733 | 0.00032 | 0.001076 | -0.4694 |
| EDN3 | -0.5253 | 2.475839 | -3.6664 | 0.00033 | 0.001099 | -0.4929 |
| MYRF | -0.8904 | 4.487354 | -3.6626 | 0.00033 | 0.001112 | -0.5056 |
| RGS7BP | -0.6035 | 2.513262 | -3.6609 | 0.00033 | 0.001119 | -0.5114 |
| TMSB4XP8 | 0.59466 | 3.556391 | 3.65467 | 0.00034 | 0.001141 | -0.5325 |
| C3 | 0.78599 | 7.698882 | 3.65311 | 0.00034 | 0.001146 | -0.5378 |
| BATF3 | 0.50161 | 2.53879 | 3.65199 | 0.00035 | 0.00115 | -0.5416 |
| CLEC7A | 0.59626 | 2.736801 | 3.65106 | 0.00035 | 0.001154 | -0.5447 |
| GPRASP1 | -0.5753 | 3.623328 | -3.6496 | 0.00035 | 0.001159 | -0.5496 |
| ISG15 | 0.70275 | 5.670755 | 3.64659 | 0.00035 | 0.00117 | -0.5598 |
| RP11-472N13.3 | 0.54843 | 2.110719 | 3.64513 | 0.00035 | 0.001175 | -0.5647 |
| PTPN6 | 0.50208 | 3.930953 | 3.64475 | 0.00035 | 0.001176 | -0.566 |
| DENND2D | 0.5457 | 2.014562 | 3.64461 | 0.00036 | 0.001177 | -0.5664 |
| NNAT | -1.0836 | 3.863959 | -3.643 | 0.00036 | 0.001183 | -0.572 |
| PIP4K2A | -0.5043 | 5.717127 | -3.6417 | 0.00036 | 0.001187 | -0.5762 |
| AKR1C2 | -0.5196 | 2.01239 | -3.6397 | 0.00036 | 0.001195 | -0.583 |
| HSD11B1 | -0.5617 | 1.820118 | -3.6377 | 0.00036 | 0.001202 | -0.5896 |
| SERPINA5 | 0.73699 | 1.284179 | 3.63683 | 0.00037 | 0.001205 | -0.5926 |
| FAS | 0.56741 | 2.838233 | 3.63672 | 0.00037 | 0.001205 | -0.593 |
| PCSK6 | -0.8588 | 4.566686 | -3.6354 | 0.00037 | 0.00121 | -0.5973 |
| ANKRD22 | 0.65517 | 1.839972 | 3.63291 | 0.00037 | 0.001221 | -0.6058 |
| SERTM1 | -0.5276 | 0.961254 | -3.6311 | 0.00037 | 0.001227 | -0.6118 |
| DKK1 | 0.56402 | 1.279286 | 3.6276 | 0.00038 | 0.001241 | -0.6236 |
| RASD2 | -0.6003 | 2.413298 | -3.6268 | 0.00038 | 0.001244 | -0.6263 |
| TMIE | -0.5298 | 2.528762 | -3.626 | 0.00038 | 0.001247 | -0.6291 |
| IGFBP5 | 0.70436 | 6.125731 | 3.62506 | 0.00038 | 0.001251 | -0.6321 |
| SPHKAP | -0.7165 | 2.366223 | -3.625 | 0.00038 | 0.001251 | -0.6325 |
| PLEK | 0.56871 | 3.174755 | 3.62284 | 0.00038 | 0.00126 | -0.6396 |
| ALOX5AP | 0.75286 | 4.824765 | 3.61931 | 0.00039 | 0.001274 | -0.6514 |
| CNP | -0.5472 | 8.124361 | -3.6182 | 0.00039 | 0.001278 | -0.655 |
| RP11-262H14.3 | -0.6882 | 1.888707 | -3.618 | 0.00039 | 0.001279 | -0.6557 |
| TNR | -0.9767 | 5.516575 | -3.6149 | 0.0004 | 0.001291 | -0.6663 |
| PPFIA2 | -0.5612 | 3.776438 | -3.6098 | 0.0004 | 0.001312 | -0.6832 |
| RP11-298D21.1 | -0.6673 | 1.625561 | -3.6081 | 0.0004 | 0.001319 | -0.6887 |
| NGFR | 0.68891 | 2.600901 | 3.60789 | 0.00041 | 0.001319 | -0.6896 |
| HLA-DRB1 | 0.95741 | 5.279701 | 3.60633 | 0.00041 | 0.001326 | -0.6948 |
| PAK3 | -0.5643 | 2.818937 | -3.6051 | 0.00041 | 0.001332 | -0.6988 |
| GDAP1L1 | -0.7903 | 5.466294 | -3.6045 | 0.00041 | 0.001334 | -0.7009 |
| IGLC1 | 1.08353 | 1.410801 | 3.60092 | 0.00042 | 0.001349 | -0.7128 |
| SELENBP1 | -0.6097 | 3.715973 | -3.6003 | 0.00042 | 0.001352 | -0.715 |
| SPRY4 | 0.72236 | 2.665523 | 3.5985 | 0.00042 | 0.001359 | -0.7209 |
| ZMAT4 | -0.5361 | 1.457562 | -3.5947 | 0.00042 | 0.001376 | -0.7337 |
| SRGN | 0.52449 | 5.864 | 3.59403 | 0.00043 | 0.001378 | -0.7358 |
| ELAVL2 | -0.7103 | 3.071027 | -3.5902 | 0.00043 | 0.001394 | -0.7486 |
| SPOCK1 | -0.6052 | 5.832055 | -3.5882 | 0.00043 | 0.001402 | -0.755 |
| S1PR5 | -0.7166 | 2.484391 | -3.5881 | 0.00043 | 0.001403 | -0.7554 |
| GZMA | 0.53583 | 1.16321 | 3.58124 | 0.00045 | 0.001433 | -0.7783 |
| HSPA1B | 0.67549 | 6.388731 | 3.57907 | 0.00045 | 0.001443 | -0.7855 |
| HSPA7 | 0.75926 | 1.856909 | 3.57621 | 0.00045 | 0.001454 | -0.7949 |
| GPC5 | -0.5619 | 2.335594 | -3.576 | 0.00045 | 0.001455 | -0.7958 |
| MMP16 | 0.55251 | 3.541164 | 3.57537 | 0.00045 | 0.001458 | -0.7977 |
| PARVG | 0.55111 | 3.641365 | 3.57438 | 0.00046 | 0.001462 | -0.801 |
| IGLC3 | 1.15273 | 1.735662 | 3.56971 | 0.00046 | 0.001483 | -0.8165 |
| PCSK1N | -0.5346 | 7.752522 | -3.5678 | 0.00047 | 0.001492 | -0.8229 |
| RP11-785H5.1 | 0.63574 | 3.514187 | 3.56651 | 0.00047 | 0.001498 | -0.8271 |
| RP11-79P5.2 | 0.74171 | 1.980471 | 3.5663 | 0.00047 | 0.001498 | -0.8277 |
| MEOX2 | 0.69319 | 0.853159 | 3.56578 | 0.00047 | 0.001501 | -0.8295 |
| MSX2 | -0.6713 | 1.583487 | -3.5625 | 0.00048 | 0.001516 | -0.8404 |
| CACNG5 | 0.51122 | 0.752456 | 3.56052 | 0.00048 | 0.001526 | -0.8468 |
| SLA | 0.5222 | 3.579679 | 3.55636 | 0.00049 | 0.001546 | -0.8606 |
| FAM57B | -0.642 | 4.28594 | -3.5551 | 0.00049 | 0.001552 | -0.8647 |
| AC007192.6 | -0.8402 | 2.393029 | -3.554 | 0.00049 | 0.001557 | -0.8684 |
| LYVE1 | -0.6786 | 2.90087 | -3.5516 | 0.00049 | 0.001569 | -0.8763 |
| MEGF10 | -0.5706 | 3.149973 | -3.5505 | 0.0005 | 0.001574 | -0.88 |
| SLC14A1 | -1.0611 | 4.120925 | -3.5488 | 0.0005 | 0.001582 | -0.8853 |
| COBL | -0.5302 | 4.724449 | -3.5488 | 0.0005 | 0.001582 | -0.8856 |
| NEGR1 | -0.6267 | 3.736781 | -3.5469 | 0.0005 | 0.001592 | -0.8919 |
| STMN4 | -0.597 | 6.594592 | -3.5436 | 0.00051 | 0.001608 | -0.9026 |
| GATSL3 | -0.5176 | 4.30708 | -3.5408 | 0.00051 | 0.001621 | -0.9117 |
| CTA-134P22.2 | -0.5883 | 3.075986 | -3.5351 | 0.00052 | 0.00165 | -0.9306 |
| CD52 | 0.694 | 2.920507 | 3.53478 | 0.00053 | 0.001651 | -0.9316 |
| STX1A | -0.693 | 4.496377 | -3.5311 | 0.00053 | 0.001671 | -0.9437 |
| FGFR3 | -0.8366 | 4.700452 | -3.53 | 0.00053 | 0.001676 | -0.9474 |
| RP11-14N7.2 | -0.6548 | 3.947753 | -3.5252 | 0.00054 | 0.001701 | -0.9629 |
| WNT7B | -0.7599 | 2.68421 | -3.5249 | 0.00054 | 0.001703 | -0.9639 |
| SIDT1 | -0.501 | 2.596534 | -3.5235 | 0.00055 | 0.00171 | -0.9686 |
| CPNE7 | -0.5914 | 1.684377 | -3.5202 | 0.00055 | 0.001729 | -0.9793 |
| CNTFR | -0.6165 | 5.309162 | -3.5201 | 0.00055 | 0.001729 | -0.9797 |
| S100A10 | 0.82583 | 6.773311 | 3.51824 | 0.00056 | 0.001738 | -0.9857 |
| NCDN | -0.598 | 5.688231 | -3.5111 | 0.00057 | 0.001777 | -1.0091 |
| PITPNM3 | -0.6097 | 2.351675 | -3.5094 | 0.00057 | 0.001786 | -1.0145 |
| C21orf37 | -0.5269 | 1.621301 | -3.5074 | 0.00058 | 0.001797 | -1.0212 |
| S100A8 | 0.90953 | 3.260148 | 3.50702 | 0.00058 | 0.001799 | -1.0223 |
| GJB1 | -0.9441 | 3.726739 | -3.5062 | 0.00058 | 0.001803 | -1.025 |
| PRSS3 | -0.6544 | 2.320567 | -3.503 | 0.00059 | 0.001821 | -1.0354 |
| RTBDN | -0.568 | 1.434057 | -3.4968 | 0.0006 | 0.001854 | -1.0555 |
| RAB34 | 0.58886 | 3.958371 | 3.49333 | 0.00061 | 0.001874 | -1.0668 |
| NEUROD2 | -0.764 | 1.888881 | -3.4927 | 0.00061 | 0.001878 | -1.0689 |
| PPP1R14A | -0.8251 | 5.324612 | -3.49 | 0.00061 | 0.001893 | -1.0776 |
| CTB-1I21.1 | -0.6171 | 1.35001 | -3.489 | 0.00062 | 0.001899 | -1.0809 |
| RN7SL3 | -0.7736 | 1.94715 | -3.4815 | 0.00063 | 0.001942 | -1.1053 |
| SERPING1 | 0.65368 | 5.58911 | 3.48002 | 0.00064 | 0.00195 | -1.11 |
| GJC2 | -0.6691 | 2.753895 | -3.48 | 0.00064 | 0.00195 | -1.11 |
| NXPH4 | 0.51675 | 2.087675 | 3.47873 | 0.00064 | 0.001958 | -1.1142 |
| RNF175 | -0.5742 | 2.492928 | -3.4784 | 0.00064 | 0.00196 | -1.1154 |
| LINC01088 | -1.0628 | 4.820186 | -3.4774 | 0.00064 | 0.001965 | -1.1184 |
| ELFN2 | -0.6931 | 3.649352 | -3.4774 | 0.00064 | 0.001965 | -1.1184 |
| CSMD1 | -0.5559 | 2.614938 | -3.4703 | 0.00066 | 0.002009 | -1.1415 |
| AC002456.2 | 0.51535 | 1.858444 | 3.46918 | 0.00066 | 0.002015 | -1.145 |
| FABP5P7 | 0.68682 | 1.091377 | 3.46881 | 0.00066 | 0.002017 | -1.1462 |
| GABBR2 | -0.7222 | 4.597393 | -3.4657 | 0.00067 | 0.002035 | -1.1563 |
| AL163636.6 | 0.54097 | 0.958638 | 3.46414 | 0.00067 | 0.002045 | -1.1613 |
| LRRC55 | 0.61158 | 3.240526 | 3.4585 | 0.00069 | 0.002078 | -1.1794 |
| CYP2J2 | -0.5569 | 3.324155 | -3.4574 | 0.00069 | 0.002085 | -1.1831 |
| PLA2G2A | 0.81806 | 0.88175 | 3.45672 | 0.00069 | 0.002088 | -1.1852 |
| PLCXD3 | -0.5263 | 1.624791 | -3.4567 | 0.00069 | 0.002088 | -1.1852 |
| DARC | 0.56811 | 2.716032 | 3.45662 | 0.00069 | 0.002088 | -1.1855 |
| CTC-360G5.1 | 0.5035 | 1.465196 | 3.45074 | 0.0007 | 0.002128 | -1.2044 |
| NR4A3 | 0.53692 | 1.719908 | 3.45 | 0.00071 | 0.002132 | -1.2068 |
| SLPI | 0.73751 | 2.757856 | 3.44462 | 0.00072 | 0.002165 | -1.224 |
| RP1-20B21.4 | 0.56652 | 1.051398 | 3.44431 | 0.00072 | 0.002167 | -1.225 |
| KLK7 | -0.6096 | 1.114667 | -3.4433 | 0.00072 | 0.002174 | -1.2283 |
| CHRNB2 | -0.5784 | 2.72164 | -3.4431 | 0.00072 | 0.002175 | -1.2288 |
| XRCC6BP1 | 0.52236 | 2.198496 | 3.44044 | 0.00073 | 0.002193 | -1.2375 |
| SLC32A1 | -0.7328 | 1.830186 | -3.4404 | 0.00073 | 0.002193 | -1.2375 |
| ADCYAP1R1 | -0.6973 | 7.1536 | -3.4367 | 0.00074 | 0.002218 | -1.2495 |
| SPP1 | 0.93286 | 9.104359 | 3.43567 | 0.00074 | 0.002224 | -1.2527 |
| IGLV2-14 | 0.86567 | 1.111071 | 3.43425 | 0.00075 | 0.002233 | -1.2573 |
| SLC39A12 | -0.6846 | 2.624511 | -3.4317 | 0.00075 | 0.00225 | -1.2655 |
| RP11-439C15.4 | -0.6273 | 3.385468 | -3.4292 | 0.00076 | 0.002267 | -1.2733 |
| LTF | 1.26493 | 2.256784 | 3.42837 | 0.00076 | 0.002273 | -1.2761 |
| CELF5 | -0.6785 | 3.92066 | -3.4214 | 0.00078 | 0.00232 | -1.2983 |
| PTPRN | -0.8306 | 4.719074 | -3.4177 | 0.00079 | 0.002347 | -1.3103 |
| ATOH8 | -0.7321 | 4.709246 | -3.4087 | 0.00081 | 0.002411 | -1.3386 |
| CYBB | 0.61633 | 4.283603 | 3.40376 | 0.00083 | 0.002447 | -1.3545 |
| AC006011.4 | 0.58954 | 2.86841 | 3.40238 | 0.00083 | 0.002457 | -1.3588 |
| GPR22 | -0.5676 | 1.36882 | -3.396 | 0.00085 | 0.002505 | -1.3791 |
| DHCR24 | -0.6018 | 5.095482 | -3.3956 | 0.00085 | 0.002507 | -1.3804 |
| CCDC19 | 0.5177 | 0.798841 | 3.39522 | 0.00085 | 0.00251 | -1.3815 |
| LHX6 | -0.5139 | 1.479858 | -3.3911 | 0.00086 | 0.002542 | -1.3947 |
| FGF13 | -0.6546 | 3.620016 | -3.3861 | 0.00088 | 0.00258 | -1.4105 |
| STXBP5L | -0.5032 | 1.823781 | -3.3845 | 0.00088 | 0.002592 | -1.4154 |
| ZDHHC8P1 | -0.5584 | 1.587403 | -3.3843 | 0.00089 | 0.002594 | -1.4162 |
| NTRK2 | -0.6061 | 8.157934 | -3.3825 | 0.00089 | 0.002608 | -1.4218 |
| DGKG | -0.5337 | 3.908801 | -3.3823 | 0.00089 | 0.002609 | -1.4224 |
| CTD-3199J23.4 | -0.617 | 3.750824 | -3.3797 | 0.0009 | 0.00263 | -1.4305 |
| JUNB | 0.68464 | 6.109001 | 3.37946 | 0.0009 | 0.002632 | -1.4314 |
| TRH | 0.79743 | 1.495834 | 3.3739 | 0.00092 | 0.002677 | -1.4489 |
| AC140481.7 | -0.5735 | 1.253328 | -3.3738 | 0.00092 | 0.002677 | -1.4491 |
| LIX1 | -0.7014 | 4.888342 | -3.3719 | 0.00092 | 0.002693 | -1.4551 |
| TGFBR3L | -0.7068 | 1.778563 | -3.368 | 0.00094 | 0.002726 | -1.4675 |
| IL8 | 0.83756 | 1.830404 | 3.36743 | 0.00094 | 0.00273 | -1.4692 |
| CLDN10 | -0.7784 | 3.544387 | -3.3637 | 0.00095 | 0.002759 | -1.481 |
| COX7A1 | -0.5946 | 3.611619 | -3.3621 | 0.00095 | 0.002771 | -1.486 |
| CTB-63M22.1 | 0.54567 | 2.85967 | 3.35886 | 0.00097 | 0.002795 | -1.4962 |
| FABP6 | -0.5885 | 1.67914 | -3.3573 | 0.00097 | 0.002807 | -1.5012 |
| SFRP4 | 0.75838 | 3.033 | 3.35697 | 0.00097 | 0.002809 | -1.5021 |
| CTC-429P9.4 | 0.81839 | 2.548675 | 3.3566 | 0.00097 | 0.002812 | -1.5033 |
| GABRA4 | -0.526 | 1.383748 | -3.3557 | 0.00098 | 0.002819 | -1.5061 |
| IGHA2 | 0.68627 | 0.935553 | 3.35252 | 0.00099 | 0.002844 | -1.5161 |
| CLDN11 | -0.9173 | 5.40308 | -3.3519 | 0.00099 | 0.002849 | -1.5179 |
| CDR1 | -0.7099 | 5.317889 | -3.3516 | 0.00099 | 0.002851 | -1.5189 |
| CP | 0.9217 | 3.38675 | 3.34874 | 0.001 | 0.002876 | -1.5279 |
| NRIP3 | -0.6331 | 2.554457 | -3.3472 | 0.001 | 0.002889 | -1.5327 |
| CXCR4 | 0.55603 | 4.457265 | 3.33805 | 0.00104 | 0.002968 | -1.5613 |
| MAP1LC3A | -0.5892 | 5.690803 | -3.3364 | 0.00104 | 0.002982 | -1.5665 |
| TNK1 | 0.5337 | 1.12131 | 3.33528 | 0.00105 | 0.002991 | -1.5699 |
| PI3 | 0.69762 | 0.780645 | 3.33321 | 0.00105 | 0.00301 | -1.5764 |
| DLGAP1 | -0.5543 | 4.871225 | -3.3307 | 0.00106 | 0.003031 | -1.5842 |
| PCLO | -0.5268 | 1.95418 | -3.3293 | 0.00107 | 0.003043 | -1.5886 |
| C6orf141 | 0.51356 | 1.0806 | 3.32559 | 0.00108 | 0.003077 | -1.6001 |
| SAMSN1 | 0.53188 | 3.422481 | 3.32323 | 0.00109 | 0.003099 | -1.6074 |
| FOLH1 | -0.7354 | 3.443604 | -3.3213 | 0.0011 | 0.003117 | -1.6136 |
| LTBP4 | 0.54437 | 5.507543 | 3.32032 | 0.0011 | 0.003126 | -1.6165 |
| FMOD | 0.67219 | 2.012729 | 3.31416 | 0.00112 | 0.003183 | -1.6356 |
| IGLC2 | 1.0126 | 1.580486 | 3.31172 | 0.00113 | 0.003205 | -1.6432 |
| RP11-320H14.1 | -0.6934 | 1.871512 | -3.3092 | 0.00114 | 0.003229 | -1.651 |
| MRAP2 | -0.6041 | 1.894444 | -3.3091 | 0.00114 | 0.003229 | -1.6514 |
| A2ML1 | -0.5105 | 1.986141 | -3.3069 | 0.00115 | 0.003248 | -1.6581 |
| LRP2 | -0.6064 | 1.908014 | -3.3034 | 0.00116 | 0.003281 | -1.6689 |
| CDS1 | -0.5258 | 1.818275 | -3.3024 | 0.00117 | 0.003293 | -1.6722 |
| IGLV1-51 | 0.60526 | 1.122662 | 3.29884 | 0.00118 | 0.003328 | -1.683 |
| SEZ6L2 | -0.6689 | 4.958679 | -3.2904 | 0.00121 | 0.003414 | -1.7089 |
| PTER | -0.5912 | 2.104201 | -3.2839 | 0.00124 | 0.003482 | -1.7292 |
| MAP6D1 | -0.565 | 4.539134 | -3.2828 | 0.00125 | 0.003494 | -1.7326 |
| RGR | -0.7601 | 3.270156 | -3.2804 | 0.00126 | 0.003517 | -1.7397 |
| KIRREL3 | -0.5476 | 2.827708 | -3.2708 | 0.0013 | 0.003617 | -1.7692 |
| CCNI2 | -0.6469 | 3.46305 | -3.2651 | 0.00132 | 0.003679 | -1.7869 |
| NIPAL3 | -0.5215 | 4.315172 | -3.2644 | 0.00133 | 0.003686 | -1.7889 |
| KCNE4 | 0.51761 | 2.23068 | 3.26404 | 0.00133 | 0.003689 | -1.79 |
| PTRF | 0.56171 | 4.421664 | 3.26311 | 0.00133 | 0.003699 | -1.7929 |
| AC073479.1 | -0.5035 | 3.117323 | -3.2623 | 0.00133 | 0.003707 | -1.7955 |
| IFI6 | 0.60256 | 7.177602 | 3.26039 | 0.00134 | 0.003726 | -1.8012 |
| ANLN | -0.6683 | 5.066473 | -3.2602 | 0.00134 | 0.003728 | -1.8018 |
| HIP1R | -0.5294 | 6.242824 | -3.2571 | 0.00136 | 0.003762 | -1.8113 |
| EFEMP1 | 0.71487 | 6.500237 | 3.25689 | 0.00136 | 0.003764 | -1.8119 |
| SIGLEC1 | 0.50755 | 1.732256 | 3.25617 | 0.00136 | 0.003771 | -1.8141 |
| KCNAB2 | -0.5478 | 4.669665 | -3.2515 | 0.00138 | 0.003824 | -1.8284 |
| AC062021.1 | -0.8366 | 2.155264 | -3.249 | 0.00139 | 0.003851 | -1.8359 |
| PRDM8 | -0.5761 | 2.775237 | -3.2477 | 0.0014 | 0.003865 | -1.8399 |
| HCN1 | -0.5845 | 1.310641 | -3.2418 | 0.00143 | 0.003928 | -1.8578 |
| B3GAT2 | -0.7103 | 3.769465 | -3.2381 | 0.00145 | 0.003971 | -1.8691 |
| BASP1 | -0.5069 | 6.619029 | -3.2311 | 0.00148 | 0.004043 | -1.8904 |
| PCDHGA3 | -0.572 | 2.221976 | -3.221 | 0.00153 | 0.00416 | -1.9209 |
| FBLN7 | 0.53751 | 1.905467 | 3.20322 | 0.00162 | 0.00438 | -1.9746 |
| LGI4 | -0.6007 | 3.699184 | -3.2018 | 0.00163 | 0.004396 | -1.9789 |
| THNSL2 | -0.6167 | 2.909169 | -3.2005 | 0.00164 | 0.004413 | -1.9829 |
| RYR1 | -0.5265 | 3.40274 | -3.1994 | 0.00164 | 0.004424 | -1.9862 |
| DNAH17 | -0.5308 | 2.608475 | -3.1844 | 0.00172 | 0.004622 | -2.0311 |
| IRX3 | -0.5189 | 2.186324 | -3.1828 | 0.00173 | 0.004643 | -2.036 |
| NOG | -0.6255 | 3.242691 | -3.1795 | 0.00175 | 0.004685 | -2.0457 |
| RP11-61I13.3 | -0.5757 | 2.890396 | -3.1794 | 0.00175 | 0.004686 | -2.046 |
| RP11-495P10.2 | 0.67729 | 2.397128 | 3.17936 | 0.00175 | 0.004686 | -2.0462 |
| TFAP2A | 0.51831 | 2.378936 | 3.17584 | 0.00177 | 0.004729 | -2.0567 |
| IGKV4-1 | 0.58805 | 0.794435 | 3.16038 | 0.00186 | 0.004942 | -2.1028 |
| GGTA1P | -0.5893 | 5.514469 | -3.1598 | 0.00187 | 0.004949 | -2.1046 |
| IGHV5-51 | 0.54234 | 0.715718 | 3.15422 | 0.0019 | 0.005027 | -2.1211 |
| HIST1H4J | 0.53415 | 2.97674 | 3.14596 | 0.00195 | 0.005146 | -2.1456 |
| EFCAB1 | -0.5352 | 1.42514 | -3.1445 | 0.00196 | 0.005168 | -2.1499 |
| CDHR1 | -0.556 | 3.616242 | -3.1411 | 0.00198 | 0.005219 | -2.1601 |
| C9orf24 | -0.7199 | 2.108607 | -3.1404 | 0.00199 | 0.005229 | -2.162 |
| GALNT9 | -0.6757 | 3.227719 | -3.1388 | 0.002 | 0.005252 | -2.1667 |
| LPPR3 | -0.6706 | 3.183171 | -3.1368 | 0.00201 | 0.005281 | -2.1726 |
| TMEM63A | -0.524 | 5.045338 | -3.1361 | 0.00202 | 0.005291 | -2.1747 |
| CADPS2 | -0.5045 | 3.009563 | -3.1359 | 0.00202 | 0.005294 | -2.1753 |
| SLC47A2 | 0.61063 | 1.505132 | 3.12016 | 0.00212 | 0.005536 | -2.2217 |
| KCNV1 | -0.5196 | 1.034828 | -3.1171 | 0.00214 | 0.005589 | -2.2308 |
| CSMD3 | -0.5558 | 2.726046 | -3.1169 | 0.00214 | 0.005591 | -2.2313 |
| RPE65 | 0.67159 | 2.447763 | 3.11448 | 0.00216 | 0.005628 | -2.2384 |
| GABRA3 | -0.6148 | 3.509388 | -3.1138 | 0.00217 | 0.005637 | -2.2403 |
| HPR | -0.7338 | 3.489458 | -3.1091 | 0.0022 | 0.005715 | -2.2543 |
| CDH8 | -0.5094 | 2.30828 | -3.1038 | 0.00224 | 0.005803 | -2.2698 |
| IGFBPL1 | 0.61129 | 1.64093 | 3.09991 | 0.00226 | 0.005865 | -2.2811 |
| HLA-DQA1 | 1.05695 | 3.230809 | 3.09931 | 0.00227 | 0.005873 | -2.2829 |
| MIAT | -0.5606 | 4.134002 | -3.0983 | 0.00228 | 0.00589 | -2.2858 |
| RGS9 | -0.5088 | 3.42172 | -3.0955 | 0.0023 | 0.005934 | -2.2941 |
| DACH2 | -0.5638 | 1.811883 | -3.091 | 0.00233 | 0.006011 | -2.3071 |
| FAM180A | 0.50338 | 1.032425 | 3.08812 | 0.00235 | 0.006059 | -2.3155 |
| IGHG4 | 0.56186 | 0.715736 | 3.08802 | 0.00235 | 0.00606 | -2.3158 |
| CTD-3049M7.1 | 0.81028 | 1.552345 | 3.08251 | 0.00239 | 0.006157 | -2.3319 |
| RHBDL3 | -0.5954 | 4.51061 | -3.0743 | 0.00246 | 0.006294 | -2.3557 |
| POPDC3 | -0.5195 | 2.1872 | -3.0725 | 0.00247 | 0.006321 | -2.3608 |
| FBLN5 | 0.58922 | 3.458855 | 3.06904 | 0.0025 | 0.00638 | -2.371 |
| ICAM5 | -0.6706 | 2.545606 | -3.0595 | 0.00257 | 0.006547 | -2.3986 |
| IGLV1-40 | 0.57594 | 0.854034 | 3.05409 | 0.00262 | 0.006643 | -2.4142 |
| SCN2A | -0.5007 | 3.10106 | -3.0455 | 0.00269 | 0.006804 | -2.439 |
| HIF3A | -0.6066 | 3.150443 | -3.0424 | 0.00272 | 0.006859 | -2.4477 |
| MSTN | 0.7957 | 2.849643 | 3.04071 | 0.00273 | 0.006889 | -2.4527 |
| ANGPTL4 | 0.57994 | 3.494179 | 3.03303 | 0.0028 | 0.00703 | -2.4747 |
| FAM189A2 | -0.5352 | 4.946278 | -3.0233 | 0.00289 | 0.00722 | -2.5025 |
| IFITM3 | 0.54204 | 7.441263 | 3.02111 | 0.00291 | 0.007263 | -2.5089 |
| STOX1 | -0.5389 | 3.380745 | -3.0184 | 0.00293 | 0.00732 | -2.5167 |
| COL22A1 | 0.67333 | 2.133883 | 3.0156 | 0.00296 | 0.007376 | -2.5246 |
| CHRDL1 | -0.5887 | 3.964771 | -3.0115 | 0.00299 | 0.007461 | -2.5363 |
| FAM181A | 0.63199 | 2.471867 | 3.00875 | 0.00302 | 0.007517 | -2.5441 |
| CDK18 | -0.5948 | 5.461312 | -3.0077 | 0.00303 | 0.007539 | -2.5471 |
| HLA-C | 0.72576 | 6.184606 | 3.00329 | 0.00307 | 0.007631 | -2.5596 |
| PCED1B-AS1 | 0.51438 | 3.951027 | 2.99566 | 0.00315 | 0.007792 | -2.5813 |
| CTD-2325P2.4 | -0.6376 | 2.157614 | -2.9949 | 0.00315 | 0.007805 | -2.5836 |
| DES | 0.52752 | 1.251105 | 2.99257 | 0.00318 | 0.007855 | -2.59 |
| PCSK2 | -0.6647 | 3.250326 | -2.9885 | 0.00322 | 0.007941 | -2.6015 |
| PADI2 | -0.6103 | 5.390337 | -2.9862 | 0.00324 | 0.007991 | -2.6081 |
| RSPO2 | -0.5087 | 1.888819 | -2.9799 | 0.0033 | 0.008131 | -2.6259 |
| C1QTNF1 | 0.50436 | 3.916384 | 2.97784 | 0.00332 | 0.008178 | -2.6317 |
| VAT1L | -0.6145 | 4.635228 | -2.9758 | 0.00335 | 0.008224 | -2.6373 |
| CEBPD | 0.55632 | 4.544151 | 2.97192 | 0.00339 | 0.008309 | -2.6484 |
| BSCL2 | -0.5163 | 5.805225 | -2.9689 | 0.00342 | 0.008371 | -2.6568 |
| SLC15A2 | -0.5483 | 4.73729 | -2.9534 | 0.00359 | 0.008728 | -2.7005 |
| GPR37L1 | -0.5026 | 4.941946 | -2.9478 | 0.00365 | 0.008857 | -2.7161 |
| C21orf62 | 0.67508 | 3.2289 | 2.94669 | 0.00366 | 0.008881 | -2.7191 |
| GAP43 | 0.57509 | 7.036955 | 2.93912 | 0.00375 | 0.009065 | -2.7402 |
| TAC1 | -0.765 | 2.255255 | -2.9361 | 0.00378 | 0.009134 | -2.7486 |
| MTND1P23 | -0.7605 | 2.637899 | -2.9325 | 0.00382 | 0.009218 | -2.7586 |
| RALYL | -0.5705 | 2.771694 | -2.9322 | 0.00383 | 0.009222 | -2.7594 |
| AC011558.5 | 0.64686 | 2.235783 | 2.93143 | 0.00384 | 0.00924 | -2.7616 |
| CTC-425F1.4 | 0.51504 | 3.305367 | 2.92828 | 0.00387 | 0.009321 | -2.7704 |
| IFI44L | 0.53985 | 4.157584 | 2.92284 | 0.00394 | 0.009455 | -2.7855 |
| SLC45A3 | -0.5286 | 2.422241 | -2.9198 | 0.00398 | 0.009531 | -2.794 |
| AKR1C3 | -0.5393 | 4.700311 | -2.9173 | 0.00401 | 0.009591 | -2.8009 |
| IGFBP3 | 0.79082 | 4.386342 | 2.91437 | 0.00404 | 0.009666 | -2.8089 |
| SEZ6L | -0.729 | 5.675631 | -2.9091 | 0.00411 | 0.009801 | -2.8235 |
| BTF3L4P2 | 0.57743 | 2.671543 | 2.90893 | 0.00411 | 0.009804 | -2.824 |
| CNTN1 | -0.5746 | 6.55012 | -2.9055 | 0.00415 | 0.009898 | -2.8334 |
| SLC6A11 | -0.6002 | 3.48152 | -2.8918 | 0.00433 | 0.010264 | -2.8713 |
| PLA2G16 | -0.52 | 6.051723 | -2.8904 | 0.00435 | 0.010303 | -2.8749 |
| MARVELD3 | -0.5667 | 2.193256 | -2.8779 | 0.00451 | 0.010647 | -2.9093 |
| GABRA2 | -0.6253 | 2.767803 | -2.8772 | 0.00452 | 0.010667 | -2.9112 |
| KIF19 | -0.5212 | 1.800945 | -2.875 | 0.00455 | 0.010718 | -2.917 |
| ACY3 | -0.5779 | 2.145783 | -2.8742 | 0.00457 | 0.010743 | -2.9194 |
| IGJ | 0.57033 | 1.161412 | 2.87355 | 0.00457 | 0.010758 | -2.9211 |
| IP6K3 | -0.5239 | 2.121824 | -2.8688 | 0.00464 | 0.010895 | -2.9342 |
| CDH18 | -0.5218 | 2.574524 | -2.8605 | 0.00476 | 0.011132 | -2.9567 |
| IGKV3-20 | 0.71541 | 1.117183 | 2.85052 | 0.0049 | 0.011424 | -2.9837 |
| PCDHB8 | 0.52515 | 2.018266 | 2.84911 | 0.00492 | 0.011468 | -2.9876 |
| MICAL2 | -0.5496 | 3.59429 | -2.8299 | 0.00521 | 0.012055 | -3.0395 |
| AQP4 | -0.6349 | 8.037838 | -2.8198 | 0.00537 | 0.012386 | -3.0667 |
| RP1-111D6.3 | -0.7569 | 3.803636 | -2.8184 | 0.0054 | 0.012431 | -3.0705 |
| IGHG3 | 0.74267 | 1.188358 | 2.81764 | 0.00541 | 0.012455 | -3.0724 |
| TAC3 | -0.6639 | 1.876491 | -2.8168 | 0.00542 | 0.012483 | -3.0748 |
| MAN1C1 | 0.54433 | 4.246678 | 2.81546 | 0.00544 | 0.01252 | -3.0783 |
| WIF1 | -0.6981 | 1.520013 | -2.8043 | 0.00563 | 0.012892 | -3.1081 |
| SMOC1 | -0.8213 | 7.08397 | -2.7925 | 0.00583 | 0.013292 | -3.1397 |
| FOS | 0.66241 | 6.977721 | 2.78113 | 0.00603 | 0.013677 | -3.1698 |
| SLC31A2 | -0.5219 | 4.317077 | -2.7775 | 0.00609 | 0.013798 | -3.1794 |
| RP3-437C15.1 | 0.87356 | 3.019875 | 2.77498 | 0.00614 | 0.013887 | -3.1861 |
| ATCAY | -0.6476 | 5.3946 | -2.7733 | 0.00617 | 0.013953 | -3.1906 |
| C4B | 0.65963 | 6.526798 | 2.77173 | 0.00619 | 0.014009 | -3.1946 |
| OSM | 0.53401 | 1.509643 | 2.76917 | 0.00624 | 0.0141 | -3.2014 |
| C4A | 0.64704 | 6.879988 | 2.75288 | 0.00655 | 0.014696 | -3.2443 |
| NSG1 | -0.5715 | 4.596296 | -2.7443 | 0.00671 | 0.015031 | -3.2669 |
| SLC38A1 | -0.5123 | 4.985856 | -2.726 | 0.00708 | 0.015744 | -3.3146 |
| HAPLN1 | -0.6885 | 3.631439 | -2.7169 | 0.00727 | 0.016119 | -3.3383 |
| UGT8 | -0.5911 | 5.170853 | -2.7148 | 0.00731 | 0.0162 | -3.3436 |
| ARC | 0.56877 | 3.592436 | 2.71291 | 0.00735 | 0.016279 | -3.3486 |
| AEBP1 | 0.67893 | 5.431486 | 2.71253 | 0.00736 | 0.016295 | -3.3495 |
| IGHV3-23 | 0.5029 | 0.762848 | 2.71192 | 0.00737 | 0.016318 | -3.3511 |
| CCL2 | 0.80412 | 5.219644 | 2.70969 | 0.00742 | 0.016401 | -3.3569 |
| ETV1 | 0.58946 | 6.361834 | 2.69926 | 0.00765 | 0.016849 | -3.3838 |
| DDIT4L | 0.5192 | 1.365827 | 2.67989 | 0.00808 | 0.017681 | -3.4336 |
| FABP5 | 0.75706 | 5.055273 | 2.67823 | 0.00812 | 0.017751 | -3.4379 |
| ELOVL2 | -0.5573 | 3.736072 | -2.6686 | 0.00835 | 0.01818 | -3.4626 |
| APOC2 | 0.54355 | 5.547955 | 2.66057 | 0.00854 | 0.018559 | -3.483 |
| GRIN2A | -0.5031 | 2.034239 | -2.6513 | 0.00877 | 0.018979 | -3.5064 |
| IGKV1D-39 | 0.67494 | 1.048332 | 2.64945 | 0.00882 | 0.019068 | -3.5112 |
| TRIM67 | -0.68 | 2.127049 | -2.638 | 0.00911 | 0.019596 | -3.5402 |
| GAD1 | -0.5329 | 4.565004 | -2.6253 | 0.00944 | 0.020209 | -3.5721 |
| DHRS9 | -0.5058 | 2.709071 | -2.615 | 0.00972 | 0.02072 | -3.5981 |
| CAPG | 0.51057 | 5.474204 | 2.59786 | 0.0102 | 0.02159 | -3.6408 |
| VSIG4 | 0.5154 | 5.293889 | 2.59217 | 0.01036 | 0.021887 | -3.655 |
| IGKV3-11 | 0.5378 | 0.755405 | 2.58073 | 0.0107 | 0.022504 | -3.6834 |
| RBP7 | -0.5101 | 2.794628 | -2.5584 | 0.01138 | 0.023754 | -3.7384 |
| EGR2 | 0.59635 | 3.378253 | 2.53506 | 0.01214 | 0.025111 | -3.7955 |
| CA10 | -0.6581 | 4.012581 | -2.5276 | 0.01239 | 0.025561 | -3.8136 |
| FGFR2 | -0.5157 | 4.852079 | -2.5191 | 0.01268 | 0.026103 | -3.8342 |
| PDLIM4 | 0.5636 | 3.151136 | 2.49529 | 0.01353 | 0.027641 | -3.8915 |
| PEG3 | -0.5075 | 4.26949 | -2.4946 | 0.01356 | 0.027675 | -3.8932 |
| RP11-82L18.4 | -0.5304 | 3.869108 | -2.4906 | 0.01371 | 0.02793 | -3.9027 |
| HP | 0.62582 | 1.926201 | 2.47321 | 0.01437 | 0.029104 | -3.9443 |
| RPL39P3 | 0.85697 | 4.111722 | 2.46679 | 0.01462 | 0.029539 | -3.9595 |
| RP11-161H23.5 | 0.63179 | 1.606418 | 2.46458 | 0.01471 | 0.029686 | -3.9648 |
| SLX1B | 0.53239 | 2.518648 | 2.45068 | 0.01527 | 0.030675 | -3.9976 |
| NPTX2 | -0.6082 | 3.001319 | -2.4287 | 0.01619 | 0.032293 | -4.0492 |
| RGS1 | 0.68357 | 5.046292 | 2.42763 | 0.01624 | 0.032368 | -4.0518 |
| HBB | 0.59955 | 7.425995 | 2.42727 | 0.01625 | 0.032385 | -4.0526 |
| RPS17 | 0.54018 | 7.462369 | 2.42721 | 0.01625 | 0.032388 | -4.0528 |
| TMEM179 | -0.5152 | 3.842403 | -2.4237 | 0.01641 | 0.032644 | -4.0609 |
| GALNT13 | -0.5532 | 4.943803 | -2.3955 | 0.01768 | 0.034832 | -4.1264 |
| CCL3L3 | -0.7132 | 3.415225 | -2.394 | 0.01775 | 0.034946 | -4.13 |
| BCAS1 | -0.6068 | 6.149885 | -2.3836 | 0.01824 | 0.035788 | -4.1539 |
| C1QL1 | 0.61178 | 7.210021 | 2.37371 | 0.01872 | 0.03661 | -4.1765 |
| PCP4 | -0.5627 | 3.546152 | -2.3731 | 0.01875 | 0.036655 | -4.178 |
| IRX1 | 0.58462 | 2.170109 | 2.36522 | 0.01914 | 0.037301 | -4.1959 |
| RPRM | -0.5296 | 3.929739 | -2.3495 | 0.01994 | 0.038609 | -4.2317 |
| VEPH1 | -0.5308 | 2.787763 | -2.3244 | 0.02128 | 0.04082 | -4.2884 |
| RP11-82C23.2 | -0.5463 | 1.501812 | -2.3157 | 0.02176 | 0.041666 | -4.3079 |
| TPPP3 | -0.5605 | 5.538296 | -2.2835 | 0.02363 | 0.044815 | -4.3794 |
| EGR1 | 0.56759 | 5.870414 | 2.23786 | 0.02652 | 0.049449 | -4.4792 |
| GPNMB | 0.60555 | 4.42098 | 2.23335 | 0.02682 | 0.04992 | -4.4889 |
