# Supplementary material for: CIA‐II is associated with lower‐grade glioma survival and cell proliferation
Source: CNS Neurosci Ther. 2023 Jul 14;30(2):e14340. doi: 10.1111/cns.14340 (PMC10848044; doi:10.1111/cns.14340)
Supplement: Supplementary file 11 — Data S2. [file CNS-30-e14340-s006.docx]

**Figure legends**

**Figure S1.** Analysis of the correlation between CIA-II and the clinical characteristics of patients with LGG. **(A)** The relationship between CIA-II expression and the clinical characteristics of LGG in CGGA dataset. **(B)** Analysis of the correlation between CIA-II expression and clinical characteristics in CGGA dataset. **(C)** Kaplan–Meier analysis showing the differences in OS between the low-CIA-II and high-CIA-II subtypes associated with important clinical characteristics (such as WHO grade, IDH status, and age) of patients with LGG in CGGA cohort (**P* < 0.05, ***P* < 0.01, ****P* < 0.001).

**Figure S2. (A-B)** Detailed proportion of the clinical features of patients with LGG between the low-CIA-II and high-CIA-II subgroups in TCGA **(A)** and CGGA **(B)** cohorts. **(C)** Kaplan–Meier analysis showing the differences in OS between the low-CIA-II and high-CIA-II subtypes associated with important clinical characteristics (such as WHO grade, IDH status, and age) of patients with LGG in TCGA cohort (**P* < 0.05, ***P* < 0.01, ****P* < 0.001).

**Figure S3.** Cox regression analyses and nomogram model of patients with LGG. **(A-B)** Univariate and multivariate Cox regression analyses of the clinicopathological features and CIA-II expression in the cohorts from TCGA **(A)** and CGGA **(B)**. (**C**) Nomogram model established with WHO grade, 1p/19q status, and CIA-II expression in TCGA cohort**. (D-E)** Calibration plots showing the accuracy of the clinical nomogram model in predicting 1-, 3-, and 5-year OS in patients with LGG in the TCGA **(D)** and CGGA cohorts **(E)**.

**Figure S4.** Distinct tumor immune microenvironment (TIME) and immunological features of the low-CIA-II and high-CIA-II subtypes in CGGA dataset. **(A)** Differences in immune-associated functions between the low-CIA-II and high-CIA-II subtypes. **(B)** Comparisons of the ESTIMATE, stromal, immune scores, and tumor purity between the low-CIA-II and high-CIA-II subgroups. **(C)** Comparisons of the abundances of 22 types of immune cells in the low-CIA-II and high-CIA-II subtypes. **(D)** Lollipop plots showing the association between CIA-II expression and TIICs. **(E)** Detailed analysis of the correlation between CIA-II expression and TIICs (**P* < 0.05, ***P* < 0.01, ****P* < 0.001).

**Figure S5.** Different responses to chemotherapy of the low-CIA-II and high-CIA-II subtypes in the TCGA dataset.
